# Abstracts from the 11th Annual Meeting of Arthroplasty Society In Asia (ASIA), the 4th Annual Meeting of the Malaysian Society for Hip and Knee Surgeons (MSHKS), and the 17th Annual Meeting of the ASEAN Arthroplasty Association (AAA)

**DOI:** 10.1186/s42836-026-00376-9

**Published:** 2026-03-26

## O1 Clinical efficacy analysis of parallel osteotomy combined with “prosthetic spacer” in stage IV septic arthritis of knee

### Liqiang Zhi, Xiangxiang Sun, Tao Ma, Jun Wang, Jianbing Ma

#### Department of Joint Surgery, Honghui Hospital, Xi’an Jiaotong University, Xi’an, China

##### **Correspondence:** Liqiang Zhi (zhiliqiang2011@126.com)

*Arthroplasty 2026*, **8(1):**O1

**Objective**

To evaluate the clinical efficacy of the parallel osteotomy combined with a “prosthetic spacer” in the treatment of Stage IV septic arthritis (SA) of the knee, and to provide a staged treatment strategy that effectively controls infection while maximizing joint function preservation.

**Methods**

A prospective analysis was conducted on 23 patients diagnosed with knee SA at the joint department of Xi’an Honghui Hospital from 2020.9 to 2023.9. All patients underwent parallel osteotomy with a “joint spacer”, categorized into a prosthetic group (12 cases, using CR femoral condyles and tibial polyethylene liners) and a bone cement group (11 cases, using cement-molded spacers). operative time, blood loss, preoperative/postoperative pain VAS scores, postoperative KSS scores, range of motion (ROM), 1-year infection recurrence rates, and secondary surgery rate were recorded. Data were analyzed using SPSS 21.0 (*P* < 0.05 considered statistically significant) (Figs. 1 and 2).

**Results**

The prosthetic group demonstrated significant improvements in operative time, blood loss, postoperative VAS scores for pain, KSS scores, and ROM compared to the bone cement group (*P* < 0.05). Both groups achieved comparable infection control rates (*P* > 0.05), and the prosthetic group had a lower rate of secondary surgery (*P* < 0.05).

**Conclusion**

The parallel osteotomy combined with a “prosthetic spacer” is a viable option for severe knee SA. This approach effectively controls infection and preserves joint function, warranting broader clinical adoption.

**Fig. 1 (Abstract O1).**
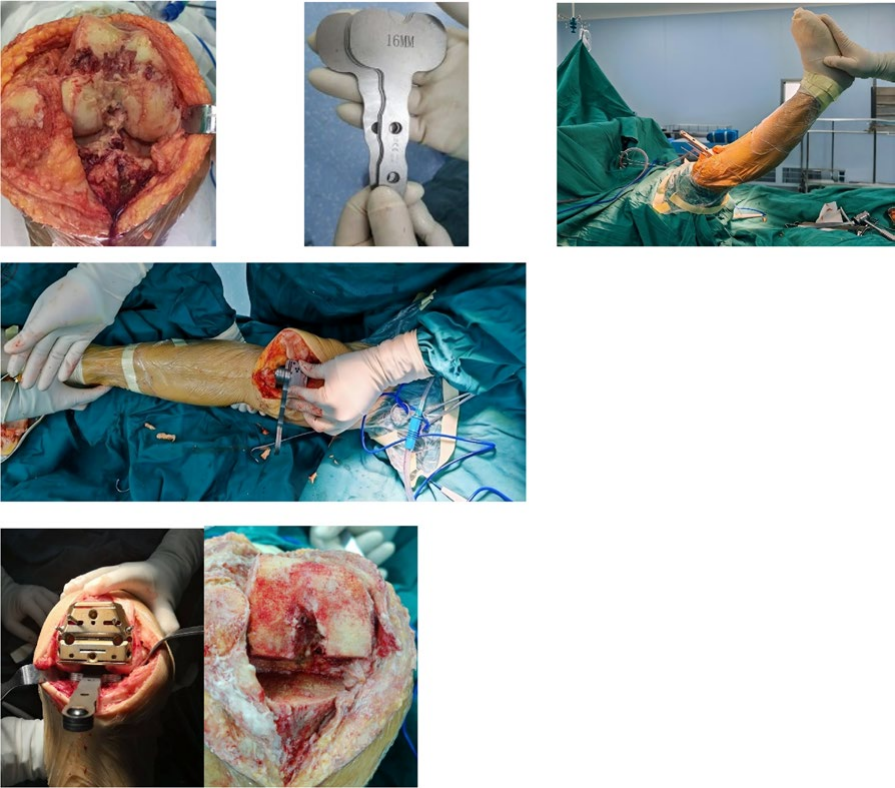
The specific operation process and steps of parallel osteotomy. In Fig. A, it can be seen that SAK has invaded the cartilage, and there is obvious joint destruction. Fig. B, self-made parallel osteotomy; Fig. C-E. Parallel osteotomy is used to make flexion and extension Spaces. Fig. F, complete effect of osteotomy

**Fig. 2 (Abstract O1).**
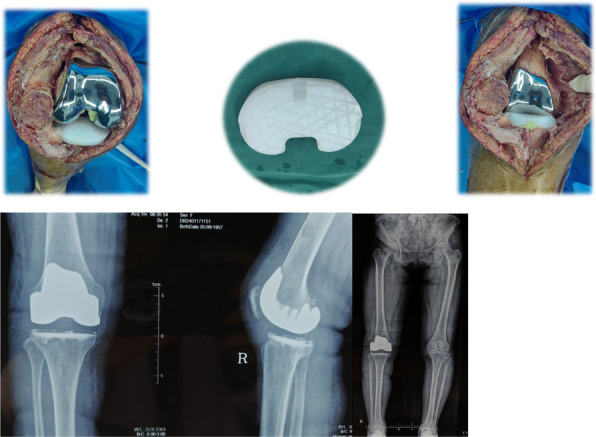
The process of performing the prosthetic spacer and postoperative X-ray

## O2 Primary total knee arthroplasty for Schatzker type II/III tibial plateau fractures: a comprehensive outcome analysis

### Xiangxiang Sun, Liqiang Zhi, Jianbing Ma

#### Department of Joint Surgery, Honghui Hospital, Xi’an Jiaotong University, Xi’an, China

##### **Correspondence:** Xiangxiang Sun (zeltasun@163.com)

*Arthroplasty 2026*, **8(1):**O2

**Objective**

To investigate the feasibility, technical strategies, and clinical advantages of primary total knee arthroplasty (TKA) in treating elderly patients with Schatzker type II/III tibial plateau fractures complicated by osteoarthritis, offering a novel approach to address the high complication and reoperation rates associated with traditional open reduction internal fixation (ORIF).

**Methods**

A prospective analysis was conducted on 15 elderly patients with Schatzker II/III fractures and concomitant osteoarthritis treated at the Department of Joint Surgery, Honghui Hospital in Xi’an (October 2021–March 2023). All patients underwent strict preoperative evaluation (imaging analysis of fracture extent, bone defect type, and osteoporosis severity) followed by single-stage TKA. The standardized surgical protocol included: 1. Anteromedial parapatellar approach with osteotomy level adjusted based on tibial tubercle integrity. 2. Three-step tibial baseplate reconstruction combined with cement fixation and stem extension for stability. 3. Constraint-level prosthesis selection guided by ligament status, with concurrent bone defect management (autograft/tumor prosthesis augmentation). 4. Accelerated rehabilitation: Partial weight-bearing within 1–3 days and full weight-bearing at 4 weeks postoperatively. Postoperative outcomes (VAS, HSS scores, ambulation time, thrombosis rates, prosthesis loosening, and patient satisfaction) were systematically evaluated.

**Results**

All 15 patients completed a 1-year follow-up. Compared to historical ORIF data, the primary TKA group demonstrated accelerated functional recovery, achieving 100% partial weight-bearing by the postoperative 3rd day. 92% achieved full weight-bearing at 4 weeks. 70% reduction in immobilization-related complications (DVT, pneumonia). Significant pain relief: VAS decreased from 7.5 ± 1.2 preoperatively to 1.8 ± 0.6 at 3 months. HSS improved from 45 ± 10.2 to 88 ± 6.4. Avoidance of secondary surgeries: 95% patient satisfaction with single-stage fracture-arthritis resolution. Long-term stability (postoperative 1 year): 0% prosthesis loosening rate. No infections or mechanical failures.

**Conclusions**

Primary TKA provides a definitive single-stage solution for elderly patients with Schatzker II/III fractures and preexisting osteoarthritis, achieving simultaneous fracture reduction, joint reconstruction, and rapid rehabilitation. This approach significantly reduces complications, shortens hospitalization, and enhances quality of life. Future studies should validate long-term outcomes in larger cohorts and refine prosthetic designs for complex bone defects.

**Table 1 (Abstract O2).** Primary TKA vs. traditional ORIF

	**Primary TKA Group (*n* = 15)**	**Traditional ORIF Group (Reference Values)**	***P*-value**
Weight-bearing time(day)	3 ± 0.3	42.1 ± 3.9	**< 0.05**
VAS pain score	Pre-op: 7.5 ± 1.2 → 3mo: 1.8 ± 0.6	3mo: 4.2 ± 1.5	
HSS functional score	Pre-op: 45 ± 10 → 3mo: 88 ± 6	3mo: 70 ± 12	
Thrombosis incidence(%)	12	25–35%	
Reoperation rate(%)	0	5%	
Prosthesis loosening rate (1-year follow-up)(%)	0	10	
Patient satisfaction	95	75%	

## O3 Risk analysis of reoperation after primary total hip arthroplasty

### Lin Li^1,2^, Lee Khuan^2^, Aidalina Mahmud^3^, Li Yang^1^, Poh Ying Lim^3^

#### ^1^Department of Nursing, The First Affiliated Hospital of Guangxi Medical University, Nanning, China; ^2^Department of Nursing, Faculty of Medicine & Health Sciences, Universiti Putra Malaysia, Selangor, Malaysia; ^3^Department of Community Health, Faculty of Medicine & Health Sciences, Universiti Putra Malaysia, Selangor, Malaysia; ^4^Department of Nursing, The First Affiliated Hospital of Guangxi Medical University, Nanning, China

##### **Correspondence:** Poh Ying Lim (pohying_my@upm.edu.my)

*Arthroplasty 2026*, **8(1):**O3

**Background**

Total hip arthroplasty (THA) is the preferred treatment for severe hip osteoarthritis; however, some patients require reoperation after surgery, imposing substantial physical, psychological, and economic burdens. Identifying risk factors for reoperation can help mitigate these outcomes. This study aims to analyze reoperation risk after primary THA.

**Methods**

This study is retrospective. Patients who underwent primary THA between 2000 and 2024 at a Chinese hospital in Guangxi were selected randomly based on inclusion and exclusion criteria. This study utilized a probability sampling approach, incorporating a random number table to streamline the patient selection procedure. Bivariate analysis was used to explore the association between independent variables and reoperation (yes or no). Simple logistic regression and multiple logistic regression were used to find factors associated with a higher probability of reoperation.

**Results**

A total of 852 patients were included in the study, comprising 279 (32.75%) reoperation cases and 573 (67.25%) without reoperation cases. The median follow-up duration was 5.75 (6.47) years, from 0 to 25 years. In total, 112 independent variables were collected to find whether they have a relationship with reoperation or not. Final model demonstrated that nationality, education level, drinking history, surgery duration, preoperative albumin (ALB), postoperative erythrocyte sedimentation rate (ESR), postoperative calcium levels, clothing (activities daily living assessment preoperative), decorate (activities daily living assessment postoperative), and postoperative thrombosis risk scores associated with a higher probability of reoperation.

**Conclusions**

This study identified critical risk factors associated with reoperation after primary THA. The findings provide valuable insights for healthcare providers to develop targeted interventions aimed at reducing reoperation rates, ultimately improving patient outcomes.

## O4 Application of 3D printing integrated acetabular prosthesis in the treatment of hip dysplasia in total hip arthroplasty

### Liangliang Cheng, Jiawei Ying, Dewei Zhao

#### Affiliated Zhongshan Hospital of Dalian University, Dalian, China

##### **Correspondence:** Dewei Zhao (zhaodewei2016@163.com)

*Arthroplasty 2026*, **8(1):**O4

The full article of this study has been published online. Please refer to the full text at: 10.1016/j.arth.2025.05.024.

## O5 Arthrocentesis in guiding hip and knee arthroplasty surgeons: analysis of data from a national arthroplasty referral centre

### Kharthik Deepan Murugesu, Khairul Anwar Ayob, Veenesh V. Selvaratnam, Loh Kwong Weng, Azlina Amir Abbas

#### Joint Replacement Unit, Orthopedic Department, University Malaya Medical Centre, Petaling Jaya, Malaysia

##### **Correspondence:** Kharthik Deepan Murugesu (kharthikdm6@gmail.com)

*Arthroplasty 2026*, **8(1):**O5

**Background**

Arthrocentesis is an aspiration of synovial fluid for diagnostic or therapeutic purposes. At the University of Malaya Medical Centre (UMMC), our team routinely conducts arthrocentesis in the preoperative setting, particularly in patients presenting with knee effusion, atypical hip pain, or requiring revision arthroplasty. In this case series, we present an 8-month review of arthrocentesis procedures performed at our centre and their impact on subsequent clinical decision-making and surgical management strategies.

**Methods**

Patients’ data were collected from the operation theatre lists, clinic, and ward registries for arthrocentesis procedures performed outside the OT setting, spanning the period from June 2024 to January 2025.

**Results**

The overall positive culture yield from arthrocentesis was 47%, with 71% of those originating from knee aspirations and 39% from hip aspirations. The most frequently isolated pathogen was MSSA, predominantly from knee aspirates. In contrast, hip aspirates yielded more antibiotic-resistant organisms at our center. Table 1 shows that PJI has the highest number of positive preoperative and intraoperative culture findings. Table 2 illustrates CUMARS’ preference for hip revision surgery, while DAIR is preferred for knee revision surgeries.

**Conclusions**

The findings of our case series underscore the importance of synovial fluid analysis in guiding surgical management decisions and tailoring antibiotic strategies, including intraoperative cement loading. A significant proportion of infections involved multidrug-resistant organisms, highlighting the need for continued vigilance and institution-specific antimicrobial stewardship.

**Table 1 (Abstract O5).** Tabulation of data comparing preoperative arthrocentesis findings to intraoperative findings based on diagnosis

Site		Diagnosis		Preoperative Arthrocentesis Culture		Intraoperative Culture	
				**Positive**	**Negative**	**Positive**	**Negative**
Hip	12	Prosthetic Joint Infection (PJI)	4	3	1	3	1
		Avascular Necrosis (AVN)	2	0	2	0	2
		Aseptic Loosening	2	0	2	0	2
		Non-Union	1	0	1	0	1
		Exacerbation of Osteoarthritis	1	0	1	0	1
		Septic Arthritis	2	1	1	-	-
Knee	18	Prosthetic Joint Infection (PJI)	12	10	2	11	1
		Avascular Necrosis (AVN)	1	0	1	0	1
		Aseptic Loosening	1	0	1	1	0
		Non-Union	1	0	1	0	1
		Exacerbation of Osteoarthritis	3	0	3	0	3
		Septic Arthritis	0	0	0	0	0

**Table 2 (Abstract O5).** Tabulation of data for culture-positive arthrocentesis based on the diagnosis, type of surgery, organism isolated, and the antibiotic used in the implant

**Site**	**Diagnosis**		**Type of Surgery**		**Organism Isolated**		**Antibiotic in Implant**
Hip	Prosthetic Joint Infection (PJI)	3	Custom-Made Articulating Spacer (CUMARS)	2	*Corynebacterium striatum*	1	Nil
					*Staphylococcus epidermidis*	1	Nil
			2-stage revision	1	*Candida Glabrata, Enterococcus Faecalis, Staphylococcus Aureus*	1	Voriconazole, Amphotericin B, Gentamicin, Vancomycin
	Septic Arthritis	1	Joint Washout	1	Mycobacterium tuberculosis complex	1	Nil
Knee	Prosthetic Joint Infection (PJI)	10	Debridement, Antibiotics, and Implant Retention (DAIR)	6	Methicillin Sensitive *Staphylococcus Aureus* (MSSA)	5	Nil
					*Escherichia coli* (E. Coli)	1	Nil
			2-stage revision	4	MSSA	1	Ceftadizine, Vancomycin, Gentamycin
					E.Coli	1	Ceftadizine, Vancomycin, Gentamycin
					*Serratia Marcescen*	1	Meropenem, Vancomycin, Gentamycin
					Methicillin-Resistant *Staphylococcus Aureus* (MRSA)	1	Vancomycin, Gentamicin

## O6 Nanozyme-engineered polyethylene for postponing wear particles-induced osteolysis

### Liming Zheng^1,2,3^, Shujie Liu^2^, Qing Jiang^3^, Hui Wei^2^

#### ^1^Department of Orthopedic Surgery, the Second Affiliated Hospital, Zhejiang University School of Medicine, Hangzhou, China; ^2^Department of Biomedical Engineering, College of Engineering and Applied Sciences, Nanjing National Laboratory of Microstructures, Jiangsu Key Laboratory of Artificial Functional Materials, Nanjing University, Nanjing, China; ^3^State Key Laboratory of Pharmaceutical Biotechnology, Division of Sports Medicine and Adult Reconstructive Surgery, Department of Orthopedic Surgery, Nanjing Drum Tower Hospital, The Affiliated Hospital of Nanjing University Medical School, Nanjing, China

##### **Correspondence:** Liming Zheng (limzheng@zju.edu.cn); Qing Jiang (qingj@nju.edu.cn); Hui Wei (huiwei@nju.edu.cn)

*Arthroplasty 2026*, **8(1):**O6

**Background**

Arthroplasty is a successful operation for treating end-stage joint disorders, while improving the longevity of prostheses remains a challenge. As the most common long-term failure cause of arthroplasty, the pathological mechanisms of aseptic loosening related to the wear debris-induced periprosthetic osteolysis (WPO) are still unclear. As the leading cause of long-term failure, the pathological mechanisms underlying aseptic loosening, particularly wear debris-induced periprosthetic osteolysis (WPO), remain unclear despite continuous advancements in mechanical improvements.

Nanozyme is a series of nanoparticles with sustained biological activity. To address WPO, we propose an innovative “proactive preventive” strategy by using nanozymes to modify ultra-high molecular weight polyethylene (UHMWPE). This approach aims to simultaneously reduce the generation of wear particles and minimize their immunostimulatory effects. The key innovation lies in the dual functionality of nanozyme-modified UHMWPE: it enhances wear resistance without compromising tribological properties, while also serving as a drug delivery system for localized anti-inflammatory agent release.

**Methods**

We synthesized a model system by incorporating ceria nanozymes into UHMWPE through a surface modification process. The material characterization was performed using XRD, FT-IR, and tensile testing to evaluate changes in crystallinity, chemical structure, and mechanical properties. To assess the anti-osteolysis effect of CZPE, we established a distal femoral implant model in ICR mice using a standardized surgical technique. Radiological evaluation via microCT and histopathological analysis was conducted at predetermined time points to monitor bone remodeling and osteolysis progression. To explore the underlying mechanisms, RNA-sequencing was employed to identify differentially expressed genes related to inflammation and bone metabolism. These findings were further validated using in vivo studies with our animal model. Finally, inhibition and overexpression of plasma cells were performed in the animal models.

**Results**

Our findings demonstrate that CZPE significantly reduces WPO. As illustrated in Fig. 1, mechanical assessments reveal a statistically significant 32% reduction in wear particle production compared to unmodified ultra-high molecular weight polyethylene (*P* < 0.05). Furthermore, as shown in Figs. 2 and 3, CZPE exhibits superior anti-osteolysis properties in vivo, with minimal trabecular bone loss and reduced inflammatory infiltrates compared to conventional PE. RNA sequencing analysis identifies macrophage-mediated inflammation and diminished intramedullary plasma cell infiltration as key contributors to the pathological process of WPO. Interestingly, treatment with bortezomib significantly enhances the therapeutic effect by inhibiting plasma cell activity. While supplementation with U266B1 cells was found to counteract these benefits, highlighting the contribution of humoral immune modulation in this context.

**Conclusions**

These results underscore the potential of CZPE as a promising material for prosthetic applications, offering enhanced durability through reduced wear particle production and improved management of inflammatory responses. Our study provides valuable insights into the development of advanced biomaterials for orthopedic implants, with implications for improving patient outcomes.

**Fig. 1 (Abstract O6).**
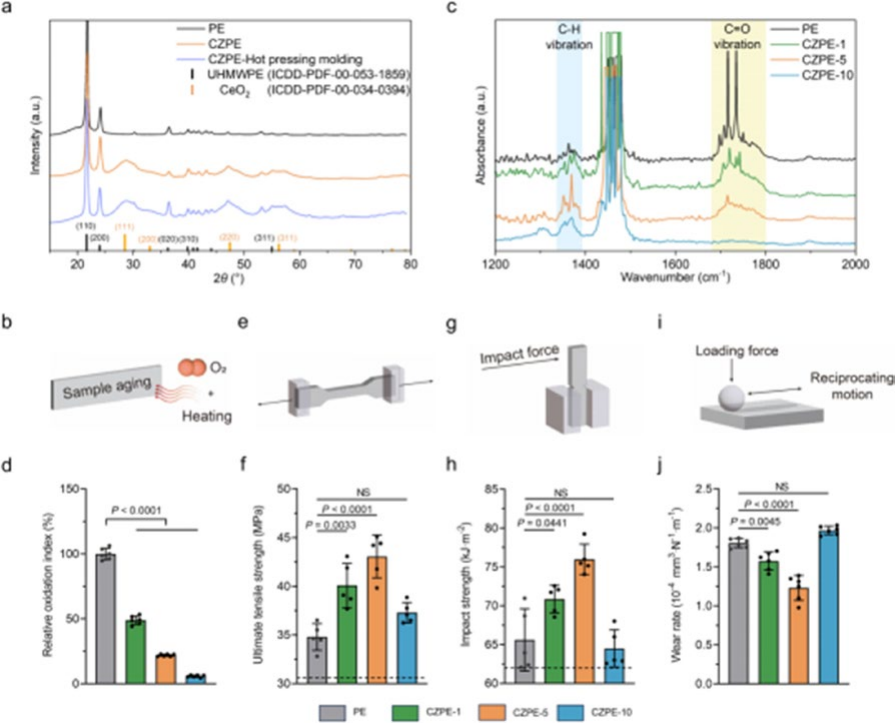
Characterizations of CZPE bulk samples. **a** XRD patterns of UHMWPE and CZPE before and after hot pressing. **b** Scheme of oxidative aging treatment. **c** FT-IR spectra of CZPE-1, CZPE-5, CZPE-10, and PE bulk samples after four months of aging treatment. **d** Relative oxidation indices of CZPE-1, CZPE-5, CZPE-10, and PE bulk samples after four months of aging (*n* = 6). **e** Scheme of tensile testing. **f** Ultimate tensile strength of CZPE-1, CZPE-5, CZPE-10, and PE bulk samples (*n* = 5). The dotted line indicates the minimum value of clinically used UHMWPE. **g** Scheme of notched Izod impact testing. **h** Impact strengths of CZPE-1, CZPE-5, CZPE-10, and PE bulk samples (*n* = 5). The dotted line indicates the minimum value of clinically used UHMWPE. **i** Scheme of friction testing. **j** Wear rates of CZPE-1, CZPE-5, CZPE-10, and PE bulk samples (*n* = 6). Data in **d**, **f**, **h** and **j** are presented as mean ± standard deviation (s.d.). *P* values were analysed by one-way analysis of variance (ANOVA) with Tukey’s multiple comparisons test. NS, not significant, *P* ≥ 0.05

**Fig. 2 (Abstract O6).**
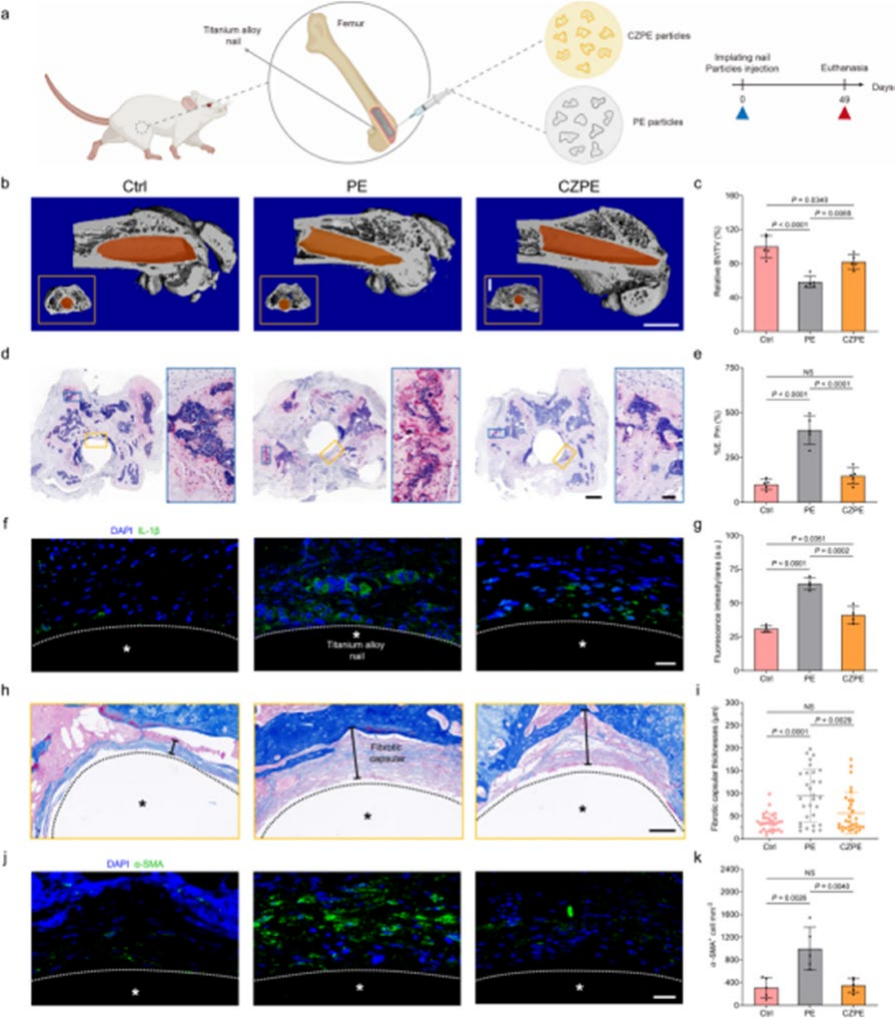
Reduced WPO-inducing potential of CZPE particles. **a** Schematic illustration and timeline of the distal femoral implant model. **b** Representative micro-CT images of the femoral transverse section at day 49 post-injection. Main images: femoral cross-section parallel to the direction of nail insertion. Insets: femoral cross-section perpendicular to the direction of nail insertion. Scale bar, 1 mm. **c** Quantifications of relative BV/TV corresponding to data in **b** (*n* = 5). **d** Representative histological images of TRAP-stained bone sections at day 49 post-surgery. Left: 3 × magnification, scale bar, 500 µm; Right: 48 × magnification, scale bar, 50 µm. **e** Quantifications of %Er. Pm corresponding to data in d (*n* = 5). **f** Representative immunofluorescence staining of femoral sections from experimental mice at day 49 post-surgery. Green indicates IL-1β; blue indicates DAPI nuclear stain. Scale bar, 200 µm. **g** Intensity quantification of IL-1β immunofluorescence staining corresponding to data in **f** (*n* = 4). **h** Representative histochemical images of femoral sections stained with Masson’s trichrome for collagen at day 49 post-surgery, showcasing areas with the most severe foreign body reactions across all groups. The areas of fibrotic capsules were marked with black line segments. Scale bar, 100 µm. **i** Fiber capsule thickness determined by measuring collagen thickness surrounding the titanium alloy nails using Masson’s trichrome staining (*n* = 5). **j** Representative immunofluorescence images for DAPI and α-SMA co-staining. Green indicates α-SMA; blue indicates DAPI nuclear stain. Scale bar, 20 µm. **k** The quantification of α-SMA^+^ cells per unit area. Data in **c**, **e**, **g**, **i** and **k** are presented as mean ± s.d. *P* values for **c**, **e**, **g**, **i** and **k** were analyzed by one-way ANOVA with Tukey’s multiple comparisons test. NS, not significant, *P* ≥ 0.05. Asterisk denotes titanium alloy bone nail

**Fig. 3 (Abstract O6).**
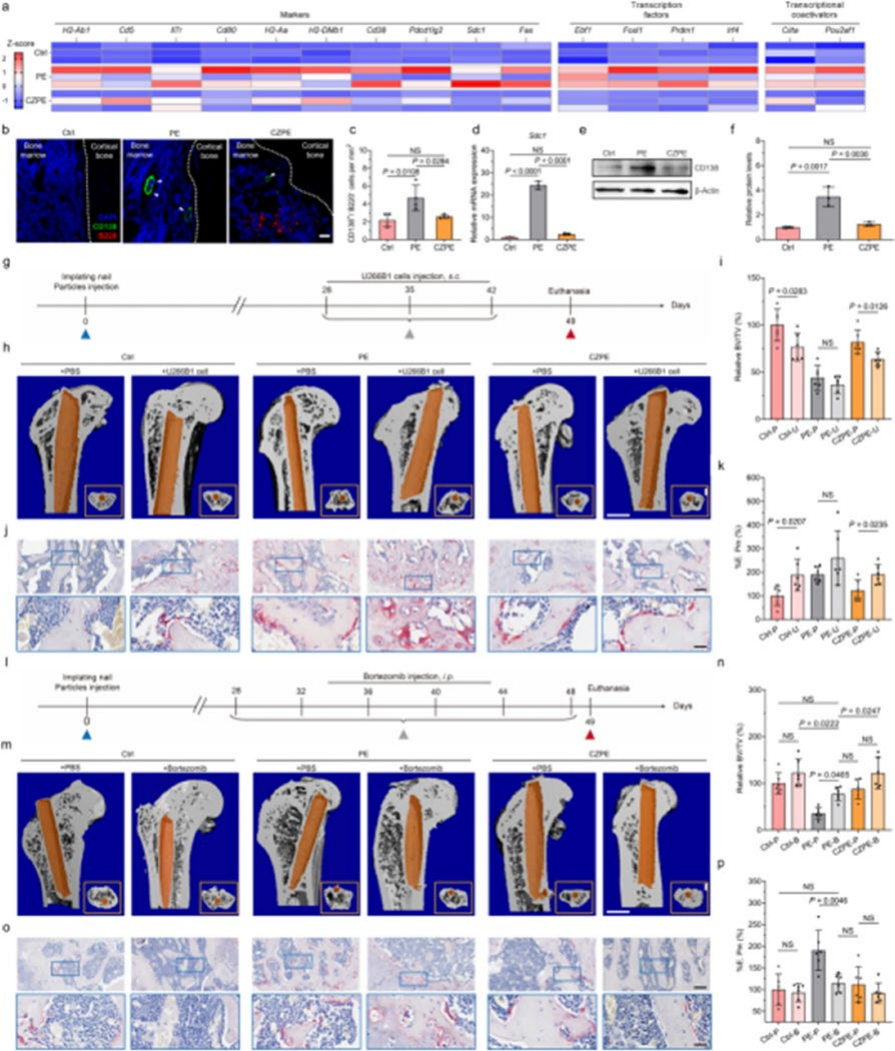
Preventive Effect of CZPE on WPO associated with plasma cell-mediated pathway. **a** Heat map of differentially expressed mRNAs (q < 0.05, |log_2_^foldchange^ > 1|) encoding proteins associated with marker proteins, transcription factors, and transcriptional coactivators involved in plasma cell development. **b** Representative immunofluorescence staining of femoral sections at day 49 post-surgery. Green indicates CD138; red indicates B220; blue indicates DAPI nuclear stain. Scale bar, 30 µm. **c** Quantification of CD138^+^/B220^–^ cell density corresponding to data in (**b**). **d** Relative expression of *Sdc1* mRNA in femur tissue samples. **e** Western blot analysis of expression levels of CD138 in femur tissue samples. β-Actin was used as a loading control. **f** Relative protein levels determined by density analysis in e.g., Schedule of the distal femoral implant models injected with U266B1 cells. **h** Representative micro-CT images of the femoral transverse section from U266B1 cells injected mice. Main images: femoral cross-section parallel to the direction of nail insertion. Insets: femoral cross-section perpendicular to the direction of nail insertion. Scale bar, 1 mm. **i** Quantifications of relative BV/TV corresponding to data in e (*n* = 6). **j** Representative histological images of TRAP-stained bone sections from U266B1 cells injected into mice. Top: 16 × magnification, scale bar, 100 µm; Bottom: 64 × magnification, scale bar, 25 µm. **k** Quantifications of %Er. Pm corresponding to data in j (*n* = 6). **l** Schedule for bortezomib administration in distal femoral implant models. **m** Representative micro-CT images of the femoral transverse section from bortezomib-treated mice. Main images: femoral cross-section parallel to the direction of nail insertion. Insets: femoral cross-section perpendicular to the direction of nail insertion. Scale bar, 1 mm. **n** Quantifications of relative BV/TV corresponding to data in m (*n* = 6). **o** Representative histological images of TRAP-stained bone sections from bortezomib-treated mice. Top: 16 × magnification, scale bar, 100 µm; Bottom: 64 × magnification, scale bar, 25 µm. **p** Quantifications of %Er. Pm corresponding to data in l (*n* = 6). In **i**, **k**, **n**, **p** “P” stands for PBS, “B” for Bortezomib, and “U” for U266B1 cells. Data in **c**, **d**, **f**, **i**, **k**, **n**, **p** are presented as mean ± s.d. *P* values for **c**, **d** and **f** were analyzed by one-way ANOVA with Tukey’s multiple comparisons test. *P* values for **i**, **k** were analyzed by an unpaired t-test. *P* values for **n**, **p** were analyzed by two-way ANOVA with Tukey’s multiple comparisons test. NS, not significant, *P* ≥ 0.05

## O7 The customized 3D-printed tri-flange in acetabular revision

### Lequan Liu, Jinping Pan, Huikang Guo

#### Jincheng General Hospital, Jincheng, Shanxi, China

##### **Correspondence:** Lequan Liu (luckenlaw@163.com)

*Arthroplasty 2026*, **8(1):**O7

**Background**

With the aging population and increasing prevalence of total hip arthroplasty (THA), revision surgeries for aseptic loosening, periprosthetic fractures, and implant-related complications have surged globally. Among these, acetabular bone defects—particularly severe Paprosky type III defects—pose significant challenges due to extensive bone loss and complex anatomical alterations. Traditional methods, such as structural bone grafting, metal augments & cages, or revision cups & “cup on cup”, all have limitations—including suboptimal implant fit, failure to achieve optimal stability and osseointegration, prolonged operative time, and high complication rates. 3D printing technology has emerged as a transformative solution, enabling patient-specific implants tailored to precise anatomical and biomechanical requirements.

**Methods**

We have designed and operated for 34 patients over the last 8 years, using preoperative CT and X-ray data to replicate patient-specific acetabular defects, working together with the engineers to design and adjust the parameters of the prosthesis, which usually takes days. ensuring meticulous reconstruction of the hip center and optimal load distribution. Their porous titanium alloy structure mimics trabecular bone, promoting osseointegration and reducing stress shielding (Fig. 1).

**Results**

All patients are doing well and are accountable, even for those cases with design flaws and the malplacement of the prosthesis. Anatomical Precision: Custom implants achieve a “perfect fit” for irregular defects, restoring hip biomechanics and alignment. Studies report postoperative acetabular cup abduction angles within the Lewinnek safe zone (40°–49°) and anteversion angles (19°–26°), significantly reducing dislocation risks. Reduced Operative Time and Morbidity: Preoperative simulations shorten surgery duration (average 151–223 min) and blood loss (600–1,500 mL). Enhanced Osseointegration: Porous structures with pore sizes of 50–500 μm facilitate bone ingrowth, improving long-term stability. Cost-Effectiveness: While initial manufacturing costs are higher, they are still a lot cheaper than cage + revision cup + augments, reduced hospital stays, and fewer complications offset expenses in the long term (Fig. 2).

**Conclusions**

3D-printed implants allow preoperative surgical simulation, enhancing precision and minimizing intraoperative risks. While challenges remain in material science and accessibility, ongoing advancements promise to solidify their role as a gold standard for complex hip revisions. Future research should prioritize long-term clinical trials and cost–benefit analyses to optimize their global implementation.

**Fig. 1 (Abstract O7).**
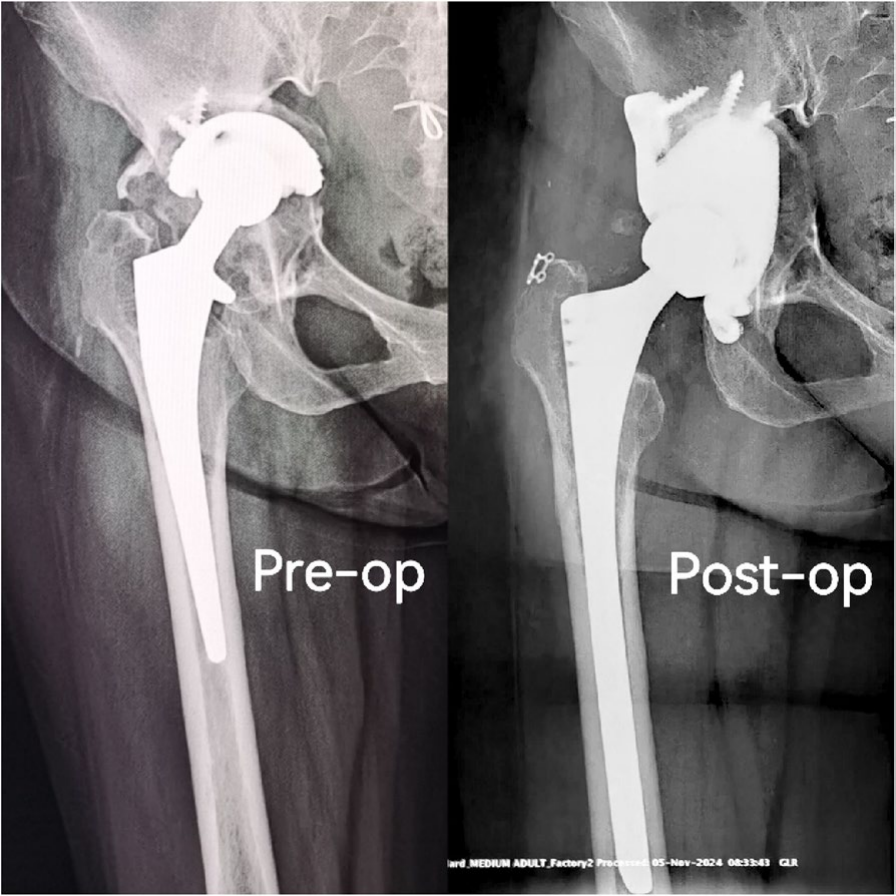
This is the preoperative and postoperative comparison image. The left image shows loosening of the acetabulum and stem, and the bone defect on the acetabular side. Post-op image shows that the hip center and offset are restored

**Fig. 2 (Abstract O7).**
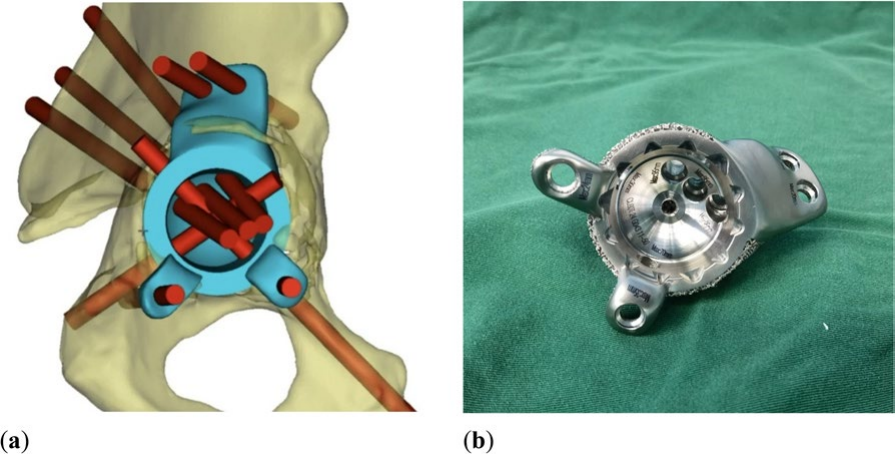
This figure illustrates the process of 3D design (**a**) and the final product (**b**), which incorporates the augment structure for bone defect, the tri-flange design to prevent the prosthesis from collapsing into the pelvis, and all possible screw holes with markers of required screw type (the matching screws are also designed, because they have different screw rods and thread lengths), and all surfaces in contact with bone are 3D-printed to simulated trabecular bone structures

## O8 Minimal clinically important difference of patient-reported outcomes after total knee arthroplasty varies according to pain type: a prospective study based on the PainDetect questionnaire

### Chao Xu^1^, Xiaofeng Chang^2^, Shuxin Yao^1^, Jianbing Ma^1^

#### ^1^Department of Knee Joint Surgery, Honghui Hospital, Xi’an Jiaotong University, Xi’an, China; ^2^Xi’an Medical University, Xi’an, China

##### **Correspondence:** Jianbing Ma (383697196@qq.com)

*Arthroplasty 2026*, **8(1):**O8

**Background**

After total knee arthroplasty (TKA), some patients with knee osteoarthritis experience persistent pain and functional impairment. Pain type—neuropathic pain (NeP) versus nociceptive pain (NP)—may differentially affect postoperative recovery. The PainDETECT questionnaire, widely used in pain assessment, effectively distinguishes between NeP and NP. However, its impact on the minimal clinically important difference (MCID) of the Western Ontario and McMaster Universities Osteoarthritis Index (WOMAC) scores remains unclear.

**Methods**

In this prospective cohort study, 625 patients with knee osteoarthritis were stratified based on PDQ scores. All patients were evaluated using the WOMAC index preoperatively and at 1 year postoperatively. MCIDs for the NeP and NP groups were determined using anchor-based and distribution-based methods. The change difference method defined the MCID as the difference in score changes between the minimal improvement group and the no-change group. Additionally, receiver operating characteristic (ROC) curve analysis was performed to calculate the MCID (Figs. 1 and 2), and the percentage of patients achieving the MCID was compared between groups. Sample size and statistical power were analyzed with G*Power software.

**Results**

At 1-year follow-up, the mean improvements in WOMAC total scores were 33.4 ± 24.1 for the NeP group and 41.0 ± 23.3 for the NP group (*P* < 0.001). Based on the change difference method, the WOMAC total score MCIDs were 23.0 for the NeP group and 16.3 for the NP group. ROC-derived MCIDs for the WOMAC total score were 23.2 and 17.5, respectively. Furthermore, the MCID thresholds for the pain and function subscales were higher in the NeP group, which also demonstrated significantly lower MCID achievement rates compared to the NP group (both *P* < 0.05).

**Conclusions**

This study provides evidence-based MCID values for Chinese TKA patients according to pain type. The findings indicate that patients with NeP require higher MCID thresholds on WOMAC scores than those with NP, and the MCID achievement rate is lower in the NeP group.

**Fig. 1 (Abstract O8).**
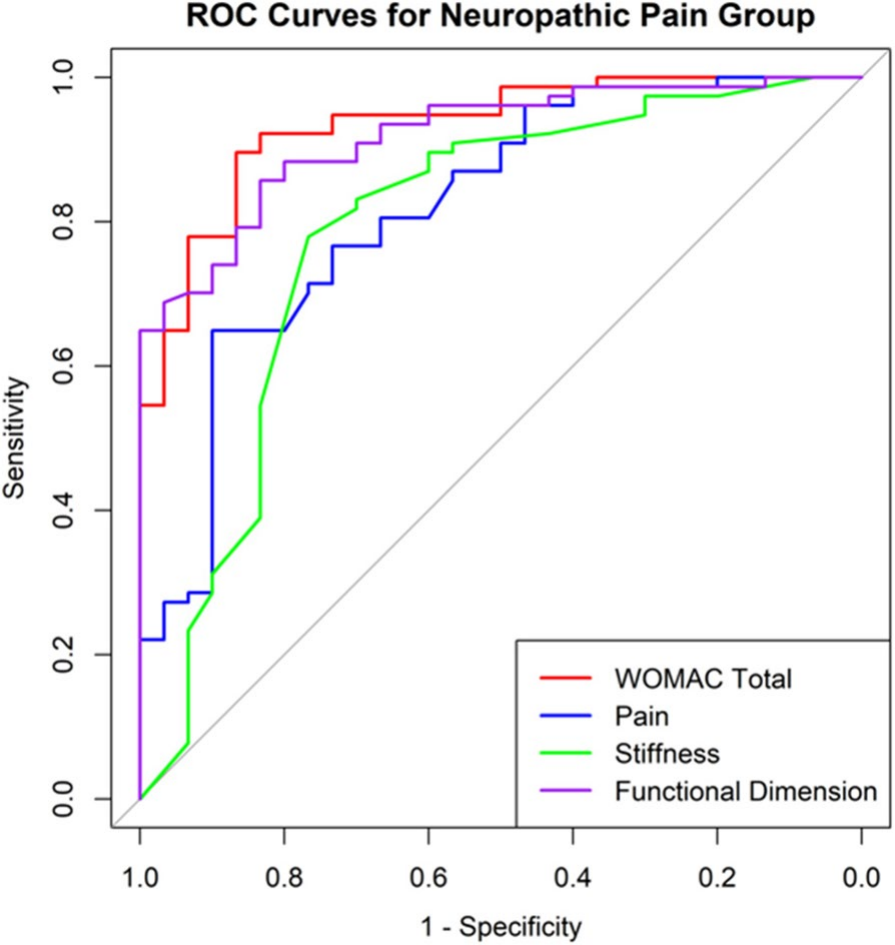
Receiver Operating Characteristic (ROC) curves for neuropathic pain group. The ROC curves illustrate the diagnostic performance of four predictive variables in identifying neuropathic pain. The red curve represents the WOMAC Total score, the blue curve represents the Pain dimension, the green curve represents the Stiffness dimension, and the purple curve represents the Functional dimension. The area under the curve (AUC) indicates the model’s diagnostic ability: an AUC of 0.5 suggests no discrimination, 0.7–0.8 is considered acceptable, 0.8–0.9 is considered excellent, and above 0.9 is outstanding

**Fig. 2 (Abstract O8).**
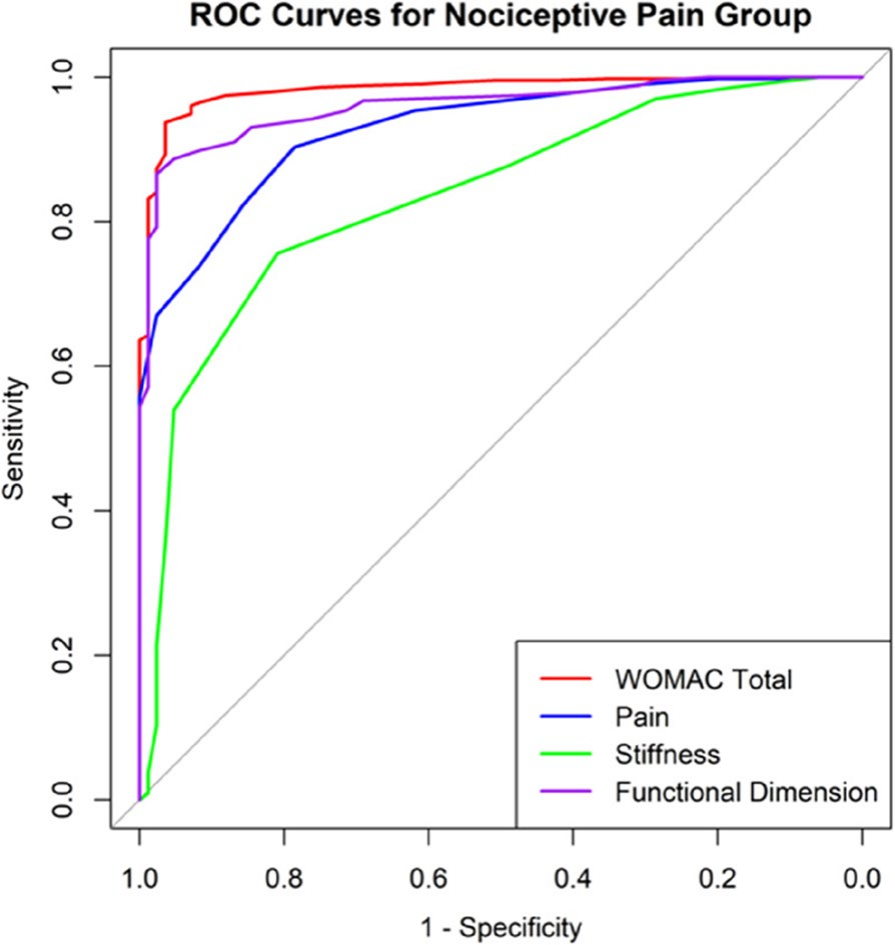
Receiver Operating Characteristic (ROC) curves for nociceptive pain group. The color scheme follows the same pattern as in Fig. 1. The AUC values provide insight into the model’s predictive performance, with higher AUC values reflecting better discrimination

## O9 Comparison of the application of lateral position DAA approach and posterolateral approach in hemiarthroplasty of elderly patients with femoral head fracture

### Ruixiang Ma, Xianli Hu, Chen Zhu

#### The First Affiliated Hospital of the University of Science and Technology of China, Hefei, China

##### **Correspondence:** Chen Zhu (zhuchena@ustc.edu.cn)

*Arthroplasty 2026*, **8(1):**O9

**Background**

Femoral neck fractures are a major public health concern, particularly among the elderly population, due to the high morbidity, mortality, and socioeconomic burden associated with these injuries. With the rapid aging of the global population—especially in developed regions such as Beijing, where individuals aged 65 and above account for a significant proportion of residents—the incidence of osteoporotic hip fractures continues to rise. Hemiarthroplasty (HA) has become a widely accepted surgical treatment for displaced femoral neck fractures in elderly patients, offering pain relief, early mobilization, and restoration of function. The choice of surgical approach in HA remains a topic of debate. The Direct Anterior Approach (DAA) in the lateral position and the posterolateral approach (PLA) are two commonly used techniques, each with distinct advantages and limitations. This study aims to analyze and compare the clinical outcomes, complication rates, and functional recovery between the lateral DAA and PLA in elderly patients undergoing HA for femoral neck fractures.

**Methods**

80 elderly patients with femoral neck fracture in the department of orthopedics of our hospital from January 2019 to December 2021 were selected. all patients were treated with artificial femoral head replacement and were divided into group A (DAA group, 45 cases) and group B (Posterolateral group, 40cases) according to different approaches. DAA approach was used in group A and posterolateral approach in group B. the operative indexes (operation time, intraoperative blood loss, drainage, hospital stay), pain degree (VAS score), hip joint function (Harris score), clinical efficacy and complications (poor incision healing, lower limb deep venous thrombosis and postoperative dislocation) were compared between the two groups.

**Results**

The drainage volume, intraoperative blood loss and hospital stay in group A were shorter than those in group B. the VAS score of group A was lower than that of group B at 1, 3 and 7 days after operation; the Harris score of group A was higher than that of group B at 1, 3 and 6 months after operation; the incidence of complications in group A was lower than that in group B, *P* = 0.34.

**Conclusions**

The application effect of the lateral position DAA approach in artificial femoral head replacement of elderly patients with femoral neck fracture is better than that of the posterolateral approach in the lateral recumbent position, which can reduce intraoperative bleeding, postoperative drainage, and shorten hospital stay. The DAA approach in the lateral recumbent position can relieve postoperative pain and help the hip joint function to recover as soon as possible.

## O10 Combined femoral and tibial osteotomy for the treatment of unicompartmental knee osteoarthritis

### Jianbin Guo, Yong Ding

#### Department of Joint Surgery, Hong Hui Hospital, Xi’an Jiaotong University, Xi’an, China

##### **Correspondence:** Jianbin Guo (drguojianbin@163.com)

*Arthroplasty 2026*, **8(1):**O10

**Background**

To evaluate the short-term clinical outcomes of combined high tibial osteotomy (HTO) and distal femoral osteotomy (DFO) in the treatment of medial unicompartmental knee osteoarthritis.

**Methods**

A retrospective study was conducted on 12 patients (7 males, 5 females; age range: 43–65 years, mean age: 53 ± 6 years) who underwent combined femoral and tibial osteotomy for knee osteoarthritis at the Department of Joint Surgery, Xi’an Honghui Hospital, between January 2022 and December 2023. All patients initially underwent arthroscopic examination and synovectomy, followed by meniscal repair or microfracture as indicated. Subsequently, double-level osteotomy (tibial and femoral) was performed to correct joint deformity. Medial open-wedge osteotomy was used for the tibia, while the femur was treated with either medial open-wedge or lateral closed-wedge osteotomy based on individual conditions. Intraoperative and postoperative complications were recorded. Preoperative and postoperative assessments included the Visual Analogue Scale (VAS), Knee Society Score (KSS), femoral-tibial angle (FTA), medial proximal tibial angle (MPTA), and medial joint space width. Follow-up duration ranged from 8 to 26 months (mean: 18 ± 5 months).

**Results**

Postoperative complications included hinge fractures in 4 patients, overcorrection in 1 patient, and delayed wound healing in 1 patient. No cases of infection or implant failure were observed. At the 6-month follow-up, all patients exhibited significant symptomatic relief. Marked improvements were noted in VAS, KSS, FTA, lateral distal femoral angle (LDFA), and MPTA, along with widening of the medial joint space. The differences were statistically significant (*P* < 0.05).

**Conclusions**

For patients with medial unicompartmental knee osteoarthritis accompanied by combined femoral and tibial deformities, double-level osteotomy effectively restores knee function with minimal tissue trauma, low complication rates, high safety, and reliable short-term outcomes, making it a clinically viable and recommended approach.

## O11 To save or sacrifice? MRI assessment of contralateral cartilage in periprosthetic joint infection after UKA

### Xinyu Fang, Wenming Zhang

#### Department of Orthopaedic Surgery, The First Affiliated Hospital of Fujian Medical University, Fuzhou, China

##### **Correspondence:** Xinyu Fang (fangxinyu0417@fjmu.edu.cn)

*Arthroplasty 2026*, **8(1):**O11

**Background**

To investigate the value of metal artifact-reduced sequence MRI (MARS MRI) in determining the extent of infection in periprosthetic joint infections (PJI) after unicompartmental knee arthroplasty (UKA) and to assess its usefulness as a preoperative guide to preserving or not preserving the contralateral compartment intraoperatively.

**Methods**

Prospectively and consecutively enrolled 17 patients with PJI following UKA treated at our hospital between January 2016 and January 2025, all of whom underwent preoperative MARS MRI. Orthopedic surgeons and radiologists independently assessed bone marrow edema (high signal on fat-suppressed T2-weighted sequences) and cartilage barrier defects (cartilage discontinuity or T2 high signal). Using intraoperative exploration as the gold standard, the sensitivity and specificity of MARS MRI in diagnosing contralateral compartment infection were analyzed, and inter-observer agreement was evaluated using the κ test.

**Results**

The sensitivity of bone marrow edema in diagnosing contralateral compartment infection was 75% (95% CI: 30.0–95.4), with a specificity of 87.5% (95% CI: 64.0–96.5). Cartilage barrier defects demonstrated 100% sensitivity (95% CI: 51.0–100.0) and 93.8% specificity (95% CI: 71.7–98.9). Overall, MARS MRI achieved 100% sensitivity and 87.5% specificity in detecting contralateral compartment involvement. Inter-observer agreement was excellent (κ = 0.73). Among patients who underwent contralateral compartment preservation (*n* = 16), postoperative infection control outcomes were favorable.

**Conclusions**

In MARS MRI, bone marrow edema and cartilage barrier defects are key imaging features for determining PJI involvement of the contralateral compartment following UKA, with cartilage integrity demonstrating particularly high sensitivity in assessing the extent of infection. MARS MRI can effectively guide intraoperative decision-making regarding preservation of the contralateral compartment, helping to balance infection eradication and joint function preservation. This provides crucial imaging evidence for the precise management of UKA-associated PJI.

## O12 Efficacy of robot-assisted core decompression and bone grafting for the treatment of arco II stage ONFH: a retrospective case–control study

### Chao Lu, Yangquan Hao, Lei Feng

#### Xi’an Honghui Hospital, Xi’an, China

##### **Correspondence:** Chao Lu (luchao0925@163.com)

*Arthroplasty 2026*, **8(1):**O12

**Background**

This study aimed to analyze the effectiveness of robot-navigation-assisted core decompression and bone grafting (RCDBG) for the treatment of ARCO II stage osteonecrosis of the femoral head (ONFH).

**Methods**

A retrospective analysis was conducted on 100 patients who underwent core decompression and bone grafting for femoral head necrosis at Xi’an Honghui Hospital from July 2017 to December 2019. The study included 50 patients who underwent RCDBG and 50 who underwent conventional core decompression and bone grafting (CCDBG). All operations were performed by the same surgeon. Outcome measures included preoperative and final follow-up Harris Hip Scores (HHS), postoperative Visual Analogue Scale (VAS), femoral head collapse, intraoperative fluoroscopic times, intraoperative blood loss, and operation time.

**Results**

All patients were followed up for 20–38 months (mean 28.53 ± 0.50 months). The RCDBG group had significantly fewer intraoperative fluoroscopic times and shorter operation times than the CCDBG group (*P* = 0.001). There were no significant differences in final HHS, postoperative VAS, femoral head collapse, or intraoperative blood loss between the two groups (*P* > 0.05).

**Conclusions**

RCDBG for ARCO II ONFH significantly improves the accuracy of core decompression and bone grafting, reduces intraoperative fluoroscopic times, and shortens operation time without affecting other measured outcomes.

## O13 Early functional outcomes after fixed-bearing medial unicompartmental knee arthroplasty in patients with patellofemoral osteoarthritis: a comparative study

### Nonn Jaruthien, Kanaphat Rattananithikul, Aree Tanavalee, Srihatach Ngarmukos, Chavarin Amarase, Chotetawan Tanavalee, Pakpoom Somrak

#### Department of Orthopaedics, Faculty of Medicine, Chulalongkorn University, Bangkok, Thailand

##### **Correspondence:** Srihatach Ngarmukos (srihatach@hotmail.com)

*Arthroplasty 2026*, **8(1):**O13

**Background**

Over its 50-year history, medial unicompartmental knee arthroplasty (UKA) has been used to treat osteoarthritis (OA) of the knee. This procedure replaces only the medial compartment of the knee. The presence of patellofemoral joint osteoarthritis (PFOA) has been questioned as a contraindication due to the limited results of fixed-bearing medial UKA in PFOA cases. The objective of this study is to compare the functional outcomes after fixed-bearing medial UKA between patients with PFOA and those without.

**Methods**

This prospective cohort study included a total of 51 fixed-bearing medial UKAs (SIGMA®HP Partial Knee, DePuy Synthes, Warsaw, USA). Intra-operative assessments (Fig. 1) categorized patients into a PFOA group (The International Cartilage Repair Society, ICRS grades I-IV) and a non-PFOA group (ICRS grade 0). Clinical information was double-blinded to the surgeon, and intra-operative information was blinded to the follow-up physician (Fig. 2). Functional outcomes were measured through both performance-based tests and Patient-reported outcome measures (PROMs). The performance-based tests included the five-times sit-to-stand test (5-STS), timed up-and-go test (TUG), and 3-min walk test (3mWT). Patient-reported outcome measures included the Oxford Knee Score (OKS), Knee Society Score (KSS), the 12-item Short Form Health Survey (SF-12), and range of motion (ROM). Statistical analyses included independent t-tests and Chi-square tests for demographic data. Paired t-tests and generalized estimating equations (GEE) were used for all pre-operative and post-operative outcome comparisons, with a significance threshold set at *P*-value < 0.05.

**Results**

We recruited 51 patients (51 knees) for this study, divided into two groups based on intraoperative patellofemoral findings: 34 knees in the PFOA group and 17 in the non-PFOA group. Demographic data showed that the PFOA group had a significantly longer duration of pain onset compared to the non-PFOA group (2.75 ± 1.5 years vs. 2.03 ± 0.84 years, *P* = 0.033). No other parameters showed a statistically significant difference. At the 1-year postoperative follow-up, there were no significant differences in functional performance or patient-based clinical scores between PFOA and non-PFOA groups across measures, including 5-STS, TUG, 3mWT, KSS pain, KSS function, OKS, SF-12, and ROM. By 6 weeks, both groups showed significant improvement over preoperative scores in 5-STS, KSS pain, OKS, and SF-12. At 12 weeks, further improvements were seen in KSS function, ROM, TUG, and 3mWT for both groups compared to preoperative scores (Table 1).

**Conclusions**

Fixed-bearing medial UKA yields similar functional outcomes in patients with and without PFOA at 1-year postoperative follow-up. Both groups showed significant early improvements in functional performance and patient-reported outcomes, including 5-STS, KSS pain, OKS, and SF-12. These findings suggest that fixed-bearing medial UKA can be an effective treatment for patients with medial compartment osteoarthritis, regardless of the presence of PFOA. Further research with larger sample sizes and longer follow-up periods is needed to confirm the long-term effectiveness of this approach in patients with PFOA.

**Fig. 1 (Abstract O13).**
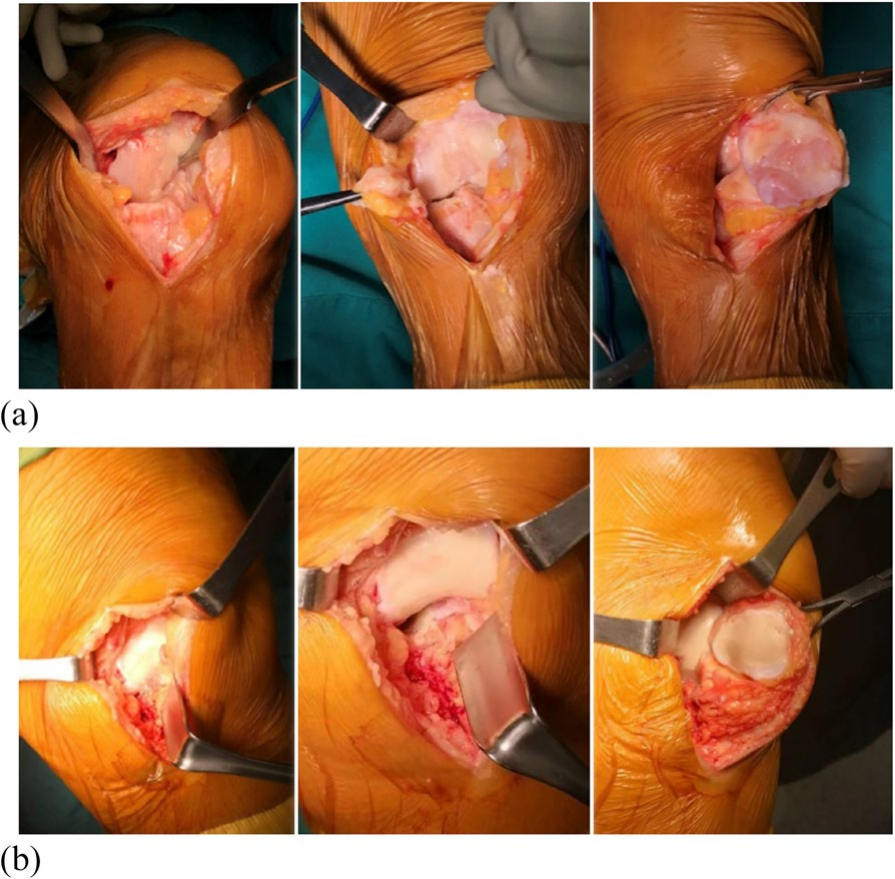
Intra-operative patellofemoral joint; **a** Non-patellofemoral osteoarthritis (Non-PFOA) with normal cartilage at the trochlear and both patellar facets (ICRS grade 0). **b** Patellofemoral osteoarthritis (PFOA) with cartilage injury grade IV according to ICRS (International Cartilage Repair Society)

**Fig. 2 (Abstract O13).**
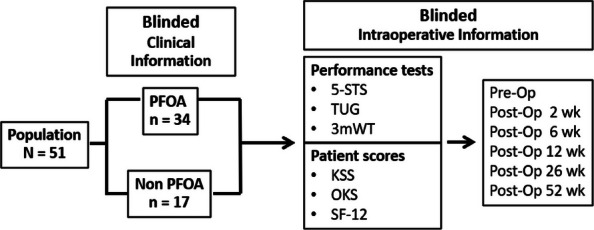
Framework. 5-STS = Five-times sit to stand, TUG = Time-up and go test, 3mWT = 3 min walk test. KSS = Knee society score, OKS = Oxford knee score, SF-12 = Short form-12

**Table 1 (Abstract O13).** Results

	**Non-PFOA (*n* = 17)**	**PFOA (*n* = 34)**	***P*-value**
Range of motion (degrees)			
Pre-operative	117.35 ± 11.87	118.29 ± 11.98	0.792
2 weeks	115.53 ± 11.8	113.59 ± 14.45	0.634
6 weeks	120.29 ± 8.56	120.76 ± 10.78	0.876
12 weeks	125.41 ± 6.15	125.18 ± 7.69	0.913
26 weeks	126.47 ± 6.06	125.12 ± 7.48	0.521
52 weeks	126.47 ± 6.06	125.41 ± 6.84	0.591
5-STS (sec)			
Pre-operative	22.67 ± 6.09	21.13 ± 5.49	0.366
2 weeks	27.21 ± 6.64	25.98 ± 7.1	0.554
6 weeks	20.96 ± 4.41	18.82 ± 4.79	0.129
12 weeks	16.12 ± 3.94	15.23 ± 4.3	0.473
26 weeks	15.34 ± 4.04	15.16 ± 4.2	0.885
52 weeks	14.43 ± 4.65	15.03 ± 3.99	0.633
TUG (sec)			
Pre-operative	15.66 ± 3.72	15.52 ± 5.16	0.918
2 weeks	24.2 ± 7.37	23.76 ± 12.07	0.873
6 weeks	16.33 ± 6.1	15.94 ± 7.78	0.857
12 weeks	12.43 ± 3.72	12.2 ± 3.91	0.842
26 weeks	12.62 ± 3.82	12.13 ± 3.64	0.659
52 weeks	12.54 ± 3.92	12.11 ± 3.36	0.700
3 m-WT (meters)			
Pre-operative	93.21 ± 19.82	95.69 ± 20.02	0.677
2 weeks	67.05 ± 19.33	80.18 ± 30.04	0.066
6 weeks	101 ± 21.65	107.88 ± 28.08	0.380
12 weeks	126.71 ± 23.13	128.87 ± 35.04	0.819
26 weeks	127.41 ± 21.1	129.24 ± 31.6	0.831
52 weeks	127.88 ± 24.48	130.59 ± 28.73	0.741
KSS pain (max 50)			
Pre-operative	31.47 ± 12.34	28.38 ± 11.4	0.379
2 weeks	31.18 ± 10.54	31.76 ± 9.53	0.842
6 weeks	41.47 ± 7.66	40.44 ± 6.44	0.616
12 weeks	47.06 ± 4.7	44.71 ± 6.74	0.203
26 weeks	47.35 ± 4.37	45.29 ± 4.25	0.113
52 weeks	46.76 ± 5.57	45.15 ± 4.52	0.271
KSS function (max 100)			
Pre-operative	56.76 ± 13.8	54.26 ± 18.14	0.620
2 weeks	37.65 ± 8.5	39.56 ± 12.99	0.585
6 weeks	61.47 ± 11.83	62.21 ± 13.66	0.851
12 weeks	80.88 ± 13.02	80 ± 14.87	0.836
26 weeks	82.06 ± 11.87	80.59 ± 13.58	0.706
52 weeks	83.24 ± 9.83	80.74 ± 12.98	0.488
Oxford knee score (OKS)			
Pre-operative	28 ± 6.53	26.68 ± 5.94	0.472
2 weeks	26.65 ± 5.44	25.79 ± 6.15	0.630
6 weeks	32.82 ± 4.54	33.09 ± 5.93	0.872
12 weeks	38.24 ± 4.75	38.65 ± 6.66	0.821
26 weeks	38.41 ± 4.68	38.38 ± 6.62	0.987
52 weeks	38.47 ± 5.23	38.24 ± 6.42	0.896
SF-12			
Pre-operative	30.71 ± 3.8	31.74 ± 4.58	0.429
2 weeks	28.12 ± 2.85	29.24 ± 3.64	0.274
6 weeks	33.12 ± 2.83	34.44 ± 3.78	0.208
12 weeks	38.29 ± 3.72	38.79 ± 4.46	0.693
26 weeks	38.29 ± 3.85	38.79 ± 4	0.672
52 weeks	38.82 ± 4.07	39.06 ± 3.95	0.843

Value presented as mean ± SD., and Mean change ± SD. *P*-value corresponds to the Paired t test and Generalized Estimating Equations (GEE)

## O14 Dental plaque disclosing gel: an effective detecting agent for guiding debridement of bacterial biofilms in periprosthetic joint infection

### Wushi Cui^1^, Jiaxuan Zou^1^, Congsun Li^1^, Songyu Hu^2^, Haobo Wu^1^, An Liu^1^

#### ^1^Department of Orthopedics, The Second Affiliated Hospital, Zhejiang University School of Medicine, Hangzhou, China; ^2^The State Key Laboratory of Fluid Power and Mechatronic Systems, College of Mechanical Engineering, Zhejiang University, Hangzhou, China

##### **Correspondence:** An Liu (la@zju.edu.cn)

*Arthroplasty 2026*, **8(1):**O14

The full article of this study has been published online. Please refer to the full text at: 10.1016/j.jinf.2025.106581.

## O15 Long-term outcomes of cementless total hip arthroplasty using a 32 mm femoral head with CT-based navigation: a comparative study with manual THA

### Shine Tone, Gai Kobayashi, Yohei Naito, Masahiro Hasegawa

#### Department of Orthopaedic Surgery, Mie University, Graduate School of Medicine, Tsu, Japan

##### **Correspondence:** Shine Tone (s-tone@med.mie-u.ac.jp)

*Arthroplasty 2026*, **8(1):**O15

**Background**

This study aims to compare long-term clinical and radiographic outcomes between cementless THA performed with CT-based navigation and manual techniques, using a 32 mm femoral head.

**Methods**

A retrospective cohort study was conducted on 93 hips in 83 patients who underwent primary cementless THA with a 32 mm femoral head between April 2011 and September 2014. All patients had a minimum of 10 years of postoperative follow-up. The CT-based navigation group (CT-based group) comprised 48 hips, and the manual THA group (Manual group) comprised 53 hips. Postoperative radiographic inclination (RI) and radiographic anteversion (RA) were evaluated using three-dimensional analysis software based on CT scans obtained two weeks after surgery. The percentage of cups placed within the Lewinnek safe zone was calculated. In the CT-based group, absolute errors between intraoperative navigation-recorded cup angles and postoperative cup angles were calculated. Clinical evaluations included the Merle d’Aubigné and Postel score, Harris Hip Score (HHS), assessed preoperatively and at final follow-up. Postoperative complications and 10-year implant survival rates (revision THA as endpoint) were also evaluated. Statistical significance was set at *P* < 0.05.

**Results**

The mean postoperative RI and RA were 40.0° ± 3.4° and 14.9° ± 3.4° in the CT-based group, and 37.0° ± 9.4° and 22.9° ± 9.1° in the Manual group, respectively. The proportion of cups placed within the Lewinnek safe zone was significantly higher in the CT-based group (100%) compared to the Manual group (49%). In the CT-based group, the mean absolute errors in cup positioning were 1.5° ± 1.7° for RI and 2.1° ± 1.8° for RA. Both groups showed significant improvement in the Merle d’Aubigné and Postel scores and HHS postoperatively. At final follow-up, the CT-based group had significantly higher Merle d’Aubigné and Postel scores and HHS than the Manual group (Figs. 1 and 2). No dislocations or cup loosening occurred in either group. One case of infection occurred in the CT-based group, requiring revision THA. The 10-year implant survival rates were 98.2% in the CT-based group and 100% in the Manual group, with no significant difference (Fig. 3).

**Conclusions**

CT-based navigated THA with a 32-mm femoral head achieves more accurate cup positioning and better long-term clinical outcomes than manual THA. Although the survival rates were comparable, cementless THA performed with CT-based navigation demonstrated advantages in clinical precision and effectiveness over a 10-year follow-up period.

**Fig. 1 (Abstract O15).**
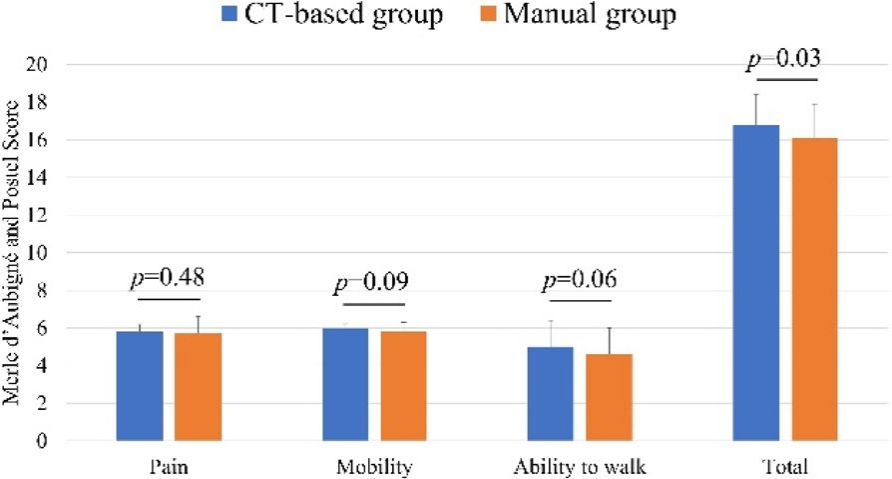
Comparison of the Merle d’Aubigné and Postel score (pain, mobility, ability to walk, and total) between the CT-based and Manual groups

**Fig. 2 (Abstract O15).**
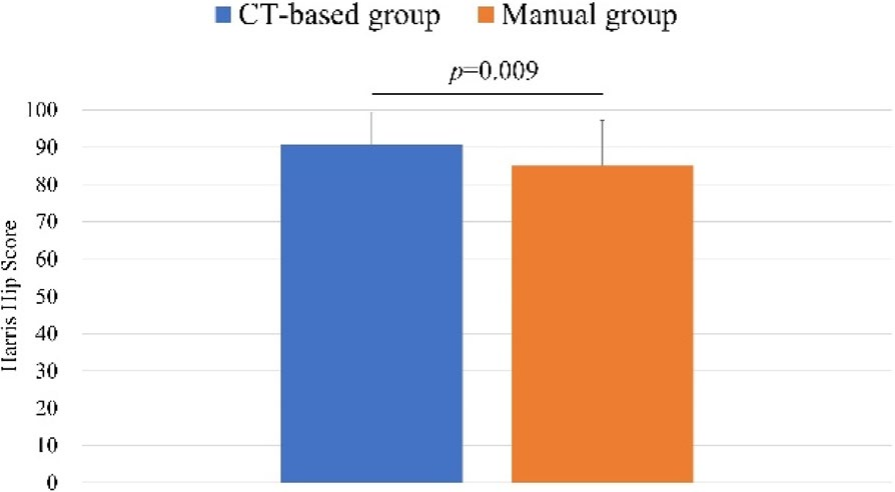
Comparison of the Harris Hip Score between the CT-based and Manual groups

**Fig. 3 (Abstract O15).**
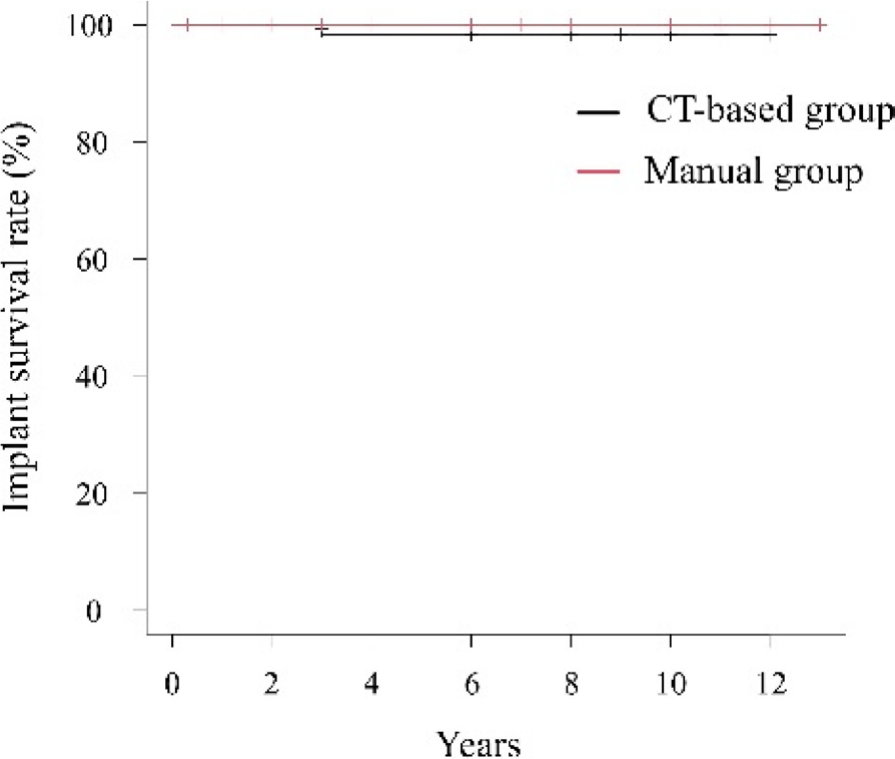
10-year implant survival rates (revision THA as endpoint)

## O16 Robotic-assisted functional alignment with adjusted mechanical alignment in total knee arthroplasty: shifts in CPAK phenotypes and clinical implications

### Chuanlong Wu^1^, Zhijie Chen^1^, Hanqi Wang^2^, Chuan He^1^

#### ^1^Department of Orthopaedics, Shanghai Key Laboratory for Prevention and Treatment of Bone and Joint Diseases, Shanghai Institute of Traumatology and Orthopaedics, Ruijin Hospital, Shanghai Jiao Tong University School of Medicine, Shanghai, China; ^2^Department of Radiology, Ruijin Hospital, Shanghai Jiao Tong University School of Medicine, Shanghai, China

##### **Correspondence:** Chuan He (drhechuan@sina.com)

*Arthroplasty 2026*, **8(1):**O16

**Background**

Total knee arthroplasty (TKA) remains the gold standard for end-stage knee osteoarthritis and complex deformities, where optimal alignment and soft-tissue balance are critical for long-term success. With the growing adoption of robotic-assisted surgery, functional alignment (FA)—prioritizing soft-tissue equilibrium—has gained traction. However, debates persist regarding unrestricted FA and the implementation/outcomes of restricted FA. This study investigates the influence of FA integrated with adjusted mechanical alignment (aMA) on postoperative CPAK classification changes and its clinical implications.

**Methods**

From May 2023 to October 2024, a prospective cohort of robotic-assisted TKA patients was retrospectively evaluated. FA + aMA (≤ 3° neutral alignment) was performed using a robotic system, with 101 well-balanced cases (medial/lateral and flexion/extension gap differences < 2 mm) included. All procedures utilized posterior cruciate-stabilizing implants. Pre- and postoperative full-leg radiographs were analyzed by three independent observers to measure: lateral distal femoral angle (LDFA), medial proximal tibial angle (MPTA), arithmetic hip-knee-ankle angle (aHKA = MPTA – LDFA), and joint line obliquity (JLO = MPTA + LDFA). CPAK classification was determined pre- and postoperatively, and comparative analysis was conducted.

**Results**

The mean patient age was 70.4 years, with 17 males and 86 females. Mean follow-up was 6.5 months. Knee Society Score improved from 57.25 ± 2.20 preoperatively to 89.82 ± 3.1 at final follow-up (*P* < 0.001).

1. CPAK I (*n* = 48, 48%): Pre- vs. postoperative: LDFA (88.60° vs. 91.30°, *P* < 0.05), MPTA (81.02° vs. 88.74°, *P* < 0.05), aHKA (− 7.57° vs. − 2.56°, *P* < 0.05), JLO (169.62° vs. 180.04°, *P* < 0.05). Preoperative varus alignment with the distal joint line apex shifted postoperatively to residual mild varus and neutral joint line (CPAK IV).
2. CPAK II (*n* = 19, 19%): LDFA (85.67° vs. 90.80°, *P* < 0.05), MPTA (85.56° vs. 89.44°, *P* < 0.05), aHKA (− 0.11° vs. − 1.36°), JLO (171.23° vs. 180.24°, *P* < 0.05). Preoperative neutral alignment with distal joint line apex transitioned postoperatively to neutral alignment and horizontal joint line (CPAK V).
3. CPAK III (*n* = 22, 22%): LDFA (82.57° vs. 89.76°, *P* < 0.05), MPTA (88.16° vs. 89.73°), aHKA (5.60° vs. − 0.029°, *P* < 0.05), JLO (170.73° vs. 179.50°, *P* < 0.05). For valgus-aligned patients, we advocate complete correction of residual valgus deformity to achieve neutral mechanical alignment. Preoperative valgus alignment with the distal joint line apex achieved postoperatively near-neutral alignment and horizontal joint line (CPAK V).
4. CPAK IV (*n* = 8, 8%): MPTA (86.35° vs. 89.21°, *P* < 0.05), aHKA (− 7.64° vs. − 1.75°, *P* < 0.05). Preoperative varus alignment with horizontal joint line shifted postoperatively to restored (residual mild) varus with horizontal joint line (CPAK V).
5. CPAK V (*n* = 4, 4%): Postoperatively, CPAK V patients remained classified as type V.
6. Given the small cohort size, no other CPAK phenotypes were observed, aligning with reports in the literature (Fig. 1).

**Conclusions**

Robotic-assisted FA + aMA facilitates balanced gap optimization in TKA. While preserving soft-tissue equilibrium, varus and valgus deformities were corrected to within 3° of neutral alignment, with joint line restoration shifting postoperative CPAK classification predominantly to types IV and V. This approach demonstrates reproducible alignment precision and functional improvement, supporting its role in personalized TKA planning.

**Fig. 1 (Abstract O16).**
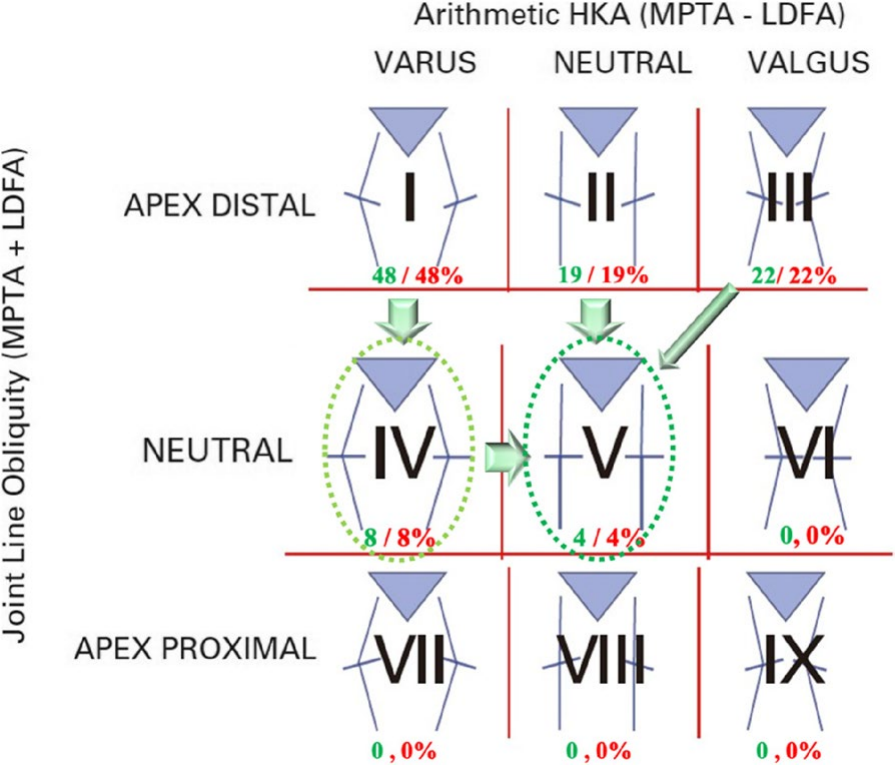
Preoperative CPAK phenotype distribution and postoperative transition patterns. The chart illustrates the preoperative CPAK classification profile (Type I: 48%, Type II: 19%, Type III: 22%, Type IV: 8%, Type V: 4%; no other phenotypes observed). Postoperatively, Type I cases predominantly transitioned to Type IV, while Types II, III, and IV converged toward Type V. Native Type V phenotypes remained unchanged

## O17 Influence of alignment and joint line obliquity on survival of total knee arthroplasty: long-term follow-up of 800 cases

### Hong Yeol Yang, Jong Keun Seon, Sung Ju Kang, Jong Eun Kim, Woo Jin Jeong

#### Department of Orthopaedic Surgery, Chonnam National University Hwasun Hospital, College of Medicine, Chonnam National University, 322 Seoyang-ro, Hwasun, Republic of Korea

##### **Correspondence:** Jong Keun Seon (seonbell@chonnam.ac.kr)

*Arthroplasty 2026*, **8(1):**O17

The full article of this study has been published online. Please refer to the full text at: 10.1016/j.arth.2025.10.114.

## O18 Incidence and characteristics of noise generation in total hip arthroplasty with ceramic-on-ceramic bearings

### Xianzuo Zhang^1^, Mo Chen^1^, Tao Zhang^1^, Haining Zhang^2^, Bo Yang^3^, Chen Zhu^1^

#### ^1^Department of Orthopedics, The First Affiliated Hospital of USTC, Division of Life Sciences and Medicine, University of Science and Technology of China, Hefei, China; ^2^Department of Joint Surgery, The Affiliated Hospital of Qingdao University, Qingdao, China; ^3^Department of Joint Surgery, Weifang People's Hospital, Weifang, China

##### **Correspondence:** Chen Zhu (zhuchena@ustc.edu.cn)

*Arthroplasty 2026*, **8(1):**O18

**Background**

Total hip arthroplasty (THA) with ceramic-on-ceramic (CoC) bearings is associated with noise generation, a complication affecting patient satisfaction. While CoC bearings offer superior wear resistance, noise prevalence ranges from 3 to 53% in long-term studies, attributed to factors like component malpositioning. Robotic-assisted surgery (RAS) enhances precision in implant placement, potentially reducing noise risk. This study compared noise incidence between RAS and conventional (CON) THA methods and evaluated predictors of noise.

**Methods**

A secondary analysis of a multicenter RCT (June 2021–July 2022) included 74 patients with CoC bearings (37 RAS, 37 CON). Noise incidence, characteristics, and duration were recorded at 14 days and 24 weeks postoperatively. Radiological assessments measured acetabular alignment, femoral component positioning, and leg length changes. Logistic regression identified predictors of noise. Statistical tests (chi-squared, paired t-test) compared outcomes, with significance at *P* < 0.05.

**Results**

Early noise incidence was significantly lower in RAS (5.4%) versus CON (32.4%) at 14 days (*P* = 0.008). At 24 weeks, noise rates were 5.4% (RAS) and 21.6% (CON) (*P* = 0.089). RAS demonstrated superior precision: COR deviation (RAS: 0.90 mm vs. CON: 11.51 mm, *P* < 0.001), femoral alignment (RAS: 1.74° vs. CON: 2.19°, *P* = 0.009), and leg length change (RAS: 2.80 mm vs. CON: 6.17 mm, *P* = 0.001, Fig. 1, Table 1). Logistic regression identified CON surgery as a noise predictor (OR = 7.10, 95% CI 1.51–33.33; *P* = 0.013). Acetabular abduction angle differed between noise (38.53°) and non-noise (42.72°) groups (*P* = 0.035). Functional outcomes (HHS, OHS) did not differ between groups.

**Conclusions**

RAS significantly reduces early postoperative noise in CoC THA, likely due to enhanced component alignment. CON surgery, posterior approach, and smaller femoral head size were key noise predictors. Despite similar functional outcomes, RAS mitigates noise-related complications, emphasizing its role in optimizing THA precision.

**Fig. 1 (Abstract O18).**
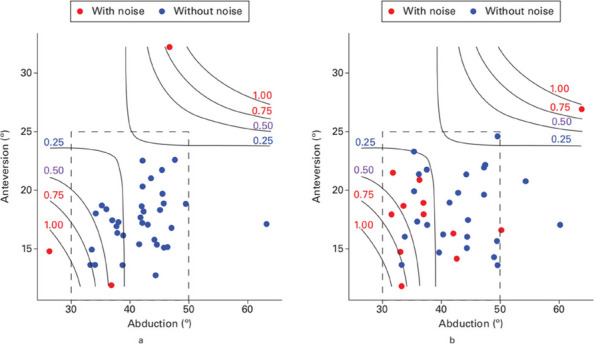
**a** Projection of the robotic-assisted surgery group data onto the noise probability map, showing the relationship between robotic surgery and noise incidence. **b** Projection of the conventional group data on the same map, offering a comparative view of manual techniques. Both **a** and **b** include grey curves with numerical labels (0.25, 0.5, 0.75, and 1) representing noise thresholds and their associated probabilities

**Table 1 (Abstract O18).** Radiographic assessments comparisons between patients with and without noise complaints

	**Without noise**	**With noise**	***P***
Abduction (°, mean (SD))	42.72 (6.18)	38.53 (9.07)	0.035
Abduction deviation (°, mean (SD))	5.27 (4.16)	7.18 (5.44)	0.135
Anteversion (°, mean (SD))	17.84 (2.81)	18.17 (5.34)	0.744
Anteversion deviation (°, mean (SD))	3.21 (2.39)	4.19 (4.53)	0.245
COR distance from planned (mm, mean (SD))	5.84 (9.86)	7.52 (7.37)	0.53
Acetabular depth (mm, mean (SD))	16.30 (2.83)	16.99 (3.35)	0.408
Femoral component anteversion (°, mean (SD))	13.82 (3.67)	14.21 (3.47)	0.699
Stem alignment (°, mean (SD))	1.93 (0.65)	2.10 (1.07)	0.43
Change in leg length (mm, mean (SD))	4.08 (4.75)	5.95 (3.25)	0.145

## O19 Mid-term outcomes of “shackle-type lower limb abduction casting” for recurrent dislocation after hip arthroplasty

### Chuanlong Wu, Zhijie Chen, Jiong Zhang, Chuan He

#### Department of Orthopaedics, Shanghai Key Laboratory for Prevention and Treatment of Bone and Joint Diseases, Shanghai Institute of Traumatology and Orthopaedics, Ruijin Hospital, Shanghai Jiao Tong University School of Medicine, Shanghai, China

##### **Correspondence:** Chuan He (drhechuan@sina.com)

*Arthroplasty 2026*, **8(1):**O19

**Background**

Dislocation remains a common complication following hip arthroplasty, with recurrent instability posing significant challenges post-reduction. Traditional immobilization methods—including skin traction, T-shoe traction, and pelvic stabilizers with bilateral/unilateral limb fixators—often compromise patient mobility and wound care accessibility. We developed an innovative “Shackle-Type Lower Limb Abduction Casting” (S-LAC) technique, providing rigid immobilization while preserving bed mobility exercises and perineal hygiene. This study evaluates its mid-term efficacy in preventing redislocation and function recovery, a method unreported in existing literature.

**Methods**

We retrospectively analyzed 27 cases (from 3,360 hip arthroplasty patients between January 2013 and December 2022) who underwent closed/open reduction combined with S-LAC, excluding cases with component loosening/malposition. Initial procedures included: bipolar hemiarthroplasty (BHA, *n* = 8), primary total hip arthroplasty (THA, *n* = 9), and revision hip arthroplasty (RHA, *n* = 10). The S-LAC technique immobilized both lower limbs in 15–20°abduction with appropriate internal/external rotation (based on dislocation mechanism) using below-knee casts connected by a crossbar, maintained for 6–8 weeks (Fig. 1). For recurrent dislocations without component malposition, we adhere to the S-LAC protocol (reapplication 6–8 weeks). Demographics, surgical history, reduction approach (closed:20; open:7), and 2-year minimum follow-up data were analyzed.

**Results**

The cohort (mean age 75.2 ± 11.15 years) comprised 17 primary dislocations (BHA:7, THA:5, RHA:5) and 10 recurrent dislocations (BHA:1, THA:4, RHA:5). Redislocation triggers included inadequate prior immobilization and neuropsychiatric noncompliance.

1. Primary dislocation group: 17 patients (closed reduction:12; open:5) achieved 100% stability at mean 6.4-year follow-up. Mean Harris Hip Score improved to 89.5.
2. Recurrent dislocation group: All 10 patients (closed:8; open:2) maintained stability post-S-LAC at mean 4.9-year follow-up. Mean Harris Hip Score improved to 89.3.
3. No cast-related complications (e.g., pressure ulcers, DVT) occurred.

**Conclusions**

The S-LAC technique demonstrates three key advantages: 1) Biomechanical efficacy: Abduction-rotation control prevents redislocation regardless of initial/recurrent status. 2) Enhanced practicality: Distal fixation preserves perineal access for wound care/skin integrity while enabling bedside sitting. 3) Safety profile: Zero cast-related complications versus traditional methods. These outcomes position S-LAC as a paradigm-shifting immobilization strategy, warranting multicenter validation and biomechanical studies.

**Fig. 1 (Abstract O19).**
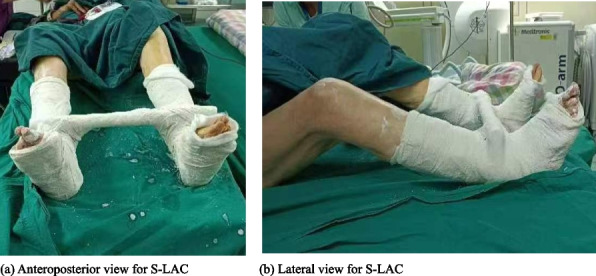
Schematic illustrations of the “Shackle-Type Lower Limb Abduction Casting” (S-LAC)

## O20 Application of extended trochanteric osteotomy combined with a longer biological femoral component in one-stage total hip arthroplasty revision for periprosthetic joint infection: a retrospective study

### Yakang Wang

#### Department of Joint Surgery, Honghui Hospital of Xi’an Jiaotong University School of Medicine, Xi’an, China

##### **Correspondence:** Yakang Wang (wangyakangde@163.com)

*Arthroplasty 2026*, **8(1):**O20

**Background**

This retrospective study aimed to investigate the efficacy of extended trochanteric osteotomy (ETO) combined with a longer biological femoral component in primary total hip arthroplasty (THA) revision for periprosthetic joint infection (PJI) and to analyze the influencing factors.

**Methods**

A total of 38 patients (mean age 56.2 years; 18 males, 20 females) who underwent ETO-assisted one-stage THA revision for PJI at two hospitals between January 2018 and January 2022 were analyzed. All patients were diagnosed based on the Musculoskeletal Infection Society criteria. Infection recurrence rate, femoral osteotomy healing, complications, and functional status (Harris Hip Score, HHS) were evaluated. T-tests were used for continuous variables, Chi-square tests for categorical variables, and logistic regression for risk factor analysis (Figs. 1 and 2).

**Results**

All patients were followed up until January 2024, with a mean follow-up of 30.8 months (range, 24–48 months). Infection was controlled in 36 patients (94.74%), with recurrence in 2 (5.26%). The average osteotomy healing time was 15.2 ± 1.6 weeks. The mean HHS significantly improved from 35.63 ± 5.15 preoperatively to 79.52 ± 5.24 at the final follow-up (*P* = 0.0017). Complications occurred in 6 cases (15.79%), including 2 infection recurrences, 2 Postoperative periprosthetic fractures, and 2 cases of mild painless limp due to limb length discrepancy > 2 cm.

**Conclusions**

ETO-assisted primary THA revision is an effective treatment for PJI, capable of effectively controlling infection and improving patients’ functional status. However, there is still a risk of complications. Influencing factors for infection control rate included patient age, sex, primary disease, comorbidities, infection type, infection duration, and bacterial species (Table 1).

**Fig. 1 (Abstract O20).**
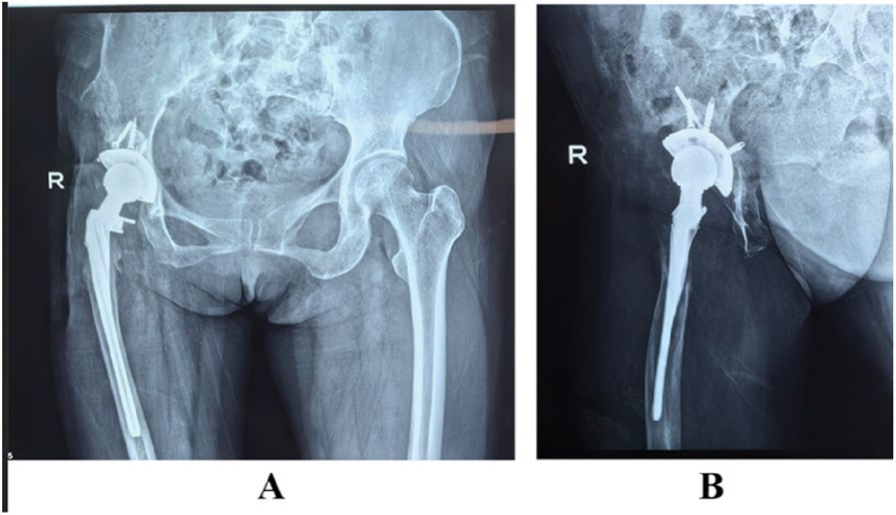
**A** Anteroposterior X-ray showing stable femoral stem fixation after right total hip arthroplasty, with partial bone absorption around the acetabulum. **B** Lateral X-ray showing partial absorption of bone around both the proximal femoral stem and the acetabulum in the right hip arthroplasty

**Fig. 2 (Abstract O20).**
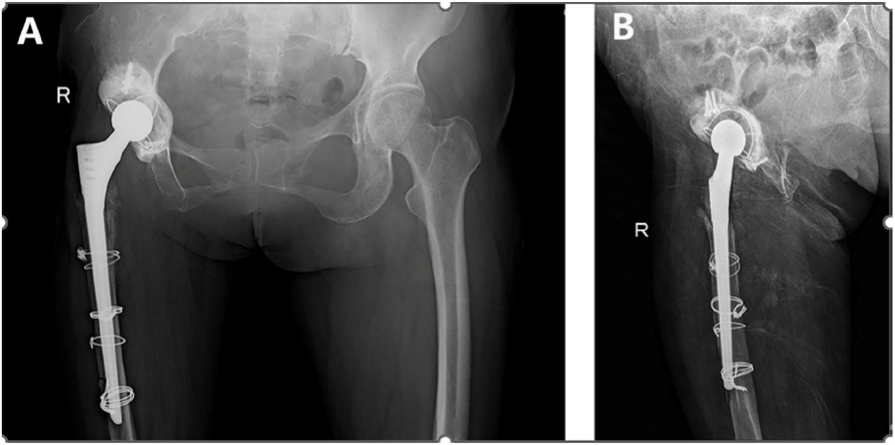
**A** Anteroposterior X-ray: due to excessive acetabular bone defects, the acetabulum is fixed using the bone cement technique. **B** Lateral X-ray: a long-stemmed cemented stem is implanted on the femoral side, with a stress fracture in the distal part of the stem

**Table 1 (Abstract O20).** Comparison of univariate and multivariate analyses of risk factors for infection control failure after one-stage revision for PJI

Risk Factor	Univariate Analysis OR (95% CI)	*P*-value	Multivariate Analysis OR (95% CI)	*P*-value
Age ≥ 65 years	4.20 (1.50–11.76)	0.006	3.21 (1.12–9.13)	0.031
Gender (Male vs Female)	3.00 (1.20–7.50)	0.020	2.78 (1.03–7.65)	0.043
Primary Disease (Rheumatoid vs Traumatic)	5.50 (1.70–17.80)	0.004	4.52 (1.38–14.76)	0.012
Infection Duration ≥ 1 year	3.50 (1.30–9.40)	0.013	2.87 (1.01–8.24)	0.048
Pathogen (Staphylococcus aureus vs Others)	3.80 (1.30–11.10)	0.015	3.14 (1.08–9.17)	0.035

## O21 Comparison of early postoperative outcomes between VELYS and MAKO robotic-assisted total knee arthroplasty

### Ren Yi Kow^1,2^, Adam Ming Yang Farid Tang^2^, Yong Ng^2^, Jeremy Tze En Lim^2^, Chun Lei Tan^2^, Ming Han Lincoln Liow^2^

#### ^1^International Islamic University Malaysia, Kuantan, Malaysia; ^2^Singapore General Hospital, Singapore, Singapore

##### **Correspondence:** Ren Yi Kow (renyi_kow@hotmail.com)

*Arthroplasty 2026*, **8(1):**O21

**Background**

Robotic-assisted total knee arthroplasty (TKA) offers precision and potential improvements in early functional recovery. While robotic-assisted TKA has been widely studied, no research to date has specifically evaluated immediate postoperative outcomes, especially on postoperative day one. This study aims to compare early functional recovery between VELYS and MAKO robotic systems and to assess how changes in Coronal Plane Alignment of the Knee (CPAK) influence these early outcomes.

**Methods**

A total of 326 patients were included in this retrospective analysis from January 2024 to January 2025, with 177 undergoing VELYS and 149 undergoing MAKO robotic-assisted TKA. The cohort comprised 210 females and 116 males, with an almost equal distribution of surgical laterality (167 left, 159 right). The mean age was 69.1 years (SD 7.6), height 157.8 cm (SD 8.8), weight 68.1 kg (SD 14.8), and BMI 27.3 kg/m^2^ (SD 5.0). Early postoperative outcomes assessed on day one included pain scores using the Visual Analogue Scale (VAS), range of motion (ROM), and walking distance.

**Results**

No significant differences were observed between the VELYS and MAKO groups for VAS during movement (VELYS 3.80 vs. MAKO 4.07) or ROM (VELYS 68.9° vs. MAKO 70.2°) on postoperative day one (Table 1). However, VELYS patients reported significantly lower VAS at rest (1.08 vs. 1.46) and achieved greater walking distances (20.6 m vs. 16.7 m) compared to MAKO patients.

For CPAK alignment analysis, only 221 patients were included due to the absence of long limb radiographs in the remaining cases. Changes in CPAK were not associated with significant differences in VAS at rest, VAS during movement, or walking distance (Table 2). However, patients with retained CPAK alignment demonstrated superior ROM (74.1° vs. 67.1°).

**Conclusions**

VELYS and MAKO robotic-assisted TKA systems yield comparable early outcomes in pain during movement and ROM. VELYS may offer advantages in pain at rest and early ambulation. CPAK alignment retention appears to contribute to improved early postoperative ROM.

**Table 1 (Abstract O21).** See text for description

Assessment	Robot	Frequency (*n*)	Mean	SD	*P*-value
VAS at rest	VELYS	177	1.08	1.49	0.030
	MAKO	149	1.46	1.63	
VAS during movement	VELYS	177	3.80	2.10	0.276
	MAKO	149	4.07	2.28	
Range of movement (^o^)	VELYS	177	68.89	22.50	0.591
	MAKO	149	70.17	20.06	
Walking distance (m)	VELYS	177	20.58	18.39	0.026
	MAKO	149	16.66	12.04	

**Table 2 (Abstract O21).** See text for description

Assessment	CPAK Change	Frequency (*n*)	Mean	SD	*P*-value
VAS at rest	No	120	1.05	1.38	0.212
	Yes	101	1.30	1.55	
VAS during movement	No	120	3.80	2.03	0.561
	Yes	101	3.97	2.32	
Range of movement (^o^)	No	120	74.06	19.07	0.013
	Yes	101	67.13	21.89	
Walking distance (m)	No	120	21.81	19.87	0.079
	Yes	101	17.89	12.84	

## O22 Automated morphological classification and measurement of the distal femur in the Chinese population based on statistical shape modeling

### Zhe Li, Qunshan Lu, Peilai Liu

#### Department of Orthopaedics, Qilu Hospital of Shandong University, Jinan, China

##### **Correspondence:** Zhe Li (601139964@qq.com)

*Arthroplasty 2026*, **8(1):**O22

**Background**

Designing prostheses that fit the anatomical characteristics of the Chinese population is crucial. However, no comprehensive classification system exists for normal distal femur morphology. Traditional manual measurement methods are labor-intensive, subjective, and lack reproducibility. Advances in computer-assisted technology now enable automated and precise measurement through machine learning approaches. This study aims to (1) establish a novel morphological classification of the normal distal femur in the Chinese population to guide prosthesis design, and (2) develop an automated measurement system for high-throughput, multi-parameter data acquisition with improved accuracy and efficiency.

**Methods**

Morphological variations were analyzed using registration algorithms, statistical shape modeling (SSM), principal component analysis (PCA), and clustering techniques. A custom automated measurement system was developed via secondary development of the 3D Slicer platform, integrating hierarchical clustering and SSM. Accuracy was validated through comparison with manual measurements, and a comprehensive morphometric analysis was conducted based on geometric transformations.

**Results**

Four distal femur types were identified: Wide type, Shallow trochlear type, Deep trochlear type, and Narrow type. The automated measurement system achieved a point matching error of 1.03 mm, a dimensional measurement error of 0.37 mm, and an angular measurement error of 0.63°. Intraclass correlation coefficients (ICCs) for automated measurements were superior to those of manual methods. A total of 33 morphological parameters were automatically measured, with results consistent with qualitative analysis findings.

**Conclusions**

A novel four-type classification of the normal distal femur in the Chinese population was established. This classification can guide prosthesis design, including the development of “wide” versions, and enhance computer-assisted preoperative planning. Additionally, a highly accurate automated measurement system based on 3D Slicer secondary development was successfully established and validated. This system enables high-throughput, multi-parameter quantitative analysis, providing a robust methodological basis for anatomical data collection to support prosthesis optimization.

## O23 Efficacy of one-stage debridement and total hip arthroplasty for acute Staphylococcus aureus-induced pyogenic arthritis of the adult hip (II-III stage)

### Yakang Wang

#### Department of Joint Surgery, Honghui Hospital of Xi’an Jiaotong University School of Medicine, Xi’an, China

##### **Correspondence:** Yakang Wang (wangyakangde@163.com)

*Arthroplasty 2026*, **8(1):**O23

**Background**

Due to the risk of recurrent infection after one-stage debridement and total hip arthroplasty (THA), clinicians often treat acute stage II-III pyogenic hip arthritis caused by Staphylococcus aureus in adults using one-stage debridement followed by two-stage THA. However, this approach incurs higher treatment costs. To evaluate the efficacy of one-stage debridement and THA in managing acute stage II-III pyogenic hip arthritis caused by S. aureus in adults.

**Methods**

A retrospective analysis was conducted on three cases of pyogenic arthritis of the hip in adults treated by a designated surgical team at our institution between August 2022 and January 2025. The study evaluated: Postoperative serum inflammatory markers, including interleukin-6 (IL-6), erythrocyte sedimentation rate (ESR), C-reactive protein (CRP), procalcitonin (PCT), white blood cell (WBC) count, and neutrophil count; The 3-month readmission rate; and Periprosthetic bone integrity assessed by hip radiographs during follow-up.

**Results**

Patients 1 and 3 demonstrated declining trends (*k* < 0) in serum IL-6, ESR, CRP, PCT, WBC count, and neutrophil count. Patient 2 maintained normal WBC and neutrophil levels throughout hospitalization, but exhibited elevated IL-6, ESR, CRP, and PCT levels upon admission that progressively declined postoperatively (*k* < 0, Fig. 1). All patients achieved 0 readmission rates within 3 months postoperation. Postoperative radiographs (taken on day 1 and at 3-month follow-up) showed no periprosthetic fractures or sclerotic bone formation around the implants (Figs. 2 and 3).

**Conclusions**

One-stage debridement and THA effectively controlled acute stage II-III S. aureus pyogenic hip arthritis in adults, with favorable periprosthetic bone stability. This study provides a theoretical basis for using one-stage debridement and THA in economically disadvantaged patients with acute S. aureus-induced pyogenic hip arthritis.

**Fig. 1 (Abstract O23).**
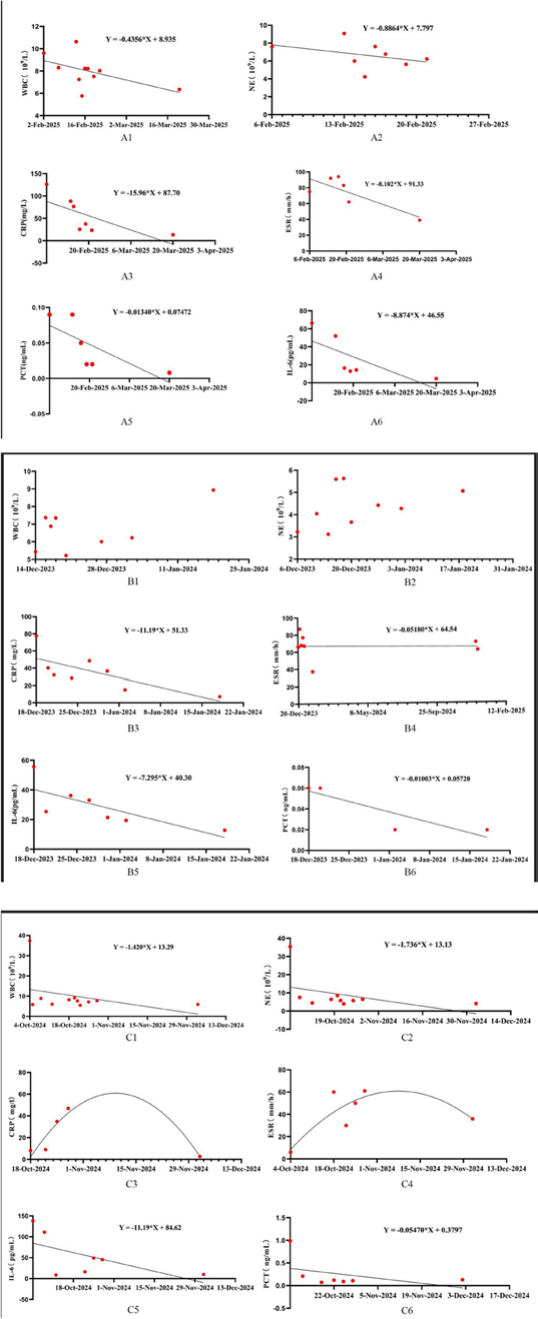
Serum leukocytes, neutrophils, C-reactive protein, erythrocyte level, interleukin-6, and procalcitonin in 3 patients with suppurative coxitis from admission to hospital until 3 months after surgery. patient 1 (A1 ~ A6), patient 2 (B1 ~ B6), and patient 3 (C1 ~ C6)

**Fig. 2 (Abstract O23).**
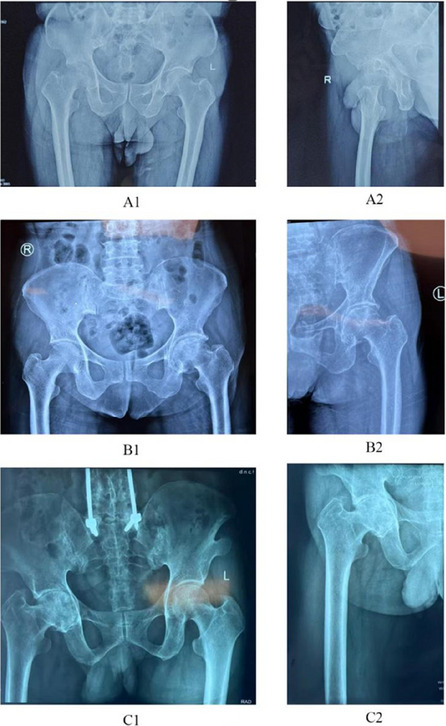
Preoperative X-rays of patients, patient 1 (A1 ~ A2), patient 2 (B1 ~ B2), and patient 3 (C1 ~ C2)

**Fig. 3 (Abstract O23).**
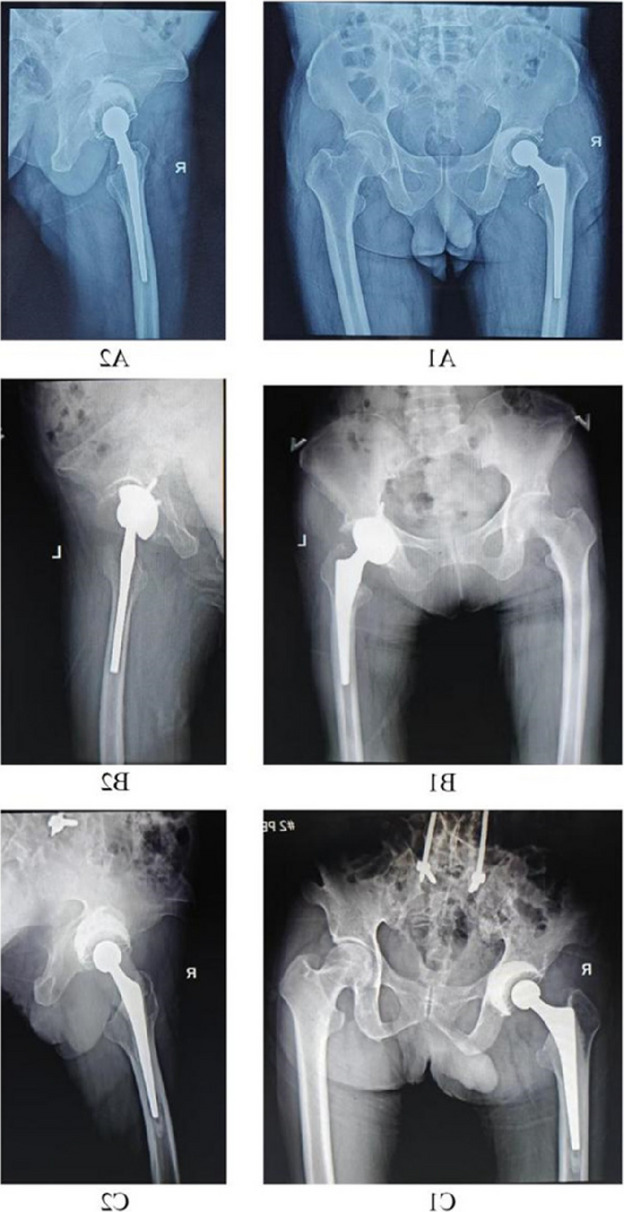
Postoperative X-rays of patients, patient 1 (A1 ~ A2), patient 2 (B1 ~ B2), and patient 3 (C1 ~ C2)

## O24 Osteoporosis and bone metabolism disorders in Chinese arthroplasty patients: a four-year retrospective cross-sectional observational study

### Xiaoxin Wu, Xinzhan Mao

#### Department of Orthopaedic Surgery, The Second Xiangya Hospital, Central South University, Changsha, China

##### **Correspondence:** Xinzhan Mao (xinzhan.mao@csu.edu.cn)

*Arthroplasty 2026*, **8(1):**O24

**Background**

Osteoporosis is a common and clinically significant comorbidity in total joint arthroplasty (TJA), particularly among patients in aging Asian populations. However, large-scale data on bone health in Chinese patients undergoing arthroplasty are lacking. This study aimed to assess the prevalence and clinical characteristics of osteoporosis and bone metabolism disorders in a large cohort of Chinese patients who underwent hip and knee arthroplasty.

**Methods**

We retrospectively analyzed 1893 patients aged ≥ 50 years who underwent elective total hip arthroplasty (THA), total knee arthroplasty (TKA), or unicompartmental knee arthroplasty (UKA) at a tertiary referral center in China from 2020 to 2024. The data collected included preoperative bone mineral density via dual-energy X-ray absorptiometry, serum bone turnover markers, 25-hydroxyvitamin D levels (25(OH)D), comorbidities, fracture history, functional status, fall risk, hospital length of stay, costs, and anti-osteoporotic treatments (Tables 1–3).

**Results**

Osteoporosis rate increased markedly with age, from 48.5% in patients aged 50–59 years to 68.8% in those aged 80–89 years. Elderly patients had the highest prevalence of both osteoporosis and vitamin D deficiency, with 25(OH)D levels < 75 nmol/L in 94.2% of the patients aged ≥ 60 years. Osteoporosis was strongly associated with older age, female sex, and several systemic comorbidities, including hypertension, cerebrovascular disease, metabolic disorders, and hepatic and renal insufficiency. These patients also had significantly lower Barthel Index scores, longer hospital stays, and higher medical costs. Although anti-osteoporotic treatment rates increased with age and disease severity, a treatment gap was noted in the oldest group, despite their higher fracture risk.

**Conclusions**

Osteoporosis and bone metabolism disorders are highly prevalent among patients undergoing arthroplasty, especially in elderly women and patients with femoral neck fractures. As periprosthetic fractures are now a leading cause of revision, particularly in THA, early treatment of osteoporosis may lead to a decrease in revision burden.

**Table 1 (Abstract O24).** Baseline characteristics and bone health in arthroplasty patients

	**THA**			**TKA**			**UKA**		
	**Male**	**Female**	**Total**	**Male**	**Female**	**Total**	**Male**	**Female**	**Total**
N	278	439	717	221	752	973	47	156	203
Age(year)	65.97	65.06	65.41	70.14	68.15	68.60	65.63	64.16	64.50
BMI(kg/m^2)	24.52	24.28	24.37	25.44	25.49	25.48	24.83	25.82	25.59
Smoking	88(31.7%)	7(1.6%)	95(13.2%)	36(16.3%)	7(0.9%)	43(4.4%)	4(8.5%)	5(3.2%)	9(4.4%)
Drinking	79(28.4%)	2(0.5%)	81(11.3%)	30(13.6%)	14(1.9%)	44(4.5%)	2(4.3%)	11(7.0%)	13(6.4%)
T-Neck	-1.48	-2.08	-1.85	-1.69	-2.27	-2.14	-1.29	-1.70	-1.61
T-Lumbar spine	-2.09	-2.25	-2.19	-1.68	-2.34	-2.19	-1.79	-2.30	-2.18
BMD tested	135(48.6%)	301(68.6%)	436(60.8%)	137(65.6%)	572(74.9%)	709(72.8%)	27(57.4%)	118(75.6%)	145(71.4%)
Normal	20(14.8%)	33(11.0%)	53(12.2%)	25(18.2%)	43(7.5%)	68(9.6%)	4(14.8%)	10(8.5%)	14(9.7%)
Osteopenia	50(37.0%)	105(34.9%)	155 (35.6%)	54(39.4%)	157(27.4%)	211(19.8%)	10(37.0%)	45(38.1%)	55(37.9%)
Osteoporosis	65(48.1%)	163(54.2%)	228(52.3%)	58(42.3%)	372(65.0%)	430(60.6%)	6(22.2%)	70(59.3%)	76(52.4%)
Fracture	7(2.5%)	5(1.1%)	12(1.7%)	4(1.8%)	14(1.9%)	18(1.8%)	2(4.3%)	2(1.3%)	4(2.0%)
FRAX-MOF(%)	9.21	7.95	8.44	7.93	8.68	8.53	7.73	7.97	7.93
FRAX-Hip(%)	5.48	5.94	5.76	5.55	6.08	5.97	5.41	5.58	5.55
PTH(pg/ml)	56.68	52.70	54.16	50.65	58.13	56.43	46.55	55.49	53.42
25VD(nmol/L)	51.99	44.52	47.25	52.60	44.43	46.29	52.13	45.55	47.07
BGP(ng/ml)	20.94	22.22	21.75	16.26	22.11	20.78	15.27	20.58	19.35
CTX(pg/ml)	598.70	717.67	674.12	494.57	692.33	647.41	411.70	575.50	537.58
PINP(ng/ml)	79.21	74.60	76.29	65.18	74.01	72.00	72.06	83.79	81.07
Ga2^+^(mmol/L)	2.22	2.26	2.25	2.25	2.26	2.26	2.23	2.26	2.25
Crea(μmol/L)	91.14	63.09	73.36	86.74	62.53	68.03	77.80	60.33	64.37

*THA* total hip arthroplasty, *TKA* total knee arthroplasty, *UKA* unicompartmental knee arthroplasty, *MOF* major osteoporotic fracture, *PTH* parathyroid hormone, *25VD* 25(OH)D, *BGP* osteocalcin, *CTX* C-terminal telopeptide, *PINP* procollagen type I N-propeptide, *Crea* creatinine

**Table 2 (Abstract O24).** Functional status, fall risk, and healthcare resource utilization in arthroplasty patients

	**Joint Arthroplasty**		
	**Non-osteoporosis**	**Osteoporosis**	***P* value**
Morse Fall Scale	28	29	0.2621
Barthel Index	91	83	< 0.0001
Hospitalization costs	33,190.64	34,758.30	< 0.0001
Length of hospitalization	9	10	< 0.0001

**Table 3 (Abstract O24).** Association between osteoporosis and comorbidities in arthroplasty patients

	**Joint Arthroplasty**			
	**Non-osteoporosis**	**Osteoporosis**	**Total**	***P* value**
Hypertension				< 0.0001
No	369	366	735	
Yes	187(33.63%)	368(50.14%)	555	
Diabetes				0.7322
No	455	606	1061	
Yes	101(18.17%)	128(17.44%)	229	
Coronary artery disease				0.0536
No	520	666	1186	
Yes	36(6.47%)	68(9.26%)	104	
Arrhythmia/Heart Failure/Valve Disease				0.0622
No	546	709	1255	
Yes	10(1.08%)	25(3.41%)	35	
Post-total hysterectomy				0.1545
No	511	689	1200	
Yes	45(8.09%)	45(6.13%)	90	
Cerebrovascular disease				0.0037
No	511	639	1150	
Yes	45(8.09%)	95(12.94%)	140	
Metabolic disorder				< 0.0001
No	527	651	1178	
Yes	29(5.22%)	83(11.31%)	112	
Hepatic insufficiency				< 0.0001
No	528	610	1138	
Yes	28(5.04%)	124(16.89%)	152	
Renal insufficiency				0.004
No	528	667	1195	
Yes	28(5.04%)	67(9.13%)	95	
Hyperthyroidism/hypothyroidism				0.5609
No	542	712	1254	
Yes	14(2.52%)	22(3.00%)	36	
History of malignant tumors				0.2255
No	536	716	1252	
Yes	20(3.60%)	18(2.45%)	38	
Anemia				0.0821
No	527	679	1206	
Yes	29(5.22%)	55(7.49%)	84	

## O25 Variations in greater trochanter clear space make proximal femoral array use challenging

### Dirk van Bavel

#### Epworth Healthcare, Melbourne, Australia

##### **Correspondence:** Dirk van Bavel (dvanbavel@bigpond.com)

*Arthroplasty 2026*, **8(1):**O25

**Background**

Total hip arthroplasty is a successful operation, but instability is the second most common reason for revision in the first 11 years. Prosthetic Stability is determined by a combination of factors, including component positioning, soft tissue factors, and spino-pelvic biomechanics. There is extensive literature focusing on the positioning of the acetabular component and, more recently, technological aids to help execute planned cup positioning. Stem positioning is, however, not well studied, and its execution is poor. The enhanced workflow with the Stryker MAKO™ system allows the femoral broach and stem to be tracked, allowing measurements of leg length, offset, and stem version. This, however, requires the insertion of an 8 mm screw into the Greater Trochanter for positioning of an array. There are concerns that there is not enough bony space available for the insertion of the necessary screw to track the femoral component.

**Methods**

MAKO CT scans of 202 consecutive patients were reviewed. These were planned for both the Exeter stem (Fig. 1) and an Accolade II stem (Fig. 2). The stems were set at 10 degrees anteversion with appropriate stem size and aiming to match the leg length and offset of the opposite side. The maximum distance from the edge of the stem to the perpendicular inner cortex of the greater trochanter was measured and recorded as the Greater Trochanter Clear Space (GTCS).

**Results**

There were 202 primary hip replacement plans recorded in 109 females and 93 males. The average age of the patients was 67. For Exeter stems, the GTCS mean was 12 mm (SD = 2.92, Range 6–20 mm), which was typically low in the GT. For Accolade II stems, the GTCS mean was 18 mm (SD = 3.07, Range 10–25 mm), which was typically high in the GT. If we consider the 8 mm screw requiring 4 mm on either side of it, only 6% of Exeter stems, but 89% of Accolade II stems had the required 16 mm of GTCS (Fig. 3).

**Conclusion**

While the MAKO Enhanced workflow is a useful aid to femoral component positioning, a large percentage of patients do not have enough Greater Trochanter Clear Space to use the proprietary system with the Exeter stem. Although we are aware of individual surgeons who have created their own solutions to this problem, a widely available solution would be of benefit.

**Fig. 1 (Abstract O25).**
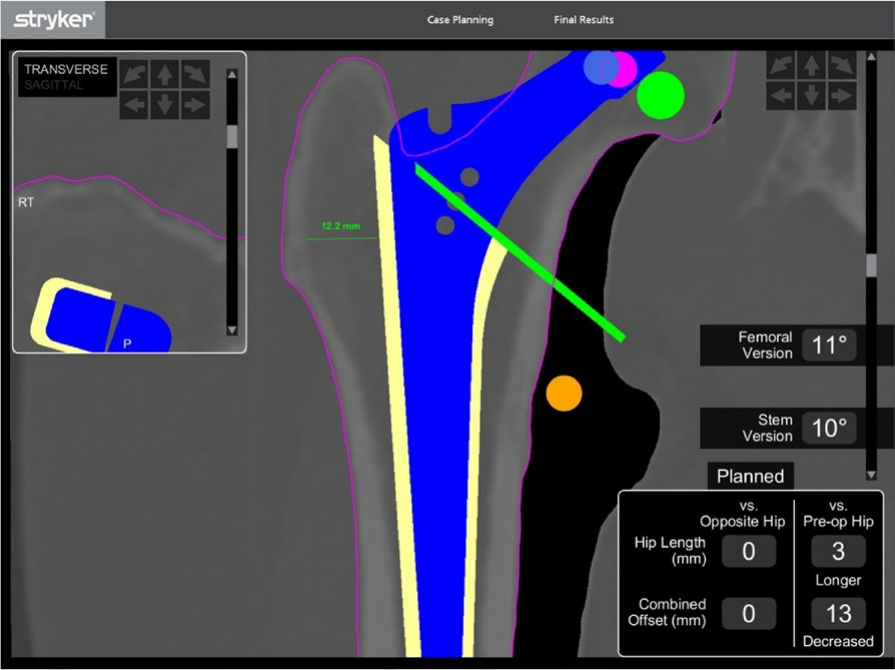
MAKO plan of Exeter stem showing the site of the typical maximal Greater Trochanter Clear Space

**Fig. 2 (Abstract O25).**
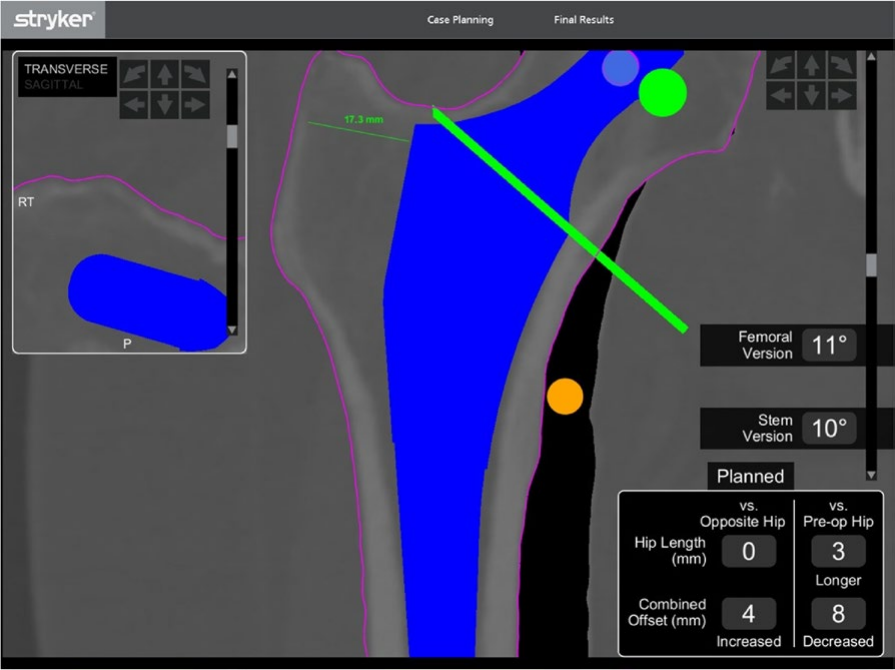
MAKO plan of Accolade II stem (of the same patient as Fig. 1 above) showing the site of typical maximal Greater Trochanter Clear Space

**Fig. 3 (Abstract O25).**
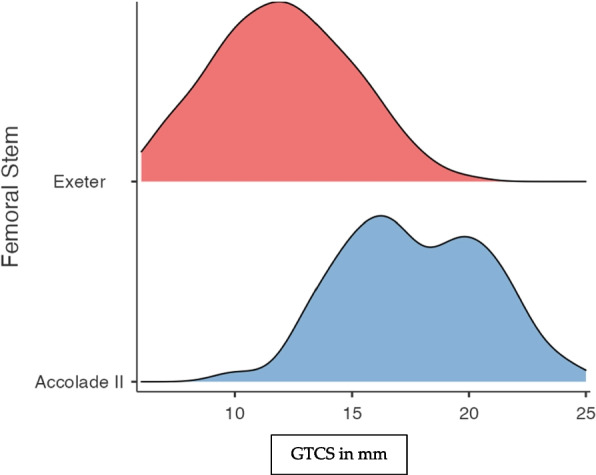
Density histogram displaying the Greater Trochanter Clear Space (in mm) for the Accolade II and Exeter stems

## O26 Ground kinematic alignment in high tibial osteotomy achieves identical clinical outcomes to classic alignment for constitutional varus knees: a short-term comparative cohort study

### Weidong Weng^1^, Shangjin Lin^1^, Lu Wang^1^, Fanghui Wu^2^, Lei Zhang^1,2^

#### ^1^Department of Orthopaedics, The First Affiliated Hospital of Wenzhou Medical University, Wenzhou, China; ^2^Department of Orthopaedics, The Third Affiliated Hospital of Wenzhou Medical University, Rui’an, China

##### **Correspondence:** Lei Zhang (zhanglei@wmu.edu.cn)

*Arthroplasty 2026*, **8(1):**26

**Background**

Constitutional varus, affecting 18%–34% of healthy adults, features compensatory medial tibial plateau parallelism during weight-bearing. While kinematic alignment (KA) principles have revolutionized arthroplasty for constitutional varus knees by prioritizing native joint line restoration over mechanical axis correction, their application in high tibial osteotomy (HTO) remains underexplored. Traditional Fujisawa-targeted HTO often requires excessive osteotomy gaps to achieve 60–62.5% weight-bearing line (WBL) correction, elevating risks of delayed union and implant failure. However, HTO protocols remain contentious, particularly regarding optimal alignment targets. This study introduces ground kinematic alignment (GKA)-HTO, a novel protocol replicating contralateral knee joint line obliquity (KJLO), to address these biomechanical challenges. We hypothesize that GKA-HTO achieves equivalent clinical outcomes with reduced surgical morbidity compared to conventional alignment strategies.

**Methods**

This prospective comparative cohort study enrolled 88 patients (mean age 54.3 ± 6.1 years) with unilateral medial compartment osteoarthritis and asymptomatic contralateral constitutional varus (HKA > 3° varus). Patients underwent CT-based 3D planning and were randomized to Fujisawa-targeted HTO (Group F, *n* = 47; target WBL 62.5% tibial plateau) or GKA-HTO (Group G, *n* = 41; target contralateral KJLO). Both groups utilized 3D-printed patient-specific instrument (PSI) guides. Primary outcomes included osteotomy gap width, functional scores (Lysholm, HSS), radiographic parameters (HKA, WBL, medial proximal tibial angle [MPTA], posterior tibial slope [PTS], joint line convergence angle [JLCA]), medial meniscus extrusion (MME), and osteoarthritis progression (K-L grade, Outerbridge classification). The clinical and radiographic outcomes and second-look arthroscopic findings were statistically compared for comprehensive assessments.

**Results**

No statistical differences were found between the two groups concerning the demographic characteristics. After a mean follow-up of 37.1 months, Group G demonstrated significantly smaller osteotomy gaps (4.2 ± 1.2 mm vs. 8.3 ± 1.8 mm, *P* < 0.001) when compared to Group F. No significant differences were observed between the Group F and G regarding the functional outcomes were comparable (Lysholm: 84.1 ± 3.7 vs. 82.3 ± 4.1, *P* = 0.213; HSS: 84.2 ± 3.3 vs. 86.5 ± 4.9, *P* = 0.407). However, we only observed significant differences between the two groups in terms of the final hip–knee–ankle angle, weight-bearing line ratio, and medial proximal tibial angle (*P* < 0.001, respectively). Radiographically, the mean KJLO of the contralateral limb was 0.7° ± 0.4° (range, − 0.2° to 1.8°). Postoperatively, Group G achieved near-physiological joint line obliquity (KJLO discrepancy 0.5° ± 0.1°, ranged 0 to 1.1°) when compared to Group F (3.8° ± 1.5°, *P* < 0.001). Second-look arthroscopy did not reveal a significant difference in meniscal healing rate between the two groups (*P* = 0.786).

**Conclusions**

As the first comparative study implementing KA principles in HTO, PSI-guided GKA-HTO reduces osteotomy gaps and preserves joint line orientation without compromising clinical outcomes at a short-term follow-up, offering a biomechanically superior alternative to Fujisawa-targeted HTO in constitutionally varus patients. Our study establishes a novel reference standard for constitutional varus correction. Study limitations include a single-center design and mid-term follow-up. Future multicenter trials should validate these findings across diverse populations and assess long-term osteoarthritis progression.

## O27 Immersive virtual reality simulation training enhances technical skill acquisition and procedural accuracy in cadaveric total knee arthroplasty

### Prevheenraj Naidu a/l Thevaraj, Khairul Anwar Ayob, Azlina Amir Abbas, Sik Loo Tan, Li Ping Wong, Kwong Weng Loh

#### Faculty of Medicine, Universiti Malaya, Kuala Lumpur, Malaysia

##### **Correspondence:** Prevheenraj Naidu a/l Thevaraj (prevheen@gmail.com)

*Arthroplasty 2026*, **8(1):**O27

**Background**

Although immersive virtual reality (iVR) is used for training in various surgical procedures, its efficacy in complex multistep orthopaedic surgeries is less established. IVR training allows surgical trainees to better achieve skills in important steps in surgical procedures. This study aimed to evaluate the effectiveness of iVR simulation training in comparison to the surgical technique guide and video for fixed-bearing total knee replacement (TKA).

**Methods**

Twenty-two orthopaedic surgery residents from a local university were randomized into two groups: one receiving surgical training for TKA using a surgical technique guide and instructional video (control group, CG), and the other using an iVR training module (VR group). Following the training, participants performed TKA on cadavers with standardized surgical instruments and implants. Subjective and objective data were collected before, during, and after the procedure. Two blinded evaluators were tasked to obtain objective data during cadaver dissection.

**Results**

Residents from a single institution were randomized equally (*n* = 11) in the VR and CG groups. The VR group achieved significantly higher scores in instrument handling and flow of operation compared to the CG group on the Objective Structured Assessment of Technical Skills (Table 1). The VR group performed significantly better than the CG group in procedure-based assessment (VR 4.3 vs CG 2.9, *P* < 0.001). VR group achieved a significantly higher mean score for correct steps performed than the CG group (VR 29.7 ± 2.9, CR 25.8 ± 5.8, *P* = 0.05). Although the VR group completes the procedure faster (54.8 ± 14.8 min), this was not found to be statistically significant when compared to CG (55.4 ± 10.9 min, *P* = 0.93). The VR group would recommend this training method to other trainees and acknowledged that it significantly increased their confidence in performing TKA.

**Conclusions**

Although there was no significant difference in TKA completion times between the VR and control groups, the VR group demonstrated superior performance in specific skill assessments and executed a higher number of TKA procedural steps correctly. Positive trainee feedback suggests iVR training is well-received and has potential for enhancing surgical education in orthopedics.

**Table 1 (Abstract O27).** Surgical performance of participants

	**Control group (min)**	**Virtual Reality group (min)**	***P*-value**
Operative time	55.4 (10.9)	54.8 (14.8)	0.93
Procedure-specific checklist			
Total score (max 35)	25.8 (5.8)	29.7 (2.9)	**0.05**
Global assessment categories			
Respect for tissue	3.1 (0.7)	3.6 (0.5)	0.10
Time and motion	2.7 (0.7)	3.1 (0.9)	0.31
Instrument handling	2.5(0.7)	3.3 (0.5)	**0.01**
Knowledge of instruments	2.7 (0.8)	3.0 (0.8)	0.49
Flow of operation	2.7 (0.5)	3.5 (0.8)	**0.02**
Use of assistants	2.8 (0.9)	3.3 (0.8)	0.24
Knowledge of a specific procedure	2.9 (0.9)	3.4 (0.8)	0.10
Procedure-based assessment			
Global summary score	2.9 (0.8)	4.3 (0.6)	**< 0.01**

## O28 A comparative study of early plantar pressure after total hip replacement via direct anterior approach and posterolateral approach in patients with femoral neck fractures

### Wei Guo^1,2^, Wenjian Yang^1,2^, Chao Cheng^1,2,3^, Hua Yang^1,2^

#### ^1^Department of Orthopaedics, Yiyang Central Hospital, Yiyang, China; ^2^Clinical Medical Technology Demonstration Base for Minimally Invasive and Digital Orthopaedics in Hunan Province, Yiyang, China; ^3^The fourth people’s hospital of Yiyang city, Yiyang, China

##### **Correspondence:** Yang Hua (250455762@qq.com)

*Arthroplasty 2026*, **8(1):**O28

**Background**

Plantar Pressure is a key parameter for measuring gait and lower extremity function, and its distribution characteristics can reflect the weight-bearing distribution and joint stability of patients during walking. The early changes of plantar pressure are closely related to the postoperative rehabilitation process, joint stability, and functional recovery.

**Methods**

Patients with femoral neck fractures who visited the Department of Joint and Sports Medicine at Yiyang Central Hospital from January to October 2024 and consented to undergo total hip arthroplasty were selected. A total of 63 patients were included. The surgical patients were randomly divided into two groups based on the inclusion and exclusion criteria: the direct anterior approach (DAA) group and the posterolateral approach (PLA) group. The study compared the data on step length, plantar pressure difference, and hip joint mobility in the early postoperative walking period between patients who underwent minimally invasive direct anterior approach hip arthroplasty and those who underwent traditional posterolateral approach hip arthroplasty. Briefly describe the main methods or treatments applied (Tables 1–3).

**Results**

1. As the postoperative time extended, both groups of patients showed gradual improvements in step length, plantar pressure difference, and hip joint mobility. One week after surgery, the DAA group had significantly better results in step length and plantar pressure difference compared to the PLA group (*P* < 0.001). The DAA group also had a better 2-min walking test result compared to the PLA group (*P* = 0.011).
2. One month after surgery, the DAA group had significantly better results in step length and plantar pressure difference compared to the PLA group (*P* < 0.001). The DAA group also had a significantly better 2-min walking test result compared to the PLA group (*P* = 0.013).
3. Three months after surgery, the DAA group had better results in step length and plantar pressure difference compared to the PLA group (*P* < 0.001). The DAA group had a significantly better 2-min walking test result compared to the PLA group (*P* < 0.001).

**Conclusions**

The recovery of step length, plantar pressure, and hip joint mobility was inconsistent between the two groups of patients after surgery. Patients in the direct anterior approach group exhibited significantly better step length, plantar pressure difference, and hip joint mobility in the early postoperative period compared to those in the posterior approach group.

**Table 1 (Abstract O28).** Step size test results (cm)

	**Group**	**Sample size**	**Mean (standard deviation)**	***P***
1 week after surgery	DAA	30	44.601 (3.379)	< 0.001
	PLA	33	41.230 (2.946)	
1 month after surgery	DAA	30	46.344 (3.325)	< 0.001
	PLA	33	43.237 (2.727)	
3 months after surgery	DAA	30	51.227 (3.450)	< 0.001
	PLA	33	45.862 (2.582)	

**Table 2 (Abstract O28).** Comparison of plantar pressure difference test results (%)

	**Group**	**Sample size**	**Mean (standard deviation)**	***P***
1 week after surgery	DAA	30	15.146 (1.607)	< 0.001
	PLA	33	20.996 (1.924)	
1 month after surgery	DAA	30	9.841 (1.706)	< 0.001
	PLA	33	15.906 (2.090)	
3 months after surgery	DAA	30	7.053 (1.370)	< 0.001
	PLA	33	9.765 (1.983)	

**Table 3 (Abstract O28).** Comparison of 2-min walk test results (m)

	**Groups**	**Sample size**	**Mean (standard deviation)**	***P***
1 week after surgery	DAA	30	111.896 (7.082)	0.011
	PLA	33	107.362 (6.684)	
1 month after surgery	DAA	30	120.251 (5.183)	0.013
	PLA	33	116.154 (7.359)	
3 months after surgery	DAA	30	130.810 (3.789)	< 0.001
	PLA	33	121.167 (6.820)	

## O29 Analysis of failure causes and risk prediction of debridement, antibiotics, and implant retention (DAIR) for acute periprosthetic joint infection

### Maocan Cai^1,2,3,4^, Yiming Lin^1,2,3,4^, Yishan Xin^1,2,3,4^, Hongyan Li^1,2,3,4^, Zida Huang^1,2,3,4^, Guochang Bai^1,2,3,4^, Ye Yang^1,2,3,4^, Zeyu Zhang^1,2,3,4^, Yufeng Guo^5^, Chengguo Huang^6^, Wenbo Li^1,2,3,4^, Xinyu Fang^1,2,3,4^, Wenming Zhang^1,2,3,4^, Chaofan Zhang^1,2,3,4^

#### ^1^Department of Orthopaedic Surgery, the First Affiliated Hospital, Fujian Medical University, Fuzhou, China; ^2^Department of Orthopaedic Surgery, National Regional Medical Center, Binhai Campus of the First Affiliated Hospital, Fujian Medical University, Fuzhou, China; ^3^Fujian Provincial Institute of Orthopedics, the First Affiliated Hospital, Fujian Medical University, Fuzhou, China; ^4^Fujian Orthopaedic Bone and Joint Disease and Sports Rehabilitation Clinical Medical Research; ^5^Department of Orthopaedic Surgery, Changtai County Hospital, Zhangzhou, China; ^6^Department of Orthopaedic Surgery, Pingnan County Hospital, Ningde, China

##### **Correspondence:** Chaofan Zhang (drcfzhang@fjmu.edu.cn); Wenming Zhang (zhangwm0591@fjmu.edu.cn)

*Arthroplasty 2026*, **8(1):**O29

**Background**

Debridement, antibiotics, and implant retention (DAIR) is the preferred treatment for acute periprosthetic joint infection (PJI), yet its failure rate remains high, and the influencing factors are not fully elucidated. This study aimed to investigate the causes of DAIR failure in acute PJI and construct a risk prediction model based on clinical characteristics, inflammatory markers, and microbiological data.

**Methods**

A retrospective analysis was conducted on 90 patients with acute PJI treated at our medical center between January 2008 and April 2024. All patients underwent standard DAIR treatment and were categorized into success (*n* = 77) and failure (*n* = 13) groups based on outcomes. Demographic data, infection characteristics, laboratory markers, microbiological results, and surgical details were collected. Univariate and multivariate logistic regression analyses were performed to identify independent risk factors, and a nomogram prediction model was developed.

**Results**

The overall success rate of DAIR was 85.6% (77/90). In the success group, microbial identification was achieved in 65 cases, while all patients in the failure group had confirmed microbiological results (Fig. 1). The failure group exhibited significantly higher rates of knee joint infection (84.6% vs. 50.6%, *P* = 0.023), acute hematogenous infection (61.5% vs. 20.8%, *P* = 0.005), preoperative peripheral White Blood Cell (WBC) (9.5 × 10^9^/L vs. 8.2 × 10^9^/L, *P* = 0.043), CRP (79.6 4 mg/L vs. 42.4 mg/L, *P* < 0.001), ESR (80.6 mm/h vs. 60.5 mm/h, *P* = 0.002), synovial fluid WBC (35,300 × 10^6^/L vs. 21,843 × 10^6^/L, *P* = 0.043), and synovial fluid polymorphonuclear leukocytes (PMNs) (91.7% vs. 83.8%, *P* < 0.001) compared to the success group. Multivariate logistic regression (Table 1) identified acute hematogenous infection (OR 11.704, 95% CI 1.957–119.357, *P* = 0.015), preoperative CRP (OR 1.022, 95% CI 1.009–1.040, *P* = 0.003), synovial fluid PMN% (OR 1.196, 95% CI 1.039–1.454, *P* = 0.039), and resistant pathogens (OR 0.107, 95% CI 0.010–0.665, *P* = 0.032) as independent risk factors for DAIR failure. The nomogram (Fig. 2) model based on these factors demonstrated robust predictive performance.

**Conclusions**

DAIR failure is closely associated with hematogenous infection, the intensity of the inflammatory response, and the presence of resistant pathogens. The proposed risk prediction model may aid clinical decision-making and optimize patient selection for DAIR.

**Fig. 1 (Abstract O29).**
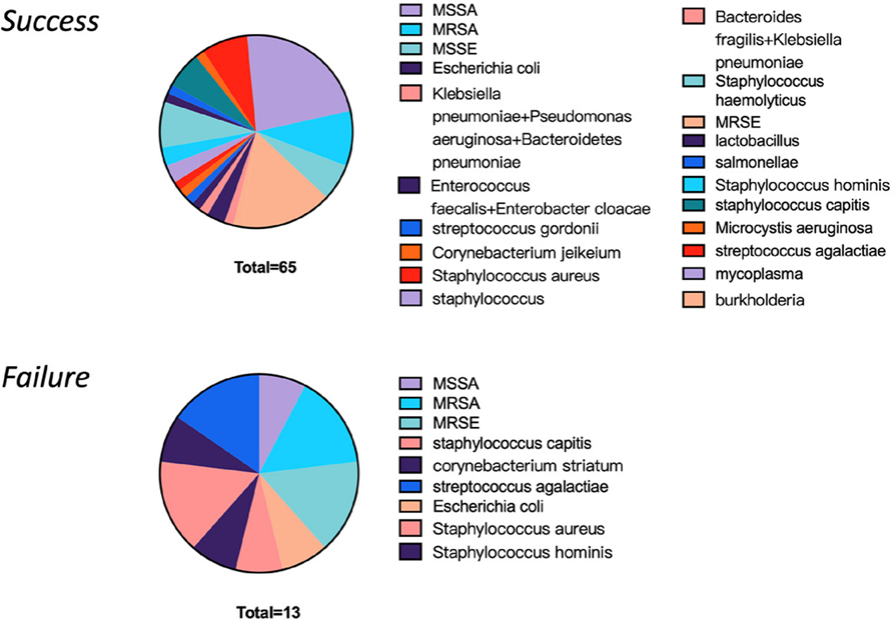
Microbiological identification results of the success and failure groups

**Fig. 2 (Abstract O29).**
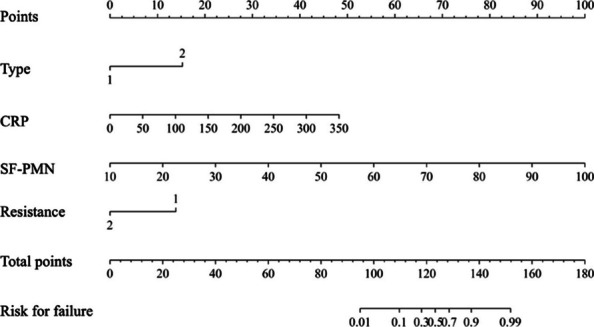
Nomogram for predicting the risk of treatment failure based on multivariate logistic regression analysis. For clinical application: (1) Locate the patient's clinical values (e.g., infection type, preoperative CRP) on the corresponding variable axes; (2) Project upward to determine the points for each parameter; (3) Sum all points to obtain the total score (range: 0–180); (4) Project downward from the total score to the “Risk of Treatment Failure” axis to determine the individualized probability of DAIR failure. (For infection type, “1” means “Tsukayama 2” and “2” means “Tsukayama 3”. For resistance, “1” means “yes” and “2” means “no”. SF-PMN: synovial fluid polymorphonuclear leukocytes)

**Table 1 (Abstract O29).** The multivariate regression analysis of the success and failure groups

Variable	β	SE	z	OR (95%CI)	*P* value
Type					
Tsukayama 2	0.0			reference	
Tsukayama 3	2.5	1.0	2.4	11.7 (2.0,119.4)	0.015
CRP	0.0	0.0	2.9	1.0 (1.0,1.0)	0.003
Synovial PMN	0.2	0.1	2.1	1.2 (1.0,1.5)	0.039
Antimicrobial resistance					
Resistant	0.0			reference	
Sensitive	− 2.2	1.0	− 2.1	0.1 (0.0,0.7)	0.032

*PMN* polymorphonuclear leukocytes

## O30 Clinical study on high-aged unstable intertrochanteric fractures of hip joint replacement through the fracture line

### Jinliang Wang, Bo Sun, Wei Mei, Qingde Wang, Shaohua Wang, Xuan Wei

#### Zhengzhou Orthopaedic Hospital, Zhengzhou, China

##### **Correspondence:** Jinliang Wang (jiajiawaers@126.com)

*Arthroplasty 2026*, **8(1):**O30

**Background**

This study introduces a new approach called the “Via Fracture Line Approach” (VFLA) for treating high-risk, unstable intertrochanteric fractures in elderly patients. The VFLA approach involves entering the hip directly through the coronal surface of the greater trochanter, without disrupting the gluteus medius, external rotators, anterior and posterior joint capsules. This approach is theoretically less likely to result in dislocation compared to other approaches. The study compared the VFLA approach with the modified Harding approach (MHA) and demonstrated its effectiveness and clinical outcomes.

**Methods**

The study included 79 patients who underwent hip arthroplasty for unstable intertrochanteric fractures between July 2018 and June 2023. There were 17 male and 62 female patients, Ao type A2:61 cases and type A3:18 cases. The VFLA group included 33 patients who underwent surgery using the VFLA, while the MHA group included 46 patients who underwent surgery using the MHA. The average age of the VFLA group was 83.3 ± 8.4 years, while the average age of the MHA group was 85.4 ± 7.3 years. The patients were evaluated using the Harris hip score to assess clinical outcomes, and the imaging findings were analyzed. The incidence of related complications was also recorded.

**Results**

The overall 1-year mortality rate was 10% in both groups. Both groups achieved good postoperative functional scores, with no significant difference in the Harris hip score between the 1-month and 3-month follow-up. The VFLA group had shorter operation times, less blood loss, and smaller differences in leg length compared to the MHA group, with statistically significant differences. However, there was no difference in the incidence of transfusion and time to weight-bearing. In terms of complications, there were no incidents of vascular or neural injury, and there were 3 cases of infection, 4 cases of deep vein thrombosis, 2 cases of prosthesis loosening, and a total dislocation rate of 7%. The dislocation rate in the VFLA group (0%) was lower than that in the MHA group (13%), and the fracture healing rate was 91%, with no significant difference between the two groups (VFLA: 96% vs MHA: 86%).

**Conclusions**

Applying the VFLA in hip arthroplasty for unstable intertrochanteric fractures in elderly patients, by exposing the fracture site through the original fracture line, preserving the hip peripheral muscle groups and joint capsule, and reducing soft tissue dissection, results in a low postoperative dislocation rate and high fracture healing rate. The overall incidence of complications is low, and the clinical efficacy is satisfactory.

## O31 Negative influence of joint line change on long-term outcomes and survival of unicompartmental knee arthroplasty

### Hong Yeol Yang, Jong Keun Seon, Sung Ju Kang, Jong Eun Kim, Woo Jin Jeong

#### Department of Orthopaedic Surgery, Chonnam National University Hwasun Hospital, College of Medicine, Chonnam National University, 322 Seoyang-ro, Hwasun, Republic of Korea

##### **Correspondence:** Jong Keun Seon (seonbell@chonnam.ac.kr)

*Arthroplasty 2026*, **8(1):**O31

**Background**

This study aimed to investigate the effect of changes in joint line height on the long-term clinical outcomes and survival rates of unicompartmental knee arthroplasty (UKA) for medial compartmental knee osteoarthritis.

**Methods**

We performed a retrospective analysis of 205 medial fixed-bearing UKAs performed between March 2005 and December 2017, with a minimum follow-up of 5 years. Patients were divided into two groups based on the degree of joint line restitution following surgery: the restored joint line group (joint line change < 2 mm; *n* = 125) and joint line lowering group (joint line change ≥ 2 mm; *n* = 80) (Fig. 1). Radiographic evaluations included the mechanical hip-knee-ankle angle, medial proximal tibial angle, lateral distal femoral angle, joint line convergence angle, and posterior tibial slope. Kaplan–Meier survival analysis was performed using conversion to total knee arthroplasty as the endpoint. Factors potentially influencing implant survival were also analyzed.

**Results**

During the follow-up period, 13 implant failures were identified, with aseptic loosening being the most commonly reported mode of failure, accounting for 46.2% of revisions (Table 1). At a mean follow-up of 10.8 years, survival rates were significantly lower in the joint line-lowering group (90.7% [95% CI, 80.1%–95.8%]) compared to the restored joint line group (97.5% [95% CI, 89.8%–99.4%]; *P* = 0.014, log-rank test) (Fig. 2). Multivariate regression analysis identified higher body mass index (hazard ratio [HR], 1.320; *P* = 0.035) and joint line lowering (HR, 1.532; *P* = 0.038) as independent risk factors for implant failure.

**Conclusions**

Joint line lowering ≥ 2 mm was associated with significantly lower survival rates following UKA at a mean follow-up of 10.8 years. These findings underscore the importance of restoring the joint line to achieve optimal long-term results after UKA for medial compartmental knee osteoarthritis.

**Fig. 1 (Abstract O31).**
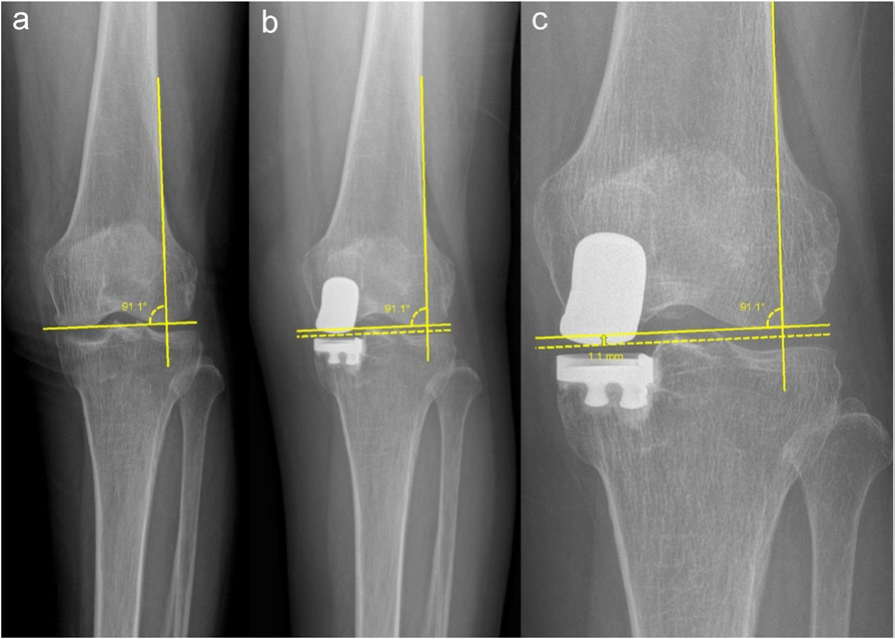
Methods of measurement of joint line height change. **a** On the preoperative radiograph, an angle is formed between a line tangential to the lateral femoral cortex and a line passing through the most distal points of the femoral condyles. **b** This reference angle is then applied to the postoperative radiograph, and the distance between the most distal point of the femoral component and the reproduced line is defined as the joint line height change, as described and validated by Herry et al. (**c**). In this patient, a joint line distalization of 1.1 mm is observed

**Fig. 2 (Abstract O31).**
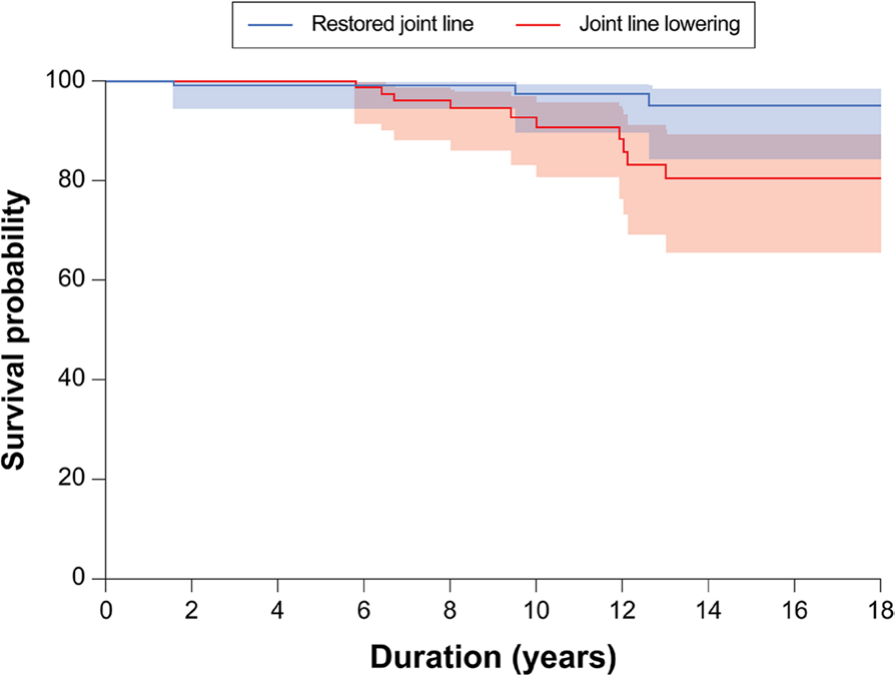
Methods of measurement of joint line height change. **a** On the preoperative radiograph, an angle is formed between a line tangential to the lateral femoral cortex and a line passing through the most distal points of the femoral condyles. **b** This reference angle is then applied to the postoperative radiograph, and the distance between the most distal point of the femoral component and the reproduced line is defined as the joint line height change, as described and validated by Herry et al. (**c**). In this patient, a joint line distalization of 1.1 mm is observed

**Table 1 (Abstract O31).** Overview of UKA cases requiring conversion to TKA*

Case	Sex	Age *(yr)*	Body mass index *(kg/m2)*	Time to revision *(yr)*	Reason for Failure	Procedure
1	Female	53.3	29.8	12.1	Polyethylene wear	Revision TKA
2	Female	61.2	28.7	11.9	Unexplained pain	Revision TKA
3	Female	67.9	25.0	1.6	Aseptic loosening	Revision TKA
4	Female	69.0	24.1	8.0	Aseptic loosening	Revision TKA
5	Male	65.1	27.8	13.0	Polyethylene wear	Revision TKA
6	Female	67.6	22.5	5.9	Aseptic loosening	Revision TKA
7	Female	57.0	24.7	12.6	Aseptic loosening	Revision TKA
8	Female	68.1	25.4	9.4	Polyethylene wear	Revision TKA
9	Female	61.9	28.8	11.9	Aseptic loosening	Revision TKA
10	Female	54.3	25.6	10.0	Polyethylene wear	Revision TKA
11	Female	74.3	30.3	10.5	Polyethylene wear	Revision TKA
12	Female	69.7	31.2	6.7	Progression of lateral osteoarthritis	Revision TKA
15	Female	77.2	26.6	6.4	Aseptic loosening	Revision TKA

*Summary of patients who underwent revision following UKA, including demographic characteristics and reasons for revision

## O32 Can non-operated leg length predict the recovery outcomes after total knee arthroplasty?

### Wing Ki Cheung, Yim Ling Amy Cheung, Michelle Hilda Luk, Ka Chun Thomas Leung, Chun Man Lawrence Lau, Chun Him Henry Fu

#### Division of Joint Replacement Surgery, Department of Orthopaedics and Traumatology, The University of Hong Kong, Hong Kong SAR, China

##### **Correspondence:** Wing Ki Cheung (suec01@hku.hk)

*Arthroplasty 2026*, **8(1):**O32

**Background**

Prior research has highlighted the significance of the non-operated leg in predicting functional outcomes following total knee arthroplasty (TKA). While it is established that limb lengthening occurs in the operated leg during TKA procedures, the impact on the non-operated leg remains unclear. Specifically, the question of whether the non-operated leg undergoes lengthening and the implications of this phenomenon on the likelihood of requiring a second-stage surgery are yet to be addressed. Understanding the potential lengthening effects on the non-operated leg is essential for optimizing surgical outcomes and informing decision-making regarding subsequent interventions. This retrospective study aims to analyze the limb length of the non-operated leg and its impact on recovery outcomes, as well as the likelihood of requiring a second-stage surgery following total knee arthroplasty.

**Methods**

Patients who underwent total knee arthroplasty as the first stage between June 2020 and December 2021 were recruited. Patients’ demographic (age, gender, and BMI) was collected. Whole-body radiographs were taken before and six months after the surgery. Measurements of femur, tibia, anatomical, and functional length were conducted. The differences between the operated and non-operated legs were calculated. The Knee Society Knee Score (KSKS) and Knee Society Functional Assessment (KSFA) were assessed at 1 year and 3 years post-surgery. Patients who proceeded to the second stage were also documented. The Wilcoxon Signed-Rank Test was utilized to compare the length of the operated and non-operated legs, as well as the preoperative and postoperative length. Spearman’s rank-order correlation was first employed to examine the relationship between limb length parameters and recovery outcomes, and then variables with less than or equal to 0.15 were included in a multivariable linear regression analysis.

**Results**

A total of 40 patients (34 female and 6 male) were included in the study. Significant differences were observed in the lengths of the femur, tibia, functional length, and anatomical length between the operated and non-operated legs both before and after surgery (all *P* > 0.05) (Table 1). All parameters were longer on the non-operated sides pre- and post-operatively, except for the functional length post-operatively. The tibia and anatomical length showed significant decreases, while the functional length significantly increased after surgery (all *P* > 0.05) (Table 2). A smaller difference in femur length between the operated and non-operated legs preoperatively (*t* = − 2.694, *P* = 0.011), being female (*t* = 3.398, *P* = 0.002), and younger age (*t* = − 2.291, *P* = 0.029) were associated with a better KSFA score at 3 years post-surgery (*F*(9,30) = 3.67, *P* = 0.003, *R*^2^ = 0.524) (Table 3). No significant multivariable regression model was found for KSKS at 1 and 3 years, and KSFA at 1 year after surgery, leading to the initiation of the second stage of surgery (Tables 1–3).

**Conclusions**

Notably, differences in femur length between the operated and non-operated legs prior to surgery, in conjunction with age and gender, were predictive of functional outcomes three years post-surgery. This suggests that clinicians should take into account femur length discrepancies when predicting post-surgical outcomes.

**Table 1 (Abstract O32).** Leg length measurements of patients

Length (mm)	Preoperative			Post-operative		
	**Operated side**	**Non-Operated side**	Differences*	**Operated side**	**Non-Operated side**	Differences*
Femur	375.7 ± 18	377.5 ± 17.2	− 1.8 ± 4.9	374 ± 18.6	375.7 ± 21.8	− 1.6 ± 15.7
Tibia	315.5 ± 14.5	318.7 ± 15.9	− 3.3 ± 4.8	310.5 ± 14.8	326.9 ± 60.1	− 16.4 ± 56.7
Functional	688.8 ± 32.8	694.9 ± 33.4	− 6.1 ± 7.3	698.2 ± 31.6	686.2 ± 66.6	12.1 ± 60
Anatomical	691.1 ± 31	696.2 ± 31.9	− 5.1 ± 6.1	684.5 ± 32	702.6 ± 66	− 18.1 ± 57.9

*Differences = Operated leg − non-operated leg

**Table 2 (Abstract O32).** Differences in leg length measurements of patients

Length (mm)	Differences between operated side and non-operated side		Differences between preoperative and post-operative	
	**At preoperative**	**At post-operative**	**At operated side**	**At non-operated side**
Femur	0.024*	0.004*	0.061	0.486
Tibia	< 0.001*	< 0.001*	< 0.001*	0.36
Functional	< 0.001*	0.036*	< 0.001*	0.844
Anatomical	< 0.001*	< 0.001*	< 0.001*	0.392

*Denotes statistical significance, *P* < 0.05

**Table 3 (Abstract O32).** Multivariable linear regression analysis of KSFA at 3 years after surgery

	Unstandardized Coefficients		Standardized Coefficients	t	Sig	95.0% Confidence Interval for B	
	B	Std. Error	Beta			Lower Bound	Upper Bound
(Constant)	− 105.913	71.232		− 1.487	0.147	− 251.388	39.562
Pre Functional length of Oper	0.105	0.618	0.188	0.17	0.867	− 1.158	1.367
Pre Anatomical length of Oper	1.108	0.826	1.878	1.342	0.19	− 0.578	2.794
Pre Femur length of NonO	− 1.412	0.897	− 1.329	− 1.574	0.126	− 3.243	0.42
Pre Tibia length of NonO	− 0.355	0.785	− 0.309	− 0.452	0.654	− 1.958	1.248
Pre Functional length of NonO	0.013	0.548	0.024	0.024	0.981	− 1.107	1.133
Pre Difference in Femur length between Oper and NonO	− 1.804	0.67	− 0.48	− 2.694	0.011*	− 3.172	− 0.436
Sex	24.12	7.098	0.477	3.398	0.002*	9.624	38.615
Age	− 1.005	0.439	− 0.317	− 2.291	0.029*	− 1.901	− 0.109
Pre KSFA	0.02	0.243	0.013	0.083	0.934	− 0.476	0.516

*Pre* preoperative, *Oper* operated leg, *NonO* non-operated leg, *KSFA* Knee Society Functional Assessment

*Denotes statistical significance, *P* < 0.05

## O33 The efficacy of low-level laser on postoperative pain and range of motion after bilateral total knee arthroplasty: randomized clinical trial

### Thakrit Chompoosang, Naruecha Jirasirisuk, Patcharavit Ploynumpon

#### Department of Orthopedics, Rajavithi Hospital, Bangkok, Thailand

##### **Correspondence:** Naruecha Jirasirisuk (N.jirasirisuk@gmail.com)

*Arthroplasty 2026*, **8(1):**O33

**Background**

Total knee arthroplasty (TKA) is a surgical procedure that can significantly improve the quality of life in patients with OA of the knee. Nevertheless, moderate to severe pain following TKA can affect physical rehabilitation and patient satisfaction. The medications can lead to side effects such as gastrointestinal ulcers, renal failure, or drug overdose, especially in the elderly. Low-level laser therapy (LLLT) is a non-invasive treatment that helps reduce pain and tissue swelling. Thus, in this study, we investigated the efficacy of low-level laser therapy (LLLT) on pain and range of motion after bilateral primary total knee arthroplasty (TKA).

**Methods**

The prospective randomized clinical trial, single-center, single-surgeon, was performed on bilateral primary TKA via mid-vastus approach with 2 groups of 16 patients. The LLLT (810 nm, 500 mW) group received LLLT postoperatively (Fig. 1), and the control group received laser in turn-off mode for the same length of time as the LLLT group. The visual analog scale pain scoring and range of motion (ROM) were recorded preoperatively and postoperatively on day 2, day 3, and month 3. Furthermore, postoperative complications due to LLLT and opioid consumption on days 2 and 3 were also documented.

**Results**

The preoperative demographic data, visual analog scale pain scoring, and range of motion were similar between groups. Compared with the control group, the LLLT group had significantly less pain on postoperative day 2, day 3 and month 3 (6.00 ± 1.46 vs 7.56 ± 1.55, 4.88 ± 1.31 vs 6.31 ± 1.58 and 2.69 ± 1.35 vs 4.13 ± 1.50, *P* value = 0.006, 0.009 and 0.008) and greater ROM on postoperative day 2 and day 3 (80.63 ± 20.16 vs 62.50 ± 21.76 and 94.69 ± 16.88 vs 73.75 ± 20.37, *P* value = 0.021 and 0.004) however, did not differ on month 3 (112.19 ± 13.29 vs 101.56 ± 16.71, *P* value = 0.056) (Table 1). In addition, the amount of opioid consumption in the LLLT group was significantly lower than that of the control group (*P* value < 0.001), and there were no complications due to LLLT.

**Conclusions**

According to this study, low-level laser therapy is effective in reducing pain, improving range of motion, and decreasing opioid consumption, and there are no complications from LLLT in acute postoperative bilateral total knee arthroplasty.

**Fig. 1 (Abstract O33).**
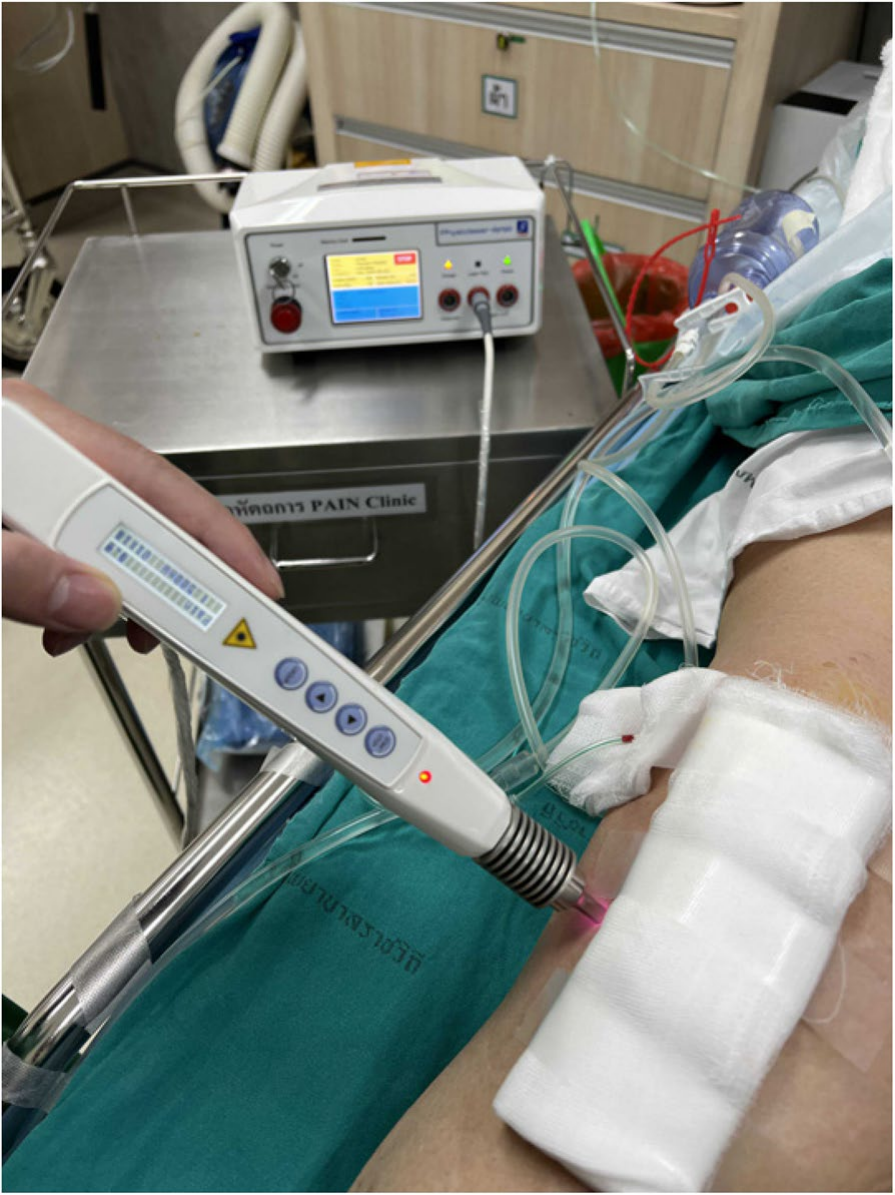
Applying a laser to the knee

**Table 1 (Abstract O33).** Comparison of outcomes of postoperative VAS pain scoring and ROM

Variables	Laser	Control	*P* value
VAS pain			
48 h	6.00 ± 1.46	7.56 ± 1.55	0.006*
72 h	4.88 ± 1.31	6.31 ± 1.58	0.009*
3 months	2.69 ± 1.35	4.13 ± 1.50	0.008*
ROM			
48 h	80.63 ± 20.16	62.50 ± 21.76	0.021*
72 h	94.69 ± 16.88	73.75 ± 20.37	0.004*
3 months	112.19 ± 13.29	101.56 ± 16.71	0.056

## O34 Comparison of functional implant position and clinical outcome between robotic-assisted THA vs conventional THA

### Thakrit Chompoosang, Danupol Sriruk, Patcharavit Ploynumpon

#### Department of Orthopedics, Rajavithi Hospital, Bangkok, Thailand

##### **Correspondence:** Danupol Sriruk (kengsoldic@gmail.com)

*Arthroplasty 2026*, **8(1):**O34

**Background**

The optimal orientation of the acetabular component is a key factor in successful THA. Component malposition in THA has been associated with an increased risk of dislocation. The use of robotic systems may improve the accuracy of component positioning. Therefore, the researchers aimed to compare the functional implant position and postoperative clinical outcomes between robotic-THA and conventional-THA.

**Methods**

A retrospective review was conducted on data from 128 consecutive patients who underwent THA, including 65 robotic-THA using the MAKO Stryker® system and 63 conventional-THA. Both groups used the Accolade II cup and Trident II stem, performed by a single surgeon using a posterior approach between 2022 and 2024. Preoperative CT scans were obtained for both groups to plan component placement before surgery. We measured the cup inclination, cup anteversion, stem anteversion, and combined anteversion from postoperative CT scans by two independent researchers with assessments based on the Lewinnek safe zone. Operative time, blood loss, complications, and the Forgotten Joint Score-12 (FJS-12) were collected and analyzed.

**Results**

Cup inclination showed a significantly better result in the robotic-THA (*P* < 0.001) (Fig. 1), but no significant difference in cup anteversion, stem anteversion, and combined anteversion (*P* = 0.41, 0.37, 0.72) between both groups. Operative time was significantly longer in the robotic-THA group (*P* < 0.001), and postoperative blood loss was also significantly higher in the robotic-THA group (*P* < 0.001). No complications were found in either group. No significant difference in the FJS-12 (*P* = 0.72) (Table 1). There were no significant differences in age, gender, side, or diagnosis between groups.

**Conclusions**

The results showed that robotic-THA was superior to conventional-THA in the placement of cup inclination, which is an important parameter that plays a significant role in the long-term success of THA. However, robotic-THA had a longer operative time and higher postoperative blood loss.

**Fig. 1 (Abstract O34).**
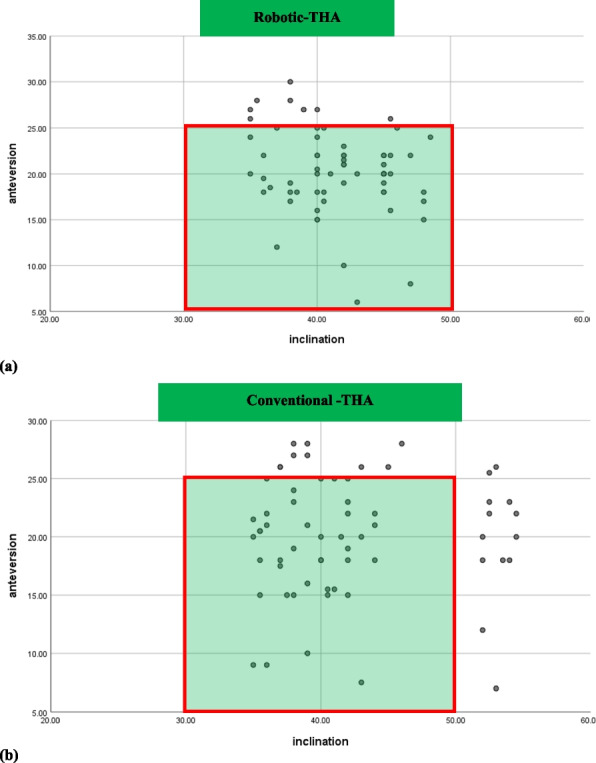
Scatterplots of the postoperative cup position **a** Robotic-THA and **b** Conventional-THA in the safe zones of Lewinnek, showed a significant outlier of inclination in the conventional-THA group compared with robotic-THA

**Table 1 (Abstract O34).** Post-operative component position and outcome between the two groups

Variable	Robotic THA	Conventional THA	*P*-value
Component position within Lewinnek’s zone *n* (%)			
Cup Inclination	65(100)	50(79.4)	< 0.001*
Cup Anteversion	57(87.7)	52(82.5)	0.41
Stem Anteversion	48(73.8)	42(66.7)	0.37
Combined Anteversion	62 (95.4)	59 (93.7)	0.72
Operative time (min)	96.9	71.9	< 0.001*
Blood loss (mL)	294.6	230.1	< 0.001*
FJS-12 score (mark)	75.7	75.9	0.72

## O35 Biomechanical and finite-element analysis of cement-screw technique for tibial bone defect in total knee arthroplasty

### Houyi Sun^1,2^, Dehua Liu^1,2^, Peilai Liu^1,2^

#### ^1^Department of Orthopaedics, Qilu Hospital of Shandong University, Jinan, China; ^2^Cheeloo College of Medicine, Shandong University, Jinan, China

##### **Correspondence:** Houyi Sun (15051514605@163.com)

*Arthroplasty 2026*, **8(1):**O35

The full article of this study has been published online. Please refer to the full text at: 10.1016/j.arth.2025.08.004.

## O36 A pilot study on the effect of type I diabetes mellitus on steroid-induced osteonecrosis of the femoral head in mice

### Gao Xu

#### HongHui Hospital, Xi’an Jiaotong University, Xi’an, China

##### **Correspondence:** Gao Xu (gaoxu3120315461@163.com)

*Arthroplasty 2026*, **8(1):**O36

**Background**

Steroid-induced osteonecrosis of the femoral head (SONFH), the most common type of non-traumatic osteonecrosis of the femoral head, has a high disability rate and a serious social and economic burden. Its risk factors are numerous, and its pathogenesis is not clear now; researchers believe that it may be the result of a combination of various factors. Among them, diabetes mellitus is one of the risk factors for osteonecrosis of the femoral head. Currently, most of the studies on the relationship between the two focus on clinical epidemiology. There is no basic research to explore the relationship between them or the relevant mechanism.

**Purpose**

This study aims to clarify the effect of type I diabetes(T1DM) on SONFH in mice through animal experiments and in vitro cell experiments, and to preliminarily explore the possible mechanism.

**Methods**

Sixty Balb/c male mice, about 25 g, 6 weeks old, were randomly divided into the DM-condition group and the non-DM condition group. STZ was used to induce the T1DM model, and the general condition, body weight, food intake, and blood glucose of the mice were regularly monitored. When random blood glucose ≥ 16.7 mmol/L, T1DM modeling was considered to be successfully established; then the non-DM condition group was randomly divided into the NOR group and the SONFH group; the DM condition group was randomly divided into the DM group and the DM + SONFH group. MPS was used to induce the SONFH model. At the 10th week, the mice were sacrificed.

Firstly, microCT, HE, and Masson staining results were compared to evaluate the effect of T1DM on SONFH. Then, RUNX2 immunohistochemical staining and TRAP staining were used to explore the possible mechanism. Finally, high-dose dexamethasone and high-glucose medium (HG) were used to intervene in bone marrow mesenchymal stem cells (BMSCs), respectively. ALP staining was used to explore the effects of the two on osteogenic differentiation of BMSCs, and to verify this with animal experiments.

**Results**

We successfully induced T1DM and SONFH mouse models. Then, by comparing the results of microCT, HE and Masson staining, we found that the degree of necrosis in DM + SONFH group was more severe than that in SONFH group: bone mass decreased significantly, bone trabeculae became thinner and more disordered, empty bone lacunae increased significantly, fat infiltration in the bone marrow cavity increased, and bone mineralization and maturation were significantly impaired. In addition, when exploring the mechanism, we found that the RUNX2 positive expression area in the DM + SONFH group was significantly reduced compared with the SONFH group, and the number of osteoclasts was significantly increased. Finally, in vitro cell experiment, HG + DEX significantly weakened bone differentiation compared with the DEX group, consistent with the animal experiment.

**Conclusions**

T1DM can affect SONFH, further inhibit osteogenic differentiation, and promote osteoclast activation, aggravating the progression of SONFH.

## O37 Arthroscopic dual decompression for ONFH collapse prevention

### Qichun Song^1^, Guangyang Zhang^1^, Yan Zhao^1^, Dazhi Wang^1^, Jiajun Jiang^1^, Xu Wang^1^, Donglong Shang^2^, Jianlong Ni^1^, Zhibin Shi^1^

#### ^1^The Second Affiliated Hospital of Xi’an Jiaotong University, Xi’an, China; ^2^Xi’an First Hospital, Xi’an, China

##### **Correspondence:** Zhibin Shi (zhibin_shi@126.com)

*Arthroplasty 2026*, **8(1):**O37

The full article of this study has been published online. Please refer to the full text at: 10.1186/s13018-022-03477-8.

## O38 Clinical outcomes of periprosthetic joint infections after unicompartmental knee arthroplasty: under the “RPAI” protocol

### Tao Zhang, Xianli Hu, Mo Chen, Zhen Pang, Haibo Sun, Rongwei Zhang, Rui Zhang, Abasi Maimaitiabula, Yao Luo, Chen Zhu

#### Department of Orthopedics, The First Affiliated Hospital of USTC, Division of Life Sciences and Medicine, University of Science and Technology of China, Hefei, China

##### **Correspondence:** Yao Luo (luoyao1223@163.com); Chen Zhu (zhuchena@ustc.edu.cn)

*Arthroplasty 2026*, **8(1):**O38

**Background**

Periprosthetic joint infection (PJI) following unicompartmental knee arthroplasty (UKA) presents unique diagnostic and therapeutic challenges due to concurrent PJI and septic arthritis. This study evaluates the efficacy of the novel RPAI protocol (Retentive cartilage radical debridement, Povidone-iodine flushing, Aspiration, Intra-articular antibiotics) in managing UKA-PJI, comparing debridement, antibiotics, and implant retention (DAIR) with one-stage exchange (OSE).

**Methods**

A single-center retrospective cohort of 38 UKA-PJI patients (DAIR: *n* = 21; OSE: *n* = 17) treated between 2016 and 2023 was analyzed. Inclusion adhered to MSIS/ICM criteria. The RPAI protocol involved staged debridement, 0.3% povidone-iodine irrigation, pulsed lavage, and intra-articular antibiotics. Outcomes included infection-free survival (primary endpoint), Hospital for Special Surgery (HSS) scores, and failure rates (Fig. 1).

**Results**

DAIR achieved 95.46% infection control and 90.91% all-cause revision-free survival at 2 years, surpassing historical benchmarks (*P* = 0.02 vs. OSE). OSE demonstrated 88.24% infection control. Metagenomic next-generation sequencing (mNGS) reduced culture-negative PJI rates from 34.21% (conventional culture) to 5.26% (*P* < 0.001), enabling targeted therapy. Synovial biomarkers (median CRP: 29.65 mg/L; WBC: 10,742 cells/μL; PMN%: 86%) exceeded TKA-PJI thresholds. Functional recovery (HSS scores) was comparable between groups, but DAIR showed superior early improvement (3-month HSS: 82.3 ± 6.1 vs. OSE: 75.8 ± 7.4, *P* = 0.02). Chronic infections exhibited higher failure rates (HR = 3.12, 95% CI: 1.08–9.01, *P* = 0.03) (Table 1).

**Conclusions**

The RPAI protocol enhances UKA-PJI management through biofilm reduction, sustained antibiotic delivery, and cartilage preservation.DAIR with mNGS-guided therapy achieves high infection control even in chronic cases, while OSE remains viable for extensive bone loss.

**Fig. 1 (Abstract O38).**
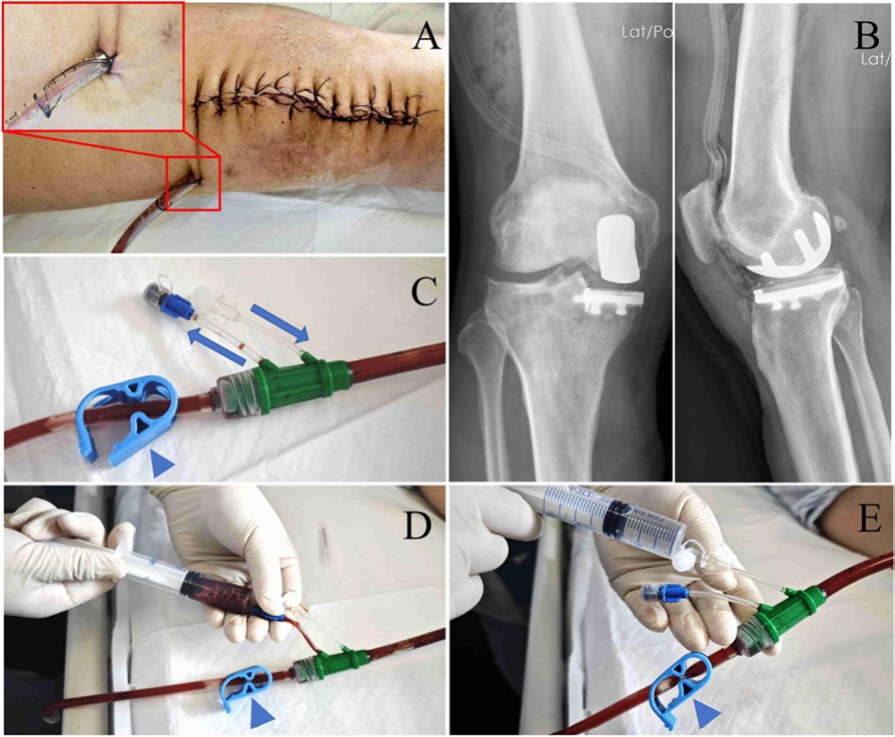
**A** Intraoperatively, a double-lumen catheter was inserted into the knee joint cavity via a suprapatellar bursa approach, entering 2–3 cm superolateral to the patella through the quadriceps tendon. The proximal end was secured to the skin, while the distal end was connected to a drainage bag for fluid collection. **B** The catheter was routed superiorly around the patella and positioned at the lowest point of the prosthesis. **C** During routine drainage, the clamp was opened. The distal port (blue cap) facilitated aspiration of intra-articular effusion, while the proximal port (white cap) was designated for intra-articular antibiotic infusion. **D** For aspiration: The clamp was closed, and the distal port (blue cap) was opened to aspirate intra-articular fluid. **E** For infusion: The clamp was closed, and the proximal port (white cap) was opened to slowly instill the sensitive antibiotic solution. Following infusion, the clamp remained closed for 6 h to maintain local high-concentration antibiotic therapy. The clamp was subsequently reopened to re-establish drainage

**Table 1 (Abstract O38).** Antibiotic treatment regimen

	**Culture Positive**	**Culture Negative**	
		**mNGS Positive**	**mNGS Negative**
Intravenous preoperative	Pathogen-sensitive antibiotics	Vancomycin 1 g	Vancomycin 1 g
Topical intraoperative	Vancomycin 0.5 g		Vancomycin 0.5 g
*Topical infusion postoperative	Anaerobic bacteria: Vancomycin 0.5 g Gram-positive organisms: Vancomycin 0.5 g Gram-negative organisms: Vancomycin 0.5 g in the morning and carbapenem 0.5 g in the afternoon		Vancomycin 0.5 g in the morning and carbapenem 0.5 g in the afternoon
Intravenous postoperative	Pathogen-sensitive antibiotics for 14 d	Vancomycin 1 g every 12 h for 14 d	
Oral postoperative	Quinolones and Rifampicin as an oral switch therapy was adopted for at least 6w until CRP and ESR returned to normal levels		

*CRP* C-reactive protein, *ESR* erythrocyte sedimentation rate

*Topical infusion antibiotic postoperative for 5–7 days after DAIR and for 14 days after one-stage exchange surgery

## O39 Clinical outcomes of functional alignment in robotic-assisted TKA for knee osteoarthritis with varus deformity

### Dasheng Lin, Dongmin Xu, Wentao Lin, Eryou Feng

#### Department of Orthopedic Surgery, Fujian Medical University Union Hospital, Fuzhou, China

##### **Correspondence:** Dasheng Lin (linds@xmu.edu.cn)

*Arthroplasty 2026*, **8(1):**O39

**Background**

To analyze the early efficacy of functional alignment (FA) technology in robotic-assisted total knee arthroplasty (RA-TKA) for osteoarthritis with varus deformity, and to provide a theoretical basis for selecting alignment strategies in TKA.

**Methods**

From January 2024 to December 2024, 40 patients (40 knees) diagnosed with osteoarthritis and varus deformity underwent FA-RA-TKA. The cohort included 12 males and 28 females, aged 62–85 years (mean 69.3 years). Surgical parameters (operative time, blood loss) and clinical outcomes were recorded, including preoperative and postoperative hip-knee-ankle angle (HKA), lateral distal femoral angle (LDFA), medial proximal tibial angle (MPTA), postoperative femoral component external rotation angle, range of motion (ROM), Visual Analog Scale (VAS) pain score, Knee Society Score (KSS), radiographic findings, and complications.

**Results**

All 40 patients completed follow-up (6–18 months, mean 11.5 months). Mean operative time was 105 min with 180 mL blood loss; incisions healed primarily. Significant improvements were observed in alignment parameters: preoperative vs. postoperative HKA (10.3° ± 2.7° [7°–17°] vs. 3.9° ± 0.8° [2°–6°]), LDFA (3.7° ± 0.6° [2°–7°] vs. 2.3° ± 0.4° [1°–3°]), and MPTA (6.6° ± 0.9° [3°–9°] vs. 2.1° ± 0.5° [1°–3°]) (*P* < 0.05). Postoperative femoral component external rotation averaged 3.9° (3°–7°). ROM improved from 93° ± 12° preoperatively to 124° ± 9° (*P* < 0.01), VAS pain scores decreased from 6.2 ± 1.8 to 2.5 ± 0.4 (*P* < 0.01), and KSS increased from 48 ± 11 to 86 ± 9 (*P* < 0.01). Radiographs demonstrated optimal prosthesis positioning without radiolucent lines, periprosthetic fractures, loosening, subsidence, or other anomalies.

**Conclusions**

FA-RA-TKA for varus-deformed osteoarthritis not only restores physiological lower limb alignment and achieves flexion–extension balance but also preserves native soft tissue envelopes, facilitating rapid postoperative recovery. Short-term clinical outcomes are promising, though mid- to long-term efficacy requires further investigation.

## O40 Robot-assisted uniportal endoscopic core decompression for the osteonecrosis of femoral head: clinical application and early outcomes

### Yingxing Xu, Jinsong Liu, Zengrui Zhang, Wenqian Xu, Zhiguang Chen, Tixiong Xia

#### Department of Orthopaedics, First Affiliated Hospital of Kunming Medical University, Kunming, China

##### **Correspondence:** Yingxing Xu (13708776227@163.com)

*Arthroplasty 2026*, **8(1):**O40

**Background**

Enhancing the accuracy of core decompression (CD) and ensuring complete lesion debridement are pivotal to optimizing hip preservation surgery outcomes in the osteonecrosis of the femoral head (ONFH). This study was designed to evaluate the clinical validity and early outcomes of robotic-assisted uniportal endoscopic core decompression (RUECD) for ONFH.

**Methods**

A prospective case–control study was conducted, enrolling 40 eligible ONFH patients undergoing joint-preserving surgery at the Department of Orthopedics, First Affiliated Hospital of Kunming Medical University. These patients were randomized into RUECD (*n* = 20) and conventional core decompression (CD) (*n* = 20) groups. Comparative parameters included: 1) perioperative metrics (operative time, blood loss, fluoroscopy frequency); 2) clinical outcomes (Harris Hip Score [HHS], International Hip Outcome Tool-33 [iHOT-33]) at 1, 3, 6, 12 and 24 months postoperatively; 3) radiographic parameters (necrotic lesion volume, ARCO classification, Tönnis grade) at 6, 12, and 24 months; and 4) femoral head survival rates (endpoints: subchondral collapse or conversion to THA) (Figs. 1&2).

**Results**

No significant intergroup differences were observed in operative time, intraoperative blood loss, HHS, and iHOT-33 scores at 1 and 3 months, as well as ARCO and Tönnis progression between the two groups. However, the RUECD group demonstrated superior outcomes in:

1. Reduced fluoroscopy frequency (4.3 ± 0.9 vs 7.1 ± 1.1 exposures, *P* < 0.001)
2. Better HHS scores (80.3 ± 4.7 vs 76.5 ± 5.3 at 6 months, *P* = 0.02; 83.1 ± 4.7 vs 79.4 ± 5.1 at 12 months, *P* = 0.025; 84.6 ± 3.9 vs 79.6 ± 4.8 at 24 months, *P* = 0.001)
3. Higher iHOT-33 scores (59.4 ± 2.1 vs 57.0 ± 3.2 at 6 months, *P* = 0.008; 67.6 ± 3.6 vs 64.2 ± 3.9 at 12 months, *P* = 0.007; 73.8 ± 4.6 vs 69.0 ± 5.5 at 24 months, *P* = 0.005)
4. Greater necrosis ratio improvement at 24 months (28.7 ± 5.1% vs 32.2 ± 4.9%, *P* = 0.031)

While 24-month survival rates with collapse as endpoint showed significant differences (90% vs 65%, *P* = 0.055), RUECD demonstrated significantly higher survival of the femoral head when considering THA conversion (100% vs 75%, *P* = 0.018).

**Conclusions**

Robotic-assisted uniportal endoscopic core decompression provides more precise necrotic tissue debridement compared to conventional CD, yielding superior early-term functional outcomes, improved lesion containment, and enhanced femoral head preservation rates for ONFH.

**Fig. 1 (Abstract O40).**
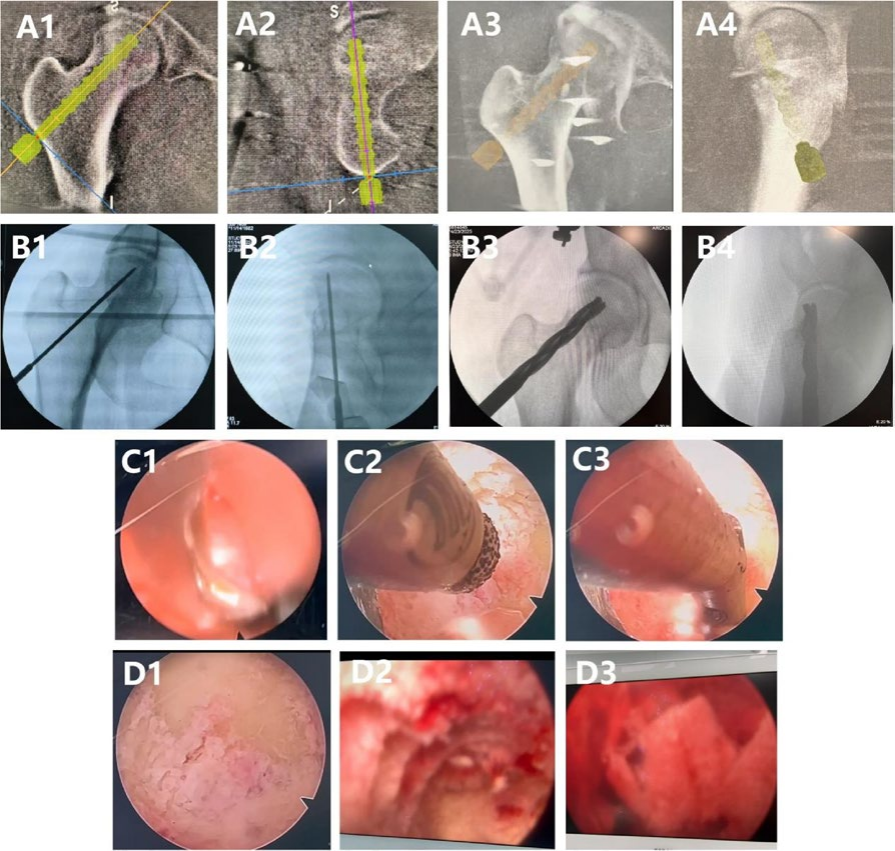
Robot-assisted uniportal endoscopic core decompression for the osteonecrosis of the femoral head. **A** The placement planning path and simulation graph were designed on the TiRobot workstation to ensure passing through the core necrotic area; **B** Core decompression based on the robot-assisted planning path; **C** Complete debridement of necrotic tissues under endoscopy; **D** Bone grafting under endoscopy after debridement until bleeding occurs

**Fig. 2 (Abstract O40).**
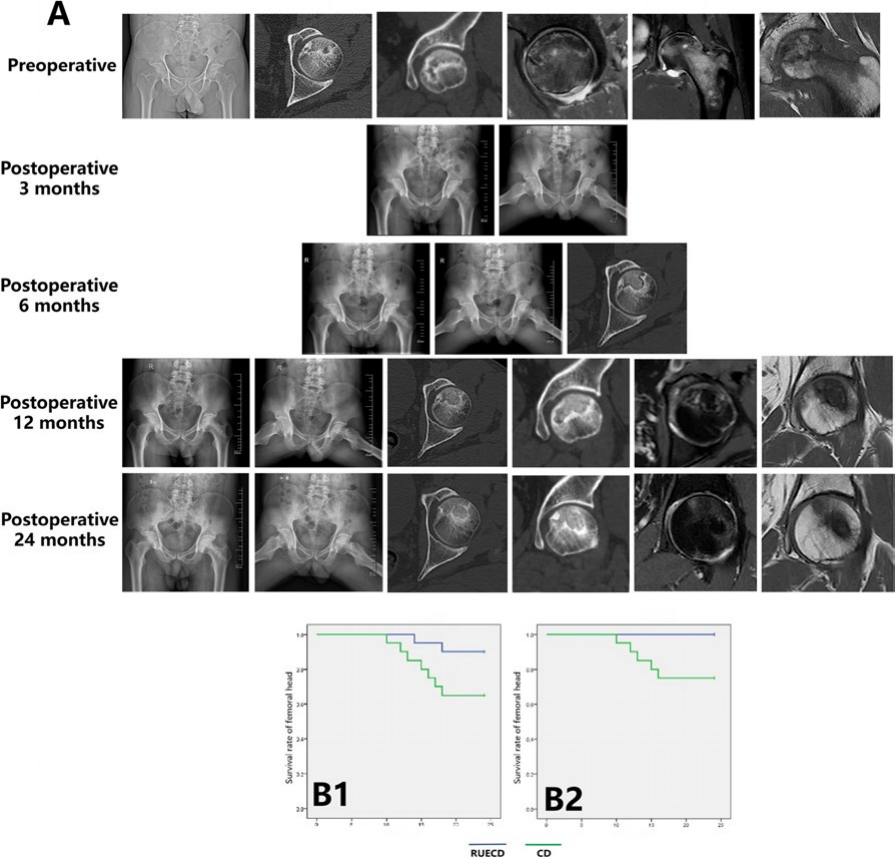
Early clinical outcomes. **A** Follow-up data of one case. **B** Comparison of survival rates of femoral head between two groups: (1) The survival curve of the femoral head with the collapse of the femoral head as the endpoint time; (2) The survival curve of the femoral head with THA as the endpoint time

## O41 Targeting Kdm5c to eliminate Piezo1-mediated mechanical memory of chondrocytes and restore its homeostasis

### Tianyou Kan^1,2^, Xuran Li^1^, Mengning Yan^1^, Zhifeng Yu^1^

#### ^1^Shanghai Key Laboratory of Orthopedic Implants, Department of Orthopedic Surgery, Shanghai Ninth People’s Hospital, Shanghai Jiao Tong University School of Medicine, Shanghai, China; ^2^ Department of Bone and Joint Surgery, Department of Orthopedics, Renji Hospital, School of Medicine, Shanghai Jiaotong University, Shanghai, China

##### **Correspondence:** Zhifeng Yu (zfyu@outlook.com)

*Arthroplasty 2026*, **8(1):**O41

**Background**

Mechanical load is a key factor in the occurrence of osteoarthritis (OA). Chondrocytes respond to mechanical load through multiple mechanical conduction pathways. However, how the nuclei of osteoarthritis chondrocytes respond to mechanical load remains unclear. The cell nucleus is mainly composed of histones and DNA. Mechanical loads may act on the cell nucleus and affect gene expression, leading to the occurrence of OA.

**Methods**

We found that abnormal mechanical loads not only caused cartilage damage but also led to significant nuclear aberrations in chondrocytes. This mechanical conduction pathway was mediated by Piezo1 (Fig. 1). The distorted nucleus leads to chromatin remodeling, and the H3K4me3 on the promoters of *Col2a1* and *Runx3* is downregulated, thereby causing OA (Fig. 2). Among them, the histone demethylase Kdm5c is mechanically activated by Piezo1. Further, we found that telmisartan has the effect of targeted inhibition of Kdm5c and delays cartilage injury caused by mechanical load in vivo and in vitro. As an antihypertensive drug, telmisartan is expected to be clinically translated for the prevention and treatment of osteoarthritis induced by mechanical loading (Fig. 3).

**Conclusions**

The mechanical load causes the down-regulation of H3K4me3 on the Col2a1 and Runx3 promoters of chondrocytes through Piezo1, leading to the occurrence of osteoarthritis. Telmesartan targets and inhibits Kdm5c to eliminate Piezo1-mediated mechanical memory of chondrocytes and restore cartilage homeostasis.

**Fig. 1 (Abstract O41).**
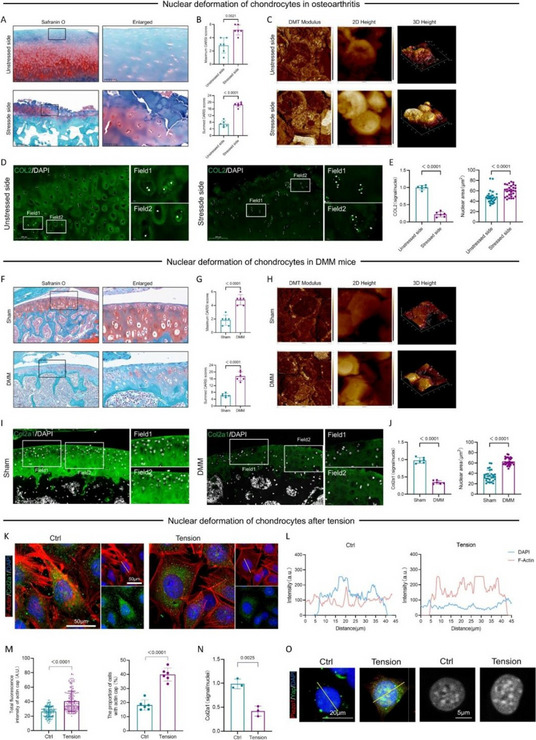
Mechanical loading leads to nuclear distortion of chondrocytes in osteoarthritis

**Fig. 2 (Abstract O41).**
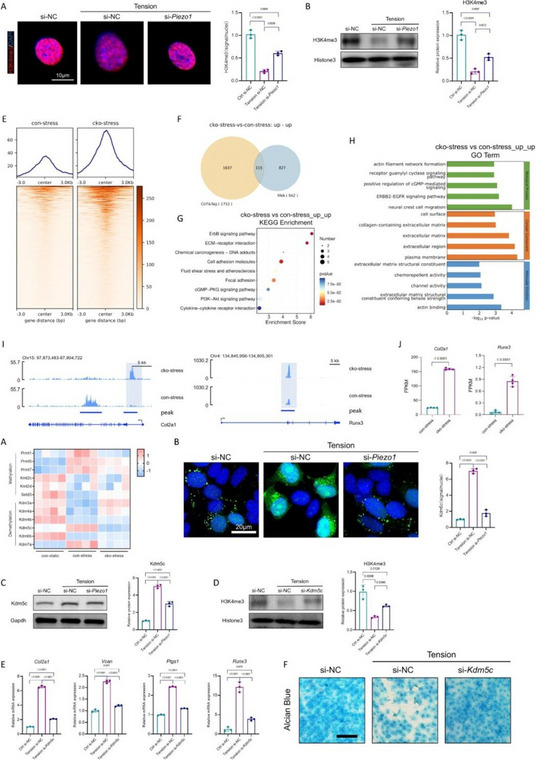
Kdm5c mediates the inhibitory effect of mechanical load on chondrocyte H3K4me3

**Fig. 3 (Abstract O41).**
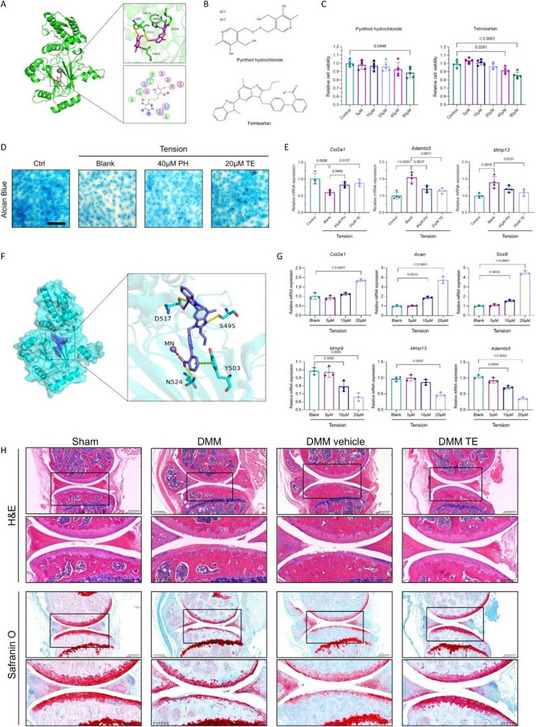
Telmisartan targets and inhibits Kdm5c to restore cartilage homeostasis

## O42 Development and validation of a spinopelvic parameter-based algorithm for optimal acetabular component positioning in total hip arthroplasty addressing hip-spine syndrome

### Yingxing Xu, Jinsong Liu, Xiaoyu Wan, Zengrui Zhang, Wenqian Xu, Zhiguang Chen, Tixiong Xia

#### Department of Orthopaedics, First Affiliated Hospital of Kunming Medical University, Kunming, China

##### **Correspondence:** Yingxing Xu (13708776227@163.com)

*Arthroplasty 2026*, **8(1):**O42

**Background**

Prosthetic dislocation is considered a common complication following total hip arthroplasty (THA), particularly prominent in patients with hip-spine syndrome. This study aims to investigate the risk factors influencing acetabular cup placement during THA in patients with spinal pathologies, as determined by spinopelvic parameters, thereby constructing a clinical predictive model for optimal cup positioning. Furthermore, it seeks to clarify the clinical value of this predictive model in guiding acetabular component placement during THA procedures.

**Methods**

The radiological parameters of patients underwent primary THA were collected in this study: spinal parameters [pelvic incidence (PI), pelvic tilt (PT), sacral slope (SS), lumbar lordosis angle (LL), sagittal vertical axis, thoracic kyphosis angle, cobb angle], pelvic parameters [cup inclination (CI), cup anteversion (CA), disparity in bilateral femoral offset], and spinopelvic parameters [absolute value of PI minus LL (|PI-LL|), change of sacral slope (ΔSS), combined sagittal index (CSI)]. The correlation between the above parameters and prosthesis dislocation after THA was assessed, and a clinical prediction model was constructed using the R language.

**Results**

These parameters, including ΔSS < 13° (OR = 4.015, *P* = 0.012), |PI-LL|> 10° (OR = 4.163, *P* = 0.012), CA > 23° (OR = 4.276, *P* = 0.008), standing CSI > 227° (OR = 4.103, *P* = 0.011) were independently associated with anterior prosthesis dislocation. Meanwhile, these parameters, including ΔSS < 13° (OR = 6.046,* P* = 0.003), |PI-LL|> 10° (OR = 3.717, *P* = 0.012), CA < 11° (OR = 8.738, *P* < 0.001), sitting CSI < 169° (OR = 4.468, *P* = 0.009) were independently associated with posterior prosthesis dislocation. The AUROC curves of the models were 0.847 (95%CI: 0.757–0.936) and 0.824 (95%CI: 0.729–0.918). Then, the Hosmer–Lemeshow test revealed that the *x*^2^ were 7.484 (*P* = 0.278) and 6.13 (*P* = 0.525), and the calibration plots for bootstrap resampling validation demonstrated satisfactory consistency. Moreover, a significant reduction in prosthesis dislocation and impingement was investigated when the acetabular cup was placed according to the clinical predictive model (Figs. 1&2).

**Conclusions**

These parameters, including ΔSS, |PI-LL|, CA, and CSI, should be fully considered when placing an acetabular cup during THA, and the clinical predictive model could reduce the dislocation and prosthetic impingement after THA in patients with hip-spine syndrome.

**Fig. 1 (Abstract O42).**
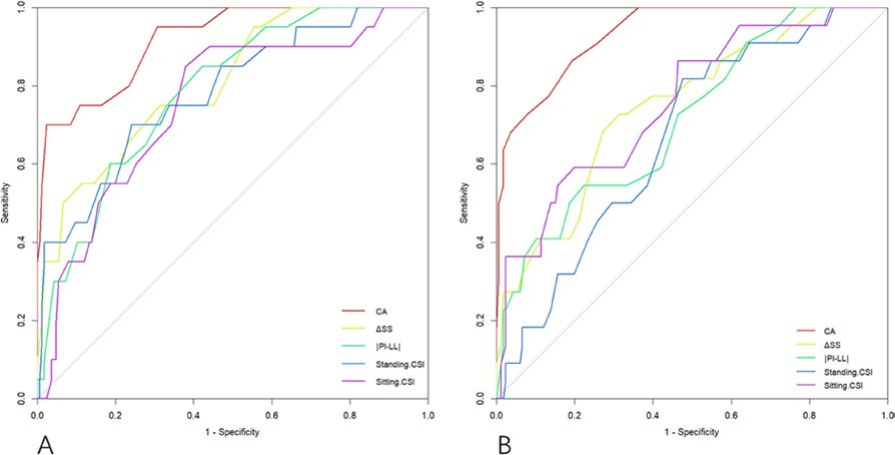
ROC curve of CA, ΔSS, |PI-LL|, standing CSI, and sitting CSI. **A** ROC of the anterior dislocation group and the non-dislocation group; **B** ROC of the posterior dislocation group and the non-dislocation group. CA = cup anteversion; ΔSS = Changes of sacral slope; |PI-LL|= absolute value of PI minus LL; standing CSI = standing combined sagittal index; sitting CSI = sitting combined sagittal index

**Fig. 2 (Abstract O42).**
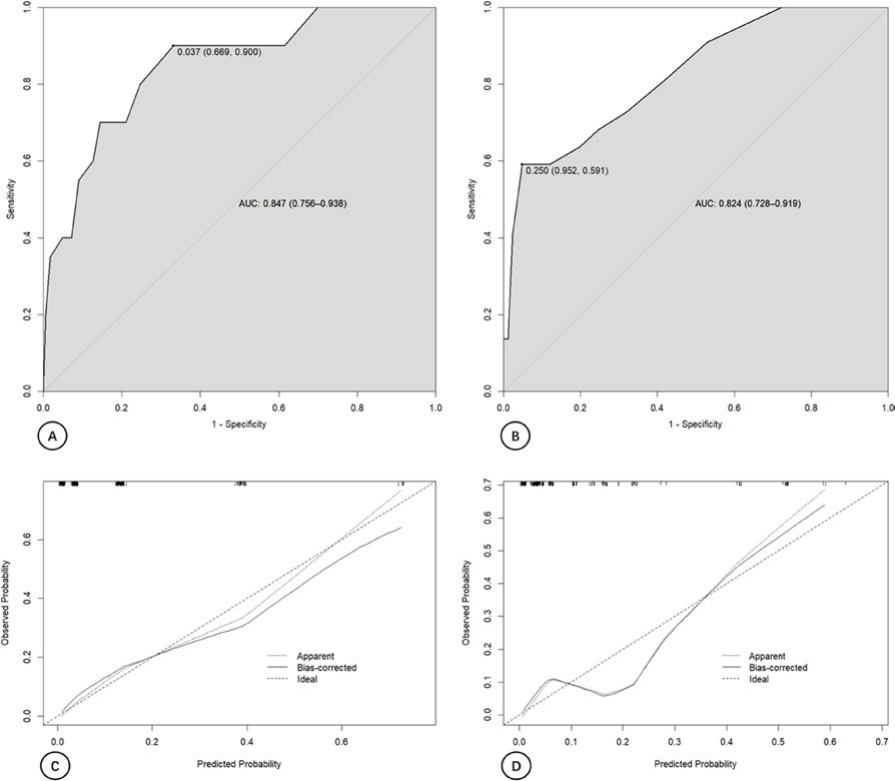
ROC and calibration curve for prediction models. ROC curves of clinical prediction models based on the data of anterior dislocation (**A**) and posterior dislocation (**B**). Calibration curve of the nomogram based on the data of anterior dislocation (**C**) and posterior dislocation (**D**)

## O43 Comparison of postoperative knee laxity and clinical outcomes between anatomically aligned and mechanically aligned robotic-arm assisted total knee arthroplasty in severe osteoarthritis patients

### Jae Hun Kim^1^, Byung Sun Choi^1^, Du Hyun Ro^1^, Myung Chul Lee^1,2^, Hyuk-Soo Han^1,2^

#### ^1^Department of Orthopaedic Surgery, Seoul National University Hospital, 101 Daehak-Ro, Jongno-Gu, Seoul, Republic of Korea; ^2^Department of Orthopaedic Surgery, Seoul National University College of Medicine, Seoul, Republic of Korea

##### **Correspondence:** Hyuk-Soo Han (oshawks7@snu.ac.kr)

*Arthroplasty 2026*, **8(1):**O43

**Background**

Mechanically aligned total knee arthroplasty (TKA) has been the gold standard of total knee arthroplasty for decades. However, only a few people have a neutral mechanical alignment of the knee before TKA. Recently, with the assistance of robotic systems, anatomically aligned TKA has been gaining attention. However, to date, there are few studies evaluating the medio-lateral gap laxity based on different alignments and their clinical outcomes. Therefore, the purpose of this study was to compare knee laxity and clinical outcomes between anatomically aligned TKA and mechanically aligned TKA.

**Methods**

We retrospectively reviewed 157 patients who were diagnosed with severe osteoarthritis and underwent robotic-arm-assisted TKA (MAKO®, Stryker, Michigan, USA) from October 2021 to May 2022. We excluded patients as follows: eight patients due to insufficient data, seven patients with rheumatoid arthritis, and two patients with post-traumatic osteoarthritis. We finally included 140 patients into 4 groups according to the type of alignment aimed in operation: mechanical alignment (*n* = 19), adjusted mechanical alignment (*n* = 40), adjusted anatomical alignment (*n* = 38), and anatomical alignment (*n* = 43). We defined medial and lateral laxity using intraoperative gap measure under varus, valgus stress, and no stress. Demographic, radiographic, clinical outcomes (pain numeric rating scale, Knee Society Scores, WOMAC, Forgotten Joint Score, Kujala score), and knee laxity were compared among the groups.

**Results**

There was no difference between groups in demographic, preoperative radiographic, and preoperative clinical outcomes. In groups with adjusted anatomical alignment and anatomical alignment, extension lateral laxity was significantly larger than extension medial laxity. The mechanical alignment group showed extension lateral laxity in a significantly smaller number of knees than the other groups, when extension lateral laxity was categorized by 2 mm. At three months after TKA, the Forgotten Joint Score was statistically significantly higher in the anatomically aligned TKA group. However, there were no statistical differences in other clinical scores at this time point. There were no statistical differences between any of the clinical scores at 1 year after TKA. However, no statistical correlation was found between the Forgotten Knee Joint Score at three months after TKA and the lateral laxity in extension and flexion.

**Conclusions**

With the application of robotic-arm-assisted total knee arthroplasty, a medio-lateral gap analysis was performed. Among the various alignments aimed at total knee arthroplasty, extension lateral laxity was larger than extension medial laxity in the groups with adjusted anatomical alignment and anatomical alignment. Additionally, at the three months after TKA, the Forgotten Joint Score was significantly higher in the anatomically aligned TKA group. Further studies involving a large number of participants and a longer follow-up period are needed.

## O44 Long-term outcome of two-stage revision surgery for prosthetic joint infection in a Malaysian tertiary hospital

### Muhindra Rao, Muhammad Azhar bin Abdullah, Ahmad Fauzey bin Kassim, Mohd Aizat Azfar bin Soldin

#### Orthopedic Department, Hospital Sultanah Bahiyah, Alor Setar, Kedah, Malaysia

##### **Correspondence:** Muhindra Rao (muhindra86@gmail.com)

*Arthroplasty 2026*, **8(1):**O44

**Background**

Prosthetic joint infection (PJI) is a severe complication following total joint arthroplasty. Two-stage revision remains the gold standard for managing chronic PJI, though reported eradication rates vary widely (54–100%). This study aimed to evaluate the long-term infection eradication rate after two-stage revision in a Malaysian tertiary hospital.

**Methods**

We retrospectively analyzed all two-stage revision procedures performed for chronic PJI from 2020 to 2025. Patient demographics, microbiological profiles, antibiotic regimens, and comorbidities were reviewed. The primary outcome was the eradication of infection, defined as the absence of reinfection requiring further surgery or long-term suppressive antibiotics. Predictors of reinfection were assessed using Firth’s logistic regression.

**Results**

A total of 26 patients underwent two-stage revision (mean age: 62.8 years; 54% female). Diabetes was the most common comorbidity (42.3%). Organisms isolated included Staphylococcus epidermidis, E. coli (ESBL), and Pseudomonas aeruginosa. IV vancomycin was the most frequently used antibiotic. Infection eradication was achieved in 24 of 26 patients (92.3%). Firth’s logistic regression revealed no statistically significant associations between reinfection and age (*P* = 0.81), sex (*P* = 0.65), BMI (*P* = 0.17), diabetes (*P* = 0.51), smoking (*P* = 0.83), alcohol (*P* = 0.75), or cardiovascular disease (*P* = 1.00) (Tables 1–4).

**Conclusions**

Two-stage revision surgery achieved a high eradication rate of 92.3% for infections in our cohort. No significant predictors of reinfection were identified, supporting the robustness of this surgical strategy in managing chronic PJI.

**Table 1 (Abstract O44).** Descriptive analysis of patients in the study

**Variable**		**Value**
Age	Age Range (years)	33–86
	Mean Age (years)	62.8
	Median Age (years)	68.5
Sex	Female (%)	53.8% (14/26)
	Male (%)	46.2% (12/26)
BMI	Mean BMI	29.0
	Median BMI	28.6
	BMI Range	23–39
ASA Group	ASA 2 (%)	65.4% (17/26)
	ASA 3 (%)	26.9% (7/26)

**Table 2 (Abstract O44).** Association between risk factors and reinfection

Risk Factor	Total patients with the risk factor	Reinfected	Total patients without risk factors	Reinfected
Diabetes	11	0	15	2
Cardiovascular	2	0	24	2
Smoking	4	0	22	2
Alcohol	3	0	23	2
Immunocompromised	0	0	21	2

**Table 3 (Abstract O44).** Firth’s logistic regression report

Predictor	Odds Ratio	*P*-value	95% CI Lower	95% CI Upper	Interpretation
Age	1.04	0.81	0.75	1.43	Not significant
Sex (Male)	7.41	0.65	0.0014	39,592	Not significant
BMI	1.45	0.17	0.85	2.48	Not significant
Diabetes	~ 0.001	0.51	~ 0.000001	960,000	Not significant
Smoking	~ 0.11	0.83	~ 0.00001	185,000,000	Not significant
Alcohol	~ 0.02	0.75	~ 0.00001	320,000,000	Not significant
Cardiovascular	≈1.00	1.00	0.00	152	No effect seen

**Table 4 (Abstract O44).** Organism isolated

Organism	Count
Staphylococcus species	15
No growth	3
Escherichia coli	3
Pseudomonas aeruginosa	2
Streptococcus agalactiae	1
Enterococcus faecalis	1
Other	1

## O45 Multidisciplinary team (MDT) impact on perioperative and functional outcomes in neck of femur fractures

### Muhindra Rao, Muhammad Azhar bin Abdullah, Ahmad Fauzey bin Kassim, Mohd Aizat Azfar bin Soldin

#### Orthopedic Department, Hospital Sultanah Bahiyah, Alor Setar, Kedah, Malaysia

##### **Correspondence:** Muhindra Rao (muhindra86@gmail.com)

*Arthroplasty 2026*, **8(1):**O45

**Background**

Neck of femur (NOF) fractures are associated with significant perioperative morbidity, mortality, and functional decline, particularly in geriatric populations. Integration of a multidisciplinary team (MDT) approach prior to surgical intervention has been proposed to mitigate these risks through comprehensive perioperative optimization. Our centre embarked on setting up a multidisciplinary team (MDT) to manage patients with neck of femur fracture prior to surgery. Hence, this study is to compare patients who had pre-operative MDT involvement and those who did not in terms of the surgical timing, perioperative outcomes, and functional recovery in neck of femur fracture patients.

**Methods**

A retrospective comparative cohort analysis was conducted on 54 patients admitted with NOF fractures between January 2023 and December 2024. This time frame was chosen as we had started MDT management in January 2024. Patients were stratified into MDT-managed (*n* = 27) (January 2024–December 2024) and non-MDT-managed (*n* = 27) (January 2023–December 2023) cohorts. MDT intervention included coordinated assessments by orthopaedic surgeons, geriatricians, anaesthetists, physiotherapists, and specialist nurses, focusing on preoperative medical stabilization and functional planning. Primary outcomes were time to surgery, length of hospital stay, Harris Hip Score, Forgotten Hip Joint Score, and incidence of inpatient complications. Statistical significance was determined using independent t-tests and chi-square tests, with *p*-values reported.

**Results**

Preoperative MDT involvement was associated with significantly expedited surgical intervention (mean 3.5 ± 1.0 days vs 11.5 ± 1.8 days; *P* < 0.001) and reduced hospital length of stay (7.5 ± 1.2 days vs 11.5 ± 1.5 days; *P* < 0.001). MDT patients demonstrated superior postoperative functional outcomes, reflected in higher mean Harris Hip Scores (80.5 ± 2.5 vs 71.0 ± 3.1; *P* < 0.001) and Forgotten Hip Joint Scores (93.6 ± 0.8 vs 91.3 ± 1.2; *P* = 0.002). Furthermore, the complication rate was significantly lower in the MDT cohort (3.7% vs 18.5%; *P* = 0.045) (Table 1).

**Conclusions**

Preoperative MDT engagement in NOF fracture management confers substantial benefits, including accelerated time to surgical fixation, shortened hospitalization, and enhanced postoperative functional recovery. These findings underscore the importance of adopting structured MDT pathways as a standard of care to optimize perioperative outcomes and functional independence in patients sustaining NOF fractures. We would recommend that all tertiary centres in Malaysia implement MDT management in patients with a neck of femur fracture.

**Table 1 (Abstract O45).** Clinical outcomes comparing MDT and non-MDT cohorts

Outcome	MDT Group (*n* = 27)	Non-MDT Group (*n* = 27)	*P*-value
Time to Surgery (days)	3.5 ± 1.0	11.5 ± 1.8	< 0.001
Length of Stay (days)	7.5 ± 1.2	11.5 ± 1.5	< 0.001
Harris Hip Score	80.5 ± 2.5	71.0 ± 3.1	< 0.001
Forgotten Hip Score	93.6 ± 0.8	91.3 ± 1.2	0.002
Complication Rate (%)	3.7%	18.5%	0.045

## O46 Effect of intra-articular infusion cocktail combined with tranexamic acid on postoperative pain and blood loss in patients with total knee arthroplasty

### Yuanzhen Cai, Yong Ding

#### Knee Preservation Department, HongHui Hospital, Xi’an Jiaotong University Health Science Center. Xi’an, China

##### **Correspondence:** Yuanzhen Cai (bonny17173@163.com)

*Arthroplasty 2026*, **8(1):**O46

**Background**

To observe the effect of an intra-articular infusion cocktail combined with tranexamic acid on pain and blood loss after total knee arthroplasty (TKA).

**Methods**

A total of 100 patients who underwent primary total knee arthroplasty for osteoarthritis from March to September 2024 were included, and all were divided into 2 groups of 50 patients each according to double-blind randomization. In the test group, 50 mL of a cocktail solution containing ropivacaine, compound betamethasone, epinephrine, and tranexamic acid was instilled into the joint cavity intraoperatively; in the control group, 50 mL of saline was instilled into the joint cavity; no drainage tube was placed in the joint cavity in either group. The visual analogue scale, postoperative maximum knee flexion, postoperative blood loss, hemoglobin decline, blood transfusion rate, and postoperative complications were observed and compared between the two groups (Fig. 1).

**Results**

Postoperative pain visual analog scores were lower in the test group at 6 h (4.0 ± 0.3), 12 h (5.3 ± 0.3), and 24 h (6.9 ± 0.5) than in the control group at 6 h (4.8 ± 0.5), 12 h (6.6 ± 0.4), and 24 h (7.9 ± 0.6), and the maximum knee flexion was greater in the test group at 24 h (59.13° ± 9.59°) and 48 h (82.48° ± 7.25°) than in the control group at 24 h (49.54° ± 10.32°), 48 h (72.88° ± 8.09°), and the total blood loss was less in the test group at 1 d (343.7 mL ± 73.2 mL), 3d (656.0 mL ± 271.2 mL) than in the control group at 1 d (657.3 mL ± 107.4 mL), 3 d (1012.0 mL ± 284.6 mL) postoperatively. The hemoglobin drop in the test group at 1 d (7.8 g/L ± 5.9 g/L), 3 d (14.3 g/L ± 14.7 g/L) was lower than that of the control group at 1 d (10.2 g/L ± 4.8 g/L), 3 d (18.7 g/L ± 12.6 g/L) postoperatively, and the difference was statistically significant (*P* < 0.05). There was no statistically significant difference in the visual analogue score of pain at 48,72 h postoperatively and the maximum knee flexion at 72 h postoperatively (*P* > 0.05). There was no statistically significant difference in the incidence of postoperative venous thrombosis in the lower limbs between the two groups, and no complications such as surgical incision infection or joint cavity hematoma occurred in either group (Tables 1–3).

**Conclusions**

The treatment of intra-articular infusion cocktail combined with tranexamic acid can effectively alleviate the early pain of patients after total knee arthroplasty and reduce the perioperative blood loss, which will not increase the risk of related complications.

**Fig. 1 (Abstract O46).**
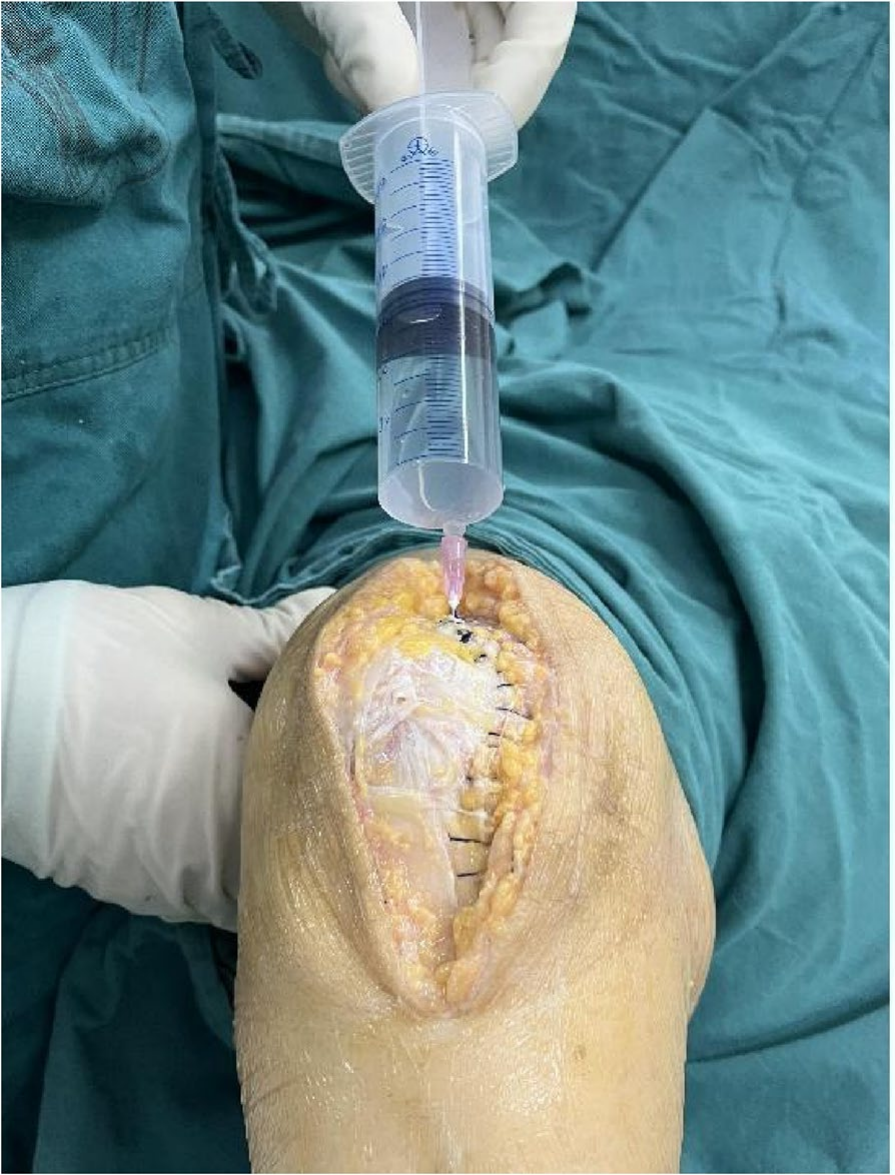
Intraoperative “cocktail” perfusion injection of TKA

**Table 1 (Abstract O46).** Comparison of visual analogy scores between the two groups at different time points after TKA

	**Experimental group**	**Control group**	***t***	***P***
6 h after surgery	4.0 ± 0.3	4.8 ± 0.5	7.72	< 0.001
12 h after surgery	5.3 ± 0.3	6.6 ± 0.4	11.78	< 0.001
24 h after surgery	6.9 ± 0.5	7.9 ± 0.6	2.58	0.018
48 h after surgery	4.2 ± 0.4	4.3 ± 0.3	0.53	0.593
72 h after surgery	3.6 ± 0.5	3.8 ± 0.4	0.54	0.592

**Table 2 (Abstract O46).** Maximum knee flexion at different time points after TKA in the two groups

	**Maximum knee flexion (x ± s°)**		
	**24 h after surgery**	**48 h after surgery**	**72 h after surgery**
Experimental group	59.13 ± 9.59	82.48 ± 7.25	93.6 ± 11.8
Control group	49.54 ± 10.32	72.88 ± 8.09	91.9 ± 10.5
*P*	0.00	0.04	> 0.99

**Table 3 (Abstract O46).** Blood loss, hemoglobin decrease, and blood transfusion rate of the two groups at 1 d and 3 d after TKA

	**Blood loss (mL)**		**Hemoglobin decrease (g/L)**		**Transfusion rate (n/%)**
	**24 h after surgery**	**72 h after surgery**	**24 h after surgery**	**72 h after surgery**	
Experimental group	343.7 ± 73.2	656.0 ± 271.2	7.8 ± 5.9	14.3 ± 14.7	3/6
Control group	657.3 ± 107.4	1012.0 ± 284.6	10.2 ± 4.8	18.7 ± 12.6	5/10
*t*	− 14.11	8.23	1.67	2.35	χ^2^ = 1.38
*P*	0.003	< 0.001	0.038	0.028	0.26

## O47 Clinical outcomes of platelet-rich plasma at varying concentrations for knee osteoarthritis: a prospective study

### Yuanzhen Cai, Yong Ding

#### Knee Preservation Department, HongHui Hospital, Xi’an Jiaotong University Health Science Center. Xi’an, China

##### **Correspondence:** Yuanzhen Cai (bonny17173@163.com)

*Arthroplasty 2026*, **8(1):**O47

**Background**

To investigate the specific clinical efficacy of platelet-rich plasma (PRP) therapy at different concentrations in the treatment of knee osteoarthritis (KOA).

**Methods**

A total of 150 patients with knee osteoarthritis admitted to the Knee Conservation Department of Xi’an Honghui Hospital affiliated to Xi’an Jiaotong University from January 2024 to December 2024 were selected and randomly divided into five groups: control group (sodium hyaluronate therapy), observation group I (PRP therapy at a concentration of 900 × 10^9^/L–1199 × 10^9^/L), observation group II (PRP therapy at a concentration of 1200 × 10^9^/L–1599 × 10^9^/L), observation group III (PRP therapy at a concentration of 1500 × 10^9^/L–1800 × 10^9^/L), and observation group IV (PRP therapy at a concentration of 1801 × 10^9^/L–2100 × 10^9^/L). Analysis was conducted on the post-treatment adverse reaction rate, VAS score, and IKDC score.

**Results**

The IKDC scores were 67.03 ± 2.46 in observation group I, 70.54 ± 6.48 in observation group II, 74.01 ± 7.38 in observation group III, and 65.98 ± 10.29 in observation group IV after 3 months of treatment; the VAS scores were 4.33 ± 1.42 in observation group I, 3.52 ± 1.41 in observation group II, 2.22 ± 1.03 in observation group III, and 3.16 ± 1.11 in observation group IV, with significant differences between groups (*P* < 0.05) (Tables 1&2).

**Conclusions**

In PRP therapy for knee osteoarthritis, if the concentration can be maintained between 1500 × 10^9^/L and 1800 × 10^9^/L, more significant clinical efficacy can be achieved. If the concentration is too low or too high, patients’ pain will increase, and knee joint function will be limited. Therefore, the plasma concentration in PRP therapy should be well controlled. This therapy is both effective and safe.

**Table 1 (Abstract O47).** Comparison of IKDC scores of patients before and after treatment

Group/Rating	IKDC scores			
	**Before treatment**	**3 months**	**6 months**	**9 months**
Control group (*n* = 30)	54.93 ± 6.79	64.39 ± 8.49	54.96 ± 5.84	56.94 ± 7.38
Observation Group I (*n* = 30)	55.29 ± 5.68	67.03 ± 2.46	58.59 ± 5.89	58.38 ± 5.75
Observation Group II (*n* = 30)	55.01 ± 5.97	70.54 ± 6.48	59.69 ± 6.03	57.32 ± 5.39
Observation Group III (*n* = 30)	54.28 ± 5.39	74.01 ± 7.38	66.43 ± 7.34	65.39 ± 7.86
Observation Group IV (*n* = 30)	55.01 ± 6.03	65.98 ± 10.29	58.37 ± 6.39	59.30 ± 6.49
*t*	0.306	4.583	4.448	4.392
*P*	> 0.05	< 0.05	< 0.05	< 0.05

**Table 2 (Abstract O47).** Comparison of VAS scores of patients before and after treatment

Group/Rating	VAS scores			
	**Before treatment**	**3 months**	**6 months**	**9 months**
Control group (*n* = 30)	6.22 ± 1.21	5.48 ± 1.25	5.72 ± 1.55	5.85 ± 1.35
Observation Group I (*n* = 30)	5.93 ± 1.11	4.33 ± 1.42	4.37 ± 1.32	4.82 ± 1.22
Observation Group II (*n* = 30)	6.13 ± 1.02	3.52 ± 1.41	4.29 ± 1.22	3.79 ± 1.01
Observation Group III (*n* = 30)	6.01 ± 1.03	2.22 ± 1.03	2.59 ± 1.03	3.42 ± 1.05
Observation Group IV (*n* = 30)	5.98 ± 1.31	3.16 ± 1.11	3.34 ± 1.23	3.88 ± 1.21
*t*	0.023	4.592	4.203	4.024
*P*	> 0.05	< 0.05	< 0.05	< 0.05

## O48 Imaging parameters for contralateral hip dysplasia in asymptomatic adults over 60 years old with femoral neck fractures

### Zhiqiang Chen^1,2^, Zhendong Zhang^2^, Hui Cheng^2^, Ziyin Xu^1,2^, Wei Chai^2^, Dianzhong Luo^2^, Hong Zhang^2^

#### ^1^School of Medicine, Nankai University, Tianjin, China; ^2^Senior Department of Orthopaedics, the Fourth Medical Center of PLA General Hospital, Beijing, China

##### **Correspondence:** Wei Chai (chaiweiguanjie@sina.com)

*Arthroplasty 2026*, **8(1):**O48

The full article of this study has been published online. Please refer to the full text at: 10.1111/os.70203.

## O49 Calibrated fluoroscopy in periacetabular osteotomy: an approach to optimize anterior coverage accuracy

### Hui Cheng

#### The Fourth Medical Center of PLA General Hospital, National Clinical Research Center for Ortho⁃ Paedics, Sports Medicine and Rehabilitation, Beijing, China

##### **Correspondence:** Hui Cheng (shenzhentie@163.com)

*Arthroplasty 2026*, **8(1):**O49

**Background**

Periacetabular osteotomy (PAO) is the gold-standard treatment for developmental dysplasia of the hip (DDH) in skeletally mature patients. However, achieving optimal acetabular correction remains challenging, particularly in assessing anterior coverage, as conventional uncalibrated fluoroscopy may not accurately represent the functional acetabular position due to variations in pelvic tilt between standing and operative positions.

**Purposes**

We asked: 1. What are the radiographic parameters (LCEA and ACEA) in PAO patients? 2. How does calibrated fluoroscopy compare to conventional techniques in achieving optimal correction? 3. What factors influence radiographic outcomes with calibrated fluoroscopy? 4. What is the interobserver reliability of this measurement technique?

**Methods**

We conducted a retrospective comparative study of 213 consecutive PAOs (212 patients) performed at our tertiary referral center between January 2018 and October 2020. After applying strict inclusion/exclusion criteria, we compared outcomes between two cohorts: a calibrated fluoroscopy group (121 hips) utilizing our novel technique of matching intraoperative imaging to preoperative standing radiographs, and a historical control group (92 hips) using conventional fluoroscopic guidance. Our calibrated fluoroscopy technique involved adjusting the C-arm position to match the obturator foramen with preoperative standing radiographs, thereby accounting for individual pelvic tilt variations. Both groups had comparable demographic characteristics, including age (median 29.0 vs 27.5 years), gender distribution (86.0% vs 79.3% female), and body mass index (22.1 ± 2.8 vs 22.1 ± 3.4 kg/m^2^). Radiographic parameters, specifically lateral center–edge angle (LCEA) and anterior center–edge angle (ACEA), were evaluated on standardized standing anteroposterior and false-profile radiographs obtained preoperatively and at a minimum 6-month follow-up when patients had achieved unassisted ambulation.

**Results**

Among 213 consecutive PAOs, preoperative analysis revealed a median LCEA of 6.3° in the calibrated group versus 3.3° in controls (*P* = 0.180), with median ACEA of 10.2° versus 7.9°, respectively (*P* = 0.572). The calibrated fluoroscopy group demonstrated significantly improved anterior coverage outcomes, with 90.9% achieving normal anterior coverage compared to 75.0% in controls (*P* = 0.004), and lower rates of excessive anterior correction (5.0% vs 18.5%). Both groups achieved comparable accuracy in lateral coverage correction (90.9% vs 90.2% within normal range, *P* = 0.921). Analysis revealed a strong positive correlation between preoperative LCEA and ACEA measurements (*r* = 0.695, *P* < 0.001), while preoperative borderline dysplasia and normal anterior coverage showed weak positive correlations with postoperative overcorrection in both parameters.

**Conclusion**

Calibrated fluoroscopy referencing preoperative standing radiographs significantly improves the accuracy of anterior coverage correction in PAO while maintaining reliable lateral correction. This practical technique accounts for individual variations in pelvic orientation during surgery and enhances radiographic outcomes.

## O50 HTO for varus knee osteoarthritis: equivalent efficacy of medial meniscus posterior root tear repair and non-repair

### Qichun Song^1^, Yan Zhao^1^, Guangyan Zhang^1^, Dazhi Wang^1^, Donglong Shang^2^, Jiajun Jiang^1^, Jianlong Ni^1^, Zhibin Shi^1^

#### ^1^The Second Affiliated Hospital of Xi’an Jiaotong University, Xi’an, China; ^2^Xi’an First Hospital, Xi’an, China

##### **Correspondence:** Zhibin Shi (zhibin_shi@126.com)

*Arthroplasty 2026*, **8(1):**O50

**Background**

Medial meniscus posterior root tear (MMPHRT) in varus knee osteoarthritis (OA) may cause medial meniscus extrusion (MME) and accelerate cartilage degeneration. Whether MMPHRT repair is necessary during open-wedge high tibial osteotomy (OWHTO) remains controversial. This study compared clinical outcomes between OWHTO with MMPHRT repair and OWHTO alone.

**Methods**

Sixty patients with knee OA and medial meniscus tears who underwent OWHTO from 2019 to 2021 were retrospectively analyzed. Thirty-five patients received OWHTO combined with total-inside repair of MMPHRT using the Fast Fix suture system (HTOR group), and 25 underwent OWHTO alone (HTO group), with a mean follow-up of 2 years. Outcomes included meniscus healing status (complete healing, partial healing, non-healing) via second-look arthroscopy, International Cartilage Repair Society (ICRS) grades of the medial femoral condyle (MFC) and medial tibial plateau (MTP), MME severity on MRI, weight-bearing radiograph parameters (medial joint space width, joint line convergence angle [JLCA], posterior tibial slope [PTS], Kellgren-Lawrence [KL] grading, hip-knee-ankle angle [HKA], weight-bearing line [WBL] ratio), and clinical scores (Knee Society Score [KSS], Western Ontario and McMaster Universities Osteoarthritis Index [WOMAC]).

**Results**

The HTOR group showed significantly higher partial/complete healing rates of MMPHRT than the HTO group (77% vs. 44%, *P* < 0.001). No significant differences were observed between groups in ICRS grades of MFC and MTP before or after surgery (Table 1). Postoperative MME (4.2 ± 1.8 mm vs. 4.1 ± 2.0 mm, *P* > 0.05), medial joint space width, JLCA, PTS, KL grading, and clinical scores (KSS, WOMAC) did not differ significantly between groups (Table 2).

**Conclusions**

While MMPHRT repair during OWHTO improves tear healing rates, it does not significantly affect imaging parameters or clinical outcomes compared with non-repair. Current evidence does not support routine MMPHRT repair during OWHTO for varus knee OA.

**Table 1 (Abstract O50).** Comparison of ICRS grading of femoral condyles and tibial plateau between the two groups before and after arthroscopic surgery

	**HTOR (*n* = 35)**	**HTO (*n* = 25)**	***P***
ICRS grading of the medial femoral condyle			
Before surgery (Grade0/1/2/3/4)	0/1/8/12/14	0/2/7/9/7	0.420
After surgery (Grade0/1/2/3/4)	0/6/9/11/9	0/4/6/9/6	0.477
ICRS grading of the tibial plateau			
Before surgery (Grade0/1/2/3/4)	0/0/4/9/12	0/1/11/12/14	0.426
After surgery (Grade0/1/2/3/4)	1/7/7/11/9	3/8/9/11/7	0.297

**Table 2 (Abstract O50).** Comparison of imaging results between the two groups before and after surgery

	**HTOR (*n* = 35)**	**HTO (*n* = 25)**	***P***	
Medial meniscus extrusion (mm)				
Before surgery	4.4 ± 1.8	4.3 ± 1.9	0.155	
After surgery	4.2 ± 1.8	4.1 ± 2.0	0.979	
Medial joint space (mm)				
Before surgery	3.5 ± 1.0	3.6 ± 1.3	0.129	
After surgery	3.8 ± 1.2	3.5 ± 1.7	0.678	
Joint line convergence angle (degrees)				
Before surgery	3.1 ± 1.2	3.0 ± 1.6	0.087	
After surgery	2.5 ± 1.1	2.3 ± 1.4	0.376	
Posterior tibial slope (degrees)				
Before surgery	8.9 ± 1.3	8.6 ± 1.9	0.467	
After surgery	8.8 ± 1.8	9.1 ± 1.7	0.534	
Weight-bearing line (WBL) ratio (%)				
Before surgery	21.2 ± 9.9	22.2 ± 12.8	0.876	
After surgery	63.1 ± 10.7	64.0 ± 11.7	0.236	
Hip-knee-ankle angle (degrees)				
Before surgery	Varus6.7 ± 2.2	Varus6.8 ± 2.6	0.841	
After surgery	Valgus 1.7 ± 1.2	Valgus 1.8 ± 1.2	0.445	
Kellgren-Lawrence (KL) grading				
Before surgery (1/2/3/4)	3/13/16/3	0/7/15/3	0.567	
After surgery (1/2/3/4)	5/16/12/2	2/11/10/2	0.335	
Before surgery	8.9 ± 1.3	8.6 ± 1.9		0.467
After surgery	8.8 ± 1.8	9.1 ± 1.7		0.534
Weight-bearing line (WBL) ratio (%)				
Before surgery	21.2 ± 9.9	22.2 ± 12.8		0.876
After surgery	63.1 ± 10.7	64.0 ± 11.7		0.236
Hip-knee-ankle angle (degrees)				
Before surgery	Varus6.7 ± 2.2	Varus6.8 ± 2.6		0.841
After surgery	Valgus 1.7 ± 1.2	Valgus 1.8 ± 1.2		0.445
Kellgren-Lawrence (KL) grading				
Before surgery (1/2/3/4)	3/13/16/3	0/7/15/3		0.567
After surgery (1/2/3/4)	5/16/12/2	2/11/10/2		0.3349

## O51 Long-term incidence of patellofemoral pain and its impact on patient satisfaction following total knee arthroplasty and Oxford unicompartmental knee arthroplasty

### Abasi Maimaitiabula, Mo Chen, Tao Zhang, Xianzuo Zhang, Chen Zhu

#### Department of Orthopedics, The First Affiliated Hospital of USTC, Division of Life Sciences and Medicine, University of Science and Technology of China, Hefei, China

##### **Correspondence:** Che Zhu (zhuchena@ustc.edu.cn); Xianzuo Zhang (zhangxianzuo@ustc.edu.cn)

*Arthroplasty 2026*, **8(1):**O51

**Background**

Over the past two decades, Oxford unicompartmental knee arthroplasty (UKA) has gained increasing popularity among patients and clinicians. However, unlike total knee arthroplasty (TKA), UKA alters the kinematic trajectory of the knee, which may increase patellofemoral pressure. This increased pressure can potentially lead to postoperative patellofemoral pain (AKP) and other complications, thus affecting patient functionality. This study retrospectively analyzed the follow-up data of 196 patients who underwent UKA or TKA at our institution.

**Methods**

This retrospective case–control study was approved by the institutional ethics committee. It included patients who underwent knee arthroplasty (UKA or TKA) between February 2018 and January 2020. A total of 179 patients who underwent UKA were matched with 179 patients who underwent TKA using propensity score matching (PSM). Clinical records were reviewed, and patellofemoral pain (AKP) occurrence was assessed as the primary outcome, comparing preoperative and postoperative results (Tables 1&2).

**Results**

For patients without preoperative AKP, the incidence of AKP within 12 months post-UKA was 21.21%, which was comparable to the 21.43% observed in the TKA group (*P* = 0.905). Among those who did not experience AKP relief post-surgery, 11.01% were in the TKA group, while 18.58% remained symptomatic in the UKA group (*P* = 0.113).

**Conclusion**

This study evaluates the long-term impact of UKA and TKA on postoperative AKP and patient satisfaction, offering valuable insights for clinical decision-making.

**Table 1 (Abstract O51).** Baseline data before and after PSM analysis

Characteristics	Before PSM			After PSM		
	**TKA**	**UKA**	***P-*value**	**TKA**	**UKA**	***P-*value**
Age (years)	65.15 ± 9.62	60.83 ± 8.32	< 0.01	61.13 ± 8.66	60.83 ± 8.32	0.7366
BMI (kg/m^2^)	31.08 ± 8.49	25.86 ± 8.49	< 0.01	25.76 ± 6.09	25.86 ± 8.49	0.8274
Gender						
Male	1685(24.85%)	36(20.11%)	0.1728	36(20.11%)	36(20.11%)	0.2711
Female	5095(75.15%)	143(79.89%)		143(79.89%)	143(79.89%)	

**Table 2 (Abstract O51).** Patients with AKP were diagnosed before and after surgery

		**Postoperative**	**TKA**	**UKA**
Preoperative	AKP	AKP	12(6.70%)	21(11.73%)
		no AKP	97(54.19%)	92(51.40%)
	no AKP	AKP	15(8.38%)	14(7.82%)
		no AKP	55(30.73%)	52(29.05%)

## O52 Clinical study on early outcomes of robot-assisted unicompartmental knee arthroplasty

### Bei Lin, Eryou Feng

#### Fujian Medical University Union Hospital, Fuzhou, China

##### **Correspondence:** Bei Lin (lb19961106@163.com)

*Arthroplasty 2026*, **8(1):**O52

**Object**

To compare the efficacy of robot-assisted (Naton) unicompartmental knee arthroplasty (UKA) versus conventional manual UKA in treating anteromedial osteoarthritis (AMOA) of the knee and evaluate the early clinical outcomes of robot-assisted UKA.

**Methods**

A retrospective analysis was conducted on 121 patients with AMOA who underwent UKA in our hospital’s Joint Surgery Department between November 2023 and October 2024. Patients were divided into two groups: robot-assisted UKA (73 cases) and conventional manual UKA (48 cases). Intraoperative metrics (surgery duration, blood loss), postoperative hospital stay, and complications were compared. Pain and functional outcomes were assessed using the Visual Analog Scale (VAS), Hospital for Special Surgery (HSS) Knee Score, and Forgotten Joint Score (FJS). Lower limb alignment was evaluated via medial proximal tibial angle (MPTA) and mechanical axis deviation (MAD). Prosthesis placement accuracy was measured by posterior tibial slope (PTS), tibial varus correction, and joint line restoration.

**Results**

No significant differences were observed between robot-assisted and manual groups in blood loss, postoperative hospital stay, final VAS, HSS, or FJS scores (*P* > 0.05). However, robot-assisted UKA required longer surgery duration (106.19 ± 21.57 min vs. 89.10 ± 19.50 min, *P* < 0.05). One case (2.9%) of pin-track bleeding occurred in the robot group. Postoperative MPTA and MAD improved significantly in the robot group (*P* < 0.05). Compared to the manual group, the robot group demonstrated superior accuracy in PTS (87% vs. 65%, *P* < 0.05), tibial varus correction (67% vs. 55%, *P* < 0.05), and joint line restoration (70% vs. 65%, *P* > 0.05).

**Conclusions**

Robot-assisted UKA shows short-term efficacy in alleviating pain and correcting deformities in AMOA. Although it requires longer surgical time and carries risks of incision-related complications (e.g., pin-track bleeding), its higher prosthesis placement accuracy suggests promising clinical potential.

## O53 The use of augmented reality for lower limb alignment in varus knees for total knee arthroplasty: a pilot study

### Manish Samson^1^, Anirudh Madhav^2^

#### ^1^Senior Consultant-Department of Orthopaedics, Apollo Hospitals, Bannergatta, Bangalore, Karnataka, India; ^2^Arthroplasty Fellow- Department of Orthopaedics, Apollo Hospitals, Bannergatta, Bangalore, Karnataka, India

##### **Correspondence:** Anirudh Madhav (anirudh.madhav03@gmail.com)

*Arthroplasty 2026*, **8(1):**O53

**Background**

Total knee arthroplasty (TKA) remains the standard of care for treating end-stage osteoarthritis of the knee. Approximately 15% to 20% of patients are dissatisfied following surgery. Lower-limb alignment in TKA can affect the longevity of the prosthesis, the functional outcome of the knee, and patient satisfaction. Recently, augmented reality (AR) has emerged as a promising technology in improving the accuracy and outcome of TKA. This study evaluates the accuracy of AR-assisted TKA.

**Methods**

The Pixee Knee + system was used to guide TKA in our institute. Our study involved 18 cases of TKA performed with the Pixee Knee + system from September 2024 to April 2025. The goal was to evaluate its accuracy by directly comparing the planned angular values for lateral distal femoral angle, medial proximal tibial angle, hip-knee-ankle axis, and tibial slope to the intraoperative obtained values and the measured angles on postoperative full leg radiographs. The values obtained and intended by the AR system were tabulated, and the cutting error was less than 1 degree. All patients were followed up with post-operative weight-bearing alignment, AP and lateral radiographs, and pre-operative and post-operative Oxford knee score.

**Results**

Eighteen patients were considered in this study. The Knee + system can perform a cutting error of less than 1° of difference in coronal alignment of femur and tibia, and less than 2° about flexion/extension of femur and posterior tibial slope. The average differences between the values of the angles obtained during surgery and the measurements on post-operative radiographs were minimal. No complications were observed. The Oxford knee score at the end of one-month follow-up was either good or excellent.

**Conclusions**

AR-assisted TKA is precise, time-effective, and has no radiation exposure. This study demonstrates that the Knee + system is accurate and effective in restoring the planned lower limb alignment in varus knees to perform TKA. No complications were reported, but further long-term, multicentric studies are needed for definitive conclusions.

## O54 Optimizing patient selection for periacetabular osteotomy in borderline hip dysplasia: beyond the lateral center–edge angle

### Liqiang Zhang^1^, Hong Zhang^2^, Hui Chen^2^

#### ^1^Shanxi Bethune Hospital, Shanxi Academy of Medical Sciences, Third Hospital of Shanxi Medical University, Tongji Shanxi Hospital, Taiyuan, China; ^2^Senior Department of Orthopedics, the Fourth Medical Center of Chinese PLA General Hospital, Beijing, China

##### **Correspondence:** Hui Chen (shenzhentie@163.com)

*Arthroplasty 2026*, **8(1):**O54

**Background**

Borderline developmental dysplasia of the hip (BDDH), defined by a lateral center–edge angle (LCEA) of 18° to 25°, presents significant challenges in determining optimal treatment strategies. While periacetabular osteotomy (PAO) and conservative treatment are both viable options, the radiographic parameters that should guide this decision remain unclear. We asked: (1) Which radiographic parameters best differentiate between BDDH patients requiring PAO versus those succeeding with conservative treatment? (2) What are the optimal radiographic thresholds for surgical decision-making? (3) How reliable are these measurements for clinical application?

**Methods**

We conducted a retrospective analysis of consecutive BDDH patients (LCEA 18°–25°) treated at our institution between 2018 and 2023. Two independent observers evaluated radiographic parameters, including lateral center–edge angle (LCEA), anterior center–edge angle (ACEA), Tönnis angle, crossover sign (COS), posterior wall sign (PWS), ischial spine sign (ISS), femoral head coverage, femoro-epiphyseal acetabular roof (FEAR) index, and Femoral torsion angle (FTA). Interobserver reliability was assessed using intraclass correlation coefficients (ICC). ROC analysis determined threshold values for surgical intervention. Radiographic parameters were compared between the PAO and conservative treatment groups.

**Results**

The PAO group showed significantly greater dysplasia across multiple parameters other than LCEA. Key findings included lower ACEA (21.0° ± 8.0° vs 24.7° ± 6.2°, *P* = 0.036), higher Tönnis angle (13.4° ± 5.3° vs 8.5° ± 4.6°, *P* < 0.001), and greater FTA (36.5° ± 10.9° vs 30.1° ± 9.2°, *P* = 0.013). Crossover sign positivity (33.3% vs 6.9%, *P* = 0.009) and posterior wall sign positivity (72.2% vs 34.5%, *P* = 0.007) were more prevalent in the PAO group. Tönnis angle emerged as the sole independent predictor of PAO necessity (OR: 1.194, 95% CI: 1.038–1.373, *P* = 0.013), with an optimal threshold of 11.7° (sensitivity 66.7%, specificity 79.3%, AUC = 0.750). All measurements demonstrated excellent interobserver reliability (ICC > 0.81), with Tönnis angle showing the highest reliability (ICC = 0.89, 95% CI 0.85–0.93).

**Conclusions**

Multiple radiographic parameters can reliably distinguish BDDH patients requiring PAO from those suitable for conservative management. A Tönnis angle threshold of 11.7°, combined with ACEA, FTA, crossover sign, and posterior wall sign, provides comprehensive guidance for surgical decision-making. These findings offer quantitative criteria for treatment selection in BDDH patients.

## O55 Risk factors and management strategies for tibial plateau fractures following unicompartmental knee arthroplasty: a retrospective cohort study and literature review

### Jinhua Guo, Zhibing Gong, Zhikun Zhuang, Fudong Xu

#### Quanzhou Orthopedic-Traumatological Hospital, Quanzhou, China

##### **Correspondence:** Jinhua Guo (sunnyguo2023@163.com)

*Arthroplasty 2026*, **8(1):**O55

**Background**

To investigate the pathogenesis and clinical management of tibial plateau fractures (TPF) following unicompartmental knee arthroplasty (UKA) and provide evidence-based recommendations for optimizing perioperative care.

**Methods**

A retrospective analysis was conducted on 752 UKA cases (May 2018–October 2024), identifying 5 TPF cases (incidence: 0.66%). Interventions included: revision to total knee arthroplasty (TKA, *n* = 1), proximal tibial screw fixation (*n* = 1), and conservative management (*n* = 3; 2 cases with 6-week restricted weight-bearing, 1 case diagnosed with delayed healing at 3 months). A systematic literature review (PubMed/EMBASE, keywords: UKA, tibial fracture, complications) was performed for evidence synthesis.

**Results**

At a mean follow-up of 6 months (range: 3–12 months), all patients achieved knee flexion ≥ 120° without pain recurrence, implant loosening, or fracture displacement. Literature analysis identified critical risk factors: Reduced bone density (T-score < − 2.5, OR = 4.3). Prosthesis-tibial size mismatch (OR = 3.8). Surgical technical factors (excessive keel preparation, stress concentration from fixation pin holes, posterior cortical breach). Intraoperative over-impaction (≥ 3 adjustments). Treatment selection depended on fracture displacement (> 5 mm favoring surgery), implant stability, and bone quality.

**Conclusions**

Although rare (< 1%), post-UKA TPF may lead to catastrophic outcomes. Its occurrence correlates with bone quality, implant selection, and surgical precision. A stepwise treatment strategy based on AO/OTA classification is recommended: Type I (non-displaced): Conservative management. Type II (partial displacement): Screw/plate fixation, Type III (complete displacement with implant failure): Revision to TKA.

## O56 Pilot study of septic arthritis irrigation using superoxidized solution

### Muhammad Farhan Md Yusoff, Badrul Akmal Hisham Yusoff, Muhammad Karbela Reza Ramlan, Norlelawati Mohamad, Azwan Aziz

#### Department of Orthopaedics and Traumatology, Faculty of Medicine, Universiti Kebangsaan Malaysia, Kuala Lumpur, Malaysia

##### **Correspondence:** Muhammad Farhan Md Yusoff (farhanafna@yahoo.com)

*Arthroplasty 2026*, **8(1):**O56

**Background**

Septic arthritis is a surgical emergency associated with high morbidity and mortality when not promptly treated. The α-toxin from common organisms swiftly destroys cartilage, necessitating immediate, effective irrigation. Irrigation optimization, including osmolarity, may counteract toxin damage. This study examines Hydrocyn, a superoxidized solution, marking the first study to evaluate its application in septic arthritis irrigation, where no prior evidence currently exists.

**Methods**

A prospective case series was conducted at Hospital Universiti Kebangsaan Malaysia involving ten adult patients (≥ 18 years) with a first episode of knee septic arthritis. Exclusion criteria were inflammatory arthritis, prosthetic joint infections, and immunosuppression (except diabetes). All patients underwent arthrotomy washout using Hydrocyn delivered according to the Hydrocyn Protocol.

**Results**

Early surgical intervention with Hydrocyn irrigation led to a consistent decline in CRP and WCC levels. Most patients demonstrated progressive improvement in knee range of motion by six weeks. Outcomes were favorable across various pathogens and comorbidities. Significant differences were observed between organism groups, suggesting broad applicability. Over 6 weeks, patients showed significant CRP and WBC reduction with improvement in terms of ROM and HSS score (*P* < 0.001).

**Discussion**

Hydrocyn showed promise as a safe and effective adjunct, enhancing infection control and facilitating early functional recovery. Its broad-spectrum antimicrobial properties and non-cytotoxic nature make it suitable even in complex clinical scenarios, including cases with resistant organisms and metabolic comorbidities. The consistent improvement in inflammatory markers and mobility suggests that Hydrocyn may enhance the efficacy of standard surgical debridement.

**Conclusions**

Hydrocyn appears to be a safe and potentially effective adjunct to standard irrigation in the surgical management of septic arthritis. These preliminary results support further research into its clinical benefits and its potential role in improving joint outcomes.

## O57 How much benefit can robot-assisted total hip arthroplasty provide for patients with severe hip dislocations?

### Yu Zhang, Qiangqiang Li, Dongyang Chen, Qing Jiang

#### Department of Orthopedic Surgery, Nanjing Drum Tower Hospital Clinical College of Nanjing Medical University, Nanjing, China

##### **Correspondence:** Qing Jiang (qingj@nju.edu.cn)

*Arthroplasty 2026*, **8(1):**O57

**Background**

Robotic-assisted total hip arthroplasty (RATHA) has rapidly developed and garnered considerable attention in recent years because of providing systematic preoperative planning and precise intraoperative navigation, thereby facilitating personalized and precise THA. However, there are limited reports on the use of RATHA in the treatment of severe hip dislocation. So, can robots offer a different experience for patients with severe hip dislocation compared to conventional THA? Here, we investigated the RATHA technology in anterior approach THA for severe hip dislocations and to evaluate its clinical safety and effectiveness.

**Methods**

Eighteen patients with severe hip dislocations (Crowe type III-IV), including 3 males and 15 females, with an average age of 47.2 ± 11.9 years (range: 23–72 years), were enrolled in our department from October 2022 to October 2023. Preoperatively, the MAKO robot was used to plan the acetabular position, prosthesis type and size, and implantation angle. The anterior approach was used to expose the hip in the supine position, with the acetabular cup prepared and installed under robotic arm assistance, while manual reaming and femoral prosthesis installation were performed. Data were collected on preoperative and postoperative (1 month, 3 months, and 1 year) Harris hip function scores, operative duration, leg length discrepancy, acetabular cup anteversion and abduction angles, preoperative and postoperative hematocrit and hemoglobin levels, and postoperative complications.

**Results**

The average operative duration was 162.5 ± 36.3 min. Hemoglobin levels decreased from 126.2 ± 12.9 g/L preoperatively to 109.0 ± 10.9 g/L postoperatively, and hematocrit decreased from 41.2 ± 12.0% preoperatively to 33.1 ± 3.1% postoperatively, with statistically significant differences (*P* = 0.009). Leg length discrepancy was corrected from an average of 35.4 ± 20.4 mm preoperatively to 9.6 ± 6.2 mm postoperatively. Postoperative measurements showed an average acetabular cup anteversion angle of 19.6 ± 4.1°, which was larger than the preoperative plan of 14.4 ± 1.6°, and an average abduction angle of 40.3 ± 5.1°, which was smaller than the preoperative plan of 43.8 ± 2.6°, with statistically significant differences (*P* = 0.007). The preoperative planning for femoral stem and acetabular cup sizes matched the intraoperative use with 100% consistency. Leg length discrepancy improved from 20.8 ± 15.1 mm preoperatively to 3.8 ± 4.7 mm postoperatively (Table 1). The Harris hip function score increased from an average of 58.2 ± 10.8 preoperatively to 79.9 ± 15.9 at 3 months postoperatively and 89.5 ± 8.3 at 1 year postoperatively, indicating significant improvement in hip function. Two patients (11.1%) experienced proximal femoral fractures, and two patients (11.1%) had postoperative prosthesis dislocations. After open reduction and fixation with braces, no recurrences occurred. No patients experienced complications such as wound infection or poor wound healing.

**Conclusions**

RATHA had demonstrated excellent advantages in the personalized selection of appropriate prosthesis types and sizes, and the prosthesis position and angle. The anterior approach facilitates adequate release of the proximal insertion of the tensor fascia lata, which is beneficial for correcting leg length discrepancy. Significant improvement in hip function can be achieved after 3 months postoperatively. However, the high risk of dislocation due to local structural deformity and muscle disuse atrophy remains an issue that robots cannot overcome. More refined perioperative soft tissue management is required to reduce the risk of dislocation.

**Table 1 (Abstract O57).** Comparison of various indicators before and after mako robot-assisted total hip arthroplasty (THA)

Indicators	Pre-op	Pos-op	*P/t*
Hemoglobin (g/L)	126.2 ± 12.9	109.0 ± 10.9	< 0.01/5.241
Hematocrit (%)	41.2 ± 12.0	33.1 ± 3.1	< 0.01/2.791
Anterversion angle (°)	14.4 ± 1.6	19.6 ± 4.1	< 0.01/4.940
Abduction angle (°)	43.8 ± 2.6	40.3 ± 5.1	0.014/2.591
Offset (mm)	8.6 ± 5.8	8.8 ± 7.4	0.130/0.897
The limb length discrepancy (mm)	35.4 ± 20.4	9.6 ± 6.2	< 0.01/5.130

## O58 Clinical efficacy of patellofemoral joint replacement in treating isolated patellofemoral osteoarthritis

### Yi Yin, Xu Peng

#### Suining Central Hospital Affiliated to Chongqing Medical University, Suining, China

##### **Correspondence:** Yi Yin (78060104@qq.com)

*Arthroplasty 2026*, **8(1):**O58

**Background**

The prevalence of radiographic PFOA was 23.9% (20.5% men vs. 25.8% women). Prevalence of lateral radiographic OA was higher than that of medial radiographic OA at the PF joint. As a method for treating patellofemoral osteoarthritis, the efficacy of patellofemoral joint replacement surgery has been reported inconsistently. This study explores the efficacy of patellofemoral joint replacement surgery in treating isolated patellofemoral osteoarthritis.

**Methods**

A retrospective analysis was conducted on 20 patients with isolated severe patellofemoral osteoarthritis who underwent patellofemoral joint replacement surgery and were followed up from March 2021 to April 2024. Among them, 6 were male and 14 were female; ages ranged from 48 to 71 years, with an average age of 54.7 ± 3.3 years. The surgeries were performed by the same physician using a patellofemoral prosthesis from the American company J&J. The efficacy was evaluated by comparing the Hospital for Special Surgery knee score (HSS) and visual analogue scale (VAS) scores before and after surgery, as well as postoperative excellent rates and patient satisfaction.

**Results**

Twenty patients were followed up for 6 to 24 months, with an average follow-up of 14.68 months. The preoperative HSS scores ranged from 42 to 65, with an average of (52.56 ± 4.51); postoperative scores were (71.04 ± 5.33) at 1 month, (87.136 ± 4.12) at 3 months, and (90.49 ± 5.23) at 12 months, showing significant improvement compared to preoperative scores (*P* < 0.05). The preoperative VAS scores ranged from 3 to 7, with an average of (4.93 ± 0.87); postoperative scores were (1.47 ± 0.69) at 1 month, (0.86 ± 0.57) at 3 months, and (0.36 ± 0.13) at 12 months, also showing a significant reduction compared to preoperative scores (*P* < 0.05) (Figs. 1–3). Among the patients, 10 had excellent outcomes, 2 had good outcomes, resulting in a 100% excellent rate, with significant improvement compared to preoperative conditions. The subjective satisfaction rate among patients was 100%. No complications such as incision infection, patellar dislocation, or prosthesis loosening occurred during the follow-up period.

**Conclusions**

By adhering to strict surgical indications, patellofemoral joint replacement can effectively treat isolated patellofemoral osteoarthritis, significantly alleviate joint pain, and improve joint function.

**Fig. 1 (Abstract O58).**
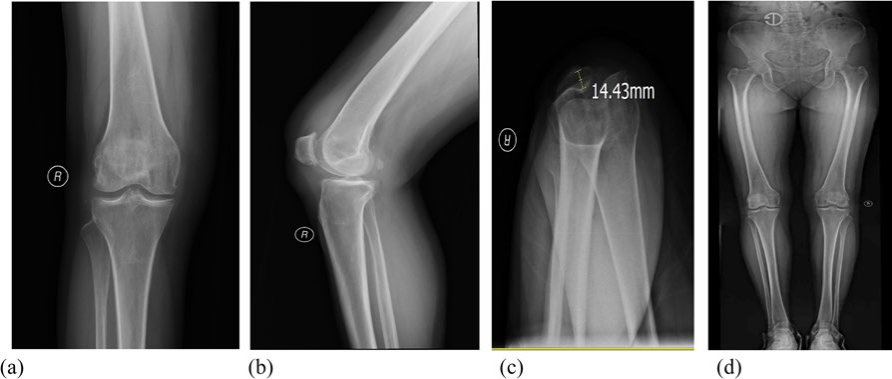
**a** Anteroposterior plain film of the knee joint (weight-bearing position), **b** Lateral plain film of the knee joint (weight-bearing position), **c** Axial flat patellar film (weight-bearing position), **d** total-length plain film of the lower extremity (weight-bearing position)

**Fig. 2 (Abstract O58).**
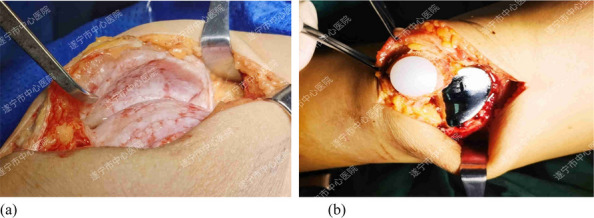
**a** Intraoperative severe osteoarthritis of the patellofemoral joint, **b** The replacement of the hip joint has been completed

**Fig. 3 (Abstract O58).**
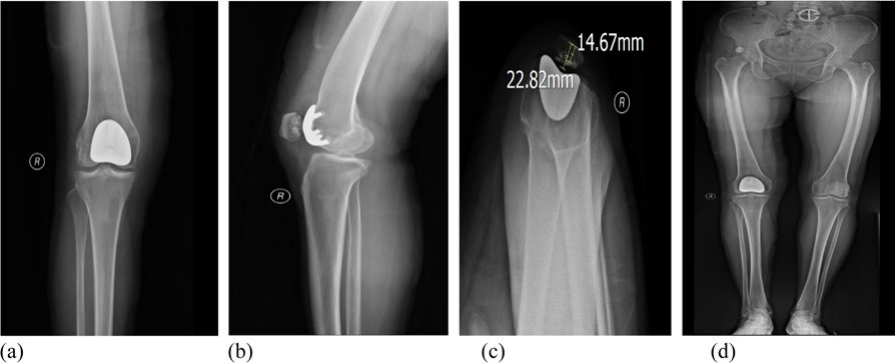
**a** Postoperative anteroposterior plain film of the knee joint (weight-bearing position), **b** Postoperative lateral plain film of the knee joint (weight-bearing position), **c** Postoperative axial flat patellar film (weight-bearing position), **d** Postoperative total-length plain film of the lower extremity (weight-bearing position)

## O59 Application of proximal femoral intertrochanteric rotational wedge osteotomy in periacetabular osteotomy

### Haitao Guo, Shuguang Liu, Yufeng Mei, Jun Li

#### Department of Arthroplasty, Xi’an Honghui Hospital, Xi’an, China

##### **Correspondence:** Jun Li (lijun67@fmmu.edu.cn)

*Arthroplasty 2026*, **8(1):**O59

**Background**

To introduce a surgical method of proximal femoral osteotomy applied in periacetabular osteotomy (PAO) for developmental dysplasia of the hip (DDH) in our department.

**Methods**

A total of 5 patients with DDH admitted to our department from 2022 to 2023 were collected. All of them were female, aged from 24 to 38 years old, with an average age of (30.12 ± 6.58) years. Unilateral surgery was performed on all 5 patients. During the operation, PAO was performed first. After screw fixation, a false oblique fluoroscopy was carried out. When it was found that the anterior coverage of the femoral head was still insufficient, proximal femoral osteotomy (PFO) to reduce the femoral anteversion angle was considered. The lateral central edge angle (LCEA), anterior central edge angle (ACEA), Tonnis angle of the hip joint, as well as the Harris hip score and the international hip outcome tool (iHOT-12) score were recorded and compared before operation and 6 months after operation.

**Results**

The surgeries of all 5 patients were successful. The LCEA and Tonnis angle of the hip joint after operation were significantly improved compared with those before operation (*P* < 0.05), and the ACEA was also satisfactorily corrected (*P* < 0.05). The X-ray examination 6 weeks after the operation showed that the osteotomy lines of the acetabulum and the femoral side were blurred. When the 5 patients were reexamined 3 months after the operation, the X-ray examination showed that the osteotomy sites had completely achieved bony union. The patients were followed up for 10 to 18 months, with an average follow-up period of 15 months. The Harris hip score was > 90 from 6 months after the operation to the last follow-up, and the VAS pain score was between 0 and 2.

**Conclusions**

This PFO surgical technique only requires a moderate extension of the original modified S-P incision. When performing an intertrochanteric osteotomy, if only the anteversion angle needs to be corrected, a horizontal osteotomy and rotation can be carried out. If the neck-shaft angle also needs to be addressed while correcting the anteversion angle, a wedge osteotomy and rotation should be performed, that is, correcting the deformity in two planes, which can make the acetabulum cover the femoral head more fully.

## O60 Rapid detection of periprosthetic joint infection using a nucleic acid-free aptamer-CRISPR/Cas14 biosensor

### Quanbo Ji^1^, Zongjie Geng^2^, Wei Chai^1^, Yan Wang^1^, Guoqiang Zhang^1^

#### ^1^Department of Orthopedics, The General Hospital of Chinese PLA, Beijing, China; ^2^Department of Orthopedics, Yantai Yuhuangding Hospital, Yantai, China

##### **Correspondence:** Guoqiang Zhang (guoqiang301@sina.com); Yan Wang (yanwang301@126.com)

*Arthroplasty 2026*, **8(1):**O60

The full article of this study has been published online. Please refer to the full text at: 10.1016/j.snr.2025.100318.

## O61 Novel technique of kinematic alignment for total knee arthroplasty using VELYS™ robotic-assisted solutions

### Ren Yi Kow^1,2^, Adam Ming Yang Farid Tang^2^, Yong Ng^2^, Jeremy Tze En Lim^2^, Chun Lei Tan^2^, Ming Han Lincoln Liow^2^

#### International Islamic University Malaysia, Kuantan, Malaysia; ^2^Singapore General Hospital, Singapore, Singapore

##### **Correspondence:** Ren Yi Kow (renyi_kow@hotmail.com)

*Arthroplasty 2026*, **8(1):**O61

**Background**

Robotic-assisted total knee arthroplasty (TKA) offers precision and potential improvements in early functional recovery. While robotic assistance has clearly advanced alignment precision in TKA, particularly in mechanical alignment (MA) and functional alignment (FA) workflows, it is important to note that robotic kinematic alignment (robotic KA) remains uncommon and under-validated. We have developed a novel technique for performing KA robotic-assisted TKA using a VELYS™, an imageless, table-mounted robotic system, and present its early outcomes.

**Methods**

This is a retrospective, single-center study of 50 knees from 49 patients who underwent robotic-assisted kinematically aligned total knee arthroplasty (KA-TKA) between 30 January 2024 and 3 July 2024. The first 10 patients were excluded from this analysis as they were considered part of the learning curve for the surgeon adopting this novel technique. Demographic data such as age, gender, height, weight, and body mass index (BMI) were recorded and analyzed. Patients were assessed on postoperative day one (POD1) and at six months. Functional knee parameters, including range of motion (ROM), walking distance, and alignment, were recorded. Pain scores using the visual analogue scale (VAS) were assessed at rest and during movement. Patient-reported outcome measures (PROMs), including the Short-Form 36 (SF-36), Knee Society Knee Score (KSKS), Knee Society Function Score (KSFS), and Oxford Knee Score (OKS), were evaluated. The minimal clinically important difference (MCID) was used for PROMs, and the number of patients achieving MCID for each score was recorded.

**Results**

The mean age was 69.4 years (SD 7.1), with a predominance of female patients (*n* = 32, 64%). The cohort had a mean height of 158.5 cm (SD 8.6), a mean weight of 68.4 kg (SD 13.9), and a mean BMI of 27.3 kg/m^2^ (SD 4.6). Surgical laterality was relatively balanced, with 56% (*n* = 28) undergoing right-sided TKA. On POD1, early functional outcomes were assessed. The mean ROM was 70.4° (SD 20.2), with a mean extension of 4.2° (SD 4.1) and flexion of 74.6° (SD 18.7). The mean walking distance was 19.0 m (SD 13.2). Pain scores measured using the VAS showed a mean of 1.14 (SD 1.60) at rest and 3.36 (SD 2.05) during movement.

At six months postoperatively, a large proportion of patients achieved MCID. Specifically, 86% achieved MCID for the OKS, and 68% reached MCID for the KSKS, demonstrating restoration of knee-specific performance. Improvements extended to broader physical and mental health, with 62% and 36% achieving MCID in the SF-36 Physical Component Summary (PCS) and Mental Component Summary (MCS), respectively. However, only 18% achieved MCID for the KSFS, a measure heavily weighted toward demanding activities such as stair climbing.

**Conclusions**

This preliminary report suggests that robotic KA-TKA yields promising results, particularly in the early postoperative period.

## O62 Total hip arthroplasty for untreated acetabular fractures: a case series

### Christian Emmanuel M. Fontanilla, John N. Hermosisima, Kenneth Alexis M. Yap, Phillipe Y. Baclig

#### Vicente Sotto Memorial Medical Center, Cebu City, Philippines

##### **Correspondence:** Christian Emmanuel M. Fontanilla (christianfontanilla@yahoo.com)

*Arthroplasty 2026*, **8(1):**O62

**Background**

Acetabular fractures are uncommon traumatic injuries that can be sustained from high-energy motor vehicular crashes. Within the past two decades, its incidence has been decreasing in the United States, but the opposite has been observed in Asia, where motorcycle transportation is predominant. When acute, acetabular fractures are managed with internal fixation. In developing countries, treatment is sometimes delayed due to socioeconomic factors and limited access to healthcare facilities. When an acetabular fracture is left untreated for more than three weeks, the hip becomes arthritic, and the femoral head undergoes osteonecrosis. Total hip arthroplasty is indicated to address these complications and allow the earliest return to function, preventing the sequelae of prolonged immobilization such as deep venous thrombosis, pressure sores, and pneumonia. The Forgotten Joint Score (FJS) and the Harris Hip Score (HHS) can be used to document the improvement in function after surgery. In a review of recent literature, only one study in Southeast Asia has documented its outcomes in managing untreated acetabular fractures with total hip arthroplasty. This was published in Indonesia, where six cases were encountered within a span of three years. No similar studies have been published in the Philippines.

**Case Presentation**

This study documented six cases of untreated acetabular fractures managed with total hip arthroplasty in Vicente Sotto Memorial Medical Center, Philippines, in 2023. The cases included five males and one female aged 21–40 years old. All cases resulted from motorcycle crashes, and most presented with untreated posterior wall fractures with posterosuperior, segmental, acetabular defects and chronic posterior hip dislocations (Fig. 1). The chronicity of the fractures ranged from 31.1 weeks to 5.3 years.

**Results**

An untreated acetabular fracture presents with reconstructive challenges: (a) contracted hip musculature, (b) a segmental acetabular defect, (c) a pseudoacetabulum, and (d) a proximally migrated hip center of rotation. These predispose the hip to post-operative dislocation. To prevent this, dual mobility cups were used, and acetabular augments were added when needed (Table 1). Short stems were selected for the femur, given the relatively young age of the patients. In this study, the untreated acetabular fractures managed with total hip arthroplasty resulted in no infection, dislocation, or implant failure in the short-term post-operative period, which ranged from two weeks to one year after surgery (Fig. 2). Functional scores of the six cases improved after surgery by 12–48% in the FJS and by 24–51% in the HHS.

**Conclusions**

Untreated acetabular fractures are managed with total hip arthroplasty to address the accompanying post-traumatic arthritis and femoral head osteonecrosis. Segmental acetabular defects and chronic hip dislocations present in these cases can be managed with acetabular augments and dual mobility cups. When these injuries present in the young, femoral short stems can be used to preserve the femoral neck, maximize the proximal metaphyseal bone stock, and decrease calcar stress-shielding. This study can further be improved by recruiting more cases and continuing to monitor the mid-term and long-term outcomes.

**Fig. 1 (Abstract O62).**
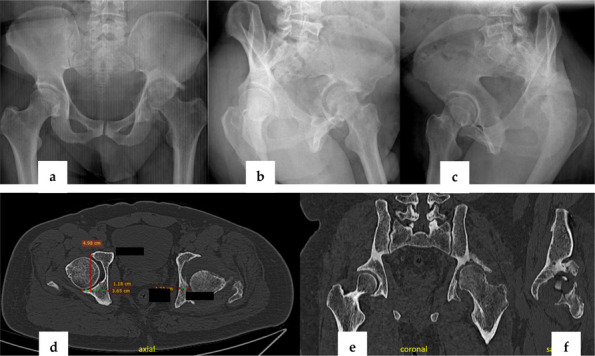
Pre-operative Diagnostic Imaging: **a** pelvis anteroposterior radiograph, **b** pelvis right obturator Judet radiograph; **c** pelvis right iliac Judet radiograph, **d** axial cut of plain pelvis computed tomography scan; **e** coronal cut of plain pelvis computed tomography scan; **f** sagittal cut of plain right hip computed tomography scan

**Fig. 2 (Abstract O62).**
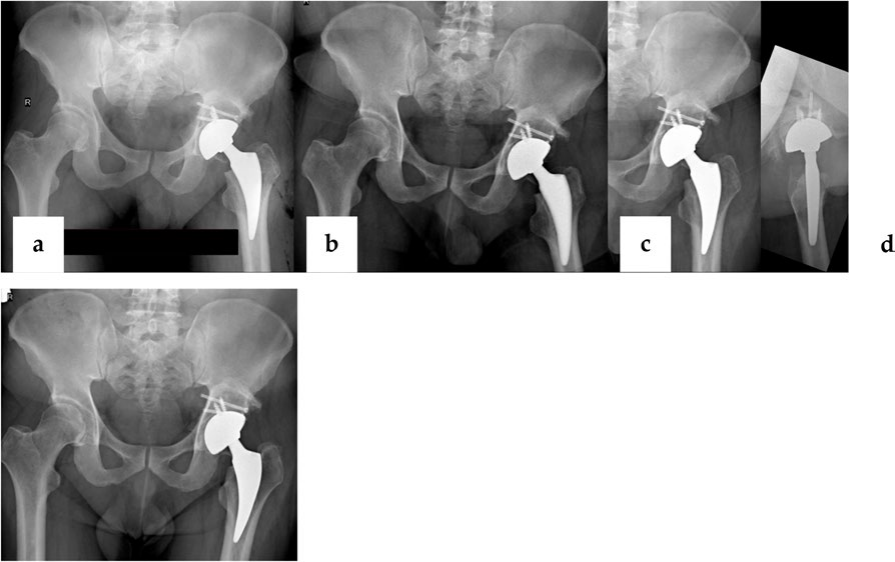
Post-operative radiographs: **a** immediate, **b** two weeks, **c** six weeks, **d** nine months

**Table 1 (Abstract O62).** Implant characteristics of the cases

Case	Acetabular Augment	Acetabular Cup	Femoral Head	Femoral Stem
1	structural autologous bone graft, 54 × 10 mm metal augment	52 mm dual mobility	22 mm metal head	size 2 type 2A short stem
2	none	48 mm dual mobility	22 mm metal head	size 4 type 2A short stem
3	structural autologous bone graft	48 mm dual mobility	22 mm metal head	size 2 type 2A short stem
4	structural autologous bone graft	50 mm dual mobility	22 mm metal head	size 2 type 2A short stem
5	none	50 mm dual mobility	22 mm metal head	size 4 type 2A short stem
6	50 × 20 mm metal augment	48 mm dual mobility	22 mm metal head	size 6 type 2A short stem

## O63 Management strategies for tibial plateau bone defects in primary total knee arthroplasty (TKA) for severe knee degeneration

### Wenteng Si, Wenzhong Chen, Yu Zhou

#### Zhengzhou Orthopaedic Hospital of Joint Surgery, Zhengzhou, China

##### **Correspondence:** Wenteng Si (swteng516@163.com)

*Arthroplasty 2026*, **8(1):**O63

**Background**

To investigate surgical management strategies for severe knee osteoarthritis with tibial plateau bone defects treated by primary total knee arthroplasty (TKA).

**Methods**

Sixty patients with severe knee osteoarthritis and tibial plateau bone defects were included: 50 cases of knee varus and 10 cases of knee valgus. According to the AORI classification, there were 10 cases of Type I, 50 cases of Type II (40 Type IIa and 10 Type IIb). Patients ranged in age from 55 to 76 years (mean, 65.8 ± 3.7 years). All underwent posterior-stabilized prosthesis implantation. Intraoperative techniques included bone spur debridement, tibial plateau reductive osteotomy, and medial soft tissue release to correct deformities. For Type I defects, 8 cases underwent autologous bone grafting and 2 received titanium pillar implants (pilar). For Type IIa defects, 15 cases were managed with cemented screw fixation, and 25 cases with extended stems combined with metal augments. For Type IIb defects, 2 cases used cemented screw fixation, and 8 cases employed extended stems with metal augments. Postoperative follow-up at 6 months and 1 year included X-ray evaluation, knee range of motion (ROM), and Lysholm score assessment.

**Results**

Follow-up ranged from 12 to 36 months (mean, 24 months). The Lysholm score improved significantly from preoperative (20.34 ± 3.12) to final follow-up (87.6 ± 4.25) (*P* < 0.05). Postoperative X-rays showed stable prosthetic positioning without loosening or subsidence. Postoperative knee ROM (88.3 ± 5.4°) was significantly better than preoperative values (54.71 ± 6.3°) (*P* < 0.05).

**Conclusions**

For severe knee osteoarthritis with tibial plateau bone defects, intraoperative techniques such as bone spur debridement, tibial plateau reduction, and medial soft tissue release, combined with defect-specific strategies (e.g., autografts, cemented screws, or extended stems with augments), provide reliable initial stability for the tibial prosthesis. This approach resulted in marked postoperative improvement in knee function and satisfactory clinical outcomes.

## O64 Biomechanical analysis and short-term follow-up of pile foundation reconstruction for severe acetabular bone defects

### Jincheng Wang, Xin Zhao, Qian Wan, Hao Chen, Meng Xu, Aobo Zhang, Qing Han

#### Orthopaedic Department, 2nd Hospital of Jilin University, Changchun, China

##### **Correspondence:** Jincheng Wang (wangjinc@jlu.edu.cn)

*Arthroplasty 2026*, **8(1):**O64

**Background**

Severe acetabular bone defects typically refer to Paprosky type III defects and pelvic discontinuity. The reconstruction surgery for these defects is complex and requires innovative strategies to address implant failure and acetabular reconstruction. Traditional reconstruction methods may be insufficient to adequately resolve severe bone loss and ensure stable fixation. Although customized triflange cups, known for their tailored design, extensive reconstruction capacity, and satisfactory outcomes, offer advantages, their application is limited by factors such as complex design customization, prolonged manufacturing time, high cost, extensive soft tissue dissection during stabilization via extra-acetabular bone fixation, significant surgical trauma, challenging implantation, and uncertain bone apposition. Pile foundation reconstruction, characterized by non-customized design and stabilization through intra-acetabular bone fixation, may serve as an innovative strategy for severe acetabular defect reconstruction. This study established a finite element model of the pile foundation reconstruction prosthesis to analyze its mechanical stability and fabricated it for clinical application, validating the short-term clinical efficacy of the prosthesis.

**Methods**

A three-dimensional model was reconstructed by scanning a sawbone biomechanical model. Based on this model, a pile foundation reconstruction prosthesis (including anterior iliac column, posterior iliac column, pubic, and ischial pile screws) was designed and applied for reconstruction. The finite element analysis loading conditions were derived from intra-articular hip joint loads during gait, measured by Burgmann et al. in patients undergoing hip replacement. Von-Mises stress on the prosthesis and pelvis, as well as relative micromotion at the prosthesis-pelvis interface, were analyzed via finite element analysis to evaluate fracture risk and initial prosthesis stability. The study investigated the required number of pile screws for stability, the load types on each screw, and the range of iliac bone defects that the pile foundation prosthesis could stably reconstruct. Subsequently, the pile foundation reconstruction prosthesis was clinically applied, and an average postoperative follow-up of 14 months was conducted for 6 cases.

**Results**

Finite element analysis demonstrated that posterior iliac column, pubic, and ischial pile screws formed the foundational configuration for prosthesis stability. Under this configuration, the peak prosthesis stress was 188.1 MPa, below the titanium alloy fatigue strength of 795 MPa. The peak micromotion at the prosthesis-bone interface was 28.7 µm, with most interface micromotions remaining below the osseointegration threshold of 28 µm. Load analysis revealed that the posterior iliac column and pubic plate screws bore primary loads. The posterior iliac column screws were subjected to compressive and bending loads, while pubic and ischial screws experienced pull-out and bending loads. The pile foundation prosthesis achieved stable reconstruction of iliac bone defects up to 70 mm in depth. The average 14-month follow-up after prosthesis application demonstrated no postoperative prosthesis loosening and a Harris Hip Score improvement of 48.7 ± 28.7 points (Figs. 1–2).

**Conclusion**

The pile foundation reconstruction prosthesis effectively addresses severe acetabular bone defects, representing a promising solution for complex acetabular reconstruction.

**Fig. 1 (Abstract O64).**
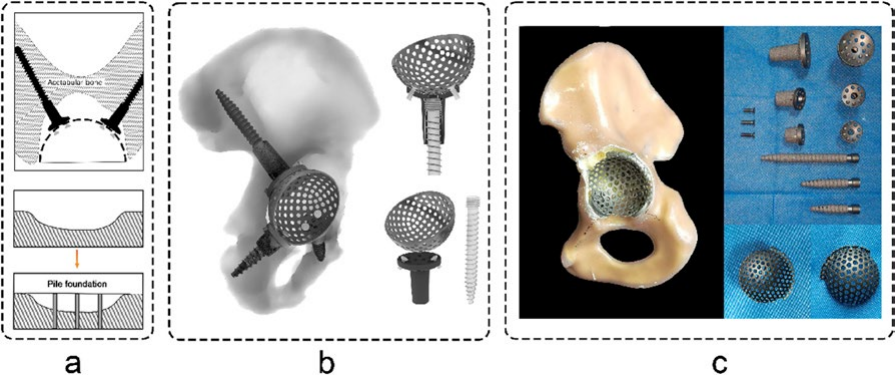
Design of the pile foundation reconstruction prosthesis. **a** Two-dimensional schematic diagram of the pile foundation reconstruction prosthesis; **b** Three-dimensional schematic diagram of the pile foundation reconstruction prosthesis and demonstration of the sleeve connection design; **c** Physical specimen of the fabricated pile foundation reconstruction prosthesis

**Fig. 2 (Abstract O64).**
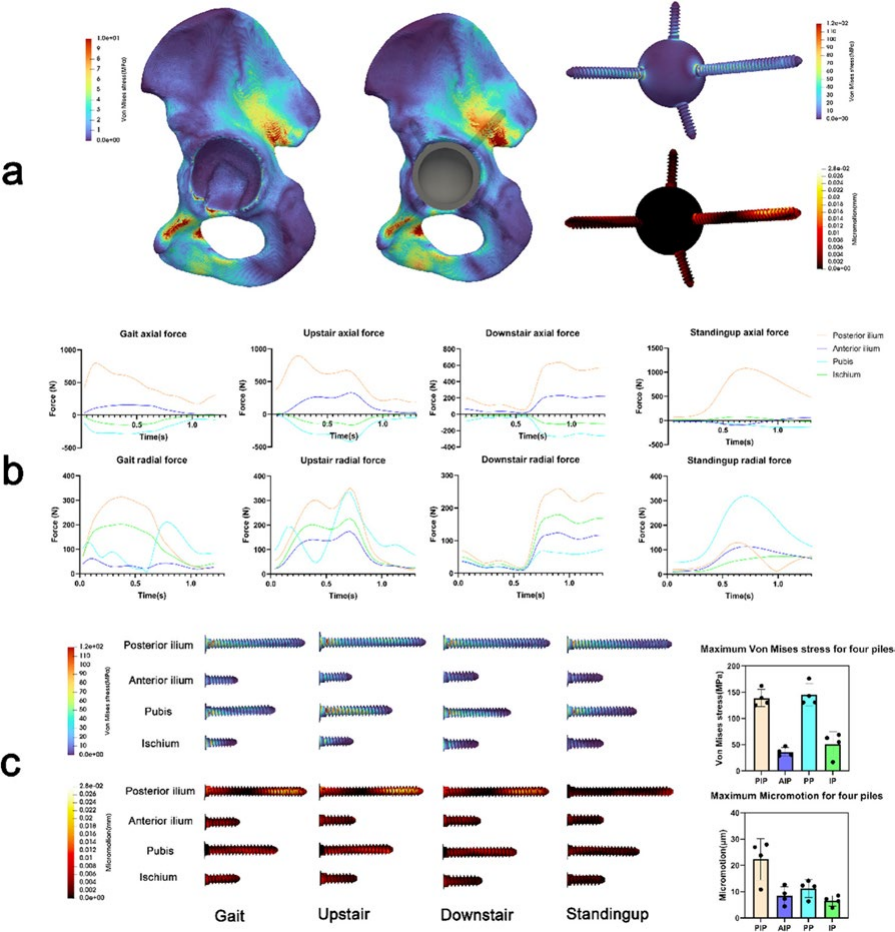
Load, stress, and micromotion of the pelvis and prosthesis under various activities after application of the pile-supported prosthesis. **a** Comparison of pelvic stress distribution between pre- and post-application of the pile-supported prosthesis versus a healthy pelvis, along with prosthesis stress and micromotion; **b** Load curves of each pile under various activities; **c** Stress and micromotion contour maps of each pile under various activities, and statistical graphs of average peak stress and micromotion for each pile. PIP = Posterior ilium pile, AIP = Anterior ilium pile, PP = Pubis pile, IP = Ischium pile

**Fig. 3 (Abstract O64).**
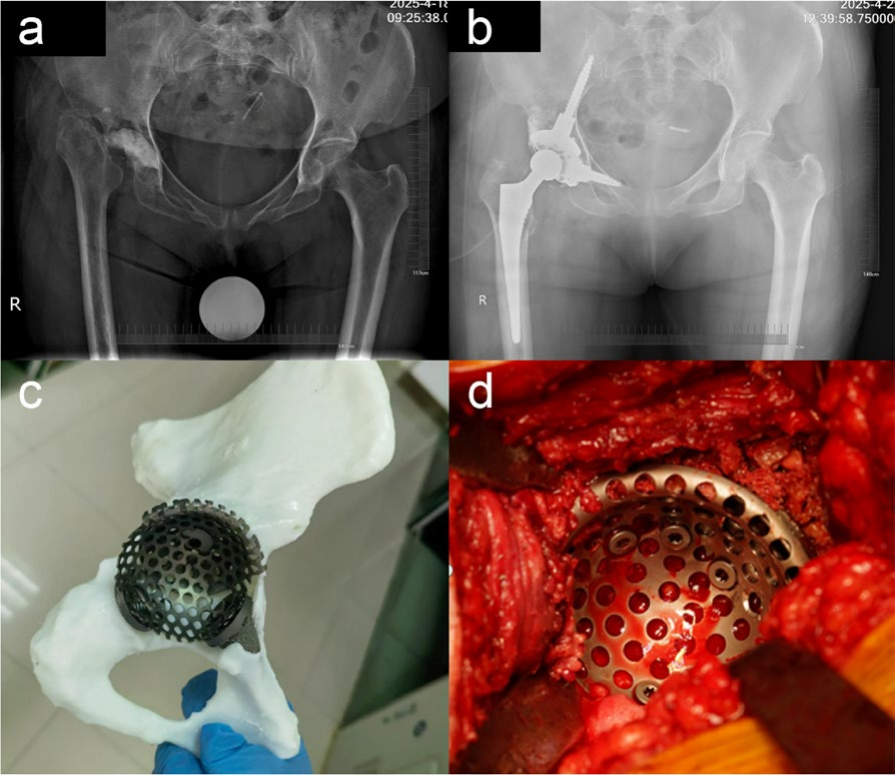
Clinical application of the pile foundation reconstruction prosthesis. **a** Preoperative X-ray; **b** Postoperative X-ray; **c** Preoperative model simulation surgery; **d** Intraoperative surgical field photograph

## O65 Postoperative CT evaluation of pelvic reference pins in computer-assisted total hip arthroplasty

### Tomoki Mae^1^, Yuki Maeda^1^, Wataru Ando^2^, Takeshi Ogawa^2^, Ryo Orito^2^, Satoshi Sasaki^1^, Nobuhiko Sugano^1^

#### ^1^Kawanishi City Medical Center, Kawanishi, Japan; ^2^Kansairousai Hospital, Amagasaki, Japan

##### **Correspondence:** Nobuhiko Sugano (n-sugano@umin.net)

*Arthroplasty 2026*, **8(1):**O65

**Background**

Secure fixation of pelvic reference pins is crucial for accurate cup placement in computer-assisted total hip arthroplasty (THA). While pin site complications are rare in hip surgery compared to knee surgery, serious complications have been reported. This study evaluated pin placement and associated complications using postoperative CT imaging in robotic-arm-assisted and navigated THA.

**Methods**

We retrospectively analyzed 307 hips (269 patients) who underwent THA between April 2023 and April 2025. Of these, 265 hips (229 patients) underwent robotic-arm-assisted THA (Mako), and 42 hips (40 patients) underwent CT-navigated THA. The cohort included 260 hips in females and 47 hips in males, with a mean age of 68.3 years (range: 16–91). Diagnoses included osteoarthritis (285 hips), osteonecrosis (11 hips), rheumatoid arthritis (1 hip), and ankylosing spondylitis (2 hips). Postoperative CT images were evaluated using Vincent software to assess pin trajectory, penetration of inner/outer tables, associated fractures, and hematomas (Fig. 1a). The distance from ASIS to the most anterior pin and intraosseous pin length were measured on 3D bone models (Fig. 1b). Mako procedures used three parallel pins with a guide, while navigation cases used two manually inserted pins.

**Results**

Pin tips in the Mako group were located external to the pelvis in 33%, within the bone in 41%, and internal to the pelvis in 23%. For navigation cases, these percentages were 27%, 54%, and 19%, respectively. The mean distance from ASIS to Pin 1 was 35.0 mm for Mako and 34.1 mm for navigation cases. Average intraosseous pin lengths for Mako were 37.2, 36.2, and 32.1 mm for Pins 1, 2, and 3, respectively, while navigation cases showed 36.1 and 35.2 mm for Pins 1 and 2. The percentage of pin tip position within the pelvis was significantly lower with Mako pin 3 than the others (*P* < 0.05, Chi-square test) (Table 1). No pin-related complications such as fractures, hematomas, lateral femoral cutaneous nerve injuries, or infections were observed.

**Conclusions**

Postoperative CT evaluation of 307 consecutive THAs revealed no pin-related complications. While navigation cases showed higher rates of pin tips within the pelvis compared to Mako cases, likely due to the manual cross-pin insertion technique, neither approach resulted in clinical complications. Further optimization of pin diameter, length, and insertion angle may be warranted, particularly for robotic-assisted cases where pins are placed more proximally on the ilium.

**Fig. 1 (Abstract O65).**
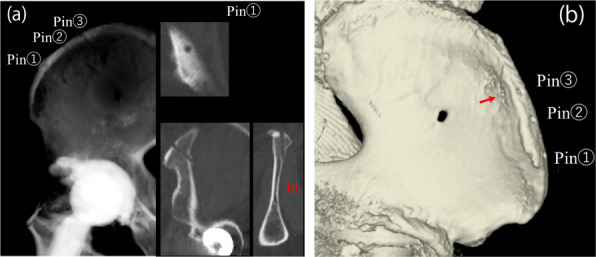
Postoperative CT 3D viewer analysis: **a** A digital projection and MPR views of the ilium; **b** A 3D pelvic model

**Table 1 (Abstract O65).** The percentage of pin tip position at the external pelvic, within the pelvis, and the internal pelvis

	**Mako**			**CT-navigation**	
Pin tip position	Pin①	Pin②	Pin③	Pin①	Pin②
External pelvis (%)	18	22	61	26	29
Within pelvis (%)	56	46	22	57	50
Internal pelvis (%)	27	29	14	17	21

## O66 An innovative approach using intraoperative CT to assess accuracy in patient-specific instrumentation-assisted TKA: comparing osteotomy plane with preoperative planning

### Bin Sun^1^, Liang Yuan^1^, Xiaohua Wang^1^, Ke Zhang^1^, Yichen Yan^2^, Xiaoshu Sun^3^, Jie Yao^2^, Bin Yang^1^

#### ^1^Department of Orthopedics, Peking University International Hospital, Beijing, China; ^2^Key Laboratory for Biomechanics and Mechanobiology of the Ministry of Education, School of Biological Science and Medical Engineering & Advanced Innovation Centre for Biomedical Engineering, Beihang University, Beijing, China; ^3^Department of Industrial Engineering, University of Bologna, Bologna, Italy

##### **Correspondence:** Bin Yang (doctoryangbin@qq.com)

*Arthroplasty 2026*, **8(1):**O66

**Background**

Patient-specific instrumentation (PSI) in total knee arthroplasty (TKA) holds promise for achieving accurate prosthesis alignment, but its precision remains controversial due to limitations in traditional evaluation methods. The key issue is whether PSI enables accurate execution of the planned resections. To date, no in vivo studies have directly validated PSI by comparing osteotomy planes. This prospective study aimed to address this issue by using intraoperative CT to quantify angular discrepancies between the intraoperative osteotomy and the preoperative 3-D planning.

**Methods**

In this prospective study, intraoperative CT scan images of 52 knees were obtained during the TKA procedure. 3-D models were created based on these intraoperative images and superimposed on the preoperative planning models. Angular discrepancies were quantified, and the proportion of outliers of the femur and tibia in coronal, sagittal, and axial planes, as well as united femorotibial angles (UFTA), were evaluated to verify whether this PSI system can precisely replicate the preoperative planning (Figs. 1&2).

**Results**

On the femoral side, the absolute value of the coronal angle discrepancy was 0.87 ± 0.65°, with an outlier ratio of 1.96%, sagittal was 2.09 ± 2.00°, 21.57%, and axial was 1.45 ± 1.26°, 17.65%. For the tibial osteotomy, coronal was 1.38 ± 0.93°, 4.08%, sagittal was 2.18 ± 1.98°, 26.53%, and axial was 4.33 ± 3.43°, 6.12% (Table 1). The angle discrepancy in UFTA was 1.20 ± 0.78° in the coronal plane, 2.87 ± 2.63° in the sagittal plane, and outlier ratios were 2.08% and 37.5%.

**Conclusions**

This study was the first to investigate the angular discrepancies of TKA osteotomy planes using intraoperative CT. The results for PSI-assisted TKA, validated by this innovative method, showed excellent performance in the coronal plane, with minimal angular deviations and low outlier rates.

**Fig. 1 (Abstract O66).**
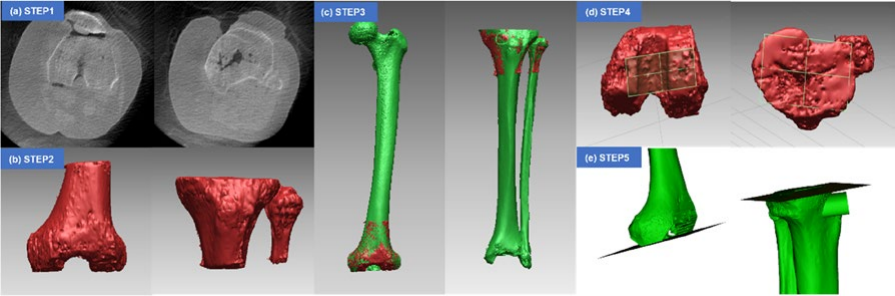
A schematic diagram of the reconstruction, registration, and the osteotomy plane creation. **a** DICOM files. **b** Reconstruction. **c** Registration with pre-operation planning. **d** PSI osteotomy plane creation. **e** Pre-operation planning osteotomy plane creation. The red model depicted the femoral and tibial structures reconstructed from intraoperative CT scan images, whereas the green model illustrated the preoperative planning, representing the anticipated postoperative bone morphology

**Fig. 2 (Abstract O66).**
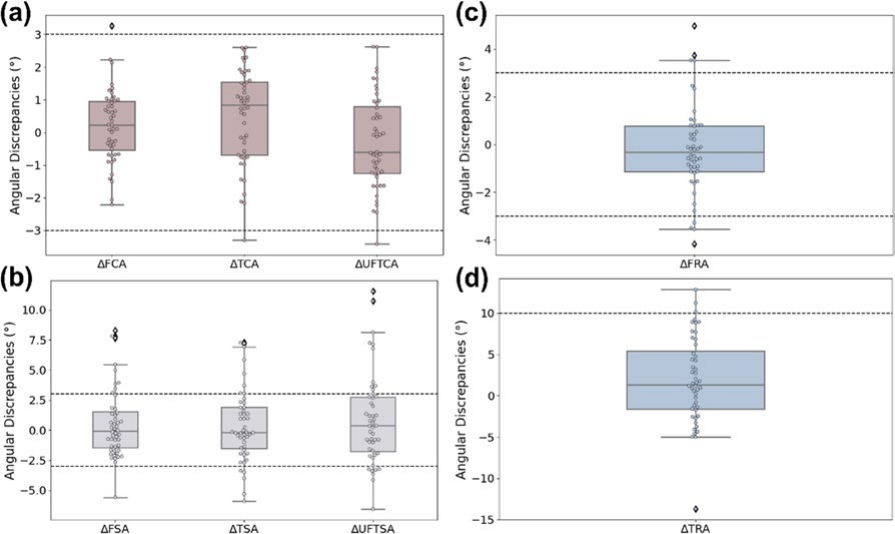
Angular discrepancies between preoperative planning and intraoperative osteotomy. **a** Coronal plane. **b** Sagittal plane. **c** Femoral axial plane. **d** Tibial axial plane. The dashed line indicates the cut-off value for the outlier

**Table 1 (Abstract O66).** Angle discrepancies between preoperative planning and intraoperative osteotomy

Side	Plane	Mean ± SD (°) ^a^	< 1°, n (%)	1–2° n (%)	2–3°, n (%)	Outliers (> 3°), n (%)
Femur	Coronal	0.87 ± 0.65	34 (66.67)	12 (23.53)	4 (7.84)	1 (1.96)
	Sagittal	2.09 ± 2.00	18 (35.29)	15 (29.41)	7 (13.73)	11 (21.57)
	Axial	1.45 ± 1.26	26 (50.98)	11 (21.57)	5 (9.80)	9 (17.65)
Tibia	Coronal	1.38 ± 0.93	21 (42.86)	18 (36.73)	8 (16.33)	2 (4.08)
	Sagittal	2.18 ± 1.98	18 (36.73)	11 (22.45)	7 (14.29)	13 (26.53)
	Axial	4.33 ± 3.43	7 (14.29)	9 (18.37)	6 (12.24)	3 (6.12)^b^
United femoral-tibial angle	Coronal	1.20 ± 0.78	22 (45.83)	19 (39.58)	6 (12.50)	1 (2.08)
	Sagittal	2.87 ± 2.63	13 (27.08)	9 (18.75)	8 (16.67)	18 (37.50)

^a^The numbers in this column were recorded by absolute value

^b^The outliers for tibial rotation were defined as internal rotation exceeding 10°, while outliers in other planes were defined as exceeding 3°

## O67 Robotic-assisted total knee arthroplasty in the presence of extra-articular deformity: reducing complexity and cost

### Sumanth Prabhakar, Jikku Haniball, Kelvin Tan Guoping, Tan Tong Leng

#### Department of Orthopaedic Surgery, Tan Tock Seng Hospital, Singapore

##### **Correspondence:** Sumanth Prabhakar (sumanth.prabhakar@mohh.com.sg)

*Arthroplasty 2026*, **8(1):**O67

**Background**

Performing total knee arthroplasty (TKA) in the setting of significant extra-articular deformity remains a technically challenging endeavour, with distorted bony anatomy and pre-existing hardware often compromising optimal implant placement with conventional TKA techniques. The introduction of robotic systems for TKA has led to improvements in the accuracy and reproducibility of bone resection and component placement, allowing surgeons to overcome many of the limitations of conventional TKA techniques in this setting.

**Methods**

We reviewed a series of 12 robotic-assisted TKAs performed on patients with underlying tibial metaphyseal and diaphyseal deformities, distal femoral deformities, and retained implants. All patients underwent a single-stage primary TKA with the MAKO robotic system, with pre-operative planning based on a 3D CT model of the patients’ knees. Patients were followed up for two years post-operatively with standard weight-bearing radiographs of the knees, full-length lower extremity radiographs, and functional scores. Cost-effectiveness analysis was performed for selected cases, evaluating the cost of a single-stage robotic-assisted TKA against a two-stage conventional TKA (Fig. 1).

**Results**

All 12 patients underwent a single-stage robotic-assisted TKA without the need for revision at the time of the latest follow-up. Radiographically, an average mechanical axis correction of 8.7 degrees was achieved, and all patients were noted to have significant improvements in functional scores and quality of life measures at the two-year follow-up mark. Significant cost savings were demonstrated for cases that utilised the MAKO robotic system to perform a single-stage TKA instead of a conventional two-stage procedure (Table 1).

**Conclusions**

Our experience demonstrates the utility of robotic-assisted TKAs in achieving optimal and cost-effective surgical outcomes in the setting of complex extra-articular deformities, with the aid of enhanced pre-operative surgical planning and intra-operative surgical feedback and guidance through the MAKO robotic system.

**Fig. 1 (Abstract O67).**
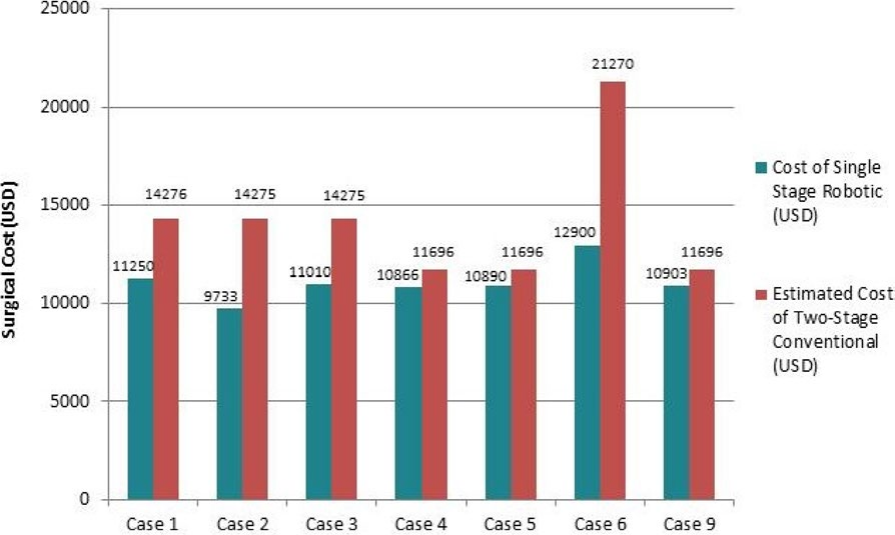
Illustrating the estimated cost savings in select cases with the use of robotic assistance to perform a single-stage procedure, compared to the estimated costs of a two-stage conventional procedure

**Table 1 (Abstract O67).** Details of the type of knee deformities encountered in each of the 12 cases in our series

Case Number	Nature of Extra-Articular Deformity	Description of Deformity	Pre-operative Range of Motion	Pre-operative Mechanical Axis	Post-operative Mechanical Axis
Category A: Retained Implant					
1	Retained Implant	Previous ORIF with LISS Plating of distal femur	0–90	Valgus 4.2	Varus 0.8
2	Retained Implant	Previous Intramedullary Nailing for femoral shaft fracture	16–110	Varus 6.0	Valgus 1.1
3	Retained Implant	Previous high tibial osteotomy (medial opening wedge) with retained implant	0–120	Valgus 3.5	Valgus 2.0
4	Retained Implant	Previous high tibial osteotomy (medial opening wedge) with retained implant	10–120	Varus 2.9	Varus 0.8
5	Retained Implant	Previous high tibial osteotomy (lateral closing wedge) with retained implant	10–120	Varus 13.1	Varus 2.5
Category B: Extra-Articular Femoral Deformity					
6	Femoral	Distal femur fracture with severe varus malunion deformity	10–92	Varus 25.3	Varus 7.5
Category C: Extra-Articular Tibial Deformity					
7	Tibial metaphyseal	Previous high tibial osteotomy (lateral closing wedge)	10–100	Varus 10	Valgus 1.7
8	Tibial metaphyseal	Schatzker V tibia plateau fracture	-5–102	Varus 12	Valgus 3.4
9	Tibial metaphyseal	Previous high tibial osteotomy (lateral closing wedge) with retained implants	5–100	Varus 3.2	Varus 1.1
10	Tibial diaphyseal	Previous tibia shaft fracture with shortening	5–105	Varus 10.7	Valgus 1.4
11	Tibial diaphyseal	Previous tibia shaft fracture	-5–102	Valgus 2.4	Valgus 3.5
12	Tibial diaphyseal	Tibia bowing with angular deformity of tibia diaphysis (etiology unknown)	0 –95	Varus 5.1	Varus 0.5

## O68 Validation of a novel genomic-based host response assay, synvichor, for discriminating septic from aseptic joint pain

### Jonathan Negus^1,2,3^, Antony Rapisarda^1^, Kevin Tetsworth^1,4^, Jeffrey Lipma^1^, Richard Hocking^5^, Andrew Kurmis^1,6^

#### ^1^OrthoDx Scientific Advisory Board, Macquarie Park, Sydney, Australia; ^2^Faculty of Medicine, Health and Human Sciences, Macquarie University, Sydney, Australia; ^3^Jointworks Research Institute, Sydney, Australia; ^4^University of Queensland Medical School, Herston, Australia; ^5^Special advisor to OrthoDx, Coral Coast Orthopaedics, Bundaberg, Australia; ^6^Discipline of Medical Specialties, University of Adelaide, Australia

##### **Correspondence:** Jonathan Negus (jon@jointworks.com)

*Arthroplasty 2026*, **8(1):**O68

**Background**

Acute presentations of swollen and painful joints are common, and their investigation and management are associated with considerable healthcare costs and resource consumption. Differentiating septic arthritis from other aseptic diagnoses remains a challenging yet critical clinical determination. No current, universally accepted, gold standard test exists for this purpose. While microscopy and culture remain the historical standard, these are slow and inaccurate, with poor clinical utility, often prompting non-specific empiric treatment pathways—the cost and morbidity of which cannot be overstated. Cutting-edge science has seen a transition away from non-specific serum markers and crude cell indices towards patient-centric diagnostic genomic assays. These validated and robust methods directly sample the physiologic host response to infection and can rapidly and accurately differentiate septic from aseptic aetiologies. This technology has tremendous potential utility in decreasing the time to diagnosis, while vastly improving diagnostic predictive value and test accuracy. This study aimed to explore the preliminary diagnostic application of the newly developed Synvichor™ assay in an Australian adult cohort. The aim was to evaluate the real-world diagnostic performance of a novel molecular polymerase chain reaction host response assay (Synvichor ™), designed to distinguish between infectious and non-infectious joint pain in both native and prosthetic joints.

**Methods**

The study employed a retrospective, observational, non-interventional design and recruited a heterogeneous cohort of adult patients from seven Australian hospital sites (*n* = 315). The performance of Synvichor™ was compared to standard-of-care diagnostic methods and with prospectively collected clinician diagnoses. Patients were recruited from 7 hospitals across Queensland and South Australia with a combination of rural, regional, and metropolitan facilities. Current standard-of-practice testing approaches were utilised for routine clinical diagnostic pathways. Patients were retrospectively diagnosed using validated Retrospective Physician Diagnosis methods. Standard-of-care diagnostic outcomes were retrospectively assessed against the blinded results of the Synvichor™ assay. Assay performance was determined using receiver operator characteristics (ROC).

**Results**

Synvichor™ demonstrated an ROC area under the curve of 0.95 for discrimination between infectious and non-infectious joint pain compared to 0.15 for culture. The relative likelihood of joint infection versus sterile arthropathy was found to increase with increasing test score (range, 0–37 +). Patient scores were split into four diagnostic bands. Bands (1) and (2) have corresponding negative predictive values (NPV) associated with aseptic inflammation, whilst bands (3) and (4) have corresponding positive predictive values (PPV) associated with septic inflammation. Overall performance resulted in maximum NPV and PPV of > 95%. The results can be obtained within 4 h compared to many days for culture.

**Conclusions**

Synvichor™ is a powerful diagnostic tool that may aid clinicians in rapidly differentiating between septic and aseptic causes of joint pain. Associated improvements in diagnostic confidence and accuracy may translate to substantial healthcare savings through earlier definitive treatment initiation, avoidance of extended emergency department stays, and unnecessary hospital admissions.

## O69 Correlation of pelvic incidence and acetabular orientation using 3D computed tomography image measurement

### Lee Pick Wah^1^, Muhammad Rajaei Ahmad Mohd Zain^1^, Mohammed Rafiq Abdul Kadir^2^, Shaifuzain Ab Rahman^1^, Amran Ahmed Shokri^1^

#### ^1^Department of Orthopaedics, School of Medical Sciences, Universiti Sains Malaysia, Health Campus Kubang Kerian, Kelantan, Malaysia; ^2^Department of Biomedical Engineering, Faculty of Engineering, Universiti Malaya, Kuala Lumpur, Malaysia

##### **Correspondence:** Muhammad Rajaei Ahmad Mohd Zain (rajaei@usm.my)

*Arthroplasty 2026*, **8(1):**O69

**Background**

Acetabular orientation, defined by parameters such as version and inclination, plays a pivotal role in determining the stability and function of the hip joint. It may be influenced by pelvic incidence, a key morphologic parameter in spinal biomechanics that reflects the spatial relationship between the sacrum and the pelvis. Given the interdependence between the spine, pelvis, and hip, understanding this relationship is essential for optimizing outcomes in both hip and spine surgeries. This study aims to investigate the biomechanical association between pelvic incidence and acetabular orientation, thereby elucidating the interplay between pelvic morphology and hip joint alignment.

**Methods**

We analyzed 100 pelvic CT scans of skeletally normal adult patients. Pelvic incidence and acetabular orientation were measured using 3D images reconstructed from the CT scans. Radiographic definitions were utilized to quantify both parameters, and Pearson’s correlation coefficient was employed to assess the relationship between acetabular orientation and pelvic incidence.

**Results**

The mean pelvic incidence among participants was 53.07° ± 9.39°. The mean acetabular orientation measurements were 17.77° ± 4.15° for acetabular anteversion and 45.78° ± 4.87° for acetabular inclination [Table 1]. Statistical analysis revealed no significant correlation between pelvic incidence and either parameter of acetabular orientation (*P* > 0.05) [Table 2]. However, statistically significant gender-based differences were identified in both pelvic incidence and acetabular orientation, suggesting a potential influence of sex-specific pelvic morphology. Pelvic Incidence and acetabular inclination showed weak positive correlation (*r* = 0.19), suggesting a minor tendency for increased pelvic incidence to be associated with greater acetabular inclination angles [Fig. 1].

**Conclusions**

In this study of the normal Malaysian population, no linear correlation was found between pelvic incidence and acetabular orientation. Despite this, the presence of significant gender differences in pelvic parameters highlights the importance of accounting for anatomical variability in orthopedic assessment and surgical planning, particularly in hip and spine-related interventions. Future studies should aim to explore the dynamic interplay between pelvic incidence, pelvic tilt, and acetabular orientation during functional activities such as standing, sitting, and squatting, using weight-bearing 3D imaging modalities.

**Fig. 1 (Abstract O69).**
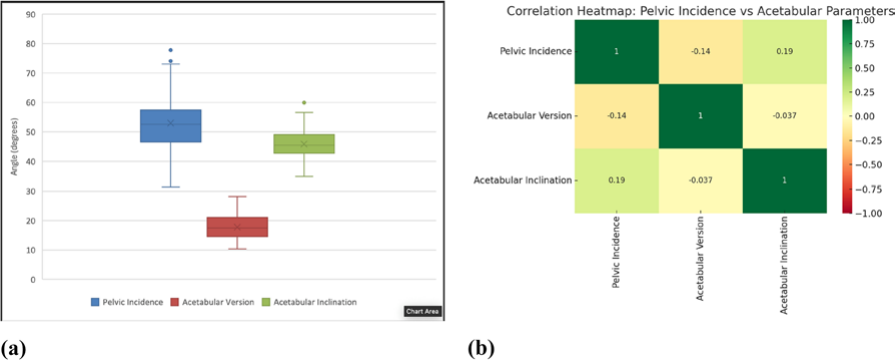
**a** Boxplot showing distribution, variability, and outliers for three parameters. **b** Correlation heatmap of pelvic incidence and acetabular orientation

**Table 1 (Abstract O69).**
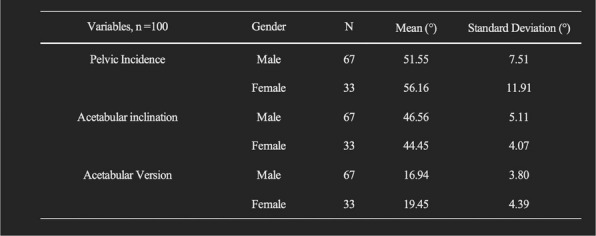
Acetabular morphometric parameters by gender

**Table 2 (Abstract O69).**
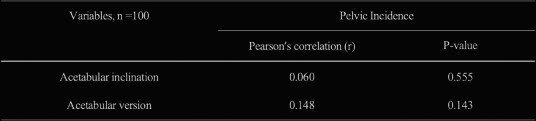
Correlation of pelvic incidence and acetabular orientation

## O70 Defect-specific strategies for femoral reconstruction in revision total hip arthroplasty

### Liang Xiong, Hui Li, Xinzhan Mao

#### Department of Orthopedics, The Second Xiangya Hospital of Central South University, Changsha, China

##### **Correspondence:** Xinzhan Mao (xinzhan.mao@csu.edu.cn)

*Arthroplasty 2026*, **8(1):**O70

**Background**

Femoral bone defects in revision total hip arthroplasty (RTHA) compromise biomechanics and fixation longevity. A comprehensive understanding of femoral bone defects and the establishment of a systematic treatment strategy tailored to the specific type of defects is critical for improving patient functional outcomes and implant survivorship. In this study, we propose defect-specific revision strategies for distinct types of femoral bone defects, based on our institutional experience in a series of RTHA cases.

**Methods**

We retrospectively reviewed 47 patients (48 hips, mean age 59 years; range, 37 to 75 years) who were admitted to our institution from January 2018 to December 2023 and underwent RTHA. The Paprosky classification system was utilized to evaluate the severity of femoral bone defects in all enrolled patients. Preoperative and post-revision Harris scores, VAS scores, and post-revision complications were evaluated.

**Results**

Among all 48 cases, there are 17 cases with Type I defects, 11 Type II, 12 Type III, and 8 Type IV defects (Figs. 1 and 2). In Type I cases, 9 received cemented stems, 4 proximally fixed cementless stems, and 4 distally fixed cementless stems. For Type II defects, 4 underwent cemented stems with impaction bone grafting (IBG) and titanium mesh for proximal femoral reconstruction, while 7 received cemented stems alone. Among Type III defects, 8 were managed with modular stems, and 4 underwent cemented stems in combination with IBG and titanium mesh. In Type IV defects, 5 cases utilized cemented stems with IBG and mesh, 2 received modular stems with IBG and mesh, and 1 was treated with a modular stem alone. These patients all reported significant improvement in their mobilization, Harris, and VAS scores post-operation. No implant migration or change in the center of rotation of the hip joint was observed in the last follow-up. At the latest follow-up, no patient had re-revision or operations related to the prosthesis.

**Conclusions**

Femoral reconstruction can be a complex and time-consuming process in RTHA, as no single technique universally addresses all scenarios. Successful outcomes require both a thorough understanding of the distinct characteristics of bone defect subtypes and mastery of diverse techniques to restore anatomy and biomechanical stability.

**Fig. 1 (Abstract O70).**
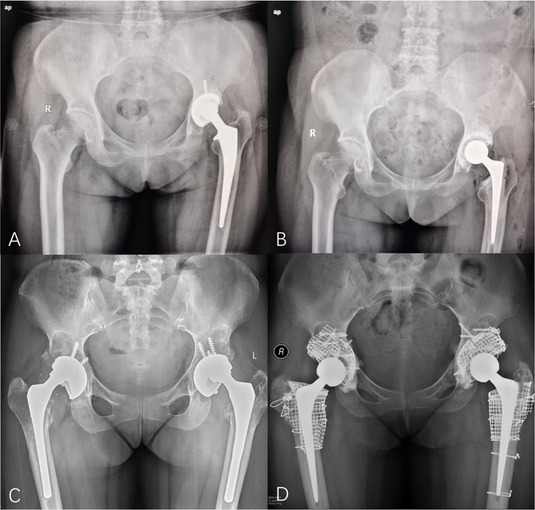
**A**, **B** Pre-op and Post-op X-ray of one case with Paprosky Type I defect; **C**, **D** Pre-op and Post-op X-ray of one case with bilateral Paprosky Type II defect

**Fig. 2 (Abstract O70).**
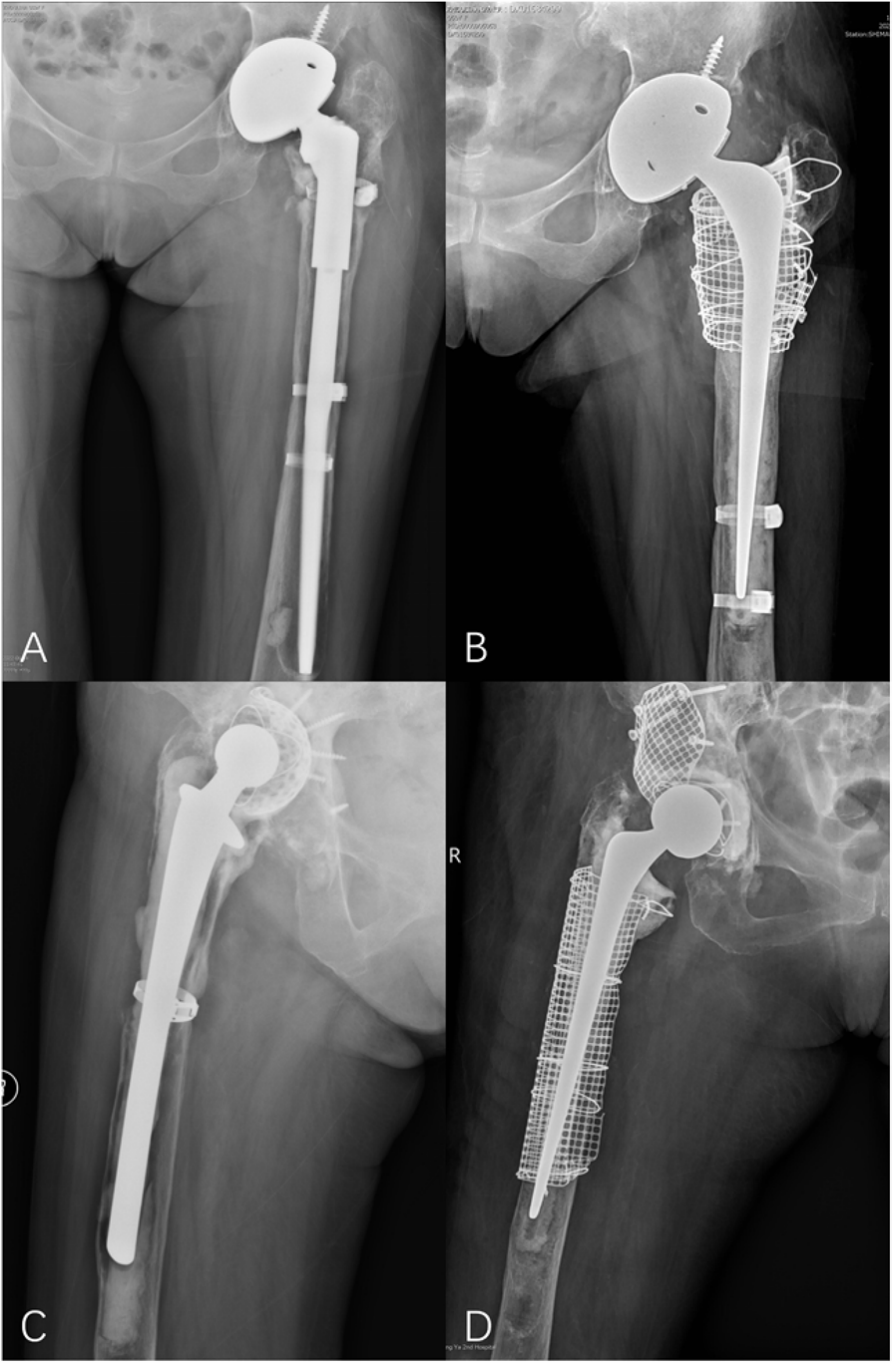
**A**, **B** Pre-op and Post-op X-ray of one case with Paprosky Type III defect; **C**, **D** Pre-op and Post-op X-ray of one case with bilateral Paprosky Type IV defect

## O71 Mid-term outcome of dual-mobility articulation in revision hip arthroplasty of porous revision acetabular component

### Shahril Shaarani^1^, Ayobami Asaju^2^, Mohammed Shaeir^3^, Sujith Konan^3^

#### ^1^Connolly Hospital 1, Dublin, Republic of Ireland; ^2^Leeds Hospital NHS Trust, Leeds, UK; ^3^University College London Hospital, London, UK

##### **Correspondence:** Shahril Shaarani (shahrilr.shaarani @gmail.com)

*Arthroplasty 2026*, **8(1):**O71

**Background**

This retrospective study aims to inform of the outcome of cementing dual-mobility articulation in porous revision shell at mid-term follow-up.

**Methods**

From a total of 102 revision hips between 2015 and 2021 in our revision database, we identified 47 cemented dual mobility articulations. Indications for revision total hip arthroplasty (rTHA) included aseptic failure of the acetabular shell (*n* = 34), infection (*n* = 7), and dislocation (*n* = 6). For this study, we only included those patients who had cemented dual mobility articulation in a porous revision shell and a minimum of one year of follow-up.

**Results**

No complications were noted intra-operatively. There was no dislocation for any of the patients. There was also no radiological evidence of loosening at the latest follow-up. The mean OHS improved from 24 (SD 13) preoperatively to 42 (SD 15) postoperatively. There has been no revision performed at the latest follow-up, and no peri-prosthetic joint infection.

**Conclusions**

No complications were noted intra-operatively. There was no dislocation for any of the patients. There was also no radiological evidence of loosening at the latest follow-up. The mean OHS improved from 24 (SD 13) preoperatively to 42 (SD 15) postoperatively. There has been no revision performed at the latest follow-up, and no peri-prosthetic joint infection.

## O72 Comparison of pain and functional performance between crystalline glucosamine sulfate and diacerein in early knee OA patients

### Chavarin Amarase^1,2^, Aree Tanavalee^1,2^, Srihatach Ngarmukos^1,2^, Chotetawan Tanavalee^1,2^, Nonn Jaruthien^1,2^, Pakpoom Somrak^1,2^, Saran Tantavisut^2^

#### ^1^Biologics for Knee Osteoarthritis Research Unit, Faculty of Medicine, Chulalongkorn University, Bangkok, Thailand; ^2^Department of Orthopaedics, Faculty of Medicine, Chulalongkorn University and King Chulalongkorn Memorial Hospital, Thai Red Cross Society, 1873 Rama IV Road, Bangkok, Thailand

##### **Correspondence:** Chavarin Amarase (tueskung@hotmail.com)

*Arthroplasty 2026*, **8(1):**O72

**Background**

Among symptomatic slow-acting drugs for osteoarthritis (SYSADOA) used to treat early knee osteoarthritis (OA), oral patented crystalline glucosamine sulfate (pCGS) and diacerein have gained popularity for controlling symptoms and slowing progression of the disease. Although studies have shown that pCGS and diacerein improve clinical outcomes, no study has compared the results between these two agents. We compared pain and functional performance between pCGS and diacerein in patients with early knee osteoarthritis (OA).

**Methods**

We retrospectively reviewed 275 patients with Kellgren-Lawrence (KL) grades I and II knee OA, who were continuously treated with either 1,500 mg pCGS per day or 100 mg diacerein per day for 1 year. The visual analogue scale (VAS) was used to assess pain, and the 5-time sit-to-stand test (5xSST), the time up-and-go test (TUGT), and the 3-min walk distance test (3MWDT) were employed to evaluate functional performance outcomes at baseline, 12-week, 24-week, and 1-year follow-ups (FUs). Changes in all parameters were evaluated and compared between the two groups.

**Results**

At the final follow-up, 215 patients completed all evaluations, with an average age of 65 years, a BMI of 26.9 kg/m^2^, and 75% being female. There were 129 patients receiving p-CGS and 86 receiving diacerein. The pCGS group showed significant improvements in VAS, 5xSST, and TUGT from the 12th week and significant improvement in 3MWDT from the 24th week. In contrast, the diacerein group showed significant improvements in TUGT from the 12th week and significant improvements in VAS, 5xSST, and 3MWDT from the 24th week. At 1-year FU, both groups had significantly improved VAS and all three functional performances without adverse events. However, there were no statistically significant differences in VAS, 5xSST, TUGT, and 3MWDT between the two groups at baseline and all three follow-up time points.

**Conclusion**

In early knee OA patients, according to individual baseline evaluations, pCGS provided faster and significantly improved VAS and 5xSST, as well as similar improvements in TUGT from the 12th week, compared to diacerein; however, there were no differences between the two groups. From the 24th week to one year, both agents provided similarly significant improvements in pain and all functional performance tests.

## O73 Does adding corticosteroids to the cocktail solution for local infiltration analgesia in TKA minimise knee inflammation? A randomised controlled trial

### Chavarin Amarase^1,2^

#### ^1^Biologics for Knee Osteoarthritis Research Unit, Faculty of Medicine, Chulalongkorn University, Bangkok, Thailand; ^2^Department of Orthopaedics, Faculty of Medicine, Chulalongkorn University and King Chulalongkorn Memorial Hospital, Thai Red Cross Society, 1873 Rama IV Road, Bangkok, Thailand

##### **Correspondence:** Chavarin Amarase (tueskung@hotmail.com)

*Arthroplasty 2026*, **8(1):**O73

**Background**

Adding corticosteroids to the cocktail solution for local infiltration analgesia (C-LIA) in total knee arthroplasty (TKA) has been shown to decrease postoperative pain and accelerate functional recovery. While most studies on C-LIA have focused on improving clinical outcomes, there have been limited investigations evaluating changes in knee inflammation associated with C-LIA. We compared changes in synovial fluid proinflammatory cytokines and knee inflammation parameters between patients undergoing total knee arthroplasty (TKA) who received C-LIA and those who received corticosteroid-free LIA (CF-LIA).

**Methods**

One hundred patients with late-stage knee osteoarthritis undergoing unilateral primary TKA were randomised to receive intraoperative CF-LIA and C-LIA. The CF-LIA mixture consisted of 20 mL of 0.5% bupivacaine, 0.3 mL of 1:1000 adrenaline, 30 mg of ketorolac, and 0.9% saline solution, for a final volume of 40 mL. The C-LIA mixture consisted of similar agents with an additional 40 mg of Solumedrol. The baseline synovial fluid (SF) was collected at surgery, and subsequent SF samples were collected on postoperative day 3 (POD3) in all patients. They were analyzed for proinflammatory and inflammatory cytokines, including IL-1β, IL-2, IL-4, IL-6, IL-8, IL-10, IL-12p70, IL-17α, IP-10, TNF-α, TGF-β1, MCP-1, and IFN-γ. Knee inflammation parameters, including VAS for pain, skin temperature, and range of motion, were recorded at preoperative, 6 h, 12 h, 24 h, 48 h, and 72 h postoperatively and analyzed between the two groups.

**Results**

There were 50 patients in each group. The mean ages of the C-LIA and CF-LIA groups were 67.8 years and 67.0 years, respectively (*P* = 0.81), with a predominance of females in both groups. There were no perioperative complications, and all patients were ambulated and discharged according to the 3-day protocol. Regarding changes in cytokine levels between baseline and POD3, there was a significant increase in the levels of IL-1β, IL-6, IL-17α, TNF-α, and MCP-1 in both C-LIA and CF-LIA groups. However, there were no significant differences in the changes in all cytokines between the two groups. All knee inflammation parameters were not significantly different between the two groups at preoperative and postoperative records.

**Conclusions**

The significant increase in the levels of inflammatory cytokines in synovial fluid confirmed the inflammation after TKA in both C-LIA and CF-LIA groups. However, although studies have shown that adding corticosteroids to the cocktail solution decreases postoperative pain and accelerates functional recovery, the present study found that it did not minimize the level of knee inflammation.

## O74 Femoral stem design influences early periprosthetic fracture risk following total hip arthroplasty

### Alexandra N. Krez^1^, Sean D. Toomey^2^, Kyle C. Swanson^3^, Carl L. Herndon^4^, Jonathan R. Danoff^1^

#### ^1^Department of Orthopaedic Surgery, North Shore University Hospital, Northwell Health, Manhasset, NY, USA; ^2^Orthopedic Physician Associates, PLLC, Department of Orthopedic Surgery, Swedish Orthopedic Institute, Seattle, WA, USA; ^3^The Orthopaedic & Fracture Clinic, Mankato, MN, USA; ^4^Department of Orthopedic Surgery, Columbia University Medical Center, New York, NY, USA

##### **Correspondence:** Sean D. Toomey (sdtoomey10@gmail.com)

*Arthroplasty 2026*, **8(1):**O74

**Background**

Periprosthetic femur fracture (PFF) is a serious complication after total hip arthroplasty (THA), with risk influenced by patient and implant factors. This study assessed whether a triple-taper, metaphyseal-filling femoral stem with a collar design reduces early PFF risk compared to other cementless stems. Secondary aims included evaluating fracture risk by age, femoral bone quality, and stem collar position (proud vs. on-the-calcar).

**Methods**

This retrospective study included 774 patients who underwent primary cementless THA: triple-taper metaphyseal stems (*n* = 311), single-wedge (*n* = 294), double-wedge (*n* = 116), and gradual taper/metadiaphyseal-filling (*n* = 53). The triple-taper cohort was drawn from a multicenter clinical trial, while comparators were from a single “urban health system” (name underlined for anonymization). Patients were stratified by stem type, age, Dorr bone quality, and collar position. The primary outcome was 90-day PFF. Logistic regression identified PFF predictors.

**Results**

PFF occurred in 53 patients (6.8%), with 38 (4.9%) requiring reoperation. Patients with PFF were older (mean 70.5 vs. 65.1 years; *P* = 0.001) and predominantly female (73.6% vs. 56.0%; *P* = 0.020). Triple-taper metaphyseal stems had significantly lower PFF risk than other stem types (OR 0.20; 95% CI, 0.08–0.48; *P* < 0.001). PFF incidence was 1.9% for triple-taper stems vs. 7.8% (single-wedge; *P* < 0.001), 14.7% (double-wedge; *P* < 0.001), and 13.2% (gradual taper; *P* < 0.001). These differences held across age groups and Dorr A/B femora. Within the triple-taper group, a proud collar was associated with increased PFF risk (6.3% vs. 0.8%; *P* = 0.020).

**Conclusions**

Triple-taper metaphyseal stems significantly reduce early PFF risk, independent of patient age or bone quality. On-the-calcar collar positioning further minimizes fracture risk. Stem design and collar seating are important considerations in preventing early postoperative PFF following THA.

## O75 Does anterior cruciate ligament damage really affect unicompartmental knee arthroplasty outcomes in the elderly?

### Pengfei Wen, Yidian Wang, Zhi Yang

#### Honghui Hospital, Xi’an Jiaotong University, Xi’an, China

##### **Correspondence:** Pengfei Wen (Wenpengfei@pku.edu.cn)

*Arthroplasty 2026*, **8(1):**O75

**Background**

An intact anterior cruciate ligament (ACL) has traditionally been considered crucial for the success of unicompartmental knee arthroplasty (UKA). However, with the evolution of surgical techniques and advancements in prosthesis design, UKA has increasingly been attempted in patients with ACL damage due to its advantages of minimal invasiveness, reduced bleeding, and faster recovery. This study aims to investigate the impact of ACL damage on the clinical outcomes of UKA.

**Methods**

We conducted a retrospective analysis of 43 patients (57 knees) with anteromedial osteoarthritis (AMOA) who underwent fixed-platform UKA at Xi’an Jiaotong University Affiliated Honghui Hospital between January 2022 and December 2023. Patients were categorized into three groups based on preoperative magnetic resonance imaging (MRI) and intraoperative findings: ACL intact group (23 knees), partial ACL damage group (28 knees), and complete ACL damage group (6 knees). We collected demographic information, preoperative hip-knee-ankle angle (HKA), follow-up duration, and preoperative and postoperative data over one year, including Hospital for Special Surgery (HSS) knee score, Lysholm score, visual analog scale (VAS) for pain, range of motion (ROM), and radiographic data. Statistical analysis was performed to determine clinical outcome differences among the three groups, and postoperative complications were recorded.

**Results**

The average age of the included patients was 74.6 years (range: 69–85 years), with an average follow-up of 49 months (range: 16–53 months). There were no significant differences among the three groups in age, body mass index (BMI), follow-up duration, preoperative HKA, baseline Lysholm score, HSS knee score, VAS score, or ROM (*P* > 0.05). Postoperatively, all three groups showed significant improvements in Lysholm score, HSS knee score, VAS score, and ROM (*P* < 0.001), with no significant differences in the degree of improvement among the groups (*P* > 0.05). Radiographic review revealed no significant differences in the position of the femoral component relative to the tibial component among the three groups (*P* > 0.05). No radiolucent lines were observed in any patient, and no infections, aseptic loosening, periprosthetic fractures, or dislocations requiring revision were noted at the most recent follow-up.

**Conclusions**

For elderly patients with AMOA and stable knee alignment in the anteroposterior direction, ACL damage does not adversely affect the mid-to-short-term outcomes of fixed-platform UKA. Even patients with partial or complete ACL damage can achieve clinical outcomes comparable to those with intact ACLs through strict patient selection and optimized surgical techniques. We suggest that ACL damage should not be considered an absolute contraindication for UKA, although further long-term studies are needed to confirm its durability and efficacy.

## O76 Application of multi-point fixation technology and theoretical system in joint revision surgery: a new approach for severe bone defects

### Pengfei Wen, Yidian Wang, Shouye Hu, Zhi Yang

#### Honghui Hospital, Xi’an Jiaotong University, Xi’an, China

##### **Correspondence:** Pengfei Wen (Wenpengfei@pku.edu.cn); Zhi Yang (xgcgfd@126.com)

*Arthroplasty 2026*, **8(1):**O76

**Background**

With the aging population and increasing incidence of joint diseases, the demand for joint revision surgeries is rising. Traditional techniques for managing severe bone defects in joint revision surgeries, such as structural bone grafting and acetabular reinforcement rings, have limitations in providing adequate initial stability and long-term fixation. The multi-point fixation technology and theoretical system, combined with computer-aided design and 3D printing, offer a promising solution by enabling personalized prosthesis design and enhanced stability.

**Methods**

A prospective study was conducted on patients with severe bone defects undergoing joint revision surgeries at the Honghui Hospital, Xi’an, China. The study included patients with acetabular bone defects classified as Paprosky III or IV, who were treated with multi-point fixation technology. Preoperative planning involved CT scans for detailed bone defect assessment, followed by personalized prosthesis design using computer-aided design (CAD) software. The prostheses were manufactured using 3D printing with porous tantalum metal, ensuring a high match with the patient’s anatomy. The surgical procedure included precise implantation of the prosthesis according to the preoperative plan. Postoperative management involved pain control, infection prevention, and rehabilitation training. Clinical outcomes were assessed using the Harris Hip Score (HHS), visual analog scale (VAS) for pain, and the European Quality of Life Five Dimensions Five Levels (EQ-5D-5L) index. Radiographic evaluations were performed to assess prosthesis positioning and bone integration.

**Results**

A total of 30 patients (35 hips) were included in the study, with an average age of 68 years (range: 55–78 years) and a mean follow-up of 24 months (range: 18*–*30 months). Preoperatively, the average HHS was 45.6 (range: 38*–*52), which improved to 89.2 (range: 83*–*95) postoperatively (*P* < 0.001). The average VAS score decreased from 7.8 (range: 6*–*9) preoperatively to 1.4 (range: 0*–*3) postoperatively (*P* < 0.001). The EQ-5D-5L index showed significant improvement in quality of life, with an average score increasing from 0.45 (range: 0.32*–*0.58) preoperatively to 0.82 (range: 0.75*–*0.88) postoperatively (*P* < 0.001). Radiographic analysis revealed no signs of prosthesis loosening or radiolucent lines, indicating successful bone integration. No postoperative infections, aseptic loosening, periprosthetic fractures, or dislocations were observed.

**Conclusions**

The application of multi-point fixation technology and theoretical system in joint revision surgery demonstrates significant improvements in clinical outcomes and patient quality of life. The personalized prosthesis design and enhanced stability provided by this technology offer a viable solution for managing severe bone defects in joint revision surgeries. Further long-term studies are warranted to confirm the durability and efficacy of this approach. This innovation has the potential to revolutionize the management of complex joint revision cases, providing better functional outcomes and reduced complications.

## O77 Correlation of patient native anatomy and postoperative component placement with demographic predictors of hip outcome scores in mako robotic-arm assisted total hip arthroplasty: a prospective analysis

### Carl Cedric Celera^1^, Qistina Adyra^2^, Kwong Weng Loh^2^, Hwa Sen Chua^1^

#### ^1^Sunway Medical Centre, Bandar Sunway, Subang Jaya, Selangor; ^2^Department of Orthopaedic Surgery, Faculty of Medicine, University Malaya, Kuala Lumpur, Malaysia

##### **Correspondence:** Carl Cedric Celera (cccelera@gmail.com)

*Arthroplasty 2026*, **8(1):**O77

**Background**

Accurate replication of native femoral and acetabular anatomy in total hip arthroplasty (THA) is crucial for optimal implant positioning and postoperative function. The Mako robotic-arm-assisted system offers enhanced precision in component placement. This prospective study investigates the correlation between patient native anatomy and postoperative femoral and acetabular positioning, and evaluates demographic predictors of hip outcome scores.

**Methods**

The study involved 171 patients (195 hips) undergoing primary THA performed by a single surgeon via the posterior approach using Mako robotic assistance. Preoperative native femoral version, acetabular anteversion, and inclination were compared with postoperative computed tomography (CT)-based component positioning. Scattergrams assessed the relationship between native acetabular anatomy and postoperative alignment, with adherence to Lewinnek and Dorr safe zones. Outcomes were measured using the Harris Hip Score (HHS) and Hip Disability and Osteoarthritis Outcome Score (HOOS). Repeated measures ANOVA evaluated HHS and HOOS over time (preoperative, 3 months, and 1 year). Analysis of covariance (ANCOVA) was used to identify demographic predictors of clinical outcomes, and pairwise comparisons were conducted to identify significant differences (*P* < 0.05).

**Results**

Mean patient age was 60.8 ± 12.5 years; 60.2% were female, 91.8% Chinese, and 45.1% overweight. Robotic-assisted THA demonstrated consistent acetabular placement accuracy: mean planned cup inclination was 43.6° ± 2.1°, with an achieved inclination of 42.1° ± 5.5°. Planned cup version averaged 19.5° ± 1.9°, closely matched by a postoperative version of 27.9° ± 6.8°. Femoral component positioning showed greater variability, with planned femoral version at –1.8° ± 14.9° and postoperative femoral version at 14.3° ± 10.4°. Planned stem version averaged 18.9° ± 9.9°, compared to 11.4° ± 9.0° postoperatively.

CT-based assessment showed a mean acetabular inclination of 42.1° ± 5.5°, with 92.2% of cases within the Lewinnek safe zone (30°–50°). Mean postoperative combined anteversion was 40.9° ± 13.4°, with Dorr safe zone adherence observed slightly above 50.0% of the cohort (25°–45°) (Fig. 1).

Repeated measures ANCOVA demonstrated significant improvements in both HHS and HOOS across all time points (preoperative, 3 months, and 1 year postoperative; *P* < 0.001), with post-hoc analyses indicating significant differences between each interval. ANCOVA identified age and sex as significant demographic predictors of postoperative outcomes. Specifically, patients aged < 55 had significantly higher HHS scores at 3 months compared to those aged > 65 (*P* = 0.046), and females had significantly higher HHS scores at 1 year compared to males (*P* = 0.016) (Tables 1 & 2).

**Conclusions**

Robotic-assisted THA achieved high accuracy in component placement and significant functional improvements, regardless of demographic factors. Minor deviations from traditional safe zones did not impact short-term recovery. Further follow-up is needed to determine the long-term effects on implant longevity and functional durability.

**Fig. 1 (Abstract O77).**
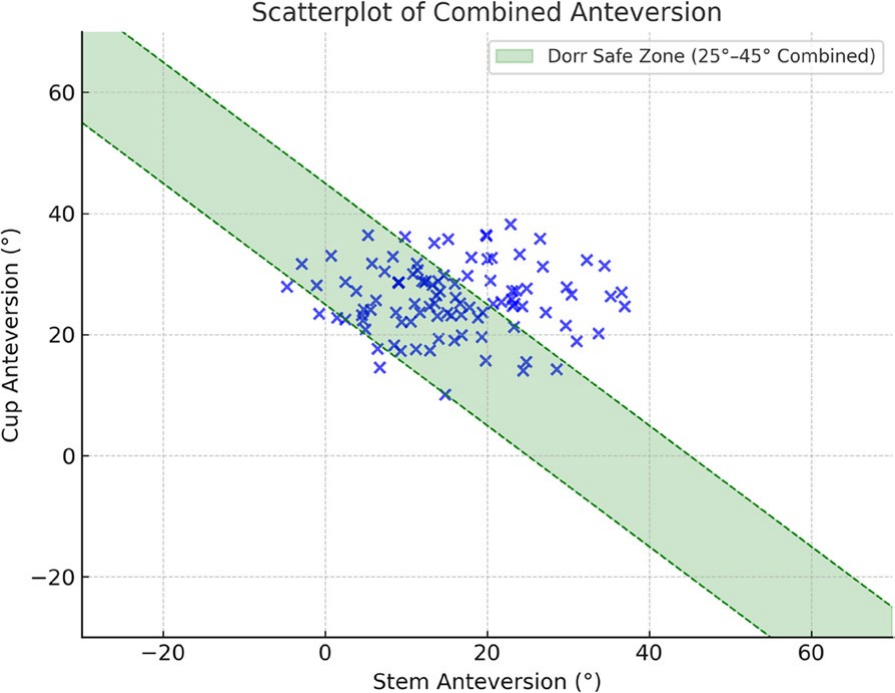
Scatterplot of postoperative stem and cup anteversion angles. The green shaded area represents the Dorr Safe Zone, corresponding to a combined anteversion between 25° and 45°

**Table 1 (Abstract O77).** Analysis of covariance showing demographic predictors of clinical outcome measures at 3 m versus preoperative

Demography		HHS score		HOOS score	
		Estimated marginal mean difference (95% confidence interval)	Significance	Estimated marginal mean difference (95% confidence interval)	Significance
Age	< 55	83.57(80.32–86.82)	*F*(2,192) = 3.207 *P* = 0.046	249.81(191.43–308.18)	*F*(2,192) = 0.029 *P* = 0.972
	55–65	81.45(78.25–84.65)		275.93(210.23–341.63)	
	> 65	78.02(74.67–81.37)		241.91(184.164–299.66)	
Sex	Male	82.19(79.25–85.13)	*F*(1,193) = 3.207 *P* = 0.046	261.28(209.06–313.50)	*F*(1,193) = 0.133 *P* = 0.716
	Female	80.52(78.16–82.89)		250.80(205.24–296.35)	
Race	Malay	81.90(72.23–91.57)	*F*(2,192) = 0.794 *P* = 0.456	228.30(46.80–409.79)	*F*(2,192) = 0.282 *P* = 0.754
	Chinese	81.95(79.91–83.98)		208.55–279.59)	
	Others	78.51(71.81–84.22)		327.90(214.66–441.13)	
BMI	Underweight	83.29(78.59–87.98)	*F*(3,191) = 0.487 *P* = 0.692	181.22(99.15–263.29)	*F*(3,191) = 1.728 *P* = 0.164
	Normal	79.78(75.80–83.76)		218.19(149.51–286.87)	
	Overweight	81.026(77.99–84.06)		289.71(228.02–351.41)	
	Obese	82.02(78.152–85.90)		305.87(234.34–377.40)	
Side of surgery	Right	81.91(79.33–84.48)	*F*(1,193) = 2.854 *P* = 0.095	258.74(211.39–306.09)	*F*(2,192) = 0.024 *P* = 0.878
	Left	80.84(78.01–83.68)		253.92(201.90–305.93)	

**Table 2 (Abstract O77).** Analysis of covariance showing demographic predictors of clinical outcome measures at 1yr versus preoperative

Demography		HHS score		HOOS score	
		Estimated marginal mean difference (95% confidence interval)	Significance	Estimated marginal mean difference (95% confidence interval)	Significance
Age	< 55	86.61(83.27*–*89.95)	*F*(2,192) = 2.962 *P* = 0.087	138.62(79.72*–*197.53)	*F*(2,192) = 1.049 *P* = 0.248
	55*–*65	86.82(83.54*–*90.10)		106.55(40.254*–*172.85)	
	> 65	83.86(80.61*–*87.12)		78.61(20.34*–*136.89)	
Sex	Male	86.89(83.98*–*89.81)	*F*(1,193) = 7.628 *P* = 0.016	101.29(48.60*–*153.99)	*F*(1,193) = 2.552 *P* = 0.115
	Female	84.97(82.41*–*87.54)		121.33(75.36*–*167.30)	
Race	Malay	86.09(77.25*–*94.93)	*F*(2,192) = 1.141 *P* = 0.349	110.59(-72.56*–*293.74)	*F*(2,192) = 0.996 *P* = 0.383
	Chinese	85.28(83.19*–*87.37)		90.92(55.08*–*126.76)	
	Others	88.51(82.48*–*94.55)		204.60(90.34*–*318.87)	
BMI	Underweight	88.53(82.94*–*94.12)	*F*(3,191) = 1.423 *P* = 0.281	51.82(-30.998*–*134.64)	*F*(3,191) = 0.497 *P* = 0.685
	Normal	81.16(76.02*–*86.31)		80.79(11.49*–*150.09)	
	Overweight	86.60(83.77*–*89.43)		124. 15(61.89*–*186.41)	
	Obese	86.25(81.95*–*90.56)		161.67(89.49*–*233.85)	
Side of surgery	Right	85.14(82.75*–*87.53)	*F*(1,193) = 0.014 *P* = 0.906	115.50(67.73*–*163.28)	*F*(1,193) = 0.009 *P* = 0.926

## P1 Rethinking osteonecrosis of the femoral head: inflammatory cell death in an immuno-metabolic framework

### Tong-jie Yang^1^, Tian-xin Chen^1^, Peng-peng Wen^2^, Hai-jun He^1^

#### ^1^Wangjing Hospital, China Academy of Chinese Medical Sciences, Beijing, China; ^2^First Affiliated Hospital of Wenzhou Medical University, Wenzhou, China

##### **Correspondence:** Hai-jun He (drhjhe@126.com)

*Arthroplasty 2026*, **8(1):**P1

**Background**

Osteonecrosis of the femoral head (ONFH) has traditionally been attributed to impaired blood supply. However, recent evidence indicates that immune and metabolic dysregulation—particularly inflammatory cell death—plays a central role in ONFH progression. This study proposes a paradigm shift: Viewing ONFH as an immuno-metabolic disease driven by pyroptosis, necroptosis, and ferroptosis, linking systemic risk factors (e.g., corticosteroids, oxidative stress) with local immune dysfunction.

**Methods**

This perspective integrates findings from histopathological, molecular, and in vivo studies. Evidence of inflammatory cell death was gathered through analysis of femoral head tissue, gene expression profiling, and rodent models of steroid-induced ONFH. Key pathways (NLRP3 inflammasome, RIPK3/MLKL necrosome, and ferroptosis regulators such as SIRT3 and GPX4) were examined.

**Results**

ONFH lesions are not immunologically silent but show infiltration of M1 macrophages, neutrophils forming extracellular traps, and dysregulated adaptive immune responses. Pyroptosis is activated via caspase-1 and gasdermin D in bone marrow stromal cells under glucocorticoid stress. Necroptosis, mediated by RIPK1/RIPK3/MLKL, damages bone microvascular endothelial cells, exacerbating ischemia. Ferroptosis occurs in osteoblasts with mitochondrial dysfunction and lipid peroxidation, and inhibition of this process by antioxidants (e.g., isovitexin, melatonin) preserved bone integrity. These processes are interlinked, forming a self-perpetuating loop of inflammation and cell loss.

**Conclusions**

We propose that inflammatory cell death is the core driver of ONFH pathology—a shift from a purely vascular hypothesis to an immuno-metabolic framework. This model explains persistent inflammation despite revascularization and opens new avenues for early diagnosis and intervention. Targeting pyroptosis, necroptosis, and ferroptosis may provide novel therapeutic strategies beyond structural repair.

## P2 Effects of closed kinetic chain exercise on muscle strength, balance, range of motion, and gait in older patients after total knee arthroplasty

### Jae Ang Sim^1^, Sang Jin Lee^1^, Kyung Jin Kim^1^, Kyeong Sik Kong^1^, Moon Hee Kim^2^, Byung Hoon Lee^1^

#### ^1^Department of Orthopaedics Surgery, Gachon University College of Medicine, Incheon, Republic of Korea; ^2^Department of Health Sciences & Technology, Gachon University College of Medicine, Incheon, Republic of Korea

##### **Correspondence:** Byung Hoon Lee (oselite@naver.com)

*Arthroplasty 2026*, **8(1):**P2

**Background**

Rehabilitation following total knee arthroplasty (TKA) is essential for optimizing functional outcomes. This study evaluated the effects of a 12-week closed kinetic chain exercise program compared to a combined kinetic chain exercise program on muscle strength, balance, range of motion (ROM), gait, knee function, and pain in older female patients after TKA.

**Methods**

Thirty-four patients who underwent TKA were allocated to three groups: closed kinetic chain exercise group (CKCG, *n* = 12), comparison group (COMG, *n* = 11), and control group (CG, *n* = 11). Knee extensor and flexor strength were measured using a portable muscle strength meter. Static balance was assessed using the single-leg standing test, and dynamic balance was measured using the Timed Up and Go test. Gait performance was evaluated with the 6-min Walk Test. Knee function was assessed using the Western Ontario and McMaster Universities Osteoarthritis Index (WOMAC), and pain was measured using the Visual Analog Scale (VAS). All measurements were taken before and after the 12-week intervention period.

**Results**

Significant increases in knee extensor strength were observed over time in both exercise groups. However, no statistically significant differences were found between groups. Knee flexor strength did not show significant changes. Static balance abilities showed no significant differences between groups or over time. Dynamic balance improved significantly over time but did not differ between groups. ROM and gait performance improved significantly over time without significant between-group differences. WOMAC scores and VAS pain scores did not show statistically significant differences between groups or over time.

**Conclusions**

Closed kinetic chain and comparison exercise programs both improved knee extensor strength, dynamic balance, ROM, and gait in older women after TKA. However, due to the absence of significant between-group differences in several outcomes, future studies should aim to control for baseline group heterogeneity and consider refining exercise protocols.

## P3 Senescence-driven osteonecrosis of the femoral head in the elderly: a distinct pathophysiological entity

### Tong-jie Yang^1^, Tian-xin Chen^1^, Peng-peng Wen^2^, Hai-jun He^1^

#### ^1^Wangjing Hospital, China Academy of Chinese Medical Sciences, Beijing, China; ^2^First Affiliated Hospital of Wenzhou Medical University, Wenzhou, China

##### **Correspondence:** Hai-jun He (drhjhe@126.com)

*Arthroplasty 2026*, **8(1):**P3

The full article of this study has been published online. Please refer to the full text at: 10.3389/fmed.2025.1653037.

## P4 Safety of tenofovir disoproxil fumarate (TDF): a data mining analysis based on the FAERS database

### Wenzhi Wang, Xianzuo Zhang, Chen Zhu

#### Department of Orthopedics, Centre for Leading Medicine and Advanced Technologies of IHM, The First Affiliated Hospital of USTC, Division of Life Sciences and Medicine, University of Science and Technology of China, Hefei, China

##### **Correspondence:** Wenzhi Wang (wangwenzhi@mail.ustc.edu.cn)

*Arthroplasty 2026*, **8(1):**P4

**Background**

Tenofovir Disoproxil Fumarate (TDF), an anti-HIV drug approved by the FDA in 2004, has been widely used in clinical practice. However, long-term use of TDF is associated with potential adverse effects such as renal and bone toxicity. Current safety studies of TDF are mostly based on individual cases or clinical trials with relatively small samples, making it difficult to comprehensively evaluate its safety in the real world. This study aims to explore the potential adverse reaction signals of TDF and evaluate its safety through data mining analysis of the FDA Adverse Event Reporting System (FAERS).

**Methods**

An observational, retrospective disproportionality analysis was employed. Data from the FAERS database from 2004 to 2023 were downloaded and imported into MySQL software for deduplication. Data with the generic name TDF and the trade name Emtricitabine, where the drug role was the primary suspect drug, were selected. Adverse events were classified using MedDRA 26.0, and the clinical characteristics of the reports were described. Four algorithms, namely the Reporting Odds Ratio (ROR), Proportional Reporting Ratio (PRR), Bayesian Confidence Propagation Neural Network (BCPNN), and Multi-Item Gamma Poisson Shrinker (MGPS), were used to quantify the adverse reaction signals. Serious and non-serious reports were compared, and data were analyzed using the chi-squared test, Fisher's exact test, and the Mann–Whitney U test.

**Results**

A total of 16,800,135 adverse event reports were obtained, among which 73,224 reports had TDF as the primary suspect drug, involving 286,984 Preferred Terms (PTs) (Fig. 1). Adverse events were more common in men, which was consistent with the epidemiology of HIV. At the System Organ Class (SOC) level, strong signals were found in renal and urinary disorders, musculoskeletal and connective tissue disorders, etc. Common adverse events such as decreased bone density, osteonecrosis, bone loss, tooth loss, multiple fractures, renal injury, and skeletal injury were consistent with the drug label. The most common indications for TDF use were HIV patients (78.03%), followed by HBV patients (6.07%).

**Conclusions**

Through the analysis of FAERS data, multiple post-marketing safety signals of TDF were identified. Some of these signals were similar to those in clinical trials, while others require further regulatory investigation to determine their significance. Healthcare providers should pay attention to serious adverse reactions to adjust drug selection and monitor patients accordingly. More studies are needed in the future to clarify the safety of TDF.

**Fig. 1 (Abstract P4).**
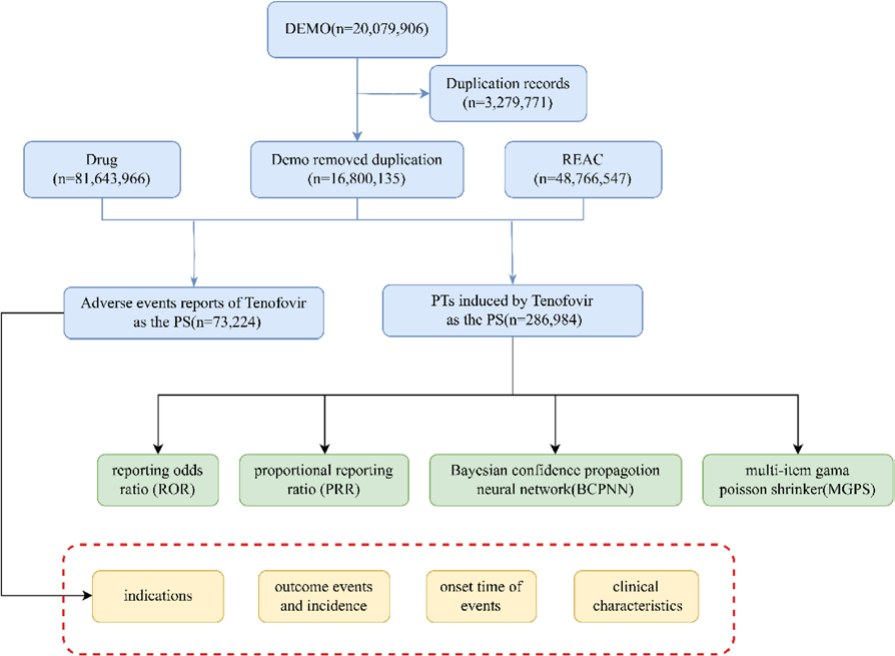
The flow diagram of selecting Tenofovir-related AEs from the FAES database

## P5 Simple, convenient, and economical method to treat femoral medullary shaft infection in prosthetic joint infection using calcium sulfate beads (stimulant Rapid Cure) with available instruments in a healthcare setup

### Rui Lin Ki, Hoe Chin Liew

#### Hospital Sungai Buloh, Selangor, Malaysia

##### **Correspondence:** Ki Rui Lin (drkiruilin@gmail.com)

*Arthroplasty 2026*, **8(1):**P5

**Background**

Calcium sulfate beads (STIMULAN® Rapid Cure; Biocomposites, UK) are widely used in the management of prosthetic joint infections and osteomyelitis. Commercially available instruments, such as the Stimulan bullet mat and the introducer, can facilitate the molding and insertion of the calcium sulfate into the femoral canal. Nevertheless, these tools increase the overall treatment cost. In this report, we describe a simple, convenient, and economical technique to mold calcium sulfate into a long tubular form using standard syringes, allowing easy insertion and reliable antibiotic delivery without the need for specialized equipment.

**Methods**

We demonstrate a technique for shaping calcium sulfate into elongated cylindrical forms using 5 cc syringes. This alternative approach produces longer molds compared to the commercially available molds, which typically generate small pellets (3–6 mm in size). A mixture of 3 g of vancomycin and 240 mg of gentamicin (in 6 mL solution) was incorporated into 20 cc of calcium sulfate. The mixture was packed into the syringes to form cylindrical rods. Intraoperative images document the procedure. Two surgical drains were placed outside the capsule and above the fascia for third-space management, and the wound closure was augmented with vacuum-assisted closure therapy (Fig. 1).

**Results**

The patient recovered well with successful infection clearance and complete wound healing. At one-year follow-up, the patient was able to bear weight on the affected limb.

**Conclusions**

This syringe-based technique for molding calcium sulfate is a cost-saving and practical alternative to the commercially available introducer system. It provides a viable and practical solution for effective antibiotic delivery in femoral canal infections, particularly in resource-limited healthcare settings.

**Fig. 1 (Abstract P5).**
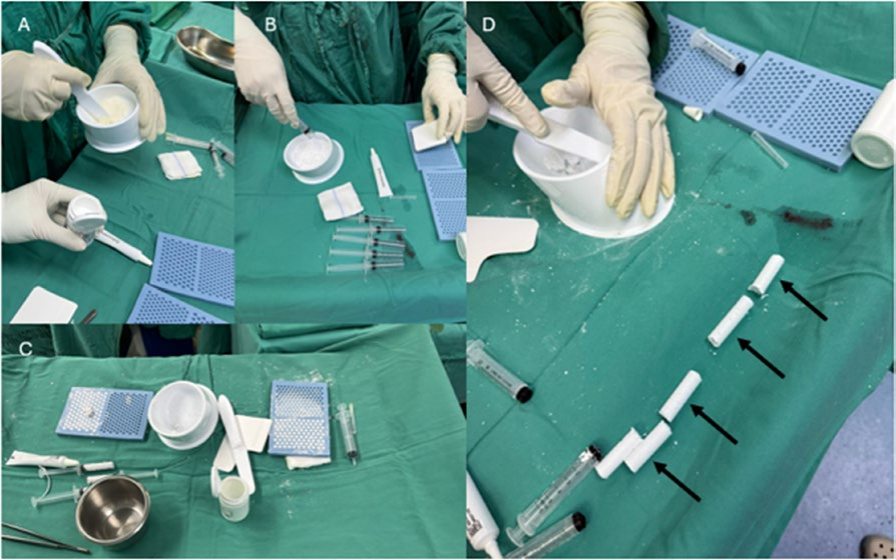
The figure above shows the Step-by-step process for creating calcium sulfate antibiotic-loaded rods: **A** A paste is prepared by mixing 3 g of vancomycin powder and 240 mg of gentamicin in 6 mL of solution, followed by the addition of 20 cc of calcium sulfate powder; **B** The solvent (sterile water) is then slowly added in small amounts and thoroughly mixed to form a uniform paste; **C** The paste is then molded into cylindrical rods using 5 cc syringes; **D** The final product consists of short rods of antibiotic-loaded calcium sulfate, ready for implantation (marked by black arrow)

## P6 Surgical efficacy and clinical outcome of robotic total knee arthroplasty at high altitude

### Li Xiong^1,6^, Myima Tashi^2^, Tingfang Yan^4^, Lobsang Choephel^3^, Jun Zhang^1^, Zhonghua Xu^1^, Cheng Yu^2^, Tianfei Ran^1^, Max Q.-H. Meng^4,5^, Tashi Norbu^3^, Yuan Zhang^1,2^

#### ^1^Joint Disease & Sport Medicine Center, Department of Orthopedics, Xinqiao Hospital, Army Medical University, Chongqing, China; ^2^Department of Orthopedics, 953 Hospital of PLA, Xigaze, Tibet, China; ^3^Department of Orthopedics, People’s Hospital of Lhasa, Tibet, China; ^4^Yuanhua Robotics & Perception AI Ltd.; Shenzhen, China; ^5^Department of Electronic and Electrical Engineering, Southern University of Science and Technology; Shenzhen, China; ^6^Department of Orthopedics, General Hospital of Tibet Military Region; Lhasa, Tibet, China

##### **Correspondence:** Yuan Zhang (zhangyuan@tmmu.edu.cn); Tashi Norbu (luosong0708@126.com)

*Arthroplasty 2026*, **8(1):**P6

>**Background**

High-altitude settings pose multiple challenges that may affect surgical implementation and postoperative recovery. Although robotic total knee arthroplasty (TKA) is advantageous in precise execution and favorable results at low altitudes, no prior study has reported on robotic TKA in high-altitude regions.

**Methods**

This prospective, comparative clinical study aimed to assess whether a surgical robot could improve surgical efficacy and clinical outcomes for robotic TKA in the high-altitude region of Tibet (3,900 m above sea level).

**Results**

The surgical robot exhibited environmental robustness as evidenced by anticipative program execution speed and alignment errors (*P* = 0.66 and 0.87). We then investigated the surgical efficacy and clinical outcome for a comprehensive evaluation. As compared to conventional manual TKA in the same region, the time for bone-cutting and gap-balancing, as well as bone-cutting errors, were significantly reduced with robotic TKA (*P* = 0.02, 0.00, and 0.00). This led to significantly improved changes of patient-reported outcome measures (PROMs) such as the Knee Society Score and SF-12-Physical Component Summary (PCS). When compared to robotic TKA in a low-altitude setting (Chongqing, 300 m), we observed significantly higher patient satisfaction and PROMs in terms of the Western Ontario and McMaster University Osteoarthritis Index and SF-12-PCS at high altitude. However, there were drawbacks such as prolonged operation times (by 13.50 min), increased blood loss (by 219.7 mL), and higher complication and readmission rates (by 37.0% and 20.0%, respectively) (*P* = 0.02, 0.02, 0.00, and 0.03).

**Conclusions**

A surgical robot can achieve desirable efficacy and outcome for TKA at high altitudes. However, concerns remain regarding increased blood loss, higher complications, and elevated readmission rates.

## P7 Development of rehabilitation norms for total knee arthroplasty patients: linking hospitals and communities through exercise function evaluation

### Byung Hoon Lee^1^, Moon Hee Kim^2^, Kyeong Sik Kong^1^, Kyung Jin Kim^1^, Sang Jin Lee^1^, Jae Ang Sim^1^

#### ^1^Department of Orthopaedics Surgery, Gachon University College of Medicine, Incheon, Republic of Korea; ^2^Department of Health Sciences & Technology, Gachon University College of Medicine, Incheon, Republic of Korea

##### **Correspondence:** Jae Ang Sim (sim_ja@gilhospital.com)

*Arthroplasty 2026*, **8(1):**P7

**Background**

Exercise rehabilitation immediately after total knee arthroplasty (TKA) is essential to prevent postoperative physical function decline and promote recovery. Despite the benefits of TKA, recovery outcomes vary significantly among patients. This study aimed to establish standardized evaluation norms for linking hospital-based rehabilitation with community care.

**Methods**

This study included 824 patients (164 men and 660 women; age: 72 ± 6.4 years) with a history of TKA. Participants were categorized based on the postoperative period (immediately after discharge to over 49 months). Physical function indicators such as muscular strength, endurance, flexibility, cardiorespiratory endurance, balance, and gait were measured. Quality of life was assessed using the WOMAC and SF-36 questionnaires. Data analysis was performed using SPSS software (version 21.0), employing descriptive statistics and one-way ANOVA to identify sex-based differences.

**Results**

There was no significant difference in the Visual Analogue Scale (VAS) according to sex; however, body composition (weight, skeletal muscle mass, body fat weight, and body fat percentage) showed significant differences between men and women. Additionally, knee flexion contracture range of motion (ROM), 6-min walking, sit-and-reach flexibility, muscle strength (flexion and extension), and Timed Up and Go (TUG) test results (Tables 1&2) showed significant differences. Using the means and standard deviations of these indicators, Cajori’s 5-grade evaluation norms were established to provide standardized rehabilitation guidelines linking hospital-based care with community-based rehabilitation services (Table 3).

**Conclusions**

The findings highlight the importance of early and tailored rehabilitation programs to address individualized physical fitness needs post-TKA. The Cajori 5-grade evaluation norms provide measurable standards for assessing recovery progress and optimizing rehabilitation strategies.

**Table 1 (Abstract P7).** Differences in VAS and body composition according to sex

	VAS	Weight (kg)	Skeletal muscle mass (kg)	Fat weight (kg)	%fat (%)	BMI kg⦁m^2^
Sex Men	3.5 ± 2.7	68.9 ± 10.2	30.6 ± 3.4	19.1 ± 6.7	27.2 ± 6.3	24.8 ± 3.9
Women	3.8 ± 2.4	60.9 ± 9.2	20.4 ± 2.6	22.4 ± 6.5	36.2 ± 6.1	25.4 ± 3.3
F value	1.410_(1,404)_	52.343_(1,404)_^***^	27.101_(1,400)_^***^	17.862_(1,396)_^***^	154.778_(1,400)_^***^	1.749_(1,406)_
Significance	0.236	0.001	0.001	0.001	0.000	0.187

*BMI* body mass index

^***^Significant difference (*P* < 0.001)

**Table 2 (Abstract P7).** Differences in exercise rehabilitation-related variables according to sex

Sex Variables		Men	Women	Total	F value	*p* value
ROM	Knee maximum flexion (°)	94.25 ± 20.25	89.65 ± 19.87	90.58 ± 20.17	6.764	0.009**
	Knee flexion contracture (°)	1.01 ± 2.84	0.45 ± 2.02	0.56 ± 2.22	8.625	0.003**
Muscle Strength	Knee flexion (kg)	14.76 ± 5.20	10.74 ± 3.95	11.55 ± 4.52	117.297	0.000***
	Knee extension (kg)	19.11 ± 7.83	13.40 ± 5.00	14.54 ± 6.11	131.518	0.000***
Muscular endurance	30 s stand up from a chair (frequency)	13.08 ± 4.87	12.09 ± 4.07	12.27 ± 4.25	5.350	0.021*
Flexibility	Sit and reach (cm)	5.56 ± 8.48	12.05 ± 8.30	10.78 ± 8.72	76.456	0.000***
Cardiopulmonary endurance	6 min walking (s)	411.71 ± 123.09	378.83 ± 125.41	385.46 ± 125.56	9.011	0.003**
Gait	Single support time (%)	36.17 ± 5.79	37.18 ± 4.29	36.97 ± 4.65	5.841	0.016*
Balance	Single-leg stance test(sec)	12.06 ± 13.79	15.82 ± 21.54	15.05 ± 20.25	4.438	0.035*
	TUG (s)	8.89 ± 3.98	10.39 ± 6.94	10.09 ± 6.94	6.964	0.008**
Quality of life	SF36 V2	55.25 ± 19.16	54.47 ± 19.53	54.61 ± 19.45	0.168	0.682
	WOMAC	28.15 ± 18.93	28.96 ± 18.91	28.81 ± 18.90	0.232	0.630

*Significant difference (*P* < 0.05)

**Significant difference (*P* < 0.01)

***Significant difference (*P* < 0.001)

**Table 3 (Abstract P7).** Norms of variables related to exercise rehabilitation function

Variables			Excellent	Very Good	Good	Poor	Very Poor
ROM	Knee maximum flexion (°)	Men	122.3 ≤	101.2–122.2	79.7–101.1	58.5–79.6	58.4 ≥
		Women	120.4 ≤	98.7–120.3	76.9–98.6	55.2–76.8	55.1 ≥
	Knee flexion contracture (°)		1.2 ≥	1.3–1.7	1.8–2.5	2.6–2.9	3 ≤
Muscle Strength	Knee flexion (kg)	Men	21.2 ≤	16.0–21.1	10.5–15.9	5.4–10.4	5.3 ≥
		Women	15.1 ≤	11.3–15.0	7.4–11.2	3.6– 7.3	3.5 ≥
	Knee extension (kg)	Men	36.4 ≤	28.2–36.3	19.8–28.1	11.6–19.7	11.5 ≥
		Women	24.9 ≤	19.1–24.8	13.3–19.0	7.5–13.2	7.4 ≥
Muscular Endurance	30 s stand up from a chair (N)		19 ≤	15–18	10–14	5–9	4 ≥
Flexibility	Sit and reach (cm)	Men	17.0 ≤	8.4–16.9	0.2–8.3	-8.7–0.1	-8.8 ≥
		Women	24.4 ≤	15.6–24.3	6.7–15.5	-2.09–6.6	-2.10 ≥
Cardiopulmonary function	6 min walking (m)	Men	581.9 ≤	460.8–581.8	339.5–460.7	218.4–339.4	218.3 ≥
		Women	551.2 ≤	424.1–551.1	296.8–424.0	169.6–296.7	169.5 ≥
Gait	Single support time (%)		45.0 ≤	39.1–44.9	33.0–39.0	27.0–32.9	26.9 ≥
Balance	Single-leg stance test (s)	Men	36.0 ≤	20.9–35.9	5.7–20.8	1.0–5.6	0.9 ≥
		Women	50.6 ≤	27.1–50.5	3.5–27.0	1.0–3.4	0.9 ≥
	TUG (s)	Men	4.8 ≥	4.9–6.7	6.8–11.3	11.4–15.8	15.9 ≤
		Women	4.7 ≥	4.8–6.4	6.5–15.6	15.7–24.6	24.7 ≤
Quality of life	SF36 (score)		89 ≤	69–88	48–68	27–47	26 ≥
	WOMAC (score)		0	1–15	16–38	39–59	60 ≤

## P8 Hip pain 10 years after total hip replacement

### Ong Lik Han^1^, Sabaruddin Bin Abdullah^2^, Ankimtay Anak Rutel^2^, Reuben Prashant Rao^2^, Ahmad Redzuan Arshad^2^, Elsonmond Vick Duin^2 3^, Ang Xi Yuan^1^, Faris Kamaruddin^2^

#### ^1^Universiti Malaysia Sarawak (UNIMAS), Sarawak, Malaysia; ^2^Sarawak General Hospital, Sarawak, Malaysia; ^3^Universiti Malaysia Sabah (UMS), Sabah, Malaysia

##### **Correspondence:** Ong Lik Han (lhong@unimas.my)

*Arthroplasty 2026*, **8(1):**P8

**Background**

Femoral stem fracture following total hip arthroplasty (THA) is an infrequent but devastating complication with an incidence of 0.1 to 3.4%. Loss of proximal support with a well-fixed distal stem causes cantilever bending that later promotes localized corrosion, fretting, and fatigue crack initiation, leading to stem failure.

**Report**

An 80-year-old man with a history of bilateral THR in 2014 presented with insidious right hip pain for one month. There was no history of trauma, and the patient was able to ambulate with a walking frame. Removal of the broken stem was done using a Burr at the lateral femoral cortex at the tip of the stem and pushed proximally, disengaging from cement grout. V40 femoral stem size 30, length 95 mm, was inserted with a 28 mm femoral head.

**Discussion**

Risk factors for stem fracture can be divided into patient (BMI, activities, bone stock), implant (design, material, modularity, cemented/cementless), and surgical (varus malposition, sizing, cementing technique) factors. Meta-analysis of 49,723 THAs noted a median time frame of 68 months post-op, 61.8 years in age, predominantly male gender, and above 80 kg. It was noted that age below 63 years, varus stem alignment, modular implants, and revision arthroplasty contributed to a statistically significant risk of stem fracture with a risk window of 15 years. Osteoarthritis, rheumatoid arthritis, and avascular necrosis were the main indications of surgery. Even with comparable male-to-female radio, 77% of stem fractures occur in males. In terms of weight, an increased risk was observed since the Charnley era, especially above 88 kg, with an average difference of 23 kg between fractured/ non-fractured groups. The risk is linear and significant between weight and time to fracture. In managing revision THA or osteoporosis, the increased strain due to bone loss, loosening, or trochanteric osteotomies will require a larger diameter implant and proximal bone support to avoid stem fracture. However, this can be technically challenging or not feasible due to the narrow proximal femur of our Asian population. 83% of stem fractures were seen to occur before 10 years, as in this patient, and rarely beyond 15 years, which is consistent with the 11-year “at risk” noted by Wroblewski et al. Patients’ activity level, stem sizing, and cement mantle are more complex measurements in assessing the risk of stem fracture and warrant further studies.

**Conclusions**

Despite the manufacturer reporting a very low (0.0006%) stem fracture rate, identifying risk factors for stem fracture and modifying them where possible forms part of a wider strategy to help reduce the risk of subsequent revision surgery in patients, which will increase cost, as well as result in poorer outcomes when compared to primary THA.

**Consent for publication**

Written informed consent was obtained from the patient for publication of this case report.

## P9 Is a 2-strut allograft better than a 1-strut allograft in periprosthetic total hip replacement fracture?

### Ong Lik Han^1^, Sabaruddin Bin Abdullah^2^, Ankimtay Anak Rutel^2^, Reuben Prashant Rao^2^, Ahmad Redzuan Arshad^2^, Elsonmond Vick Duin^2 3^, Ang Xi Yuan^1^, Faris Kamaruddin^2^

#### ^1^Universiti Malaysia Sarawak (UNIMAS), Sarawak, Malaysia; ^2^Sarawak General Hospital, Sarawak, Malaysia; ^3^Universiti Malaysia Sabah (UMS), Sabah, Malaysia

##### **Correspondence:** Ong Lik Han (lhong@unimas.my)

*Arthroplasty 2026*, **8(1):**P9

**Background**

Periprosthetic hip fracture is a potentially devastating complication following total hip arthroplasty (THA), with high first-year mortality (15–20%). Vancouver B1 generally requires open reduction internal fixation with an implant with or without a strut graft. We report a case of dual strut allograft stabilized with cable tie reconstruction in a chronic kidney disease patient with osteoporosis.

**Report**

A 74-year-old lady with a known case of chronic renal failure with a history of right long stem total hip replacement 20 years ago presented with right thigh pain after a fall. Radiograph noted Vancouver Type B1 periprosthetic fracture of the right femur. Allograft strut graft with cable ties was done.

**Discussion**

Managing periprosthetic femoral fractures requires individualized strategies based on patient comorbidities and bone quality. In this patient, severe osteoporosis and chronic kidney disease made revision arthroplasty (RA) high-risk. While RA is often the standard, fixation is preferable when implant preservation is viable. Although locking plates are commonly recommended, they were not technically feasible in this case. Instead, we opted for stabilization using bone allografts without anatomical reduction. Crucially, we confirmed stem stability intraoperatively, as preoperative radiographs can frequently misclassify loose stems as stable (Vancouver B1). The cortical struts in this dual-allograft construct effectively function as biological plates, offering mechanical stability and potential bone stock enhancement where standard plating or single struts might be insufficient.

**Conclusions**

Cable plate with single cortical strut allografts has been proven to be an effective treatment method; dual strut allograft dual plane cable tie fixation will require further studies/ series to evaluate effectiveness and functional outcome. Therefore, cortical struts essentially represent or can act as biologic bone plates that provide mechanical stability, enhance fracture healing, and can potentially increase bone stock.

**Consent for publication**

Written informed consent was obtained from the patient for publication of this case report.

## P10 Patient-specific versus Oxford microplasty instrumentation in unicompartmental knee arthroplasty

### Qilong Jiang, Yu Deng

#### Department of Orthopaedic Surgery, Chongqing Orthopedic Hospital of Traditional Chinese Medicine, Chongqing, China

##### **Correspondence:** Yu Deng (dengyu1984gkyy@163.com)

*Arthroplasty 2026*, **8(1):**P10

**Background**

The purpose of the study was to compare patient-specific instrumentation (PSI) with Oxford microplasty instrumentation (MPI) in unicompartmental knee arthroplasty (UKA) for patients with anteromedial knee osteoarthritis.

**Methods**

We performed a prospective study at a single high-volume orthopaedic hospital. All patients were randomly assigned to undergo either PSI-assisted UKA performed by inexperienced surgeons (PSI group) (Fig. 1) or MPI-assisted UKA performed by experienced surgeons (MPI group) at a 1:1 ratio. Radiological measurements included the femoral component varus and valgus angle, flexion and extension angle, tibial component varus and valgus angle, tibial posterior slope angle, and hip-knee-ankle angle (HKAA). The Knee Society Score (KSS) and Hospital for Special Surgery (HSS) scores were assessed at one, three, six, and 12 months. Mixed-effects modelling was used to analyze repeated measurements.

**Results**

A total of 73 participants (37 in the PSI group and 36 in the PM group) were enrolled from June 2021 to July 2023. No patients were lost to follow-up. No significant differences between groups were found in the femoral component varus and valgus angle, flexion and extension angle, tibial component varus and valgus angle, tibial posterior slope angle, or hip-knee-ankle angle (all *P* > 0.05) (Table 1). At the 12-month follow-up, the PSI group and MPI group achieved mean KSSs of 93.4 points (95% CI, 88.6–97.8) and 93.6 points (95% CI, 89.1–98.4), respectively (Fig. 2). There were no significant between-group differences in the KSS and HSS score improvements from baseline to each follow-up point.

**Conclusions**

PSI-assisted UKA performed by inexperienced surgeons can yield radiological and functional outcomes comparable to those of MPI-assisted UKA performed by experienced surgeons in the treatment of knee osteoarthritis. PSI is emerging as a promising alternative for practitioners inexperienced at UKA on a learning curve.

**Fig. 1 (Abstract P10).**
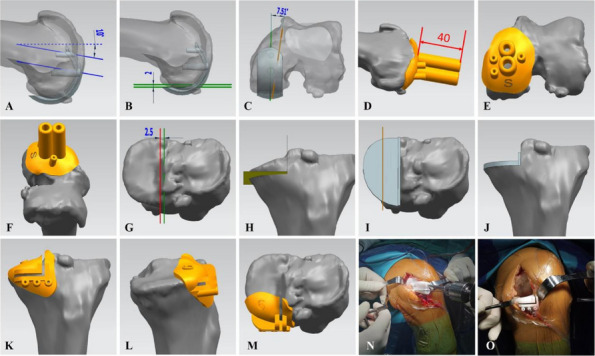
Preoperative planning and definitive cutting block of the femoral PSI. **A**, **B**, **C** Femoral component designs for sizing, alignment, and implant positioning that match the femoral anatomy. **D**, **E**, **F** Femoral PSI 3D images of the definitive position superimposed on the preoperative plan. **G**, **H**, **I**, **J** Tibial component designs for sizing, alignment, and implant positioning that match the tibial anatomy. **K**, **L**, **M** Tibial PSI 3D images of the definitive position superimposed on the preoperative plan. **N**, **O** The definitive cutting block of the femoral and tibial PSI. PSI, patient-specific instrumentation

**Fig. 2 (Abstract P10).**
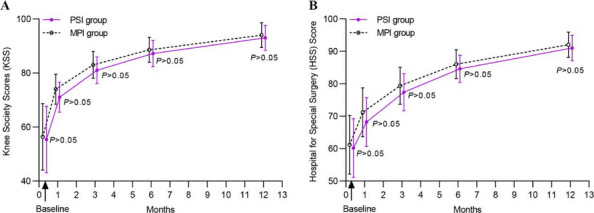
Postoperative measurements of the Knee Society Score (KSS) and Hospital for Special Surgery (HSS) score at baseline and one, three, six, and 12 months post-operatively. The KSS and HSS score range from 0 to 100, with higher scores indicating better functioning. PSI, patient-specific instrumentation. MPI, Oxford microplasty instrumentation

**Table 1 (Abstract P10).** Radiological measurements at post-operative one day

	**PSI group** **(*n* = 37)**	**MPI group** **(*n* = 36)**	**Mean difference**
Femoral component			
Varus/valgus angle	0.88 (− 0.41 to 2.16)	2.47 (1.10 to 3.84)	− 1.59 (− 3.38 to 0.19)
Flexion/extension angle	8.38 (7.76 to 9.01)	9.04 (8.31 to 9.77)	− 0.66 (− 1.56 to 0.25)
Tibial component			
Varus/valgus angle	3.16 (2.57 to 3.76)	4.26 (2.99 to 5.52)	− 1.10 (− 2.41 to 0.22)
Posteroinferior tilt	3.91 (2.26 to 4.57)	4.18 (3.28 to 5.08)	− 0.27 (− 1.33 to 0.78)
Hip-knee-ankle angle	176.25 (175.61 to 176.89)	175.80 (174.91 to 176.70)	0.45 (− 0.59 to 1.49)

## P11 1.5-stage knee revision for PJI: lessons from five cases and the road to refinement

### Jeffrey Chong, Khairul Anwar Ayob, Veenesh Selvaratnam, Loh Kwong Weng, Azlina Amir Abbas

#### University Malaya Medical Centre, Kuala Lumpur, Malaysia

##### **Correspondence:** Jeffrey Chong (jcmanutd1990@hotmail.com)

*Arthroplasty 2026*, **8(1):**P11

**Background**

Periprosthetic joint infection (PJI) is a challenging complication following knee arthroplasty. Traditional two-stage revision remains the gold standard with high success rates, albeit with significant patient morbidity, functional decline, and multiple surgeries. A hybrid strategy—known as the 1.5-stage revision, a novel approach, uses a robust, antibiotic-loaded articulating spacer meant for prolonged service with the possibility for conversion to a second-stage revision in cases of reinfection or failure. This case series evaluates the outcomes of five patients treated with a 1.5-stage revision technique for persistent PJI at our centre.

**Methods**

Between 2021 and 2024, five patients with confirmed knee PJI underwent a 1.5-stage revision procedure at our centre (Fig. 1). Preoperative evaluation encompasses a comprehensive surgical history, including prior implant selection, perioperative complications, medical comorbidities, radiographic analysis, physical examination, and diagnostic evaluation. All of the patients developed symptoms more than 3 months after the index procedure. Knee aspirations were performed to get samples, and no antibiotics were started before revision surgeries. Preoperative radiographic evaluation involves weight-bearing anteroposterior, lateral, and sunrise radiographs to look at radiolucencies, bone loss, constraint levels, and appropriate implant selection. CT scans were not employed routinely. Physical examination encompasses an inspection of skin integrity, sinus tracts, scars, erythema, swelling, gait analysis, passive and active range of motion, flexion contracture, flexion–extension instability, and varus-valgus laxity or deformity. Intraoperative protocol:

- **Surgical Debridement:** Removal of old scar, infected synovium, devitalised tissue, old cement, and implants followed by canal preparation.
- **Spacer Construction:** Handcrafted spacers fashioned from primary femoral components, all-poly tibial components, or inserts with cemented dowels created by hand-coating Steinmann pins with antibiotic-laden bone cement. Stems were molded using a syringe, placed around the stem, and allowed to harden on the back table.
- **Cement Implantation:** Cementing metaphyseal defects, providing rotational stability, and facilitating future surgeries if necessary.
- **Postoperative Management:** Broad-spectrum antibiotics, transitioned to culture-specific regimens, serial radiographs, and inflammatory markers. Functional outcomes were assessed using KOOS.

**Results**

Of the five patients treated with 1.5-stage revision, four patients achieved infection-free outcomes and retained their spacers with satisfactory functional recovery, with KOOS scores ranging from 75 to 94. One patient developed reinfection, requiring conversion to a first-stage revision, underscoring the importance of careful patient selection. Overall, this technique has shown excellent short-term outcomes and promising midterm results in our centre.

**Conclusion**

The 1.5-stage revision method offers several advantages, namely fewer surgeries, reduced morbidity, earlier mobility, and potential cost savings by avoiding a second major surgery. Reinfection remains a concern, especially in high-risk patients. Careful patient selection, meticulous surgical technique, and long-term follow-up are needed to refine patient selection criteria and improve the efficacy of this emerging technique.

**Fig. 1 (Abstract P11).**
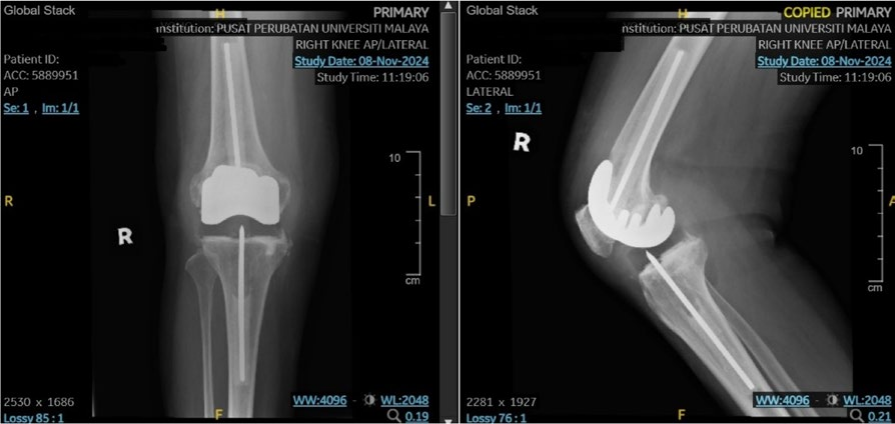
Postoperative radiographs of a patient post-explantation and placement of an articulating spacer for PJI

## P12 Three-dimensional acetabular containment surgery improves femoral head morphology and clinical outcomes in older children with Legg-Calvé-Perthes disease

### Ningtao Ren

#### 4th Medical Center, PLAGH, Beijing, China

##### **Correspondence:** Ningtao Ren (ningtaoren@163.com)

*Arthroplasty 2026*, **8(1):**P12

**Background**

Legg-Calvé-Perthes disease (LCPD) in older children (≥ 6 years) presents unique therapeutic challenges due to limited remodeling potential and high risk of permanent deformity. While various surgical interventions exist, the optimal treatment strategy for patients with substantial femoral head subluxation remains controversial. Traditional containment procedures often fail to provide adequate three-dimensional coverage when the femoral epiphyseal extrusion index exceeds 20%.

**Questions**

We asked: (1) Can triple pelvic osteotomy and periacetabular osteotomy provide three-dimensional improvement in acetabular coverage for older LCPD patients with high femoral epiphyseal extrusion index? (2) How does the morphology of the acetabulum and femoral head evolve postoperatively? (3) What are the clinical and functional outcomes following these procedures?

**Methods**

We retrospectively reviewed 10 patients (10 hips; mean age, 9.12 years; range, 6–13 years) who underwent either triple pelvic osteotomy (*n* = 7) or periacetabular osteotomy (*n* = 3) between 2015 and 2023. All patients had femoral epiphyseal extrusion index > 20% and minimum follow-up of 12 months (mean, 60.1 months; range, 15.6–105.8 months). We assessed three-dimensional coverage using lateral center–edge angle (LCEA), anterior center–edge angle (ACEA), Tönnis angle, and femoral epiphyseal extrusion index. Femoral head morphology was evaluated using the modified sphericity deviation score and Stulberg classification. Clinical outcomes were measured using the modified Harris Hip Score and McKay classification.

**Results**

Both procedures significantly improved three-dimensional coverage: LCEA increased from 10.8° ± 1.8° to 36.1° ± 3.2° (*P* < 0.001), ACEA from 25.7° ± 2.8° to 39.9° ± 3.8° (*P* = 0.008), and femoral epiphyseal extrusion index decreased from 29.9% ± 3.0% to 5.3% ± 3.3% (*P* = 0.001). The mean postoperative modified sphericity deviation score was 8.5% (range, 0–30.5%). At final follow-up, 9 hips (90%) achieved Stulberg I/II classification. The modified Harris Hip Score improved from 79.0 ± 2.4 to 95.1 ± 1.2 (*P* < 0.001), with 80% of patients achieving excellent McKay functional outcomes. Multivariate analysis revealed significant correlations between postoperative sphericity and preoperative Herring classification (*P* = 0.035), postoperative LCEA (*P* = 0.05), ACEA (*P* = 0.003), and BMI (*P* = 0.042).

## P13 3D printed metal augment or one⁃piece acetabular prosthesis for reconstruction of bone defects in hip revision

### Jinliang Wang, Bo Sun, Wei Mei, Qingde Wang, Shaohua Wang, Xuan Wei

#### Zhengzhou Orthopaedic Hospital, Zhengzhou, China

##### **Correspondence:** Jinliang Wang (jiajiawaers@126.com)

*Arthroplasty 2026*, **8(1):**P13

**Background**

To investigate the clinical efficacy of 3D printed metal augment or one⁃piece acetabular prosthesis for reconstruction of bone defects in hip revision.

**Methods**

A total of 11 patients with hip revision who were admitted to the Joint Disease Department of Zhengzhou Orthopedics Hospital with bone defects from June 2020 to January 2023 were retrospectively analyzed, 3D printed metal augment (9 cases), one-piece acetabular prosthesis (2 cases). The time from initial replacement to revision is an average of 11.8 years. Recording clinical efficacy, complications, and imaging results.

**Results**

The operation time is 110–159 min, with an average of 145 min. The intraoperative bleeding volume was 680–1450 mL, with an average of 950 mL. The follow-up time was an average of 21 months (15–31 months). At the last follow-up, the Harris score of the hip joint was (74.34 ± 11.39) points, which was significantly higher than preoperative points (32.23 ± 7.69) (*P* = 0.000); The VAS score for pain was(1.77 ± 0.91) points, significantly lower than preoperative (6.13 ± 1.58 points) (*P* = 0.000); Leg Length Discrepancy (LLD) was (0.69 ± 0.52) cm, which was significantly reduced compared to preoperative (1.97 ± 0.71 cm) (*P* = 0.000). The vertical position of the center of affected rotation (VCOR) was (1.88 ± 0.46) cm, showing significant differences when compared with those before operation (3.48 ± 0.78 cm) (*P* = 0.000), showing no difference when compared with those of a healthy hip (1.67 ± 0.35 cm) (*P* = 0.242). The horizontal position of the affected rotation center (HCOR) was (3.48 ± 0.55) cm, showing no difference when compared with those of the healthy hip (3.54 ± 0.32 cm) and those before operation (3.35 ± 0.42 cm) (*P* > 0.05). Complications: intraoperative fracture of the greater trochanter (1 case), heterotopic ossification (1 case). There were no complications such as infection, prosthesis loosening, dislocation, or nerve injury.

**Conclusions**

In hip revision, a 3D printed metal augment or one-piece acetabular prosthesis can reconstruct the acetabular bone defect, optimize and simplify surgical procedures, personalize matching of acetabular bone defects, and reconstruct the relatively normal rotation center of the hip joint. The short-term clinical effect is satisfactory; the long-term effectiveness needs further follow-up.

## P14 Impaction bone graft (IBG) tibia for right knee post-traumatic osteoarthritis with large medial defect (AORI type 3)

### Wei Cheong Eu^1^, Shengqun Zhang^2^, Hui Li ^2^, Shuo Jie^2^, Xianzhe Huang^2^, Xiaoxin Wu^2^, Xinzhan Mao^2^

#### ^1^Hospital Kuala Lumpur, Malaysia; ^2^The Second Xiangya Hospital, Central South University, Changsha, China

##### **Correspondence:** Wei Cheong Eu (euweicheong@gmail.com)

*Arthroplasty 2026*, **8(1):**P14

**Background**

Bone defects in knee arthroplasty should be addressed meticulously to prevent the risk of early loosening and failure. There are two ways, namely biological and non-biological methods, for tackling bone defects. The current trend has shifted towards the use of metal substitutes, including augment, cone, sleeve, or 3D-printed customised implant. Here, we describe a case of IBG, the biological way to reconstruct a bone defect.

**Case Report**

A 45-year-old female with the chief complaint of right knee pain for more than 6 years. She had a history of an open right knee fracture 6 years ago and underwent multiple surgeries, including debridement, external fixator, and flap repair surgery. She sustained an open distal femur fracture and a comminuted right medial tibia plateau fracture. Clinically, her right knee is stiff with limited ROM of 30–45 degrees. There are multiple scars with a large skin flap on the anteromedial aspect of the right knee. CT scan shows AORI Type F2A, T3A defect with 3 cm bone loss in the medial tibia plateau and Hoffa fracture right medial femoral condyle with a defect of 1 cm. In MRI, the MCL was avulsed at the medial tibia. Incision was made following the most lateral margin of the previous skin flap and undermined to follow the extensile medial parapatellar approach. Quadricepsplasty was performed, and scar tissue was removed from the anterior femur. The bone-cut graft was used to reconstruct the postero-medial femoral condyle. The tibia trial implant with a long stem, one size bigger in diameter, was kept in place. The metal mesh was shaped to reconstruct the proximal-medial cortical rim. Morselized bone grafts were impacted to reconstruct the metaphysis and epiphysis region, converting this into a contained defect. Finer grafts (< 5 mm) were packed first, followed by the bigger grafts (5–7 mm) proximally. Cement was pressurized, and a hinge knee implant was inserted.

**Discussion**

There were four main challenges in this case. First, the large and uncontained medial tibia defect without a cortical rim. Second, exposure to the stiff and limited motion of the knee. Third, skin incision in a case with multiple scars and the presence of a skin flap. And lastly, the loss of MCL. There are a few options in managing AORI type 3 defect, which are either a metal cone or metaphyseal sleeve, a structural allograft, or a customized megaprosthesis. Considering the young age, the IBG method was selected to reconstruct the proximal-medial defect. Preserving the bone stock is important in the anticipation of future revision. IBG is time-consuming and technically demanding. Multiple studies have shown its durability in restoring bone stock, with no mechanical failure. A long stem with adequate rim support restored by IBG is associated with adequate stability and long-term survival. Formation of the neocortex is expected in previously defective areas filled by grafts. Histological analysis had shown active new osteoid formation in allograft areas with vascular stroma seen between bone fragments.

**Conclusion**

The Impaction Bone Graft Tibia technique is technically demanding but safe and important in preserving bone stock, especially in younger patients.

**Consent for publication**

Written informed consent was obtained from the patient for publication of this case report.

## P15 Learning curve of THA using anterior minimally invasive surgery (AMIS) approach and evaluation of outcomes with bikini incision

### Hironori Unno^1^, Takahiro Hasegawa^1^, Shinya Takigawa^1^, Masayoshi Sato^1^, Masahiro Hasegawa^2^

#### ^1^Department of Orthopaedic Surgery, Iga City General Hospital, Mie, Japan; ^2^Department of Orthopaedic Surgery, Mie University Graduate School of Medicine, Mie, Japan

##### **Correspondence:** Hironori Unno (hironori08071984@gmail.com)

*Arthroplasty 2026*, **8(1):**P15

**Background**

The anterior minimally invasive surgical (AMIS) approach was designed to minimize the extension of the surgical exposure for THA performance through DAA. AMIS for THA has gained popularity among hip surgeons in recent years; however, surgeons still fear the learning curve, which was estimated to be between 50 and 100 surgeries. Moreover, bikini incision may improve postoperative scar cosmesis and patient satisfaction while reducing wound complications, but the surgical field is poorly developed. In this study, we compared the outcomes of early introduction, late introduction, and bikini incision groups in THA using the AMIS approach.

**Methods**

Patients who underwent surgery for AMIS-THA in our hospital were included in the study. After receiving training, the bikini incision was introduced from the 95th case, and was limited to female patients only and to cases with non-severe deformities. We divided patients into three groups (Group NE: normal incision, early introduction, Group NL: normal incision, late introduction, Group B: bikini incision). Demographic and clinical parameters were retrospectively collected: age, BMI, disease, time of surgery, intraoperative blood loss, incision length, cup alignment (inclination, anteversion), stem alignment (varus, anterior tilt), perioperative fractures, and compared in each group.

**Results**

In this study, a total of 141 patients (group NE: 2 male and 48 female, group NL: 17 male and 46 female, group B: 28 female) were included. No significant differences were found among the groups in age, BMI, or disease. The time of surgery was significantly shorter in the NL and B groups than in the NE group, with the B group being the shortest significantly (NE: 108 min, NL: 99 min, B: 85 min; *P* < 0.05). Incision length was significantly longer in the NE group (NE: 8.5 cm, NL: 8.0 cm, B: 8.0 cm; *P* < 0.05).

There were no significant differences in cup alignment or stem alignment among the groups. 92.0% of the NE group, 92.1% of the NL group, and 92.9% of the B group were within Lewinnek’s safe zone, and no significant differences were observed. Perioperative fractures occurred in 2 patients in the NE group, 1 patient in the NL group, and 1 patient in the B group.

**Conclusions**

In this study, the latter AMIS introduction group showed significantly shorter incision length and shorter operation time, suggesting the existence of a significant learning curve. The bikini incision group was introduced after the surgeons had fully mastered AMIS, which allowed for further shortening of surgery time and successful implant placement. However, perioperative fractures were observed in each group, indicating that careful operation is necessary regardless of skill level. In conclusion, AMIS-THA has a learning curve, but once the surgeon has sufficient skill, the bikini incision can be performed safely, and the outcomes have been good.

## P16 Multiview deep learning framework for early detection of PJI from preoperative MRI

### Zheng Su, Tao Zhang, Ruixiang Ma, Chen Zhu

#### Department of Orthopedics, The First Affiliated Hospital of USTC, Division of Life Sciences and Medicine, University of Science and Technology of China, Hefei, China

##### **Correspondence:** Zheng Su (suz924@ustc.edu.cn); Chen Zhu (zhuchena@ustc.edu.cn)

*Arthroplasty 2026*, **8(1):**P16

**Background**

Periprosthetic joint infection (PJI) is a severe complication following joint replacement surgery associated with increased morbidity, poor functional outcomes, and high healthcare costs. Early and accurate diagnosis is critical to improving patient prognosis and guiding timely surgical or pharmacological intervention. Current diagnostic methods, including serological tests and synovial fluid analysis, are often invasive and limited in sensitivity and specificity.

**Methods**

To address these limitations, we developed a novel deep learning-based tool that integrates U-Net for MRI-based region of interest (ROI) segmentation and DenseNet201 for classification to predict the incidence of postoperative PJI. The U-Net architecture was enhanced with skip connections and squeeze-and-excitation (SE) modules to improve boundary feature extraction and segmentation accuracy. We applied class-weighted loss functions to address the class imbalance, and the Youden Index was used to determine the optimal classification threshold, maximizing sensitivity and specificity.

**Results**

In a validation cohort, the model achieved an area under the precision-recall curve (AUC) of 0.9938 with an optimal threshold of 0.3481 (Fig. 1a), yielding a recall of 0.96 and an F1-score of 0.98 for the infection class (Table 1). Then, the t-distributed stochastic neighbor embedding (T-SNE) analysis showed that our model extracts powerful features for distinguishing different classes in the latent space (Fig. 1b).

**Conclusions**

This tool offers a noninvasive and highly accurate approach to preoperative risk stratification, supporting orthopedic surgeons in the early identification of high-risk patients and improving clinical decision-making to reduce postoperative complications.

**Fig. 1 (Abstract P16).**
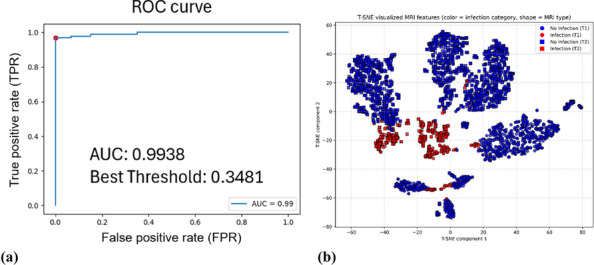
**a** Analysis and evaluation of deep learning models; **b** T-SNE visualized MRI features at coronal view, sagittal view, and axial view

**Table 1 (Abstract P16).** Classification report

	**Precision**	**Recall**	**F1-score**
Non-infection	0.99	1.00	1.00
Infection	1.00	0.96	0.98

## P17 Disengagement of tibial insert locking pin in total knee arthroplasty is rare, but it happens

### Leong Jin Kai^1^, Samsher Singh Nahal^1^, Wong Shu Kok^1^, Kishore Naath Muniandy^1^, Naga Bharathi Nesan^1^, Teh Hak Lian^1^, Veenesh Selvaratnam^2^

#### ^1^Joint replacement unit, Department of Orthopaedics, Hospital Raja Permaisuri Bainun, Ipoh, Malaysia; ^2^Joint reconstruction unit, National Orthopaedic Centre of Excellence for Research and Learning (NOCERAL), Department of Orthopaedic Surgery, Faculty of Medicine, University Malaya, Kuala Lumpur, Malaysia

##### **Correspondence:** Teh Hak Lian (drteh1984@yahoo.com)

*Arthroplasty 2026*, **8(1):**P17

**Background**

Total knee arthroplasty (TKA) has seen numerous advancements over the decades. Different polyethylene locking mechanisms were designed to minimize micromotion and wear issues. These include central, peripheral, both central and peripheral, as well as a locking pin mechanism. Locking pin mechanism for polyethylene insert was designed to reduce interface micromotion and backside wear in modular implants. However, unexpected dislodgement of the tibial insert locking pin may compromise the stability of the knee, giving a traumatic experience to the patient and surgeon.

**Report**

A 56-year-old gentleman with a medical history of diabetes mellitus, hypertension, and dyslipidaemia presented to us with grade 4 left knee osteoarthritis. He underwent a left TKA using a design with a locking pin mechanism in the tibial polyethylene insert. Four months postoperatively, the patient was in pain with a limp. He denied a history of trauma or fever. There was a palpable, sharp medial knee swelling with a restricted range of motion. Plain radiographs of the knee revealed a dislodged locking pin from the tibial component. He has to undergo surgery in the form of removal of the dislodged pin and old polyethylene insert, reinsertion of a new polyethylene insert, which was secured with a new locking pin. Intraoperatively, it was noted that the hook-end of the pin was bent downwards and was caught in the medial subcutaneous tissue. Cultures from the joint fluid and tissue samples were negative for infection.

**Discussion**

Disengagement of the locking pin may lead to dislocation of the insert and subsequently dislocation of the knee. It is uncertain what the causative factor is for such a complication. Possible incomplete locking effort or improper locking fit at the index surgery may be the root cause, or the patient himself did not disclose a history of trauma.

**Conclusions**

Although disengagement of the locking pin is not common, it should be suspected if a patient presents with acute medial knee swelling and pain post-TKA.

## P18 The role of applying metagenomic next-generation sequencing (mNGS) in periprosthetic joint infection with sinus tract: a retrospective study

### Xuhui Yuan, Xinyu Fang, Wenming Zhang

#### Department of Orthopaedic Surgery, the First Affiliated Hospital, Fujian Medical University, Fuzhou, China

##### **Correspondence:** Wenming Zhang (zhangwm0591@fjmu.edu.cn)

*Arthroplasty 2026*, **8(1):**P18

The full article of this study has been published online. Please refer to the full text at: 10.2147/IJGM.S531444.

## P19 Close to home or far away: impact of geographical distance on recovery post-total knee arthroplasty

### Wing Ki Cheung, Yim Ling Amy Cheung, Michelle Hilda Luk, Ka Chun Thomas Leung, Chun Man Lawrence Lau, Chun Him Henry Fu

#### Division of Joint Replacement Surgery, Department of Orthopaedics and Traumatology, The University of Hong Kong, Hong Kong SAR, China

##### **Correspondence:** Wing Ki Cheung (suec01@hku.hk)

*Arthroplasty 2026*, **8(1):**P19

**Background**

Patients who have undergone Total Knee Arthroplasty need to return to the hospital regularly for follow-up, physiotherapy, and occupational therapy. Many patients have reported experiencing significant pain, which has led to reluctance to move around and attend hospital follow-up appointments. This retrospective study aims to analyze the impact of geographical and social factors on the recovery outcomes of patients.

**Methods**

Patients who underwent unilateral total knee arthroplasty for the first time between June 2020 and December 2021, with a three-year follow-up, were included in the study. Data on age, sex, body mass index, number of individuals residing together, distance from home to the hospital, and whether patients needed to climb stairs to access their homes were gathered. The study also took into account the assumption that patients utilized the fastest public transportation available, and measurements were taken for the number of transitions from home to the hospital, time taken, and walking distance required. Knee Society Knee Score (KSKS), which evaluates patients at pain, range of motion, stability, flexion contracture, extension lag, and alignment, and Knee Society Functional Assessment (KSFA), which evaluates the mobility during walking, stairs, and the use of walking aids, data were collected at preoperative, 1-month, 1-year, and 3-year postoperative intervals. Spearman’s rank-order correlation was first applied, and variables with a *P*-value less than 0.15 were included in multivariable linear regression to examine the relationship between the parameters and the outcomes of recovery.

**Results**

Out of a total of 130 patients screened, 41 (35 Female and 6 male) were included in the study. At 1-month post-surgery, the pain index from KSKS showed a weak positive correlation with the need to climb stairs (*r* = 0.329, *P* = 0.046). Age (*t* = − 2.42, *P* = 0.021) and the need to climb stairs (*t* = 2.35, *P* = 0.024) significantly predicted KSFA at 1-month post-surgery (*F*(3,37) = 3.59, *P* = 0.23, *R*^2^ = 0.225) (Table 1). A higher number of public transport transitions (*t* = 2.07, *P* = 0.046) and a better preoperative KSFA score (*t* = 2.41, *P* = 0.022) were significant predictors of KSFA at 1-year post-surgery (*F*(5,33) = 3.54, *P* = 0.011, *R*^2^ = 0.349) (Table 2). A higher number of public transport transitions (*t* = 2.07, *P* = 0.047) and being female (*t* = 3.19, *P* = 0.003) significantly predicted higher KSFA scores at 3-year post-surgery (*F*(6,32) = 3.90, *P* = 0.005, *R*^2^ = 0.422) (Table 3). Lastly, no variables were found to be statistically significant predictors of pain index at 1 year and 3 years post-surgery, as well as KSKS scores at 1 month, 1 year, and 3 years post-surgery.

**Conclusions**

The results suggest that in the long term, a larger number of transitions from home to the hospital and being female are associated with better functional recovery. No factors were found to be associated with pain at 1 and 3 years after surgery. Therefore, it is unlikely that regular follow-up will result in poor recovery.

**Table 1 (Abstract P19).** Multivariable linear regression analysis of KSFA at 1 month after surgery

	**Unstandardized Coefficients**		**Standardized Coefficients**	**t**	**Sig**	**95.0% Confidence Interval for B**	
	**B**	**Std. Error**	**Beta**			**Lower Bound**	**Upper Bound**
(Constant)	85.046	24.051		3.536	0.001	36.315	133.778
Age	− 0.697	0.288	− 0.361	− 2.42	0.021*	− 1.281	− 0.114
Stairs	9.566	4.066	0.344	2.352	0.024*	1.327	17.805
Preoperative KSFA	0.018	0.14	0.019	0.129	0.898	− 0.265	0.301

*KSFA* Knee Society Knee Assessment

*Denotes statistical significance, *P* < 0.05

**Table 2 (Abstract P19).** Multivariable linear regression analysis of KSFA at 1 year after surgery

	**Unstandardized Coefficients**		**Standardized Coefficients**	**t**	**Sig**	**95.0% Confidence Interval for B**	
	**B**	**Std. Error**	**Beta**			**Lower Bound**	**Upper Bound**
(Constant)	22.477	56.581		0.397	0.694	− 92.638	137.592
Age	− 0.7	0.529	− 0.206	− 1.323	0.195	− 1.776	0.376
Number Of Transition	8.343	4.03	0.3	2.07	0.046*	0.144	16.543
BMI	0.524	0.697	0.115	0.752	0.457	− 0.894	1.942
Live Alone	16.672	9.151	0.269	1.822	0.078	− 1.945	35.289
Preoperative KSFA	0.578	0.24	0.367	2.405	0.022*	0.089	1.067

*BMI* body mass index, *KSFA* Knee Society Knee Assessment

*Denotes statistical significance, *P* < 0.05

**Table 3 (Abstract P19).** Multivariable linear regression analysis of KSFA at 3 years after surgery

	**Unstandardized Coefficients**		**Standardized Coefficients**	**t**	**Sig**	**95.0% Confidence Interval for B**	
	**B**	**Std. Error**	**Beta**			**Lower Bound**	**Upper Bound**
(Constant)	40.393	46.459		0.869	0.391	− 54.24	135.027
Age	− 0.823	0.464	− 0.252	− 1.774	0.086	− 1.767	0.122
Number Of Transition	11.205	5.419	0.421	2.068	0.047*	0.166	22.243
Travel Time	− 0.07	0.146	− 0.097	− 0.478	0.636	− 0.367	0.228
Sex	23.598	7.395	0.438	3.191	0.003*	8.536	38.66
Live Alone	7.99	8.372	0.134	0.954	0.347	− 9.064	25.043
Preop KSFA	0.258	0.219	0.171	1.178	0.248	− 0.188	0.704

*KSFA* Knee Society Knee Assessment

*Denotes statistical significance, *P* < 0.05

## P20 Early outcomes of kinematic alignment in robotic-assisted medial UKA

### Dasheng Lin, Dongmin Xu, Wentao Lin, Eryou Feng

#### Department of Orthopedic Surgery, Fujian Medical University Union Hospital, Fuzhou, China

##### **Correspondence:** Dasheng Lin (linds@xmu.edu.cn)

*Arthroplasty 2026*, **8(1):**P20

**Background**

To investigate the early outcomes of kinematic alignment in robotic-assisted medial UKA.

**Methods**

From January 2024 to December 2024, 20 patients (20 knees) underwent robotic-assisted medial UKA using kinematic alignment, including 7 males and 13 females, aged 52–75 years (mean 62.5 years), with a body mass index (BMI) of 19.5–34.8 kg/m^2^ (mean 25.6 kg/m^2^). Postoperative evaluations included radiographic outcomes, knee function, and complications. Radiographic assessments utilized weight-bearing knee X-rays and full-length lower limb radiographs to measure prosthesis positioning, angular deviations, and posterior tibial slope (PTS). Functional outcomes included range of motion (ROM), American Knee Society Score (KSS), Oxford Knee Score (OKS), and Forgotten Joint Score (FJS).

**Results**

All patients were followed for 6–18 months (mean 10.6 months). Operative time ranged from 60–85 min (mean 72 min), with intraoperative blood loss of 50–150 mL (mean 74 mL). Incisions healed primarily. At final follow-up, all prostheses remained well-positioned without abnormal angular deviations compared to immediate postoperative measurements. Knee ROM improved from 112.3° ± 12.7° preoperatively to 127.9° ± 14.2° at final follow-up. KSS and OKS scores showed significant improvement (*P* < 0.01), with a mean FJS of 86.4 ± 3.7. Two patients experienced anterior knee pain within 3 months postoperatively, which resolved after symptomatic management.

**Conclusions**

Robotic-assisted medial UKA using kinematic alignment precisely replicates the pre-osteoarthritic anatomical state of the knee, enhances osteotomy accuracy, optimizes prosthesis positioning, and facilitates rapid recovery. Short-term follow-up demonstrated favorable prosthesis survival, no positional abnormalities, excellent functional outcomes, and superior patient-reported clinical results. However, mid- to long-term efficacy requires further validation.

## P21 Deeper flexion and male gender predict the ability to squat and/or kneel after knee replacement: a study amongst the Southeast Asian population

### Ervin Sethi, Tong Leng Tan, Remesh Kunnasegaran, Michael Gui Jie Yam, Ho Poh Wong, Yee Hong Teo, Yin Peng Low, Kelvin Guoping Tan

#### Department of Orthopaedic Surgery, Tan Tock Seng Hospital, Singapore

##### **Correspondence:** Ervin Sethi (Ervin.sethi@mohh.com.sg)

*Arthroplasty 2026*, **8(1):**P21

**Background**

Squatting and kneeling are activities that play a significant role in our daily activities, especially in many Asian cultures. Western data has shown that the ability to do so after knee arthroplasty has traditionally been low, citing that patients did not require squatting and kneeling to perform most of their daily activities. However, in many Asian cultures, these actions are needed for essential activities such as religious prayers and having a meal. This study aims to evaluate if patients can squat and kneel 1 year after knee arthroplasty procedures and to identify factors that predict them.

**Methods**

A total of 852 knees from 791 patients (252 males, 539 females, Age 44–89) who have completed 1-year follow-up were identified from an institution’s knee registry database. Oxford Knee Score (OKS) and Knee Society Scores (KSS), and the ability to squat and/or kneel were collected as part of the database functional outcomes assessments.

**Results**

Of the 852 knees recruited, 342 (40.14%) could squat 1 year post-knee arthroplasty surgery, while 294 (34.51%) patients were able to kneel. 246 (28.88%) of the 852 knees were able to achieve both squatting and kneeling at 1-year post-surgery (Fig. 1). We analyzed each of these sub-groups independently based on the objective and clinical scores mentioned above. Further analysis showed that male gender and patients’ post-operative degree of knee flexion were two significant (*P* < 0.05) factors that correlated with the patient’s ability to squat and/or kneel post-operatively. Patients’ inability to squat and/or kneel pre-operatively was also a significant negative predictor of their ability to do so after surgery. Functional outcome scores (OKS and KSS) were higher in each sub-group of patients as compared to those who were unable to squat and/or kneel. This also translated into higher patient satisfaction.

**Conclusions**

This study reinforced the fact that kneeling and squatting can be achieved after knee arthroplasty surgery. We also managed to identify that gender and post-operative degree of knee flexion are key factors that influenced patients’ ability to squat and kneel post-operatively. Having the ability to squat and/or kneel also translated into better functional outcomes for patients. This valuable information can help serve as a guide when counselling patients pre-operatively and managing their expectations on post-operative functional outcomes.

**Fig. 1 (Abstract P21).**
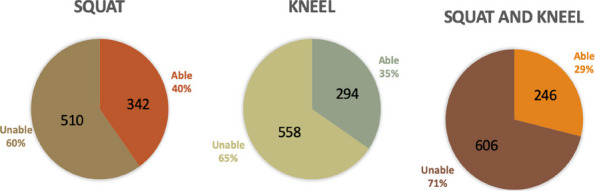
Breakdown of patients able to squat, kneel, or both

## P22 Risk factors associated with very early functional outcomes following robot-assisted total knee arthroplasty

### Pengcheng Li, Runkai Zhao, Qingqing Qi, Jianfeng Yang, Zeyu Feng, Quanbo Ji, Guoqiang Zhang

#### Department of Orthopaedics, General Hospital of Chinese People's Liberation Army, Beijing, China

##### **Correspondence:** Guoqiang Zhang (gqzhang_plagh@163.com)

*Arthroplasty 2026*, **8(1):**P22

**Background**

Total knee arthroplasty (TKA) is the definitive treatment for end-stage knee osteoarthritis, but postoperative recovery often depends on multiple factors. Clinically, some patients achieve significant functional improvement as early as two weeks postoperatively. However, the factors influencing very early (2-week) functional outcomes after TKA remain unclear. This study aims to comprehensively evaluate the impact of preoperative clinical indicators and surgical factors on very early functional outcomes in patients with knee osteoarthritis, using changes in the functional Knee Society Score (fKSS) at two weeks postoperatively to guide rehabilitation strategies for robot-assisted TKA (RA-TKA).

**Methods**

Clinical data were collected from 31 patients with end-stage knee osteoarthritis who underwent RA-TKA at the Department of Orthopaedics, General Hospital of the Chinese People’s Liberation Army, between December 2024 and January 2025. All patients underwent RA-TKA performed by the same senior surgeon and received outpatient-guided rehabilitation exercises. Variables included demographic data, preoperative imaging, laboratory tests, surgical details, and preoperative and 2-week postoperative scale scores (Table 1). Univariate analysis of variance or Pearson correlation analysis was performed, and variables with *P* < 0.05 were included in a multivariate linear regression model.

**Results**

Correlation analysis revealed significant associations between postoperative fKSS changes and the following variables: Physical Functioning of SF-36 (*P* = 0.0454), Bodily Pain of SF-36 (*P* = 0.0038), Social Functioning of SF-36 (*P* = 0.0337), and serum uric acid (*P* = 0.0448). Other variables, such as Role-Physical, Role-Emotional, and WOMAC scores, also showed trends (Table 2). Multivariate linear regression identified Bodily Pain of SF-36 as the most significant predictor (*P* = 0.037) (Table 3), with a negative correlation estimate of − 0.492.

**Discussion**

Preoperative bodily pain was an independent risk factor for very early functional outcomes after TKA. Severe preoperative pain may hinder rapid functional recovery through outpatient-guided rehabilitation, possibly linked to psychological factors. Other potential predictors, such as UA and Role-Emotional, align with prior studies on osteoarthritis progression and TKA outcomes. Larger sample sizes may reveal additional factors.

**Conclusions**

This study comprehensively evaluated preoperative and surgical factors influencing very early functional recovery after RA-TKA. It highlighted the critical role of preoperative pain in early rehabilitation, providing insights to optimize rapid recovery and functional exercise within two weeks postoperatively. These findings support tailored strategies to enhance outcomes in patients undergoing RA-TKA.

**Table 1 (Abstract P22).** Patient clinical characteristics

Characteristic	All patients (31)
Gender	5 (16.13%) males; 26 (83.87%) females
Age (years)	66.26 ± 4.97
BMI (kg/m^2^)	26.35 ± 3.12
Charlson comorbidity index	4.32 ± 1.9
Pain Duration (years)	9.83 ± 10.27
Postoperative Hospital Stay (days)	3.77 ± 1.33
Total Hospital Stay (days)	9 ± 2.98
Flexion (degrees)	117.58 ± 17.79
Extension (degrees)	5.16 ± 7.5
HKA (degrees)	7.97 ± 8.57
mMPTA (degrees)	84.42 ± 3.78
mLDFA (degrees)	90.22 ± 4.74
JLCA (degrees)	3.69 ± 2.07
Femoral AMA (degrees)	6.5 ± 1.77
K-L Grade	Grade 3: 12 (38.71%); Grade 4: 19 (61.29%)
Surgery Duration (minutes)	122.14 ± 24.76
Laterality	19 left (61.29%); 12 right (38.71%)
Femur Distal Medial Resection (mm)	7.65 ± 1.92
Femur Distal Lateral Resection (mm)	6.6 ± 2.31
Femur Posterior Medial Resection (mm)	8.13 ± 1.32
Femur Posterior Lateral Resection (mm)	5.82 ± 2.17
Tibia Medial Resection (mm)	4.66 ± 1.86
Tibia Lateral Resection (mm)	8.08 ± 2.16
Patellar Treatment	Retained: 24 (77.42%); Replaced: 7 (22.58%)
Femoral Prosthesis Size	4.71 ± 0.97
Tibial Prosthesis Size	3.81 ± 1.01
Liner Thickness (mm)	7.4 ± 2.16
Preoperative SF-36 PF	32.42 ± 18.97
Preoperative SF-36 RP	16.13 ± 33.26
Preoperative SF-36 BP	39.26 ± 17.26
Preoperative SF-36 GH	55.71 ± 18.42
Preoperative SF-36 VT	57.9 ± 21.09
Preoperative SF-36 SF	50.38 ± 20.94
Preoperative SF-36 RE	39.81 ± 43.4
Preoperative SF-36 MH	68.77 ± 17.45
Preoperative SF-36 HT	69.39 ± 22.1
Preoperative WOMAC	46.41 ± 14.92
Preoperative KSS Function	35.45 ± 14.77
Postoperative KSS Function	38.95 ± 19.89
WBC (10^9^/L)	5.40 ± 1.11
Hemoglobin (g/L)	125.37 ± 18.32
NLR	1.38 ± 0.57
PLR	630.92 ± 207.65
Albumin (g/L)	41.62 ± 2.65
PT (s)	11.42 ± 0.98
APTT (s)	28.35 ± 3.03
Fibrinogen (g/L)	2.67 ± 0.55
ESR (mm/h)	8.42 ± 5.3
IL-6 (pg/mL)	4.23 ± 3.77
CRP (mg/dL)	0.12 ± 0.09
Uric Acid (μmol/L)	256.37 ± 63.54
RDW (%)	12.32 ± 0.63
INR	0.99 ± 0.07
Sodium (mmol/L)	141.54 ± 2.91

**Table 2 (Abstract P22).** Correlation analysis of factors affecting 2-week postoperative knee function

Variable	*r* Value	*P* Value
Age	0.063	0.7368
BMI (kg/m^2^)	− 0.086	0.6447
Comorbidity Index	0.238	0.1978
Pain Duration (years)	− 0.018	0.9249
Postoperative Hospital Stay (days)	− 0.092	0.6236
Total Hospital Stay (days)	− 0.005	0.9779
Flexion Angle (degrees)	0.184	0.323
Extension Angle (degrees)	0.081	0.6665
HKA Angle (degrees)	− 0.222	0.2296
mMPTA (degrees)	0.032	0.864
mLDFA (degrees)	− 0.183	0.3238
JLCA (degrees)	0.007	0.9718
Femoral AMA (degrees)	− 0.294	0.1085
Surgery Duration (minutes)	0.11	0.5568
Femur Distal Medial Resection Depth (mm)	0.292	0.1115
Femur Distal Lateral Resection Depth (mm)	− 0.179	0.3342
Tibia Medial Resection Depth (mm)	0.124	0.5057
Tibia Lateral Resection Depth (mm)	− 0.044	0.8136
Femur Posterior Medial Resection Depth (mm)	− 0.135	0.4704
Femur Posterior Lateral Resection Depth (mm)	0.204	0.2701
Liner Thickness	− 0.068	0.7157
Tibial Prosthesis Size	0.073	0.6979
Femoral Prosthesis Size	− 0.033	0.8597
Preoperative SF-36 PF	​​ − 0.362​​	​​0.0454​​
Preoperative SF-36 RP	− 0.322	0.0773
Preoperative SF-36 BP	​​ − 0.505​​	​​0.0038​​
Preoperative SF-36 GH	− 0.21	0.2579
Preoperative SF-36 VT	0.001	0.9963
Preoperative SF-36 SF	​​ − 0.383​​	​​0.0337​​
Preoperative SF-36 RE	− 0.347	0.0557
Preoperative SF-36 MH	− 0.045	0.8083
Preoperative SF-36 HT	− 0.035	0.8537
Preoperative WOMAC	0.327	0.0722
WBC (10^9^/L)	0.137	0.4632
Hemoglobin (g/L)	0.054	0.7728
NLR	0.161	0.386
PLR	0.181	0.3289
Albumin (g/L)	0.148	0.4276
PT (s)	0.299	0.1019
APTT (s)	0.273	0.1374
Fibrinogen (g/L)	0.22	0.2353
ESR (mm/h)	− 0.129	0.4892
IL-6 (pg/mL)	0.199	0.2836
CRP (mg/dL)	0.079	0.6708
Uric Acid (μmol/L)	​​0.363​​	​​0.0448​​
RDW (%)	− 0.113	0.5467
INR	0.23	0.2137
Sodium (mmol/L)	− 0.162	0.3847

**Table 3 (Abstract P22).** Multivariate linear regression analysis of factors affecting 2-week postoperative knee function

Variable	Estimate	Std. Error	Statistic	*P* Value	95% CI
(Intercept)	− 1.352	18.239	− 0.074	0.941	− 38.842 to 36.138
Preoperative SF-36 PF	− 0.013	0.187	− 0.071	0.944	− 0.398 to 0.371
Preoperative SF-36 BP	− 0.492	0.224	− 2.196	0.037	− 0.953 to − 0.031
Preoperative SF-36 SF	0.031	0.183	0.17	0.866	− 0.345 to 0.407
Uric Acid (μmol/L)	0.09	0.052	1.741	0.093	− 0.016 to 0.196

## P23 Early experience with patient-specific instrumentation for opening-wedge high tibial osteotomy

### Hui Ming Leon, Khairul Anwar Ayob, Kwong Weng Loh

#### Department of Orthopaedic Surgery, Universiti Malaya, Kuala Lumpur, Malaysia

##### **Correspondence:** Hui Ming Leon (drleonhuiming@outlook.com)

*Arthroplasty 2026*, **8(1):**P23

**Background**

Patient-specific instrumentation (PSI) has been introduced in high tibial osteotomy to enhance surgical accuracy and optimize clinical outcomes in the management of medial compartment knee osteoarthritis. However, the radiological and clinical outcomes associated with its use have yet to be established in our clinical setting. This study aims to evaluate the radiological and clinical outcomes of PSI-assisted opening-wedge high tibial osteotomy (OWHTO).

**Methods**

This retrospective case series evaluates the impact of PSI in OWHTO, focusing on surgical accuracy and functional outcomes. Patients with medial compartment knee osteoarthritis who underwent OWHTO using PSI were included. Postoperative alignment correction was assessed from radiographic measurements. Clinical outcomes were evaluated in patients who completed a minimum of 24 months of follow-up, using patient-reported outcome measures (PROMs) (Fig. 1). Data were analyzed using two-tailed paired t-tests.

**Results**

At a mean follow-up of 52 (± 16.6) months, clinical outcomes assessed using patient-reported outcome measures (PROMs) were available for five patients, as summarized in Table 1. A statistically significant improvement was observed in the WOMAC score (*P* = 0.011). However, no significant differences were noted in the KOOS (*P* = 0.050) and OKS (*P* = 0.055) scores. Radiographic analysis of 12 knees demonstrated significant improvements in mechanical axis deviation, hip-knee-ankle angle, and medial proximal tibial angle (all *P* < 0.001), as shown in Table 2. There was no significant difference in the posterior medial tibial slope compared to preoperative measurements (*P* = 0.462). Complications included one case of lateral tibial plateau fracture requiring conversion to total knee arthroplasty and one case of prominent implant requiring removal.

**Conclusions**

In this case series, the use of PSI in high tibial osteotomy enhanced surgical precision, resulting in improved alignment accuracy and satisfactory clinical outcomes. Although further studies with larger cohorts and longer follow-up periods are warranted, our findings suggest a positive trend toward improved activities of daily living and return to work following PSI-guided OWHTO, with minimal risk of major complications.

**Fig. 1 (Abstract P23).**
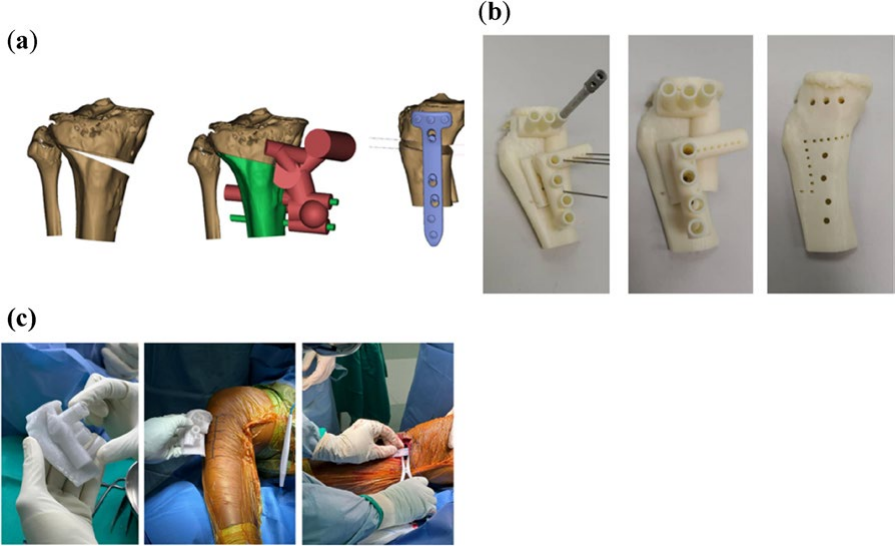
**a** Preoperative 3D imaging of OWHTO using PSI; **b** Trial of PSI on bone model; **c** Intraoperative use of PSI in patients undergoing OWHTO

**Table 1 (Abstract P23).** Patient-reported outcome measures (mean ± standard deviation)

PROM	Preoperative	Postoperative	*P* value
KOOS	89.2 ± 42.1	28.6 ± 35.2	0.05
WOMAC	28.6 ± 13.2	5.2 ± 9.1	0.011
OKS	49.8 ± 42.9	7.2 ± 15	0.055

**Table 2 (Abstract P23).** Radiographic alignment measures (mean ± standard deviation)

	**Preoperative**	**Postoperative**	***p* value**
Mechanical axis deviation (mm)	41.28 ± 14.96	− 0.97 ± 14.98	< 0.001
Hip-knee-ankle angle (°)	12.75 ± 4.20	− 1.30 ± 4.98	< 0.001
Medial proximal tibial angle (°)	6.23 ± 1.96	− 1.29 ± 3.68	< 0.001
Posterior medial tibial slope (°)	10.7 ± 3.2	11.7 ± 4.07	0.462

## P24 Comparison of outcomes of medial unicompartmental knee replacement and total knee replacement for patients with medial knee osteoarthritis and anterior cruciate ligament injuries

### Miao Wang^1^, Hong Fan^1^, Jian Sun^1^, Kun Tao^2^

#### ^1^Department of Orthopedics, Jiading branch of Shanghai General Hospital, Shanghai Jiaotong University School of Medicine, Jiading District Jiangqiao Hospital, Shanghai, China; ^2^Department of Orthopedics, The Shanghai Tenth People’s Hospital of Tongji University, Shanghai, China

##### **Correspondence:** Kun Tao (doctk@163.com)

*Arthroplasty 2026*, **8(1):**P24

**Background**

Unicompartmental knee replacement is a more conservative and ligament-sparing surgical approach for treating medial knee osteoarthritis. however, there remains controversy that patients with anterior cruciate ligament injuries do not require the UKA for possible knee instability and limitation of the prosthesis design in managing torn ACL. This study aimed to investigate and compare the outcomes of UKA and TKA for patients with medial knee osteoarthritis and secondary ACL injuries.

**Methods**

Forty patients with medial knee osteoarthritis and secondary ACL injuries were included in this retrospective study, which had a one-year follow-up, and they were divided into two groups based on different treatment methods. 20 patients received TKA, and 20 patients received Unicompartmental knee replacement. Preoperative and postoperative X-ray images and complications were taken. Outcome measures were the visual analog scale (VAS) and the Hospital for Special Surgery score (HSS).

**Results**

All the patients completed the one-year follow-up. Postoperative complications included surgical site infection and knee joint stiffness, which were observed in two patients of 20. At one-year follow-up, in the TKA group, the average VAS score was 4.1 ± 1.2, and the average HSS score was 75.5 ± 10.4. In the UKA group, the average VAS score was 3.2 ± 1.5, and the average HSS score was 87.1 ± 13.5. The two groups showed no statistically significant difference in VAS score. Meanwhile, a statistically significant difference was demonstrated in the HSS score.

**Conclusions**

The TKA group showed better clinical outcomes compared to the UKA group with statistically significant differences in our study, while TKA and UKA could both effectively alleviate patients’ symptoms.

## P25 The impact of periacetabular osteotomy combined with proximal femoral derotational osteotomy on foot progression angle: a retrospective study

### Xing Qiu, Ningtao Ren, Hui Cheng, Dianzhong Luo, Yong Li, Haigang Jia, Hong Zhang

#### Senior Department of Orthopedics, The Fourth Medical Center of Chinese PLA General Hospital, Beijing, China

##### **Correspondence:** Hong Zhang (zhanghongmd@163.com)

*Arthroplasty 2026*, **8(1):**P25

**Background**

Developmental dysplasia of the hip (DDH) and excessive femoral anteversion can lead to biomechanical derangements in the hip joint and abnormal foot progression angle (FPA). While periacetabular osteotomy (PAO) combined with proximal femoral derotational osteotomy (PFO) effectively corrects bony deformities, its systemic impact on FPA remains understudied.

**Methods**

Single-center retrospective study (April 2015–December 2024) involving 56 DDH patients (inclusion criteria: age > 16 years, Tonnis DDH grade I–III, lateral center–edge angle < 20°, femoral anteversion > 40°), excluding neuromuscular disorders. Outcome Measures: Radiographic: Preoperative and 6-month postoperative CT measurements of acetabular anteversion, femoral anteversion, and femoral torsion. Gait Analysis: Foot progression angle assessed via footprint method combined with 2D imaging (due to equipment limitations), defined as the angle between the foot’s longitudinal axis and walking direction. Clinical Scores: Harris Hip Score (HHS) and gait symmetry index. Statistical Analysis: Paired t-tests for preoperative-postoperative comparisons; Pearson correlation for associations.

**Results**

Radiographic Improvements: Lateral center–edge angle increased from 18.5 ± 3.2° to 25°–30°, anterior center–edge angle from 12.8 ± 2.5° to 20°–25°, and femoral anteversion decreased from 45.6 ± 5.1° to 10°–15° postoperatively. FPA Correction: FPA shifted from preoperative internal rotation (2.2 ± 8.3°) to near-neutral alignment (9.7 ± 7.8°), with gait symmetry improved by 30%. Correlation: Strong positive correlation between femoral anteversion correction and FPA improvement.

**Conclusions**

PAO combined with PFO effectively corrects hip-femoral deformities and improves FPA, likely mediated by rotational realignment of the lower limb. This study provides biomechanical evidence to optimize combined surgical strategies.

## P26 Total knee arthroplasty in a patient with ochronosis: a case report

### Sachin Kumar, Iknoor Singh Mann, Kiran K V Acharya

#### Department of Orthopaedics, Kasturba Medical College, Manipal, Manipal Academy of Higher Education, Karnataka, India

##### **Correspondence:** Sachin Kumar (devadiga.sachin@manipal.edu)

*Arthroplasty 2026*, **8(1):**P26

**Background**

Ochronotic arthropathy is a rare manifestation of alkaptonuria, characterized by the accumulation of homogentisic acid in connective tissues, leading to progressive pigmentation and degeneration of cartilage. The disease often results in early-onset osteoarthritis, particularly affecting large weight-bearing joints such as the knees and hips. Total knee replacement (TKR) has emerged as an effective intervention for end-stage ochronotic arthropathy of the knee, offering significant pain relief and functional improvement. However, the pathological changes in connective tissue quality and pigmentation can pose unique intraoperative challenges. A better understanding of surgical outcomes in this subgroup is critical to guide clinical decision-making and optimize patient care.

**Report**

A 64-year-old man came with a history of bilateral knee pain, more severe on the left side than the right. The X-ray revealed severe degenerative changes in the left knee, including osteophytes and symmetrically reduced joint space, and dished-out proximal tibial articular surface. The patient underwent left total knee replacement. A standard midline incision was performed. On exposure, the quadriceps tendon, patellar tendon, femur, tibia, and patellar articular cartilage showed blackish discoloration. Tissue samples from bone and the synovium were sent, and an intraoperative presumptive diagnosis of ochronosis was made. During eversion of the patella, as the tendon was friable due to abnormal pigment deposition, there was a thickened patellar tendon rupture. The ruptured tendon was repaired. Following a biopsy report and a positive urine homogentisic acid test postoperatively, ochronotic arthritis was diagnosed.

**Discussion**

Total knee replacement in patients with ochronotic arthropathy has shown outcomes comparable to those observed in primary osteoarthritis. Intraoperative findings often include brittle, discolored cartilage and darkened synovium, requiring careful soft tissue handling to prevent complications. The altered tissue characteristics and potential for spinal involvement require thorough preoperative planning. Awareness of the systemic nature of alkaptonuria is also important for optimizing perioperative management. Long-term results suggest that prosthetic survival in ochronotic patients is favorable.

**Conclusions**

Our case study shows that individuals with ochronotic arthropathy have great results with total knee arthroplasty. Given the high incidence of TKRs in ochronotic arthropathy, there is a considerable risk of intraoperative complications due to tendon attenuation and degeneration. Preoperative diagnosis is therefore essential to treat the tendon more carefully during TKR.

**Consent for publication**

Written informed consent was obtained from the patient for publication of this case report.

## P27 Biomimetic discrete design of ceramic hip prostheses inspired by turtle shells and armadillo scales

### Shan Wei^1^, Xinxin Zhang^1^, He Liu^1^, Chengtao Xu^1^, Assia Faik^1^, Zhengyu Wang^2^

#### ^1^Anhui Polytechnic University, Wuhu, China; ^2^The First Affiliated Hospital of Wannan Medical College, Yijishan Hospital, Wuhu, China

##### **Correspondence:** Shan Wei (ws@ahpu.edu.cn)

*Arthroplasty 2026*, **8(1):**P27

**Background**

Artificial hip replacement is a critical treatment for hip joint injuries, yet conventional implants designed with Western biomechanical parameters fail to accommodate the greater hip range of motion (ROM) required by Asian populations during high-flexion activities (e.g., squatting, kneeling). This necessitates thinner ceramic acetabular liners with larger femoral heads, increasing fracture risks in traditional monolithic designs.

**Methods**

An innovative biomimetic prosthesis was developed, featuring 133 radially arranged 2.5-mm alumina ceramic plates within a titanium framework, inspired by turtle/armadillo armor morphology. The design was optimized through CT-based 3D reconstruction and finite element analysis (FEA), leveraging ceramic’s small-size effect and natural stress-distribution mechanisms. Performance was evaluated against monolithic designs via FEA and experimental validation, including fatigue testing (5 million cycles), surface roughness, flatness, and hardness measurements (Figs. 1–3).

**Results**

The biomimetic design reduced acetabular wall thickness by 15–20% while maintaining structural integrity, with peak stresses (34.995 MPa) well below alumina’s allowable limit (70 MPa). ROM reached 142.9°, exceeding physiological demands. Stress concentration decreased by 30%, and microcrack propagation risk dropped by 40%. Maximum ceramic deformation (0.074741 mm) was 82.5% lower than that of traditional designs (0.42895 mm), mitigating dislocation risks. Experimental data confirmed surface roughness ≤ 0.1 μm, flatness ≤ 0.6 μm, Vickers hardness of 2513.2 MPa, and fatigue-induced microcrack distribution aligned with FEA predictions.

**Conclusions**

This study demonstrates that biomimetic discrete architectures overcome inherent material limitations in hip prostheses. The design significantly enhances mechanical performance and ROM for high-flexion demands, offering a clinically viable solution for Asian populations and advancing biomimetic approaches in joint arthroplasty.

**Fig. 1 (Abstract P27).**
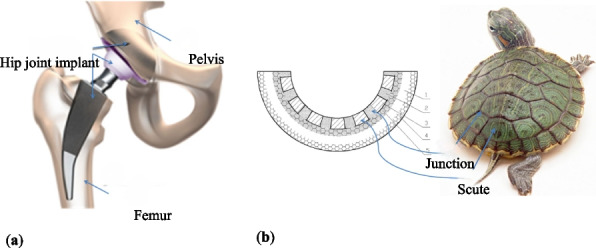
**a** Intracorporeal hip arthroplasty device; **b** Biological turtle, 1 Acetabular backing; 2 Modular interface; 3 Ceramic bearing component; 4–5 Microporous backing liner

**Fig. 2 (Abstract P27).**
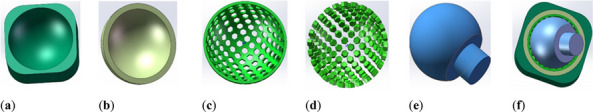
3D explosive assembly diagram of a discrete artificial hip joint. **a** Pelvic acetabular fossa; **b** Acetabular lining back; **c** Model of titanium stent; **d** Ceramic pieces; **e** Femoral head models; **f** Monolithic models

**Fig. 3 (Abstract P27).**
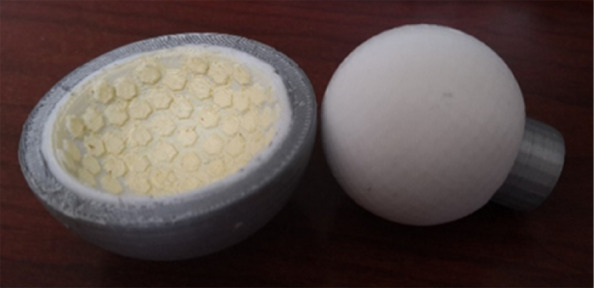
3D-printed physical prototype of a distributed hip joint prosthesis

## P28 Realignment concepts in knee osteotomy: a joint preservation approach for early osteoarthritis

### Van Loi Do^1^, Hung Long Pham^1^, Truong Tin Trinh^2^, Phong Le Dang^1^, Dinh Vinh Pham^1^, Ha Nam Anh Tang^1^

#### ^1^Xuong Khop Viet Clinic, Ho Chi Minh City, Vietnam; ^2^Sante Hospital, Ho Chi Minh City, Vietnam

##### **Correspondence:** Van Loi Do (drvanloi2202@gmail.com)

*Arthroplasty 2026*, **8(1):**P28

**Background**

Osteotomy around the knee is a well-established joint-preserving intervention for early-stage unicompartmental osteoarthritis (OA), especially in younger and active patients. By addressing coronal plane malalignment, procedures like High Tibial Osteotomy (HTO), Double-Level Osteotomy (DLO), and Tibial Condylar Valgus Osteotomy (TCVO) aim to redistribute mechanical load, reduce compartmental stress, and delay the need for arthroplasty.

**Objective**

To illustrate the principles, surgical indications, and outcomes of different realignment osteotomies in managing early-stage knee OA through selected clinical cases.

**Methods**

Three representative cases were presented. Case 1 underwent HTO for medial compartment OA with 9° varus deformity. Case 2, with a more severe multi-level varus deformity (12°), was treated using a DLO combining closed-wedge distal femoral and open-wedge tibial osteotomies. Case 3 underwent TCVO to correct intra-articular malalignment in a patient with residual pain post-PCL reconstruction. Arthroscopy was performed in all cases to confirm cartilage status and guide decision-making.

**Results**

All patients achieved correction to near-neutral mechanical alignment (postoperative HKA ~ 1° valgus), with significant improvement in clinical scores (KOOS increased from 42–63 preoperatively to 76–90 postoperatively). Radiological parameters, including mLDFA, MPTA, and JLCA, were normalized. Patients reported pain relief and returned to daily or moderate activities. No major complications occurred. Use of intraoperative fluoroscopy, protective K-wire, and digital planning enhanced surgical precision and safety.

**Conclusions**

Realignment osteotomy remains a reliable technique for joint preservation in early knee OA. Personalized surgical planning based on comprehensive deformity analysis and intraoperative control is critical for successful outcomes. Selecting the appropriate osteotomy type (HTO, DLO, TCVO) ensures optimal joint biomechanics and functional recovery.

**Consent for publication**

Written informed consent was obtained from the patient for publication of this case report.

## P29 Clinical outcomes of simultaneous bilateral total hip arthroplasty via DAA with robotic assistance for bony ankylosis of the hip: a case report

### Huihuang Chen, Eryou Feng

#### Fujian Medical University Affiliated Union Hospital, Fuzhou, China

##### **Correspondence:** Eryou Feng (fey9001@126.com)

*Arthroplasty 2026*, **8(1):**P29

**Background**

To report a case of simultaneous bilateral total hip arthroplasty (THA) via the direct anterior approach (DAA) under the assistance of an orthopedic robot for the treatment of bilateral bony ankylosis of the hip, and to evaluate the efficacy and safety of this technique in complex bilateral hip reconstruction.

**Methods**

A retrospective analysis was conducted on a 58-year-old female patient with bilateral hip bony ankylosis secondary to childhood pyogenic arthritis. The patient’s medical history, clinical manifestations, diagnostic evaluations, surgical procedures, and follow-up outcomes were documented.

**Results**

The patient had a history of bilateral pyogenic hip arthritis treated conservatively 50 years prior, with progressive hip pain and restricted mobility for over 40 years. Preoperative imaging revealed bilateral hip ankylosis and pelvic obliquity. Physical examination showed a BMI of 27.59 kg/m^2^, spinal lordosis, lumbar scoliosis, bilateral hip flexion contracture (20°), left hip adduction deformity, and restricted hip motion. Preoperative scores included a Visual Analog Scale (VAS) score of 1, left Harris Hip Score (HHS) of 45, Oxford Hip Score (OHS) of 40, right HHS of 60, and OHS of 38. The KUNWU Orthopedic Robotic System (GuSheng Yuanhua, Shenzhen, China) was utilized for preoperative CT-based 3D modeling, implant sizing, and surgical planning. Simultaneous bilateral THA via DAA was performed in the supine position, with a total operative time of 324 min and blood loss of 850 mL. Corail® cementless implants (DePuy Synthes, USA) were placed bilaterally. Postoperative management included prophylactic antibiotics, analgesia, anticoagulation, and early rehabilitation. No special restrictions on position and movement, and the patient was discharged 18 days after surgery. No blood transfusion or complications (infection, deep vein thrombosis, dislocation, nerve injury, or implant loosening, e.g.) occurred. At 3-month follow-up, radiographic evaluation demonstrated optimal acetabular component positioning: left cup anteversion 24.5°, abduction 41.1°; right cup anteversion 16.2°, abduction 41.5°. Hip range of motion improved significantly: left hip flexion 95°, extension 0°, internal rotation 10°, external rotation 15°, adduction 15°, abduction 10°; right hip flexion 100°, extension 0°, internal rotation 10°, external rotation 20°, adduction 10°, abduction 20°. Leg length discrepancy was 0.52 cm, and the combined offset difference was 0.95 cm. Postoperative scores improved to VAS 1, left HHS 76, OHS 26; right HHS 79, OHS 23. The patient resumed unaided ambulation and daily activities without restrictions.

**Conclusions**

Robotic-assisted simultaneous bilateral THA via DAA achieves precise implant placement, minimal invasiveness, rapid recovery, and unrestricted postoperative positioning. This technique demonstrates efficacy and safety in treating complex bilateral hip arthropathy, with no increased surgical risks.

**Consent for publication**

Written informed consent was obtained from the patient for publication of this case report.

## P30 Robot-assisted total hip arthroplasty for adult slipped capital femoral epiphysis with developmental dysplasia of the hip: a case report

### Huihuang Chen, Eryou Feng

#### Fujian Medical University Affiliated Union Hospital, Fuzhou, China

##### **Correspondence:** Eryou Feng (fey9001@126.com)

*Arthroplasty 2026*, **8(1):**P30

**Background**

Total hip arthroplasty (THA) has improved the quality of osteoarthritic patients, yet challenges persist. The robotic arm-assisted system, integrated into THA, aims to improve outcomes. But there is a lack of data concerning the use of robotic devices in complex THA cases.

**Methods**

A retrospective analysis was conducted on a 56-year-old male patient with bilateral slipped capital femoral epiphysis (SCFE) and developmental dysplasia of the hip (DDH) secondary to childhood trauma. The patient’s medical history, clinical symptoms, physical examination, treatment process, and follow-up outcomes were documented.

**Results**

The patient, with a height of 145 cm, a weight of 50 kg, and a BMI of 23.8 kg/m^2^, presented with progressive bilateral hip pain and restricted mobility for over 40 years. Physical examination revealed thoracic scoliosis, bilateral inguinal tenderness, positive “4” sign, and limited left hip range of motion (flexion 80°, extension 0°, adduction 10°, abduction 20°, internal rotation 5°, external rotation 15°). Preoperative assessments included a VAS score of 5, a Harris Hip Score (HHS) of 63, and an Oxford Hip Score (OHS) of 32. Preoperative imaging confirmed severe acetabular dysplasia, reduced bone stock, and narrow femoral medullary canals. Using the KUNWU Orthopedic Robotic System (GuSheng Yuanhua, Shenzhen, China), preoperative planning with thin-slice CT modeling determined optimal implant placement: a 42 mm ACT acetabular cup (Aikang Medical, Beijing) with 43° inclination and 10° anteversion, paired with a 28 mm ceramic femoral head and an SRII modular femoral stem (size 2 stem, 14SB sleeve). The posterolateral approach was taken. Placed the signal tracker, and then made sequential procedures including surgical exposure, anatomical registration, and acetabular reaming. The acetabular cup coverage and positioning were digitally assessed. Upon confirming satisfactory implant positioning parameters, robotic-assisted precise implantation of the acetabular component was performed at the predefined anatomical coordinates. Subsequently, surgical exposure of the proximal femoral canal was achieved. Intraoperatively, slight proximal femoral fissuring occurred during sleeve reaming, which was stabilized with a titanium cable. The hip stability assessment was conducted, and anatomical reconstruction of the hip capsule and short external rotator musculature was performed. The procedure lasted 135 min with 550 mL of blood loss. Postoperatively, the patient received standard prophylactic antibiotics, analgesia, anticoagulation, and rehabilitation and was discharged 7 days after surgery. At 1-month follow-up, imaging demonstrated satisfactory acetabular cup positioning (inclination 40.3°, anteversion 18.5°). The left hip range of motion improved to flexion 95°, extension 0°, adduction 15°, abduction 30°, internal rotation 10°, and external rotation 25°. The VAS score decreased to 3, the Harris Hip Score increased to 79, and OHS improved to 26. A 5.5 cm leg length discrepancy was managed with a 4 cm shoe lift, enabling unassisted ambulation. No blood transfusion or complications such as infection, dislocation, or implant loosening were observed.

**Conclusions**

Robot-assisted THA demonstrates advantages in precise preoperative planning, accurate implant positioning, minimal intraoperative trauma, and rapid postoperative recovery for complex hip pathologies. This technique offers promising clinical outcomes without increasing surgical risks, warranting broader clinical application.

**Consent for publication**

Written informed consent was obtained from the patient for publication of this case report.

## P31 Differences in knee function between patients scheduled to undergo unilateral TKA and bilateral TKA (1-week interval)

### Yu Bin Lee, Seung Ik Cho, Joon Kyu Lee

#### Konkuk University Medical Center, Seoul, Korea

##### **Correspondence:** Joon Kyu Lee (20190546@kuh.ac.kr)

*Arthroplasty 2026*, **8(1):**P31

**Background**

Among patients who have TKA, some have both severe knee arthritis and pain, while some have only one knee problem and a rather mild and painless contralateral knee. Patients with severe knee arthritis and pain are reluctant to walk, and they have reduced daily activities. This leads to leg muscle weakness and decreased knee function. The purpose of this study was to compare the clinical and functional knee statuses of patients who were scheduled to undergo unilateral TKA and bilateral TKA (1-week interval). The hypothesis was that patients scheduled for only unilateral TKA would have better clinical and functional knee status than patients scheduled for bilateral TKA.

**Methods**

One hundred and fifty consecutive patients who underwent either unilateral or staged (1-week interval) bilateral TKAs between March 2021 and January 2023 were subjects of this study. Among patients who had unilateral TKA, 25 patients who had previously received contralateral TKA, or who also had severe (KL grade 4) arthritis and pain on the contralateral knee but were reluctant to receive bilateral TKA at a 1-week interval were excluded. 62 patients received unilateral TKA (Group U) and 63 patients received bilateral TKAs (Group B). Preoperatively, demographic data and clinical scores (Lysholm, IKDC, Tegner, Korean knee score, WOMAC) were checked. Time up and go (TUG) test and 4-m walking time test, which are considered to indicate sarcopenia status, were performed. Isokinetic knee muscle strength (60°/sec for both knee extension and flexion) and hip abductor strength tests were performed. Two thigh point circumferences were measured (5 cm, 15 cm from the upper pole of the patella) to check circumference differences in both thighs.

**Results**

There were no differences in age and sex between the two groups. BMI was significantly lower in Group U compared to Group B (*P* = 0.021). Lysholm score (*P* = 0.016) and IKDC score (*P* = 0.046) were significantly better in Group U; however, there were no differences in KKS, Tegner, and WOMAC scores. There were also no differences in the TUG and the 4-m walking test. Meanwhile, the knee extensor strength difference was significantly higher in Group U compared to Group B (*P* = 0.002), with a significantly higher thigh circumference difference at 15 cm from the upper pole of the patella (*P* = 0.012). The knee flexor and hip abductor strength differences and circumference differences of both thighs at 5 cm from the upper pole of the patella were similar in both groups.

**Conclusions**

Patients scheduled for only unilateral TKA had significantly lower BMI and better Lysholm and IKDC scores compared to patients scheduled for bilateral TKA. They also showed significantly more knee extensor strength and proximal thigh circumference imbalances. However, they did not show better sarcopenic status, and knee flexor and hip abductor strength imbalances were not significantly different compared to patients scheduled for bilateral TKA. Age and sex meant little.

## P32 15 years later: a case of ceramic on metal total hip arthroplasty

### Muhamad Amir Azfar Sahadun, Mohamad Fauzlie Yusof

#### Ministry of Health, Melaka, Malaysia

##### **Correspondence:** Muhamad Amir Azfar bin Sahadun (iamamirazfar@gmail.com)

*Arthroplasty 2026*, **8(1):**P32

**Background**

Total hip arthroplasty (THA) is nowadays the gold standard treatment for patients with hip osteoarthritis for whom non-operative management has been exhausted. It should also be considered for elderly and active patients with neck of femur fractures. The survivorship of implants has dramatically improved over the years, thanks to the advancement of bearing surfaces and good surgical techniques. We would like to report a case of aseptic loosening with femoral stem fracture in uncemented ceramic on metal (CoM) THA.

**Reports**

A 65-year-old gentleman presented to us with gradually worsening right hip pain, which has finally confined him to a wheelchair for ambulation. He had a history of right THA performed in 2010 following complications of right femoral neck fracture. Plain radiographs of the pelvic and hip showed a broken uncemented femoral stem with lucency around the femoral prosthesis. Inflammatory markers taken were within the normal range. He was scheduled for revision of the femoral stem and acetabular liner. Intra-operatively, there was a presence of greyish sterile fluid collection with a loose and broken femoral stem. To our surprise, the bearing surface used during the first surgery was CoM with a tightly fitted acetabular cup and metal liner. A decision was made to proceed only with a cemented femoral stem using metal-on-metal (MoM) articulation. The broken tip of the femoral stem was not extracted and pushed deeper into the femoral canal, distal to the cement plug. At 5 months post-operatively, the patient was able to weight bear without pain using a walking stick. Plain radiographs showed a fitted femoral prosthesis with a good cement mantle. The loose cerclage wire used to secure the tip of the greater trochanter was left in situ as the patient did not complain of any pain.

**Discussions**

CoM was first introduced to reduce loss of metal debris, adhesive, and corrosive wear from MoM, as well as to reduce the risk of fracture and squeaking from CoC articulation. However, its use was rapidly declining due to the fear of ion toxicity and poor implant survivorship, in addition to the limited data on long-term outcomes. Mehta et al. in 2021 reported a reasonable functional outcome with CoM at 9 years of follow-up. Failure of CoM does happen in a similar way to MoM, but at a slower rate. Saracco et al. in 2022 concluded that CoM bearing is safe and reliable at long-term follow-up when the implant is correctly positioned. He also suggested annual monitoring of Co and Cr ion levels, with revision to be considered should the value exceed the normal range.

**Conclusions**

Thorough pre-operative planning is important prior to any revision THA, especially in the event where previous medical records and documentation are not available. In-depth knowledge of bearing surfaces will help the surgeon to produce the best long-term outcome possible for the patient.

**Consent for publication**

Written informed consent was obtained from the patient for publication of this case report.

## P33 Let’s get moving again: primary modified quadriceps V–Y turn down during total knee replacement

### Maniventhan Nachimuthu^1^, Mohd Hezery bin Harun^1^, Mohd Shahrul Azuan Bin Jaffar^2^, Ng Min Guan^2^

#### ^1^Universiti Putra Malaysia, Selangor, Malaysia; ^2^Hospital Tengku Ampuan Rahimah, Klang, Malaysia

##### **Correspondence:** Maniventhan (nmaniventhan@gmail.com)

*Arthroplasty 2026*, **8(1):**P33

**Background**

Local augmentation Quadriceps V–Y turndown technique was first described by Scuderi et al. in 1958, and is often used as salvage for extensor mechanism rupture during TKR. Primary quadriceps V–Y turndown is rarely used for atrophied extensors during TKR. We present such a case encountered at our center.

**Methods**

A 51-year-old patient with over 40 years of left knee stiffness in extension and significant quadriceps atrophy due to childhood trauma presented to us with severe knee pain secondary to osteoarthritis. Adapted to ambulating with a stiff knee since childhood, he sought help to relieve his worsening knee pain. Guided by clinical and radiologic imaging, he was advised to undergo Total Knee Replacement (TKR). During the procedure, a modified quadriceps V–Y turndown as described by Insall in 1983 was done to preserve the vital genicular vessel supply. During the following months, the patient was able to finally restore flexion of his knees, with adequate muscle gain, much to the joy of the patient.

**Results**

In patients with longstanding knee stiffness and quadriceps atrophy and weakness, restoration of motion is often not considered, causing patients to endure lifelong knee pain. TKR augmented with primary quadriceps V–Y turndown offers to improve knee motion by partially restoring quadriceps function, which is critical for rehabilitation after TKR.

**Conclusions**

The quadriceps V–Y turndown during TKR offers a valuable solution for patients with chronic knee stiffness, flexion contractures, and quadriceps atrophy due to long-term osteoarthritis. By addressing both the deformity and muscle weakness, this technique improves knee function and accelerates postoperative recovery, enhancing the overall quality of life for patients with advanced osteoarthritis.

**Consent for publication**

Written informed consent was obtained from the patient for publication of this case report.

## P34 Tibial tracker pin wounds heal safely in robot-assisted total knee replacement

### Kim-Soon Oh^1,2^, Keat Hwa Lee^1^, Khai Oon Ng^1^

#### ^1^Island Hospital, Penang, Malaysia; ^2^MK Faculty of Medicine & Health Sciences UTAR, Malaysia

##### **Correspondence:** Kim-Soon Oh (ohkimsoon@utar.edu.my)

*Arthroplasty 2026*, **8(1):**P34

**Background**

Robot-assisted total knee replacement surgery utilises femoral and tibial pin trackers for navigation. While femoral tracker pins may be placed within the original incision, many surgeons relegate tibial pins distally outside the main incision to allow room for tibial component intramedullary preparation. These additional wounds are potential sources of wound-related complications.

**Methods**

We studied the incidence of tibia pin-related bleeding, wound oozing, and infection in our series of 146 patients with robot-assisted primary total knee replacements. Ages spanned 54 to 83. Four patients had sequential bilateral knee surgeries. Tibial pin calibre measured 3.2 mm (ROSA™ Zimmer, Warsaw, Indiana) in 111 knees, while 35 had 4 mm pins (VELYS™, DePuy Synthes, Warsaw, Indiana). All tibia pin stab incisions (< 7 mm) were outside of the main incision wound using a size-10 surgical scalpel blade. Drilling did not ensnare skin edges and soft tissue. Wound closure utilised interrupted external non-absorbable 000 sutures (Figs. 1 and 2).

**Results**

There were no infections encountered at the end of 90 days. Observed Safety Rate: 143/146 = 97.9%. Exact one-sided binomial test:

- Null hypothesis(H_0_): True Safety Rate ≤ 95%
- Alternative hypothesis (H1): True Safety Rate > 95%
- *P* value 0.063

95% Confidence Interval (Wilson Method) for 143/146 = 93.6% to 99.4%. While the observed safety rate of 97.9% is high, its *P*-value of 0.063 does not achieve statistical significance. However, the confidence interval exceeds 95% (Table 1).

**Discussion**

Tracker pin complications may include infection, muscle injury, nerve injury, and iatrogenic bone fractures. Pin placement and pin calibre have been the subject of recent studies. Several points are pertinent: (1) Incorporating both femoral and tibial pins would have resulted in a long incision wound, increasing morbidity and degrading cosmesis. (2) Bone marrow leak in the thin tibia subcutaneous tissue may be troublesome in younger patients, where fatty replacement has not been extensive. (3) Skin fragility is higher over the anteromedial shin, especially in thin patients. (4) Basic tenets of proper wound closure must be practised. Staples have been discouraged due to a higher infection rate and painful removal, while we have no experience with adhesive strips.

**Conclusions**

No significant wound morbidity was seen in placing tibial tracking pins outside the main incision wound during robot-assisted total knee replacements. Proper wound closure must be done.

**Fig. 1 (Abstract P34).**
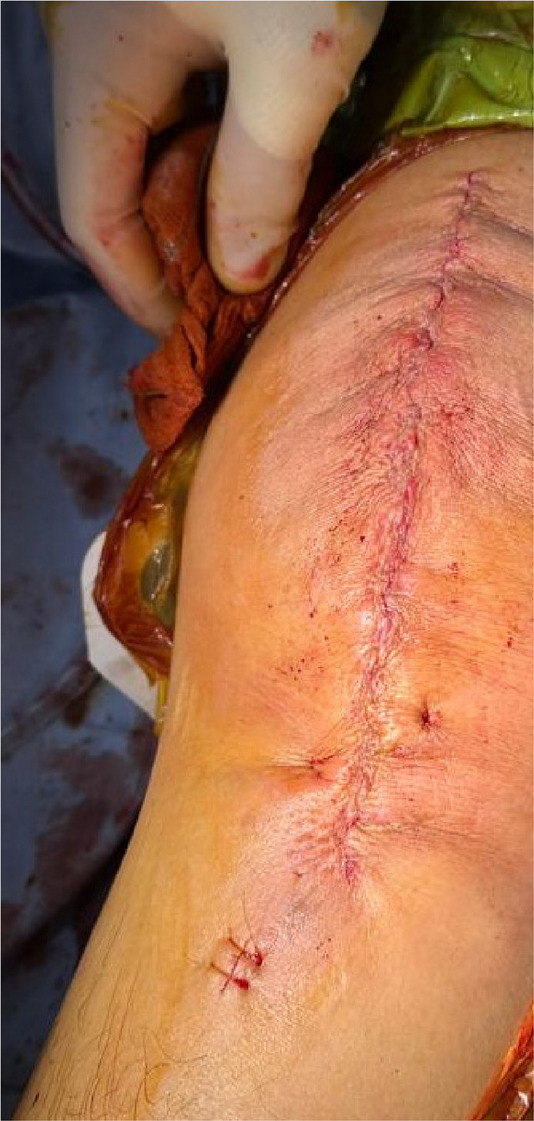
External sutures

**Fig. 2 (Abstract P34).**
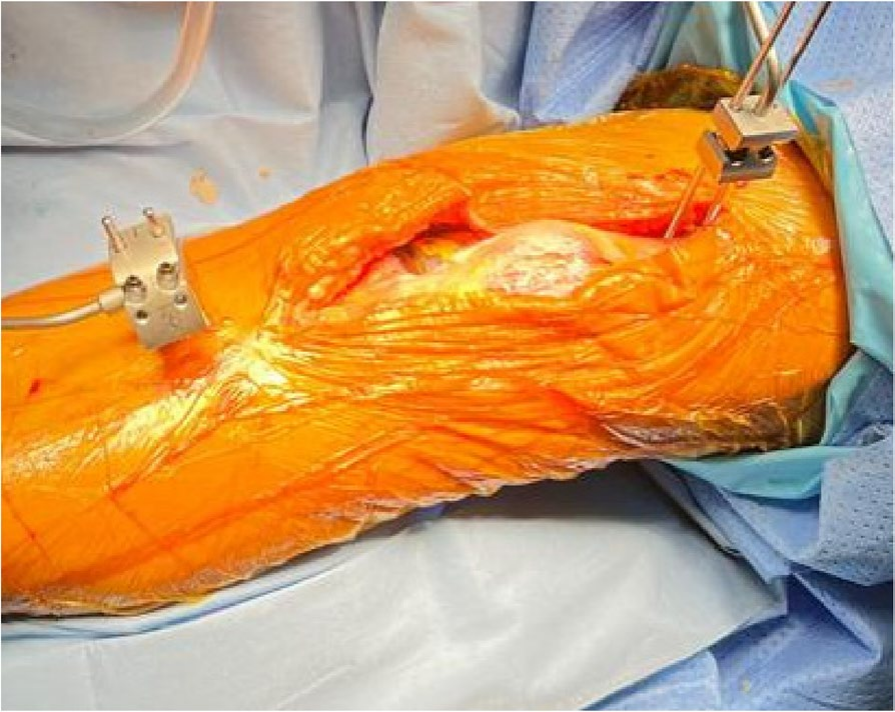
Femoral and tibial pin placements

**Table 1 (Abstract P34).** Outcomes of our distal pin wounds

	**3.2 mm**	**4.0 mm**
Heals within 2 weeks	98	34
Stained dressings	10	1
Oozed out, requiring a change of dressings	2	-
Delayed tibia pin wound healing for more than 2 weeks	1	-
Tibial Pin Infections (within 90 days)	-	-

## P35 Robotic-assisted total knee arthroplasty reduces the need for constrained implant in patients with severe coronal plane deformity: a case series

### Rachel Jingjing Ting, Jade Pei Yuik Ho, Kunalan Ganthel

#### Department of Orthopaedic Surgery, Joint Replacement Unit, Hospital Kuala Lumpur, Malaysia

##### **Correspondence:** Rachel Ting Jingjing (tingjingjingrach@gmail.com)

*Arthroplasty 2026*, **8(1):**P35

**Background**

Total knee arthroplasty (TKA) for patients with severe coronal plane deformity and bony defects is challenging and often necessitates the use of constrained implants. However, the advent of robotic assistance, which provided higher precision in bone resections and better soft tissue balancing, has made it possible to use primary implants in severe varus deformities. We submit two cases of knee osteoarthritis with severe varus deformity in which primary implants have been successfully used with robotic assistance, avoiding the need for constrained implants.

**Report**

**Case A**

A 68-year-old man presented with progressive left knee pain for 13 years. Examination elicited an antalgic gait with varus thrust, 25 degrees of varus deformity, and 20 degrees of fixed flexion deformity. Knee range of movement (ROM) was 20 to 110 degrees with grade 3 laxity of the lateral collateral ligament (LCL). Radiographs showed a marked reduction in tibiofemoral joint space with erosion of the medial tibial condyle and abundant osteophytes, HKA of 29 degrees, lateral distal femoral angle (LDFA) of 90 degrees, and medial proximal tibial angle (MPTA) of 37 degrees. He underwent ROSA robotic-assisted TKA with a standard medial parapatellar approach and recovered uneventfully. Postoperative radiographs exhibited LDFA of 93 degrees and MPTA of 85 degrees.

**Case B**

A 62-year-old man presented with worsening left knee pain for 2 years. Examination revealed a varus thrust gait, 20 degrees varus deformity with ROM 0–100 degrees, and grade 3 laxity of LCL and anterior cruciate ligament (ACL). Radiographs showed obliteration of the medial joint space with erosion of the medial tibial condyle and abundant osteophytes, HKA of 34 degrees, LDFA of 90 degrees, and MPTA of 86 degrees. He underwent ROSA robotic-assisted TKA with a standard medial parapatellar approach and recovered uneventfully. Postoperative radiographs demonstrated LDFA of 97 degrees and MPTA of 82 degrees.

**Discussion**

Knees with severe varus deformity pose difficulties in achieving mechanical alignment, accurate bone cuts, balanced soft tissue tension, and proper implant positioning. Conventional TKA, with its aim to restore the mechanical axis, often requires extensive bone resections and soft tissue releases, which may necessitate the use of a constrained implant to attain stability. Instead, functional alignment achieved via robotic assistance allows precise, patient-specific implant positioning, minimizing soft tissue disruption and reducing the need for constrained implants.

**Conclusion**

Robotic-assisted TKA, through restoration of functional alignment, enables the use of primary implants even in cases with severe coronal plane deformity without resorting to constrained implants.

**Consent for publication**

Written informed consent was obtained from the patient for publication of this case report.

## P36 Anthropometry of the proximal tibia in Malaysian females undergoing knee arthroplasty: implications for tibial component sizing

### Manoosh Raj, Loh Kwong Weng, Khairul Anwar Ayob

#### Department of Orthopaedic Surgery, NOCERAL, Universiti Malaya, Malaysia

##### **Correspondence:** Manoosh Raj (manyuch@yahoo.com)

*Arthroplasty 2026*, **8(1):**P36

**Background**

Knee arthroplasty is a widely accepted intervention for end-stage osteoarthritis (OA), aiming to relieve pain and restore function with minimal complications. The success of this procedure heavily relies on selecting implants that match the patient's anatomy, particularly the tibial component, which is more prone to postoperative issues than the femoral component. Asian populations, especially females, typically have smaller skeletal structures than Western populations, yet most prosthetic designs are based on Western anthropometry. This mismatch can result in poor knee component fit, jeopardizing implant stability and long-term function. The issue is particularly critical in the UKA, where smaller tibial components are linked with a greater risk of periprosthetic fractures. Given the rising number of knee replacements among Malaysian females and the limited data on their tibial dimensions, this study aims to assess the proximal tibia dimensions in this group to support the development of better-fitting, gender-specific implants.

**Methods**

A retrospective study was conducted on 84 knees from 68 female patients who underwent knee arthroplasty between 2019 and 2021 at Universiti Malaya Medical Centre and Universiti Malaya Specialist Centre. Patient demographics and implant types were retrieved from case records. The tibial component dimensions—anteroposterior (AP) and mediolateral (ML)—were documented based on manufacturer sizing charts. The relatively small sample size was due to reduced elective surgeries during the COVID-19 pandemic.

**Results**

The mean age of patients was 67.6 years. Of the procedures, 51 were TKR and 17 were UKR (Table 1). The average tibial component dimensions were 45.74 ± 2.90 mm (AP) and 63.47 ± 6.86 mm (ML) (Table 2). Comparative analysis with international data revealed population-specific variation: Thai females had 46.36 mm (AP) and 72.52 mm (ML); Kenyan, 49.38 mm (AP) and 69.38 mm (ML); Japanese, 50.3 mm (AP) and 71.4 mm (ML); Indian, 43.4 mm (AP) and 65.4 mm (ML); and Caucasian, 45.2 mm (AP) and 70.2 mm (ML). Malaysian females showed consistently smaller ML dimensions, highlighting a potential risk of component overhang or poor coverage when using standard implants.

**Conclusions**

Malaysian females possess smaller proximal tibial dimensions compared to other populations. These anatomical differences underscore the need for population and gender-specific implant designs. Incorporating local anthropometric data into prosthesis design may improve implant fit, reduce complications, and enhance long-term outcomes in knee arthroplasty for Malaysian patients.

**Table 1 (Abstract P36).** Type of knee replacement done on the operated side

Operated Side	Total Knee Replacement (TKR) (*N* = 51)	Unicompartmental Knee Replacement (UKR) (*N* = 17)
Right	26	7
Left	15	4
Bilateral	10	6

**Table 2 (Abstract P36).** Mean tibial component sizes used

Tibial component dimension	
**Parameter**	**Value (mean ± SD)**
Average AP dimension (mm)	45.74 ± 2.90
Average MP dimension (mm)	63.47 ± 6.86

## P37 Temporary relief, permanent pain? Evaluating transcutaneous pulsed radiofrequency in end-stage knee osteoarthritis: a case series

### Amirul Amiruddin, Siti Munira Seri Masran, Fahrudin Che Hamzah

#### HSAAS, UPM, Serdang, Selangor, Malaysia

##### **Correspondence:** Amirul Shaiful Amiruddin (amirulshaiful90@yahoo.com.my)

*Arthroplasty 2026*, **8(1):**P37

**Background**

Knee osteoarthritis (OA) is a leading cause of chronic musculoskeletal pain and impaired quality of life, particularly among the elderly. Management strategies range from pharmacologic interventions and physiotherapy to total knee replacement (TKR). However, many patients are either unfit for surgery or decline it due to personal preferences or comorbidities. Pulsed radiofrequency (PRF) has gained attention as a novel pain modulation technique. Unlike conventional radiofrequency ablation, PRF delivers intermittent electrical pulses, avoiding significant tissue heating and preserving nerve integrity. The transcutaneous application of PRF (TCPRF) offers a needle-free, outpatient-friendly alternative. Taverner et al. demonstrated significant short-term pain relief in patients awaiting TKR following TCPRF treatment. Sluijter et al. proposed the RedoxPRF hypothesis, which suggests PRF exerts its effects at the cellular level by modulating oxidative stress and interrupting inflammatory pathways—offering a theoretical rationale for its use in OA-related pain.

**Methods**

A retrospective case series was conducted in our orthopaedic outpatient clinic at *HSAAS, UPM*. Six patients (4 female, 2 male; aged 69–84 years) with Kellgren–Lawrence grade 3–4 knee OA were included. All had declined surgical intervention and consented to undergo TCPRF. Each patient received a single session of TCPRF using bipolar self-adhesive electrodes placed over standard anatomical landmarks. Parameters included 15 min of stimulation at 80 V, pulse width 20 ms, and frequency of 2 Hz, delivered via the Neurotherm NT 1000 generator. Pain intensity was assessed using the Visual Analog Scale (VAS) at baseline, 1 week, and 4 weeks post-treatment. Subjective reports of functional improvement were also extracted from clinical notes.

**Results**

Baseline VAS scores ranged from 6 to 9. At 1 week post-treatment, 3 patients (50%) experienced a 1–2 point reduction in VAS, while the remainder showed no significant change. By week 4, all patients reported a return of pain to baseline levels. No adverse events or complications were observed. Those who experienced short-term relief noted temporary improvements in daily activities such as walking, though these effects were not sustained.

**Conclusions**

TCPRF is a safe, non-invasive modality that may offer short-term pain relief in patients with advanced knee OA. However, its therapeutic benefit appears limited in end-stage disease, where structural degeneration predominates. Future studies should assess its potential in earlier OA stages and explore whether repeated or adjunctive therapies can improve and sustain outcomes.

## P38 White tongue, failing knee: fungal PJI unveiling Candida tropicalis

### Leong Jin Kai^1^, Samsher Singh Nahal^1^, Wong Shu Kok^1^, Kishore Naath Muniandy^1^, Teh Hak Lian^1^, Veenesh Selvaratnam^2^

#### ^1^Joint Replacement Unit, Department of Orthopaedic Surgery, Hospital Raja Permaisuri Bainun, Ipoh, Malaysia; ^2^Joint Reconstruction Unit, National Orthopaedic Centre of Excellence for Research and Learning (NOCERAL), Department of Orthopaedic Surgery, Faculty of Medicine, University Malaya, Kuala Lumpur, Malaysia

##### **Correspondence:** Teh Hak Lian (drteh1984@yahoo.com)

*Arthroplasty 2026*, **8(1):**P38

**Background**

Prosthetic joint infection (PJI) is a dreaded complication following total joint arthroplasty, including total knee replacement (TKR). Even a minimal microbial burden can precipitate infection due to the biofilm-forming potential of pathogens. While bacterial organisms are the predominant culprits, fungal PJIs are exceedingly rare and often devastating. Fungal infections are typically associated with multifactorial risk factors, including immunosuppression, prolonged antibiotic exposure, and underlying autoimmune conditions.

**Report**

An 81-year-old male, fully independent in activities of daily living, with a background of bronchial asthma, dyslipidemia, benign prostatic hyperplasia, iron-deficiency anaemia, bilateral apical fibrosis with granuloma, and previous bilateral TKRs (left TKR in 2022; right TKR in 2023), was admitted under the geriatric unit for acute gouty arthritis and dehydration-related hypernatraemia and hypercalcemia. During the admission, he complained of right knee pain and was referred to the joint replacement unit for evaluation. On clinical examination, the right knee was warm with significant medial and lateral joint line tenderness. Passive motion was limited by pain, although distal perfusion and sensation remained intact. A working diagnosis of PJI was made. Notably, the patient also had a whitish tongue, raising the suspicion of fungal infection. The infectious disease team started him on oral nystatin for oral candidiasis treatment. Synovial fluid aspiration of the right knee grew Candida tropicalis. The patient subsequently underwent a first-stage revision with insertion of an articulating antibiotic cement spacer (Amphotericin B 200 mg per 40 g of cement). Intraoperatively, a medial parapatellar approach was employed. Findings included loosening of the medial tibial component, sloughy and inflamed soft tissue, minimal pus, and biofilm formation on implant surfaces. A complete synovectomy was performed. Intraoperative tissues and synovial fluid cultures all grew Candida tropicalis. He was started on Fluconazole and planned to continue for at least 6 months before second stage replantation. Second-stage surgery will only be performed once the infection markers have normalized and the patient is clinically free of infection.

**Discussion**

This case underscores a rare but serious fungal knee PJI caused by Candida tropicalis, in the setting of concomitant oral candidiasis. This highlights the importance of considering systemic fungal involvement in atypical PJIs and the role of multidisciplinary evaluation in uncovering potential occult comorbidities.

**Conclusions**

Aggressive surgical debridement, antifungal therapy, and systemic assessments remain essential in managing fungal PJIs.

**Consent for publication**

Written informed consent was obtained from the patient for publication of this case report.

## P39 Hybrid total hip arthroplasty in acetabular defect Paprosky IIB using a combined autologous structural bone graft and impaction of bone graft technique

### Thow Soon Yong, Mohd Shukri Omar, Ng YH, Sa’adon Ibrahim

#### Hospital Sultan Ismail, Johor Bahru, Malaysia

##### **Correspondence:** Thow Soon Yong (soonyong@yahoo.com)

*Arthroplasty 2026*, **8(1):**P39

**Background**

Acetabular defects are frequently encountered in total hip replacement. The goals of reconstruction are to reconstitute acetabular bone stock, ensure secure fixation of the acetabular component, restore the hip centre, and restore leg length. Impaction bone grafting (IBG) is a reliable method to restore acetabular bone-stock deficiency and to form a durable scaffold for the acetabular implant, incorporated into the host bone, and undergo gradual remodeling. (1) Here we describe a case using combined autologous structural bone grafts and bone graft impaction technique for a hybrid total hip arthroplasty with an acetabular defect Paprosky IIB secondary to tuberculosis arthritis.

**Case report**

This is a 38-year-old gentleman. No co-morbid. Presented with right hip pain for 2 years and worsening for 4 months. He was unable to ambulate due to pain. He underwent a CT-guided biopsy of the right hip after an MRI of the right hip; the HPE result was granulomatous inflammatory. HRCT thorax done, and features are in favor of bilateral pulmonary tuberculosis changes. He was treated for pulmonary and extra-pulmonary tuberculosis and started anti-TB treatment for 1 year. Prior to the operation, a CT pelvis was done to evaluate the acetabular bone loss. He underwent a hybrid right total hip replacement after completion of anti-tuberculosis treatment. Intraoperative findings: AVN head of the right femur, and there is cavitation over the superolateral and superomedial of the acetabulum, with a partial defect over the superior rim. Chondral bone over the femoral head was removed, and the head and neck were cut into blocks and small pieces. The defects are filled with autologous structural bone graft and fixed with screws. Bone graft impaction done, cement and acetabular component inserted. Postoperatively, the patient was told not to weight for 6 weeks and started on an anti-osteoporosis agent. Patient started to weight bearing 6 weeks post op and was able to ambulate without aid at 3 months post op.

**Discussion**

Abu-Zeld et al. reported 50 patients with IBG for contained acetabular defects in THA; more than 70% of patients achieved complete graft incorporation. (1) Kwong-ting et al. reported 8 patients using structural bone graft fixed with two screws in acetabular reconstruction, the survival rate for the grafts was 100%, with either revision or loosening at the end point. (2) Structural bone graft has advantages, including anatomical placement over the acetabular defect, provision of support for acetabular components, and reconstitution of bone stock that is beneficial for future revision surgery. Using the impaction bone grafting technique with cement can improve the strength of the bone defect area, allowing stable placement of the acetabular component.

**Conclusions**

Our case proved that combining structured bone grafts fixed with screws, followed by impaction bone graft and cementing, the acetabular component is stable, and the bone grafts are completely incorporated into the body. Bone health is also an important factor to consider to prevent migration of the components.

**Consent for publication**

Written informed consent was obtained from the patient for publication of this case report.

## P40 Biologic augmentation in early idiopathic osteonecrosis of the femoral head: long-term outcomes of core decompression with bone marrow aspirate concentrate and teriparatide

### Puravi Ganesh Shanmugam^1^, Khairul Anwar bin Ayob^1^, Azhar Mahmood Merican^2^

#### ^1^Universiti Malaya, Kuala Lumpur, Malaysia; ^2^Universiti Malaya Specialist Center, Kuala Lumpur, Malaysia

##### **Correspondence:** Puravi Ganesh Shanmugam (puravi.ganesh@gmail.com)

*Arthroplasty 2026*, **8(1):**P40

**Background**

Idiopathic osteonecrosis of the femoral head (ONFH) affects young adults and, if untreated, can progress to femoral head collapse and early arthroplasty. Joint-preserving techniques such as core decompression combined with biologic augmentation like bone marrow aspirate concentrate (BMAC) and anabolic agents such as teriparatide offer promising alternatives in early-stage disease.

**Methods**

Two female patients with ARCO Stage I–II ONFH underwent core decompression and injection of 10 cc of BMAC derived from the iliac crest. Patient A had isolated left hip involvement (ARCO Stage II) confirmed by CT, while patient B presented with bilateral ONFH: right hip (ARCO Stage II) and left hip (Stage I–II) confirmed via MRI. Each hip was treated surgically in a staged manner and supplemented with adjunctive teriparatide therapy (in patient B). Long-term follow-up assessments included clinical examination and serial radiographs at 8- and 11-year post-op, respectively.

**Results**

Both patients demonstrated complete resolution of pain, with full return to function within 6 months of the procedure. Radiographic follow-up showed no evidence of femoral head collapse, joint space narrowing, or degenerative changes. No further surgical interventions were required after initial surgery.

**Conclusions**

Core decompression augmented with BMAC with or without adjunctive teriparatide may offer durable joint preservation in early idiopathic ONFH. These two long-term cases demonstrate the potential of regenerative and anabolic strategies to halt disease progression and preserve native hip function in ARCO Stage I–II patients.

**Consent for publication**

Written informed consent was obtained from the patient for publication of this case report.

